# Brasilianoids A–F, New Meroterpenoids From the Sponge-Associated Fungus *Penicillium brasilianum*

**DOI:** 10.3389/fchem.2018.00314

**Published:** 2018-07-27

**Authors:** Jianping Zhang, Bochuan Yuan, Dong Liu, Shuang Gao, Peter Proksch, Wenhan Lin

**Affiliations:** ^1^State Key Laboratory of Natural and Biomimetic Drugs, Institute of Ocean Research, Peking University, Beijing, China; ^2^Institute of Life Sciences, Wenzhou University, Wenzhou, China; ^3^Institute für Pharmazeutische Biologie und Biotechnologie, Heinrich-Heine-Universität üsseldorf, Düsseldorf, Germany

**Keywords:** sponge-associated fungus, *Penicillium brasilianum*, brasilianoids A–F, stimulation of filaggrin and caspase-14, inhibition of NO production

## Abstract

3,5-Dimethylorsellinic acid (DMOA) derived meroterpenoids comprise an unique class of natural products with diverse scaffolds and with a broad spectrum of bioactivities. Bioinformatics analysis of the gene clusters in association with the qRT-PCR detection of the amplification of two key genes led to speculate that the sponge associated fungus *Penicillium brasilianum* WZXY-m122-9 is a potential producer of meroterpenoids. Chromatographic separation of the EtOAc extract of this fungal strain on a large-scale fermentation resulted in the isolation of six new DMOA-related meroterpenoids with trivial names of brasilianoids A–F (**1**-**6**), together with preaustinoid D and preaustinoid A2. The structures were determined by extensive analyses of spectroscopic data, including the X-ray diffraction and the ECD data for configurational assignment. Brasilianoids A and F showed an unprecedented skeleton with a γ-lactone in ring A, while brasilianoids B–C featured a 7/6/6/5/5 pentacyclic ring system finding in nature for the first time. The biosynthetic relationship among the isolated compounds was postulated. Compound **1** significantly stimulated the expression of filaggrin and caspase-14 in HaCaT cells in dose-dependent manner, while compounds **2** and **3** showed moderate inhibition against NO production in LPS-induced RAW 264.7 macrophages.

## Introduction

**Figure 1:**
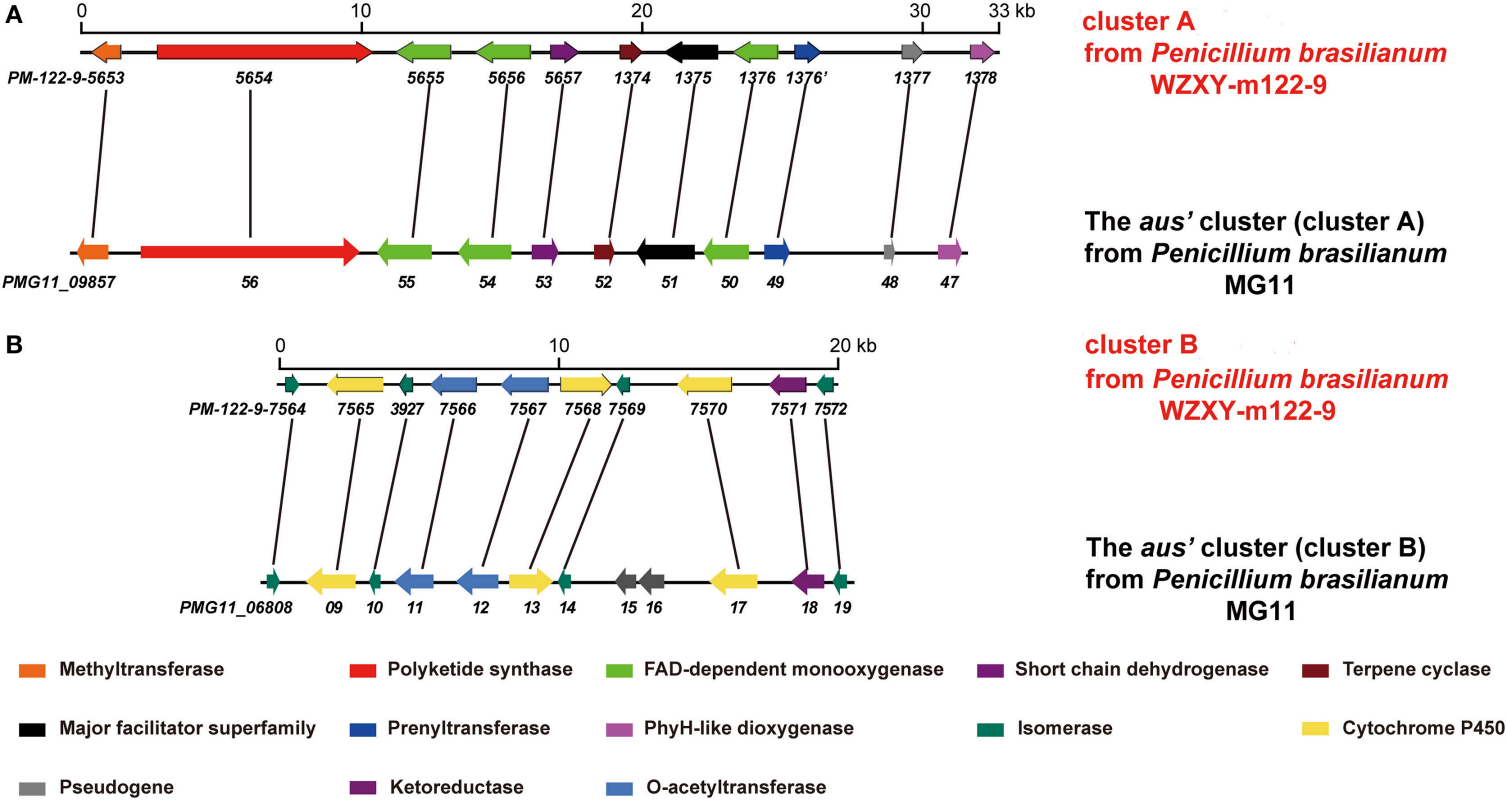
Comparison of the gene clusters between *P. brasilianum* WZXY-m122-9 and *P. brasilianum* MG11. **(A)** Comparison of the genes in cluster A of *P. brasilianum* WZXY-m122-9 with the *aus*'s genes (cluster A) from *P. brasilianum* MG11. The homologous genes between the gene clusters were linked with lines. **(B)** Comparison of the genes in cluster B of *P. brasilianum* WZXY-m122-9 with the *aus*'s genes (cluster B) from *P. brasilianum* MG11. The homologous genes between the gene clusters were linked with lines. *PM-122-9-5653* and others mean the genes in *P. brasilianum* WZXY-m122-9.

**Figure 2:**
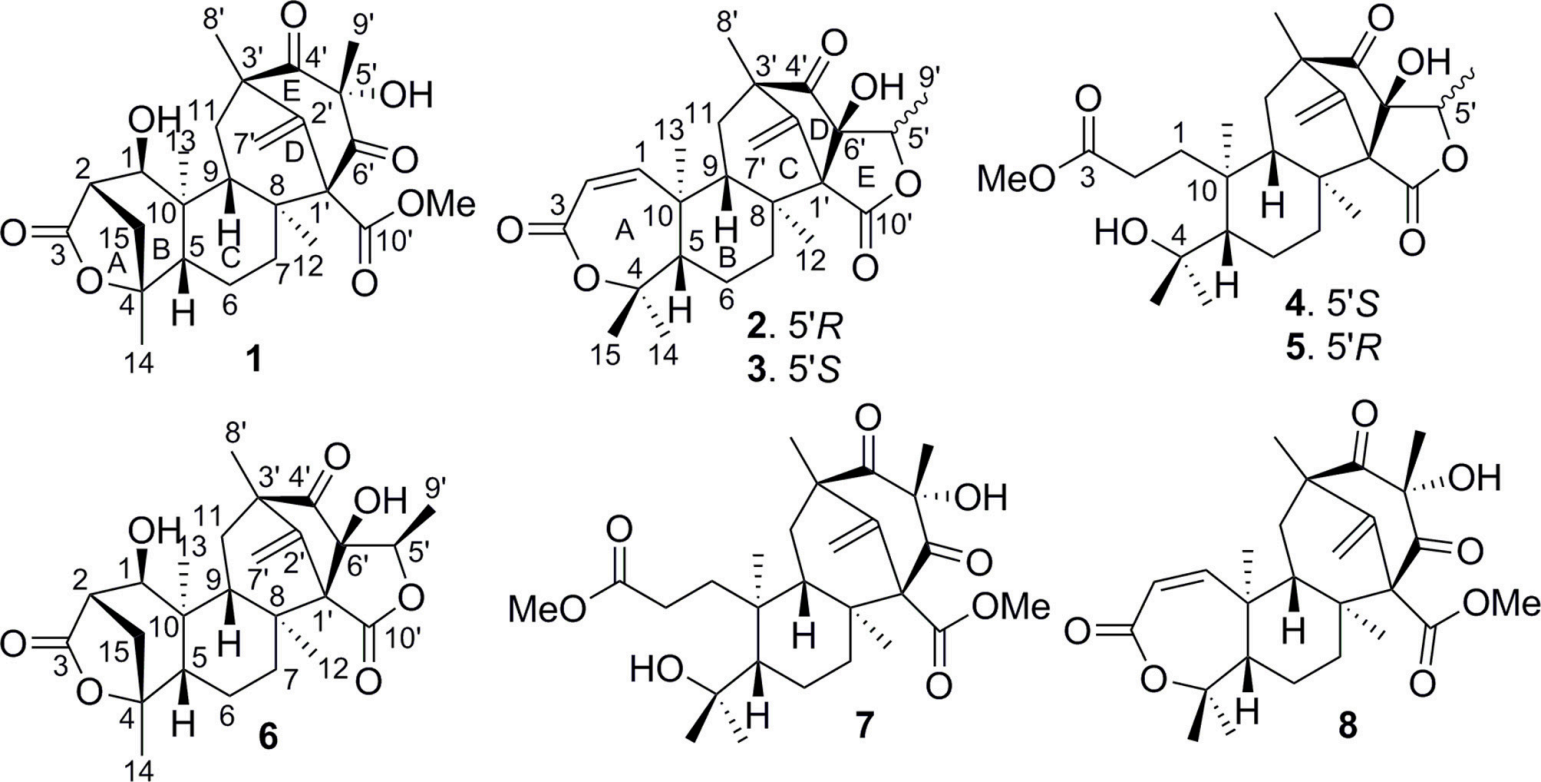
Structures of **1–8**.

The fungal meroterpenoids as the fascinating hybrid natural products are widely distributed in marine environments with diverse molecular architectures, that are assembled by terpene moieties with other precursors such as polyketide unit by various biosynthetic pathways (Iida et al., 2008; Silva et al., 2011; Guo et al., 2012; Qi et al., 2017). Among the fungus-derived meroterpenoids, a polyketide-terpenoid biosynthetic pathway that has C-alkylation of 3,5-dimethylorsellinic acid (DMOA) with farnesyl pyrophosphate (FPP) generated more than 100 secondary metabolites with a number of unique scaffolds (Geris and Simpson, 2009; Matsuda and Abe, 2016). The biogenetic pathways of these typical natural products have been extensively investigated, uncovering a series of synthetic gene clusters and functional enzymes (Lo et al., 2012; Matsuda et al., 2016; Mori et al., 2017). The structure variation of the DMOA-based meroterpenoids was attributed to sequential cyclization, complex oxidative ring rearrangement, and recyclization. Based on the carbocyclic frameworks, the DMOA-FPP derived meroterpenoids can be classified into seven subtypes. Andrastins with a 6,6,6,5-*tetra*-carbocyclic skeleton are the potent inhibitors of RAS proteins, which are important for controlling cell division and the development of cancer (Nielsen et al., 2005). Terretonin-type congeners possessing a δ-lactone in ring D are originated from terrenoid (andrastin-type) by D-ring expansion and unusual rearrangement of the methoxy group (Matsuda et al., 2015). Berkeleyone-type (or protoaustinoid-tyep) derivatives as the caspase-1 inhibitors are the meroterpenoids representing a series of unique and functionalized chemical scaffolds, which are characterized by the presence bicyclo[3.3,1]nonane or its rearranged bicyclo[3,2,1]octane unit in rings C and D. (Stierle et al., 2011), and are derived by the same intermediate as for andrastins with different rearrangement. Austinol and its analogues featured a pentacyclic scaffold with a spiro-δ-lactone in ring A and a γ-lactone in ring E, that were derived from protoaustinoid through oxidation and ring rearrangement (Matsuda et al., 2013). Chrysogenolides are a group of DMOA-based compounds with a unusual seven-numbered ring B, which exhibited the inhibition of nitric oxide production (Qi et al., 2017), while anditomin analogues featured the presence of a unique and highly oxygenated bridged-ring system (Matsuda et al., 2014). Fumigatonin and novofumigatonin are an additional subtype containing highly oxidized and complicatedly condensed ring system (Okuyama et al., 2008; Rank et al., 2008). These meroterpenoids have been reported to possess a range of biological activities (Rank et al., 2008; Zhang et al., 2012). *Penicillium brasilianum* is a remarkable microorganism with great potential to produce secondary metabolite with a variety of carbon skeletons and with interesting biological activities. Fungal strains of *P. brasilianum* are mostly isolated from terrestrial sources. They produced diverse metabolite scaffolds including meroterpenenes, polyketides, alkaloids, and cyclopeptides (Bazioli et al., 2017). In the course of our search for new terpenoids with pharmaceutical bioactivity, a sponge-associated fungal strain, *P. brasilianum* WZXY-m122-9, was selected due to the same fungal species has been reported to produce meroterpenoids with chemical diversity and novelty (Matsuda et al., 2016; Bazioli et al., 2017). Anti-SMASH genome sequence analysis revealed that two gene clusters (clusters A and B) in WZXY-m122-9 are highly identical to the *aus'* clusters (Figure 1), that playing key role for the biosynthesis of DMOA-derived meroterpenoids in *P. brasilianum* MG11 (Matsuda et al., 2016). qRT-PCR detection showed that prenyltransferase gene (PM-122-9_1376) and terpene cyclase gene (PM-122-9_1374) in clusters A and B were highly expressed. These findings suggested WZXY-m122-9 strain to be a potential producer of terpenoids. Chromatographic separation of the EtOAc extract of the cultured fungus on a large scale resulted in the isolation of eight meroterpenoids, including six new compounds namely brasilianoids A-F (**1**-**6**) and known analogues preaustinoids D (**7**) and A2 (**8**) (Figure 2).

## Materials and methods

### General experimental procedures

Optical rotations were measured with an Autopol III automatic polarimeter. IR spectra were measured with a Thermo Nicolet Nexus 470 FT-IR spectrometer. 1D and 2D NMR spectra were recorded on a Bruker Avance-400/600FT NMR spectrometer using TMS as internal standard. HRESIMS data were acquired on a Bruker APEX IV 70 eV FT-MS spectrometer. ESIMS data were obtained on a Finnigan MAT 95 mass spectrometer. The column chromatographic substrates included silica gel (200–300 mesh) and HF254 silica gel for TLC (Qingdao Marine Chemistry Co. Ltd.), Sephadex LH-20 (18–110 μm; Pharmacia Co., Ltd.); ODS (50 μm; YMC, Milford, MA). Semipreparative HPLC separation was performed with an Alltech instrument (426-HPLC pump) equipped with an UV detector at 210 nm and using a Prevail-C_18_ column (Semipreparative, 5 μm). X-ray data were measured with a Bruker SMART APEX-II DUO instrument.

### Fungal strain and identification

The fungus *P. brasilianum* WZXY-m122-9 was isolated from an unidentified marine sponge, which was collected in July 2016 from Weizhou Island in the South China Sea, and was identified by microscopic examination and 18S rDNA ITS sequence's BLAST in GenBank (GenBank accession number HM469396). A voucher specimen (WZXY-m122-9) was deposited at the State Key Laboratory of Natural and Biomimetic Drugs, Peking University, China.

### Genome sequencing and analysis

Genome sequencing of *P. brasilianum* WZXY-m122-9 was performed by Sangon Biotech (Shanghai) Co., Ltd. (Shanghai, China) with an Illumina HiSeq 2000 system. Sequence assembly was performed with SPAdes version 3.5.0 (http://cab.spbu.ru/software/spades/) to yield 1,367 contigs covering approximately 34.7 Mb. Gene prediction was then performed with Prokka (https://github.com/tseemann/prokka). Anti-SMASH (antibiotics and secondary metabolite analysis shell) analysis of genome sequences was performed to detect secondary metabolite gene clusters, and accurate gene cluster alignment was performed manually by comparisons with homologous genes found in the NCBI database. Anti-SMASH of WZXY-m122-9 genome analysis displayed that two gene clusters namely cluster A and cluster B, showed high identity compared with *aus'* clusters that had been reported in *P. brasilianum* MG11 (Matsuda et al., 2016).

### Quantitative RT-PCR for gene expression

Degenerate primers were designed according to the conserved sequences of prenyltransferase (PM-122-9_1376′) and terpene cyclase (PM-122-9_1374) genes within clusters A and B, and the length of PCR products were 805 bp and 529 bp, respectively. The gene expression levels were detected by qRT-PCR. Total RNA of WZXY-m122-9 was obtained from the rice culture medium, and cDNA was synthesized from 1 μg of total RNA in a volume of 20 μL using TransScriptIIAll-in-One first-strand cDNA synthesis super mix (Transgene) according to the manufacturer's instructions. 0.4 μL cDNA, forward primer (10 μM), reverse primer (10 μM) and 10μL 2 × TransStart Top Green qPCRSuperMix (Transgene) were used in subsequent RT-PCR reactions with supplement ddH_2_O to 20 μL. The specific primers included actin-F (5′-ACCTGCTCTGCGACTACAAC-3′), actin-R (5′-ACACCGCCCTCATAATAAAG-3′), PM-122-9_1376′-F (5′-CCACCAAAGGGGATTACCA-3′), PM-122-9_1376′-R (5′-GAGCAGAAATGTCGCAGGAA-3′), PM-122-9_1374-F (5′-CGGTAGGATGGTCGGTCAAC-3′), PM-122-9_1374-R (5′-ACGGCGGAGTGTAGGAAGAA-3′). Each primer pair gave a single PCR product. The β*-actin* gene was treated as an internal reference gene, optimized PCR conditions were 94°C for 30 s; 45 cycles of 94°C for 5 s; 60°C for 15 s; and 72°C for 10 s; followed by dissociation stage. Three parallel tests for each reaction and recording their respective Ct values. According to amplification curves, every sample was different from no template control (NTC), which indicated that the genes were amplified from cDNA template. Under current culture conditions, the *aus*′ gene cluster was expressed in the plasmids of WZXY-m122-9, and the expression level of prenyltransferase (PM-122-9_1376′) and terpene cyclase (PM-122-9_1374) genes was higher than β*-actin* due to Ct_1376_ and Ct_1374_ less than Ct_actin_.

### Fermentation of the fungus

The fermentation was carried out in 40 Fernbach flasks (500 mL), each containing 80 g of rice. Distilled H_2_O (100 mL) was added to each flask, and the contents were soaked overnight before autoclaving at 15 psi for 30 min. After cooling to room temperature, each flask was inoculated with 5.0 mL of the spore inoculum (10^7^/mL) and incubated at 25°C for 25 days.

### Extraction and isolation of metabolites

The fermented material was extracted with EtOAc (3 × 1 L), successively. The EtOAc extract was evaporated to dryness under reduced pressure to afford a crude residue (20.5 g). The crude extract was suspended in 90% MeOH-H_2_O and then extracted with petroleum ether (PE) for three times, while the MeOH layer was concentrated under vacuum to obtain MeOH soluble extract (8.0 g). Part of the MeOH fraction (4 g) was then subjected to silica gel (200–300 mesh) vacuum liquid chromatography with gradient elution using PE-EtOAc (from 8:1 to 0:1, v/v) to obtain four fractions (FA-FD). FC (0.9 g) was detected by ^1^H NMR spectrum, showing the signals featured meroterpenoid analogues, and it was chromatographed over an RP-C_18_ column eluting with MeOH-H_2_O (65:35, v/v) to afford six subfractions (FCa–FCf). FCb (60 mg) was separated on a semipreparative RP-C_18_ HPLC column using MeOH-H_2_O (50:50, v/v, 2 mL/min) to yield compounds **7** (8 mg) and **1** (4 mg). FCc (110 mg) was purified by a semipreparative RP-C_18_ HPLC column eluting with MeCN-H_2_O (40:60, v/v, 2 mL/min) to afford compounds **2** (6 mg), **3** (6 mg) and **6** (2 mg). FCd (70 mg) was separated using a semipreparative RP-C_18_ HPLC column eluting with MeOH-H_2_O (55:45, v/v, 2 mL/min) to afford **4** (5.0 mg), **5** (5.0 mg), and **8** (45 mg).

Brasilianoid A (**1**) (methyl (1*R*, 2*S*, 5*R*, 5a*S*, 7a*S*, 8*R*, 10*S*, 12*R*, 13a*R*, 13b*S*)-1, 10-dihydroxy-5,7a,10,12,13b-pentamethyl-14-methylene-3,9,11-trioxotetradecahydro-2,5:8,12-dimethanocycloocta[3,4]benzo[1,2 -c]oxepine-8(5H)-carboxylate). White amorphous powder; ${\left[\alpha \right]}_{\text{D}}^{20}$ −22 (*c* 0.5, MeOH); UV (MeOH) λ_max_ (log ε) 203 nm; IR v_max_ (KBr) 3,419, 2,985, 2,935, 1,754, 1,720, 1,708, 1,662, 1,227, and 1,025 cm^−1^. ^1^H and ^13^C NMR data, see Tables 1, 2; HRESIMS *m/z* 473.2176 [M–H]^−^ (calcd for C_26_H_33_O_8_, 473.2175).

**Table 1:** ^1^H NMR data of **1**–**6** (DMSO-*d*_6_, δ ppm, *J* in Hz).

**H**	**1**	**2**	**3**	**4**	**5**	**6**
1	3.47, t (4.2)	6.30, d (12.6)	6.31, d (12.2)	1.32, m; 2.43, m	1.33, m; 2.43, m	3.50, t (4.5)
2	2.50, dd (4.2, 4.9)	5.76, d (12.6)	5.76, d (12.2)	1.72, m; 2.42, m	1.73, ddd (4.0, 12.5, 15.0) 2.42, m	2.54, ddd (4.5, 5.0, 10.1)
5	1.24, dd (1.7, 11.7)	2.02, br d (12.1)	1.94, dd (2.7, 12.7)	1.29, m	1.29, dd (2.0, 12.0)	1.67, dd (2.0, 12.0)
6	1.43, dt (3.2, 13.0) 1.61, br ddd (1.7, 2.0, 13.0)	1.65, m 1.68, m	1.62, ddd (2.7, 3.0, 13.0) 1.75, ddt (3.0, 12.7, 13.0)	1.49, m; 1.51, m	1.48, m; 1.50, m	1.51, m 1.62, m
7	1.85, dt (3.2, 12.0) 2.07, ddd (2.0, 3.2, 12.0)	1.70, m 2.23, ddd (3.0, 12.0, 12.5)	1.63, dt (3.0, 13.0) 2.30, dt (3.0, 13.0)	1.53, m 2.23, dt (3.0, 12.5)	1.54, m 2.15, dt (4.0, 12.3)	1.63, m 2.16, dt (4.1, 12.7)
9	1.50, dd (3.6, 12.9)	1.99, dd (2.0, 12.5)	2.10, br dd (4.1, 13.7)	2.07, br dd (5.8, 12.1)	1.92, dd (8.9, 13.0)	2.59, dd (3.8, 13.7)
11	1.68, t (12.9) 1.74, dd (3.6, 12.9)	1.84, dd (12.5, 13.0) 1.87, dd (2.0, 13.0)	1.84, t (13.7) 1.89, dd (4.1, 13.7)	1.60, dd (5.8, 13.0) 1.63, dd (12.1, 13.0)	1.48, dd (8.9, 12.0) 1.57, dd (12.0, 13.0)	1.56, dd (11.7, 13.7) 1.93, dd (3.8, 11.7)
12	1.17, s	1.18, s	1.20, s	1.12, s	1.11, s	1.14, s
13	0.76, s	1.08, s	1.08, s	0.87, s	0.88, s	0.78, s
14	1.29, s	1.35, s	1.35, s	1.10, s	1.10, s	1.30, s
15	1.81, br dd (4.9, 11.2) 2.24, d (11.2)	1.30, s	1.31, s	1.13, s	1.13, s	1.87, dd (5.0, 11.1) 2.29, brd (11.1)
5′		4.30, q (6.4)	4.46, q (7.2)	4.42, q (7.2)	4.29, q (6.4)	4.21, q (6.4, 6.4, 6.4)
7′	4.67, s; 5.21, s	4.86, s; 5.09, s	4.93, s; 5.14, s	4.89, s; 5.08, s	4.82, s; 5.04, s	4.81, s; 5.03, s
8′	1.34, s	1.20, s	1.16, s	1.12, s	1.16, s	1.16, s
9′	1.33, s	1.14, d (6.4)	1.03, d (7.2)	1.03, d (7.2)	1.15, d (6.4)	1.15, d (6.4)
OMe	3.57, s			3.57, s	3.57, s	
OH-1	5.16, d (4.2)					5.16, d (4.5)
OH-4′				4.09, s		
OH-5′	6.28, s				6.76, s	
OH-6′		6.95, s	7.09, s	7.04, s		6.68, s

**Table 2:** ^13^C NMR data of **1**–**6** (DMSO-*d*_6_, δ ppm).

**C**	**1**	**2**	**3**	**4**	**5**	**6**
1	70.0	155.0	155.6	34.3	34.3	70.3
2	48.0	119.8	120.1	27.7	27.7	48.3
3	177.5	166.9	167.0	174.3	174.3	177.8
4	86.8	85.1	85.2	74.3	74.3	87.0
5	48.3	55.5	55.7	51.1	51.2	48.2
6	18.6	22.7	23.0	22.5	22.3	19.4
7	32.7	32.6	32.6	32.7	32.7	33.6
8	47.0	41.3	42.1	42.2	41.3	41.5
9	44.0	47.4	47.2	42.5	42.8	43.1
10	42.6	44.2	44.0	41.1	41.1	42.7
11	37.5	39.4	39.7	39.0	38.6	38.2
12	18.1	18.8	18.5	18.5	18.6	20.2
13	17.6	15.7	15.6	20.3	20.4	17.9
14	23.0	26.3	26.5	28.0	27.9	23.3
15	40.7	32.2	32.3	33.9	34.0	41.1
1′	72.6	66.5	65.0	65.7	67.2	66.8
2′	144.1	147.6	148.8	149.4	148.1	148.4
3′	50.5	55.3	55.7	55.6	55.2	55.3
4′	208.0	213.4	215.2	215.9	214.0	213.0
5′	76.9	76.4	83.5	83.3	76.1	76.2
6′	206.0	90.8	90.3	90.3	90.9	90.7
7′	112.3	106.5	107.6	106.8	105.8	105.9
8′	22.0	16.3	15.9	16.0	16.4	16.5
9′	17.5	13.2	18.2	18.3	13.4	13.3
10′	169.8	172.6	172.6	172.8	172.8	172.8
MeO	52.2			51.6	51.6	

Brasilianoid B (**2**) ((5a*S*, 5b*S*, 7*R*, 8a*R*, 9*S*, 11a*R*, 11b*S*, 13a*S*)-8a-hydroxy-1, 1, 5a, 7, 9, 11b-hexamethyl-14-methylene-5a, 5b, 6, 7, 8a, 9, 11b, 12, 13, 13a-decahydro-1H, 11H-7,11a-methanofuro[3″,4″:3′,4′]cyclohepta[1′,2′:3,4]benzo[1,2-c]oxepine-3,8,11-trione). Colorless monoclinic crystals (MeOH-Acetone-H_2_O, 5:5:1); m.p. 170–172°C; ${\left[\alpha \right]}_{\text{D}}^{20}$ −40 (*c* 0.5, MeOH); UV (MeOH) λ_max_ (log ε) 206 nm; IR v_max_ (KBr) 3,420, 2,931, 2,966, 1,754, 1,693, 1,604, 1,384, and 1,043 cm^−1^. ^1^H and ^13^C NMR data, see Tables 1, 2; HRESIMS *m/z* 473.2170 [M + HCOO]^−^ (cald for C_26_H_33_O_8_, 473.2175).

Brasilianoid C (**3**) ((5a*S*, 5b*S*, 7*R*, 8a*R*, 9*R*, 11a*R*, 11b*S*, 13a*S*)-8a-hydroxy-1, 1, 5a, 7, 9,11b -hexamethyl-14-methylene-5a,5b,6,7,8a,9,11b,12,13,13a-decahydro-1H,11H-7, 11a-methanofuro[3″,4″:3′,4′]cyclohepta[1′,2′:3,4]benzo[1,2-c]oxepine-3,8,11-trione). Colorless monoclinic crystals (MeOH-H_2_O, 8:1); m.p. 171–173°C; ${\left[\alpha \right]}_{\text{D}}^{20}$ −40 (*c* 0.5, MeOH); UV (MeOH) λ_max_ (log ε) 208 nm; IR v_max_ (KBr) 3,420, 2,930, 2,876, 1,760, 1,694, 1,604, 1,385, and 1,027 cm^−1^. ^1^H and ^13^C NMR data, see Tables 1, 2; HRESIMS *m/z* 473.2181 [M + HCOO]^−^ (cald for C_26_H_33_O_8_, 473.2175).

Brasilianoid D (**4**) (methyl 3-((3*R*, 3a*R*, 5*R*, 6a*S*, 7*S*, 8*S*, 10a*S*, 10b*R*)-3a-hydroxy-8- (2-hydroxypropan-2-yl)-3,5,7,10a-tetramethyl-11-methylene-1,4-dioxodecahydro-1H,3H-5,10b-methanobenzo[3,4]cyclohepta[1,2-c]furan-7-yl)propanoate). Colorless monoclinic crystals; (MeOH-CHCl_3_, 5:1); m.p. 255–258°C; ${\left[\alpha \right]}_{\text{D}}^{20}$ −60 (*c* 0.5, MeOH); UV (MeOH) λ_max_ (log ε) 202 nm; IR v_max_ (KBr) 3,420, 2,965, 2,856, 1,750, 1,661, 1,634, 1,384, and 1,072 cm^−1^. ^1^H and ^13^C NMR data, see Tables 1, 2; HRESIMS *m/z* 507.2590 [M + HCOO]^−^ (calcd for C_27_H_39_O_9_, 507.2594).

Brasilianoid E (**5**) (methyl 3-((3*S*, 3a*R*, 5*R*, 6a*S*, 7*S*, 8*S*, 10a*S*, 10b*R*)-3a-hydroxy-8- (2-hydroxypropan-2-yl)-3,5,7,10a-tetramethyl-11-methylene-1,4-dioxodecahydro-1H,3H-5,10b-methanobenzo[3,4]cyclohepta[1,2-c]furan-7-yl)propanoate). Colorless orthorhombic crystals (MeOH-CHCl_3_-H_2_O, 5:3:1); m.p. 225–228°C; ${\left[\alpha \right]}_{\text{D}}^{20}$ −60 (*c* 0.5, MeOH); UV (MeOH) λ_max_ (log ε) 202 nm; IR v_max_ (KBr) 3,445, 2,966, 2,939, 1,750, 1,662, 1,607, 1,385, and 1,065 cm^−1^. ^1^H and ^13^C NMR data, see Tables 1, 2; HRESIMS *m/z* 507.2600 [M + HCOO]^−^ (cald for C_27_H_39_O_9_, 507.2594).

Brasilianoid F (**6**) ((1*R*, 4*S*, 5*R*, 5a*S*, 5b*R*, 7*R*, 8a*R*, 9*R*, 11a*R*, 11b*S*, 13a*S*)-5, 8a-dihydroxy-1,5a,7,9,11b-pentamethyl-14-methylenedodecahydro-1H,11H-1,4:7,11a-dimethanofuro[3″,4″:3′,4′]cyclohepta[1′,2′:3,4]benzo[1,2-c]oxepine-3,8,11-trione). White amorphous powder; ${\left[\alpha \right]}_{\text{D}}^{20}$ −48 (*c* 0.5, MeOH); UV (MeOH) λ_max_ (log ε) 202 nm; IR v_max_ (KBr) 3,419, 2,986, 2,949, 1,754, 1,720, 1,603, 1,383, and 1,088 cm^−1^. ^1^H and ^13^C NMR data, see Tables 1, 2; HRESIMS *m/z* 443.2077 [M – H]^−^ (cald for C_25_H_31_O_7_, 443.2070).

### ECD calculation

Conformational searches were carried out by random searching in the Sybyl-X 2.0 using the MMFF94S force field with an energy cutoff of 5.0 kcal/mol. Due to the confirmed NOESY correlations and relatively rigid skeleton, the results showed the lowest energy conformers for (1*R*, 2*S*, 4*R*, 5*S*, 8*S*, 9*R*, 10*S*, 1′*R*, 3′*R*, 5′*S*)-**1**′, 1*R*, 2*S*, 4*R*, 5*S*, 8*S*, 9*R*, 10*S*, 1′*R*, 3′*R*, 5′*R*, 6′*R*-**6**′ and for 5*S*, 8*S*, 9*S*, 10*S*, 1′*R*, 3′*R*, 5′*R*, 6′*R*-**7**′ within 5.0 kcal/mol. Subsequently, the conformers were re-optimized using DFT at the B3LYP/6-31+G(d) level in gas phase by the GAUSSIAN 09 program. The energies, oscillator strengths, and rotational strengths (velocity) of the first 60 electronic excitations were calculated using the TDDFT methodology at the b3lyp/6-311++g(d, p) level in vacuum. The ECD spectra were simulated by the overlapping Gaussian function (half the bandwidth at 1/e peak height, σ = 0.35 for **1** and 0.25 for **6**). By comparison of the calculated ECD spectra with the experimental curves, the absolute configuration of **1** and **6** were resolved.

### Assay for *in vitro* anti-HBV effects

HepAD38 cells were cultured with DMEM in 48-well plate at 1 × 10^5^ cells/well for 24 h, then treated with 10 μM of each compound for 72 h. After 4 days, the cells were washed with precooled PBS for 2 times. HBV progeny DNA of HepAD38 cells were extracted using QIAamp DNA Blood Mini kit (Biomiga) according to the manufacturer's instruction. Total DNA was reversely transcribed using PrimeScript RT reagent Kit (Takara, Dalian, China). The primers were designed and synthesized by Takara, and the sequences of the primers are indicated in Table S9. PCR amplification was performed on an StepOne Plus real time PCR system (Applied Biosystems, Foster City, CA) using the SYBR Green Master Mix (Applied Biosystems, Foster City, CA). All experiments were performed in triplicate, and the relative levels of assayed HBV DNA were calculated with the delta–delta CT method using lamivudine as a positive control, and normalized to non-treated control.

### Assay for protective effects on skin barrier functions in *in vitro*

HaCaT cells were cultured with DMEM in 6-well plate at 1 × 10^5^ cells/well for 24 h, then treated with 20, 10, 5 μM of compounds **1**–**7** for 72 h. Total RNA of HaCaT cells were extracted using RNA Miniprep kit (Biomiga) according to the manufacturer's instruction. Then total RNA was reverse transcribed using PrimeScript RT reagent Kit (Takara, Dalian, China). The primers were designed and synthesized by Takara, the sequences of the primers are indicated in Table S10. PCR amplification was performed on an StepOne Plus real time PCR system (Applied Biosystems, Foster City, CA) using the SYBR Green Master Mix (Applied Biosystems, Foster City, CA). All experiments were performed in triplicate, and the relative levels of assayed mRNAs were calculated with the delta–delta CT method using ACTIN expressions as endogenous control, and normalized to non-treated control.

HaCaT cells were seeded at 5 × 10^5^ cells per well in 6-well plates and incubated under starvation conditions for 24 h using serum-free MEM. HaCaT cells were exposed to UVB irradiation (UVB lamp: SUV-1000, Sigma-China) at a dose of 30 mJ/cm^2^. After UVB irradiation, DMEM medium containing **1** (20 μM) or epigallocatechin gallate (20 μM) was added and the cells were incubated for 24 h, and then the MTT assay was performed.

### Assay for the inhibition of nitric oxide production in RAW264.7 macrophages

All compounds were assayed by measuring the inhibitory effects on NO production induced by LPS in mouse macrophage RAW 264.7 cells. The RAW 264.7 cells were seeded in each well of a 96-well plate at a concentration of 5 × 10^5^ cells/ml in DMEM medium containing 10% fetal bovine serum, 2 mmol/l glutamine, 100 U/ml penicillin, and 100 μg/ml streptomycin. After incubation at 37°C in a humidified atmosphere of 5% CO_2_ air for 12 h, cells were treated with LPS (1 μg/ml) and compounds (50 μg/ml for each compound, and compound was dissolved in DMSO to dilute until the final DMSO concentration <0.1%, v/v), then incubation for 24 h at 37°C. NO production was determined by measuring the quantity of nitrite in the culture medium by the Griess reagent. Aminoguanidine was selected as a positive control. A 50 μl of the supernatant of culture medium was mixed with 100 μl of the Griess reagent. Using nitrite to generate a standard curve, nitrite production was measured by a microplate reader at 540 nm. Cell viability was examined by the MTT method (Wu et al., 2016). The compounds with inhibitory rate more than 50% at a dose of 50 μg/ml were further detected in the gradient concentrations to calculate IC_50_ values.

### Statistical analysis

All quantitative values are given as means ± SEMs. Significant differences among the experimental groups were assessed by one-way ANOVA. Statistical significances were considered at the ^*^P < 0.05 and ^**^P < 0.01 levels.

### Crystal structure analysis

X-ray crystal data of **2**–**5** and **8** were acquired on an Eos CCD with a graphite monochromated Cu Kα radiation (λ = 1.5418 Å) (Supporting Information). These structures was solved with ShelXT-97 using direct methods and refined with ShelXL using full matrix least-squares on F_2_. Crystallographic data for **2**–**5** and **8** have been deposited at the Cambridge Crystallographic Data Center with the numbers of CCDC 1572280 for **2**, CCDC 1572282 for **3**, CCDC 1576933 for **4**, CCDC 1576930 for **5** and CCDC 1583230 for **8**. Crystallographic data are deposited in the Cambridge Crystallographic Data Centre. and are available free of charge via the Internet at www.ccdc.cam.ac.uk/products/csd/request.

## Results

### Identification of new compounds

The molecular formula of brasilianoid A (**1**) was determined as C_26_H_34_O_8_ according to the HRESIMS ion at *m/z* 473.2176 [M – H]^−^ and the NMR data, requiring ten degrees of unsaturation. The ^1^H NMR data presented five tertiary methyl singlet at δ_H_ 0.76 (s), 1.17 (s), 1.29 (s), 1.33 (s), and 1.34 (s), and a methoxy group at δ_H_ 3.57 (s), one oxygenated methine at δ_H_ 3.47 (t, *J* = 4.2 Hz), as well as the terminal olefinic methylene protons at δ_H_ 4.67 (s) and 5.21 (s) (Table 1). The ^13^C NMR and DEPT spectra exhibited a total of 26 carbon resonances (Table 2), which were classified into six methyl, five methylene (one olefinic carbon), four methine, and eleven quaternary carbons involving two ketones and two ester carbonyl carbons. Analyses of the 1D and 2D NMR data revealed that **1** is a preaustinoid-related meroterpenoid, while the partial structure regarding rings C/D/E of **1** was corresponded to rings B/C/D of preaustinoid A2 (**8**) (dos Santos and Rodrigues-Fo, 2003) with the exception of ring A. The COSY correlation coupled H-2 (δ_H_ 2.50, dd, *J* = 4.2, 4.9 Hz) to H-1 (δ_H_ 3.47) and H_2_-15 (δ_H_ 1.81, 2.24), along with the extension of COSY coupling between H-1 and a D_2_O exchangeable proton at δ_H_ 5.16 (d, *J* = 4.2 Hz), ascertained the location of a hydroxy group at C-1 (δ_C_ 70.0). A methylene bridge across C-2 (δ_C_ 48.0) and C-4 (δ_C_ 86.8) was evident from the HMBC correlations from H_3_-14 (δ_H_ 1.29, s) to C-4, C-5 (δ_C_ 48.3), and C-15 (δ_C_ 40.7), and from H_2_-15 to C-1, C-2, C-4, and C-5. Additional HMBC correlation from the carbonyl carbon C-3 (δ_C_ 177.5) to H-1, H-2, and H_2_-15 in association with the degrees of molecular unsaturation confirmed the formation of a γ-lactone for ring A.

**Figure 3:**
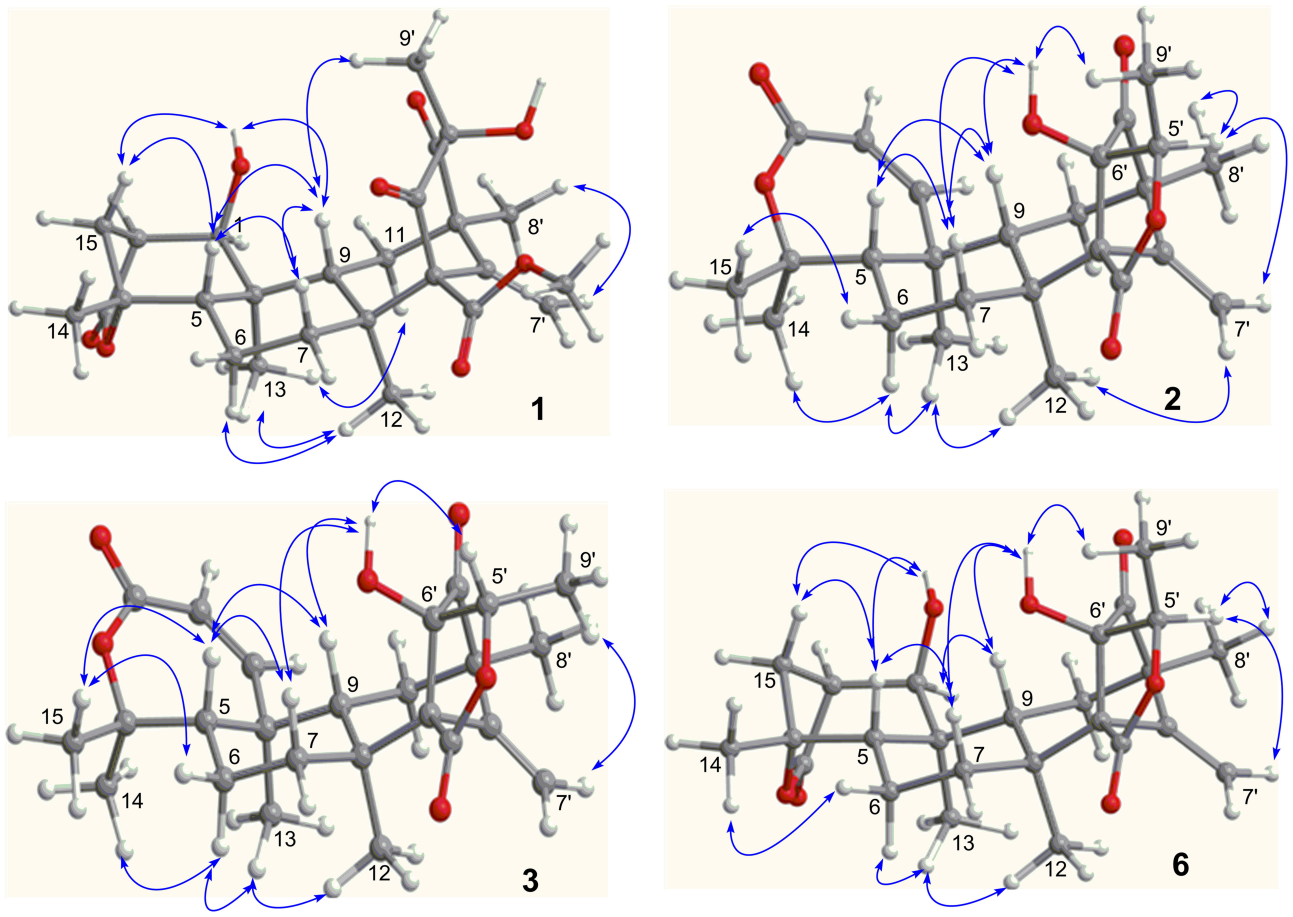
Key NOE correlations of **1–3** and **6**. Arrow line means the NOE correlation from one proton to the other proton.

**Figure 4:**
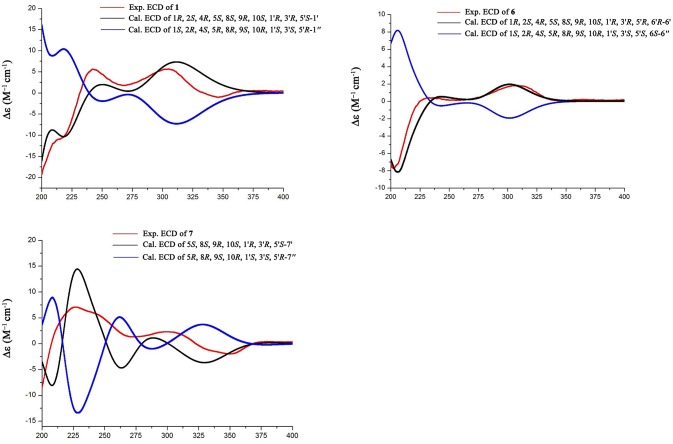
Experimental and calculated ECD of **1, 6**, and **7**.

The relative configuration of **1** was established by the NOE data. As shown in Figure 3, the NOE correlations from H-15a (δ_H_ 2.24) to OH-1 and H-5, and from H-9 (δ_H_ 1.50) to OH-1, H-5, and H_3_-9′, in association with the correlation between H_3_-12 (δ_H_ 1.17) and H_3_-13 (δ_H_ 0.76), clarified *trans* fusion between B/C and C/D rings. When H-1 was arbitrarily assigned to α-orientation, the methylene bridge H_2_-15 was ascertained to be in β-orientation. The relative configuration in rings C to E was the same as that of preaustinoid A2, based on the similar NOE relationship. The spatial closure between H_3_-9′ and H-9 was recognized by their NOE interaction, while the NOE correlation between H_3_-12 and H_2_-7′ indicated that the *exo*-vinyl group across C-1′ and C-3′ was α-oriented. In biogenetic consideration, compound **1** was assumed to be derived from preaustinoid A2 by the formation of C-2/C-15 bond. Thus, both compounds were suggested to share the same absolute configuration in rings C-E. Since the absolute configuration of preaustinoid A2 was determined by the single crystal X-ray diffraction analysis using Cu K_α_ radiation in present work, the stereogenic centers in rings C-E of **1** were supposed to be 8*S*, 9*S*, 10*R*, 3′*R*, 5′*S*, and 7′*R* configurations (Supporting Information). Thus, the absolute configuration in rings A and B was in agreement with 1*R*, 2*R*, 4*S*, and 5*S*, based on the NOE relationships. This assignment was further supported by the ECD data (Stephens and Harada, 2010), of which the Cotton effects of experimental and calculated ECD for the model molecule of **1** with 1*R*, 2*R*, 4*S*, 5*S*, 8*S*, 9*S*, 10*R*, 3′*R*, 5′*S*, and 7′*R* were comparable (Figure 4).

Brasilianoid B (**2**) was isolated as colorless monoclinic crystals. Its molecular formula was determined as C_25_H_32_O_6_ based on the HRESIMS (*m/z* 473.2170 [M + HCOO]^−^) and NMR data, containing ten degrees of unsaturation. The ^1^H and ^13^C NMR data of **2** (Tables 1, 2) regarding rings A-C resembled those of preaustinoid A2, while the distinction was attributed to rings D and E. An exocyclic olefinic group at C-2′ (δ_C_ 147.6) in ring C was recognized by the HMBC correlations from the olefinic methylene protons H_2_-7′ (δ_H_ 4.86, 5.09) to C-1′ (δ_C_ 66.5) and C-3′ (δ_C_ 55.3). Additional HMBC correlations from H_3_-8′ (δ_H_ 1.20, s) to C-2′, C-3′, and a ketone carbon C-4′ (δ_C_ 213.4), and from OH-6′ (δ_H_ 6.95, s) to C-4′, C-1′, and C-6′ (δ_C_ 90.8) established a cyclopentanone unit for ring D. Moreover, the HMBC correlations from H-5′ (δ_H_ 4.30, q, *J* = 6.4 Hz) to C-1′, C-4′, C-6′, and a carbonyl carbon C-10′ (δ_C_ 172.6), in addition to the COSY coupling between H-5′ and H_3_-9′ (δ_H_ 1.14, d, *J* = 6.4 Hz), deduced a γ-lactone unit for ring E, while a hydroxy group and a methyl group were located at C-6′ and C-5′, respectively.

**Figure 5:**
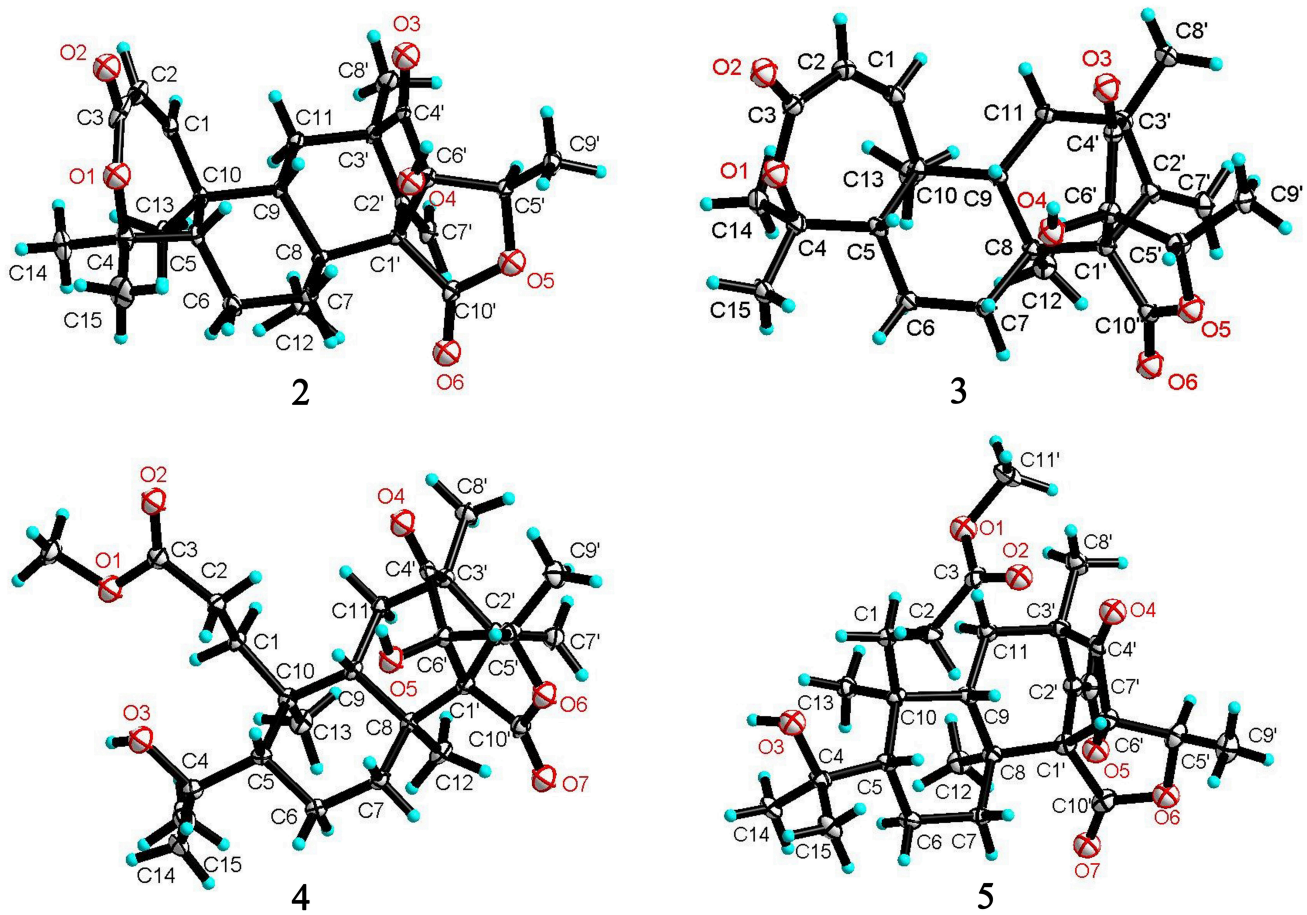
X-ray crystallographic structures of **2-5**.

The relative configuration of **2** in rings A-C was the same as that of preaustinoid A2, based on the similar NOE relationships. The NOE correlations from OH-6′ to H-7a (δ_H_ 2.23) and H-9 (δ_H_ 1.99) demonstrated the spatial closure of these protons, suggesting OH-6′ to be in β-orientation. Additional NOE correlations between OH-6′/H_3_-9′, H-5′/H_2_-7′, H-5′/H_3_-8′, and H_2_-7′/H_3_-12 (Figure 2) clarified the relative configuration in rings D and E. Based on the Flack parameters [−0.04(13)] measured by the Cu-Kα X-ray diffraction of the single crystal, the absolute configurations of **2** were finally determined as 5*S*, 8*S*, 9*S*, 10*S*, 1′*R*, 3′*R*, 5′*R*, and 6′*R* (Figure 5).

The planar structure of brasilianoid C (**3**) was the same as that of **2**, based on the 1D and 2D NMR data in association with the same molecular formula as determined by the HRESIMS data. The distinction was ascribed to the carbons C-5′ (δ_C_ 83.5) and C-9′ (δ_C_ 18.2), which showed more deshielded resonances than the corresponding carbons of **2** (Table 2). The NOE data raised from rings A-D were in accordance with those of **2**, indicating that this partial structure in **3** was the same as that of **2**. However, the NOE correlations between OH-6′ (δ_H_ 7.09, s) and H-5′ (δ_H_ 4.46, q) and from H_3_-9′ (δ_H_ 1.03, d) to H_2_-7′ (δ_H_ 4.93, 5.14) and H_3_-8′ (δ_H_ 1.16, s) suggested **3** to be an C-5′ epimer of **2**. This assignment was confirmed by the Cu-Kα X-ray diffraction data, that clarified 5′*S* configuration in **3** (Figure 5).

Brasilianoid D (**4**) has a molecular formula of C_26_H_38_O_7_ as determined by the HRESIMS and NMR data, bearing nine degrees of unsaturation. Analyses of the 2D NMR (COSY, HMQC, HMBC) data established the partial structure in rings B-E of **4** to be the same as that of **3**. However, the NMR data of **4** presented a methoxy group (δ_H_ 3.57, δ_C_ 51.6), and the methoxy protons correlated to a carbonyl carbon C-3 (δ_C_ 174.3) in the HMBC spectrum to form a methyl ester. The HMBC correlations from H_2_-1 (δ_H_ 1.32, 2.43) and H_2_-2 (δ_H_ 1.72, 2.42) to C-3 and C-10 (δ_C_ 41.1) demonstrated a methyl propionate unit to be positioned at C-10. In addition, a D_2_O exchangeable proton at δ_H_ 4.09 (s) showed the HMBC correlation with C-4 (δ_C_ 74.3), C-5 (δ_C_ 51.1), and two methyl carbons C-14 (δ_C_ 28.0) and C-15 (δ_C_ 33.9), and therefore pointed the substitution of a 2-hydroxyisopropane unit at C-5. Thus, compound **4** was likely derived from preaustinoid A1 (Geris dos Santos and Rodrigues-Fo, 2002) by acyclized ring A and the cyclic rearrangement in ring D. The NOE relationship of OH-6′ (δ_H_ 7.04, s) with H-7a (δ_H_ 2.23), H-9 (δ_H_ 2.07), and H-5′ (δ_H_ 4.42), and from H_3_-9′ (δ_H_ 1.03, d) to H_2_-7′ (δ_H_ 4.89, 5.08), and H_3_-8′ (δ_H_ 1.12, s), deduced the same relative configuration of both **4** and **3**. The absolute configuration of **4** was in agreement with that of **3** according to the Flack parameters of the Cu-Kα X-ray diffraction for the single crystals (Figure 5).

The planar structure of brasilianoid E (**5**) was the same as that of **4**, as determined by the HRESIMS data (*m/z* 507.2600 [M + HCOO]^−^, cald for C_27_H_39_O_9_, 507.2594) and the comparable 1D and 2D NMR data. The distinction was attributed to the NMR resonances at C-5′ (δ_C_ 76.1) and C-9′ (δ_C_ 13.4) which shifted 7 and 5 ppm, respectively, to upfield in comparison with those of **4** (Tables 1, 2). As the case of **2**, the NOE correlation between OH-6′ (δ_H_ 6.76, s) and H-5′ (δ_H_ 4.29) and between H_3_-9′ (δ_H_ 1.15) and H_2_-7′ (δ_H_ 4.82, 5.04) clarified **5** to be an C-5′ epimer of **4**. The Cu-Kα X-ray crystallographic diffraction of the single crystal using Flack parameters (-0.006(9)) further confirmed the absolute configurations of **5** to be 5*S*, 8*S*, 9*S*, 10*S*, 3′*R*, 5′*R*, 6′*R*, and 7′*R* (Figure 5).

Brasilianoid F (**6**) has a molecular formula of C_25_H_32_O_6_ as determined by the HRESIMS (*m/z* 443.2077 [M-H]^−^, cald for C_25_H_31_O_7_, 443.2070) and NMR data, requiring ten degrees of unsaturation. Analyses of 1D and 2D NMR data indicated that rings A to C are in accordance with those of **1**, as evident from the proton spin systems from OH-1 (δ_H_ 5.16, d, *J* = 4.5 Hz) to H_2_-15 (δ_H_ 1.87, 2.54) and from H-5 (δ_H_ 1.67) to H_2_-7 (δ_H_ 1.63, 2.16) in the COSY spectrum, in addition to the key HMBC correlations observed from H_2_-15 to C-1, C-2, C-3, C-4 and C-5; H_3_-14 to C-4, C-5 and C-15; H_3_-12 to C-7, C-8, C-9, and C-1′; H_3_-13 to C-1, C-5, C-9, and C-10, and OH-1 to C-1, C-2 and C-10. Moreover, the HMBC and COSY correlations assigned the partial structure of rings D to E to be the same as that of **2**. The similar NOE data, such as the key NOE correlations from OH-1 to H-15b (δ_H_ 2.54), H-5 and H-9 and between H_3_-12 and H_3_-13 as observed in the NOESY spectrum of **1** suggested the same relative configuration in rings A-C of both **6** and **1**. The NOE correlations from H-5 to H-15b and H-7b (δ_H_ 2.16), H-7b to H-9 and OH-6′, and between OH-6′ and H_3_-9′ (Figure 2) clarified these protons to be cofacial, indicating the same relative configuration in rings C-E of both **6** and **2**. Additional NOE correlations from H-5′ to H_2_-7′ and H_3_-8′ supported the *exo*-vinyl bridge C-2′ to be in the same orientation as H-5′. In order to assign the absolute configuration, the ECD data of **6** and its enantiomer were calculated at the B31YP/6-311++G(2d, 2p) level in the gas phase using the B3LYP/6-31G(d) optimized geometries after conformational searches via the MMFF94S force field (Ding et al., 2007). Comparison of the experimental ECD data with those calculated for the enantiomers indicated that **6** (Figure 4) led to the identification of 1*R*, 2*S*, 4*R*, 5*S*, 8*S*, 9*S*, 10*R*, 3′*R*, 5′*R*, 6′*R*, and 7′*R* configurations.

Analyses of 1D and 2D NMR data established the planar structure of **7** to be identical to preaustinoid D (Duan et al., 2016), while the similar NOE correlation and ECD data clarified both compounds to be identical. However, comparison of the experimental ECD data with that calculated for a model molecule of (5*S*, 8*S*, 9*S*, 10*S*, 3′*R*, 5′*S*, 6′*R*, 7′*R*)**-7** (Figure 4) suggested that the structure of preaustinoid D should be revised to its enantiomer. Compound **8** was identical to preaustinoid A2 based on the comparison of its spectroscopic data with these reported in the literature (dos Santos and Rodrigues-Fo, 2003), while the absolute configuration was determined by the Flack parameters of the X-ray diffraction of the single crystals.

The NMR data of the epimers **2**/**3** and **4**/**5** revealed that the chemical shifts for C-5′ and C-9′ in **2** and **5** with 5′ *R* exhibited the resonances at δ_C_ 76 (C-5′) and δ_C_ 13 (C-9′), whereas those for 5′*S* isomers (**3**, **4**) showed more deshielded resonances at δ_C_ 83 (C-5′) and δ_C_ 18 (C-9′). The similar cases were also found in other analogues reported in literature. These NMR distinction provided the evidence to identify the configuration of C-5′ in austin-related meroterpenoids (Duan et al., 2016; Park et al., 2018). For instance, the chemical shifts of C-5′ (δ_C_ 76.2) and C-9′ (δ_C_ 13.3) in **6** and C-5′ (δ_C_ 78.5) and C-9′ (δ_C_ 12.2) in **7** implied the same orientation of OH-6′ and H_3_-9′.

### Biological activity

**Figure 6:**
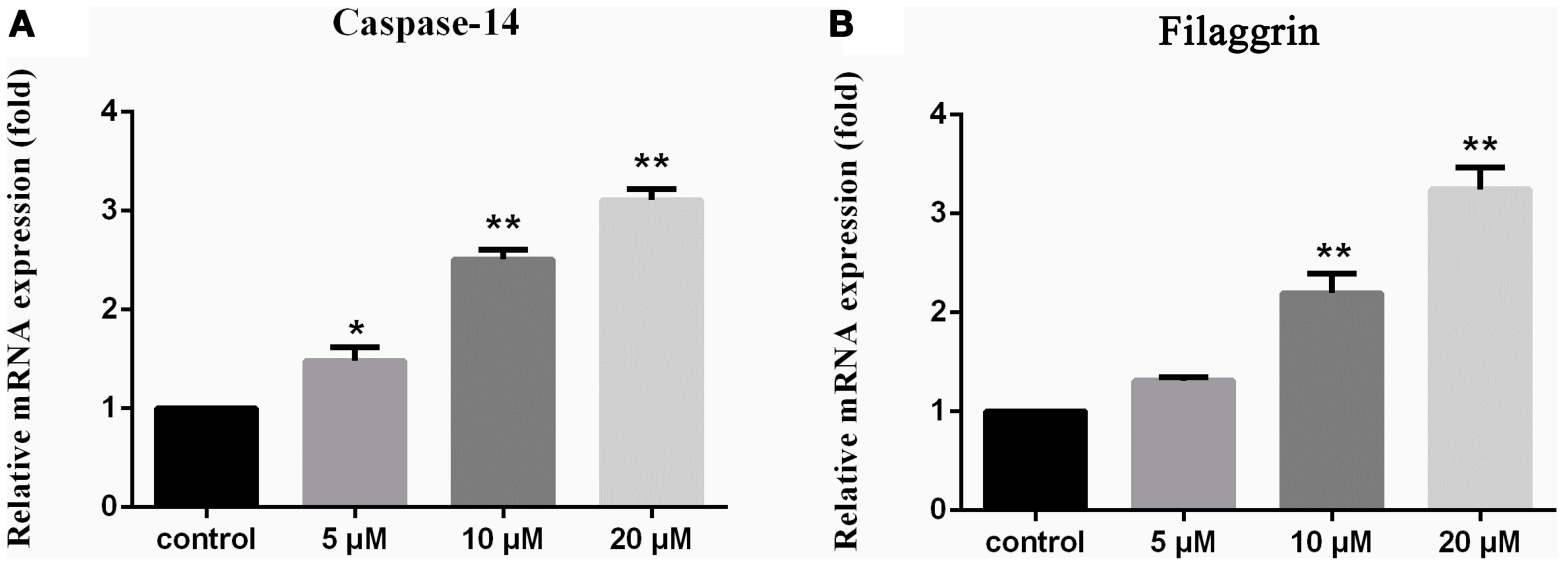
Upregulation of caspase 14 and filaggrin in HaCaT cells induced by **1** 1% DMSO for the dissolution of compounds is used as the control. The vertical axis represents the expression of caspase-14 **(A)** and filaggrin **(B)** in HaCaT cells, and the horizontal axis means the doses of compound **1**. Statistical significances were considered at the *P < 0.05 and **P < 0.01 levels.

Compounds **1–8** showed no cytotoxic activities toward tumor cell lines A549, U937 and SMMC7721 with IC_50_ > 10 μM. However, compound **1** significantly stimulated the expression of filaggrin and caspase-14 in HaCaT cells with a dose dependent manner (Figure 6). Filaggrin is a key natural moisturizing factor that maintains the ability to regulate the skin moisture barrier (Rawlings and Harding, 2004), while the mutation of the filaggrin gene resulted in skin losing water to allow the entrance of bacteria, leading to allergies, irritation and infection (Nomura et al., 2007). Caspase-14 deficiency was associated with the accumulation of incompletely degraded filaggrin fragments within the stratum corneum, decreased stratum corneum hydration, increased transepidermal water loss, and sensitivity to UVB photodamage (Eckhart and Tschachler, 2011). To test the skin protective activity against UVB irradiation, the cytotoxicity of **1** against HaCaT cells was measured by the MTT assay. The cell viability was decreased to 70% compared to the normal group after exposure to UVB 30 mJ/cm^2^. Treatment of the damaged cells with compound **1** (20 μM) resulted in the cell viability increasing to 77%, while the positive control epigallocatechin gallate increased the cell viability to 75% at the same dose. This finding indicated that compound **1** is able reduce the UVB-induced cell damage.

Compounds **2** and **3** showed moderate inhibition toward NO production in LPS-induced RAW 264.7 macrophages (Table 3). In addition, compounds **3–5** (10 μM) inhibited the DNA expression of the HBV virus in HepG2.2.15 cells with the inhibitory rates of 25, 15, and 10%, respectively, whereas lamivudine as a positive control exerted the inhibitory rate of 75% in the same dose (Supporting Information, Figure S8).

**Table 3:** Inhibitory effects against LPS induced NO production in RAW264.7 macrophages.

**Compounds**	**NO inhibition % (50 μM)**	**IC_50_ (μM)**	**CC_50_ (μM)**
		**NO**	**Cytotoxicity**
**1**	14		>100
**2**	58	37.69 ± 5.25	>100
**3**	65	33.76 ± 3.13	>100
**4**	32		>100
**5**	36		>100
**6**	22		>100
**7**	28		>100
**8**	14		>100
Aminoguanidine		7.62 ± 0.08	—

*IC_50_, 50% inhibitory concentration; CC_50_, 50% cytotoxic concentration*.

## Discussion

In present work, we reported *P. brasilianum* WZXY-m122-9 to be obtained from a marine sponge for the first time. It provides a new source to produce austin-related meroterpenenes with the uniquely modified structures, implying the same fungal species from different origins activating distinct biosynthetic pathway or enzyme function. Since austin-related meroterpenenes have been reported to possess insecticide and antibiotic activities, the sponge-associated fungus may generate the relevant metabolites to play chemoecological role in the association with host for the defense of micro- or macro-organism invasion.

**Scheme 1:**
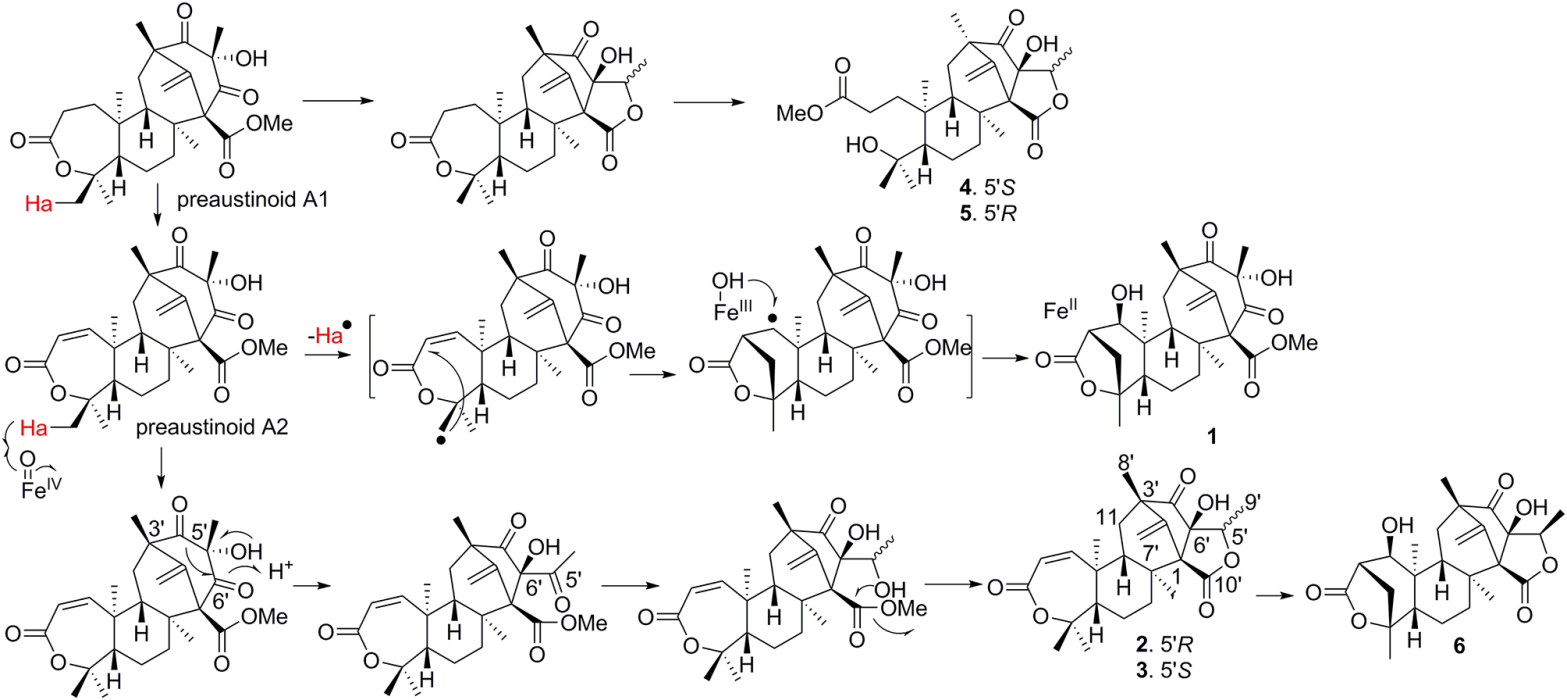
Proposal of putative biosynthetic pathways for the compounds.

The early-stage in the biosynthesis of **1**–**6** was considered to follow the similar pathway of austin (Scott et al., 1986; Ahmed et al., 1989). The PKS synthesizes DMOA, which followed by farnesylation, methylesterification, epoxidation, terpene cyclization, Baeyer-Villiger oxidation to yield preaustinoid A1 (a product coexisted with **1**–**6** in the fungal strain) (Itoh et al., 2012). The latter compound can be converted to preaustinoid A2 by dehydrogenation. Compounds **1** and **6** presented as unique scaffold with γ-lactone unit in ring A. Both compounds were biogenetically presumed to be converted from preaustinoid A2 by the radical-based pathway, similar to the case for the biotransformation of preandiloid C to andiconin (Matsuda et al., 2014). During the formation of γ-lactone, Me-15 in preaustinoid A2 was catalyzed by a Fe(II)/α-KG-dependent dioxygenase to yield a radical intermediate, which subsequently formed a C-15/C-2 bond with the electron-rich olefin at C-2 to generate an intermediate with a free radical at C-1. The latter carbon was hydroxylated to yield compound **1**. The formation of γ-lactone in **6** was suggested to follow the similar manner as for **1**. Conversion of preaustinoid A2 to **2** and **3** was presumably mediated by the enzymes as the case of the biotransformation from preaustinoid A3 to austinol. Coexistence of C-5′ epimers (**2**, **3**) with equal amount suggested that the stereogenic selection for the reduction of ketone at C-5′ to hydroxy group is unspecific. Compounds **4** and **5** are regarded to be derived from preaustinoid A1, which followed the similar rearrangement as that for the conversion of preaustinoid A2 to **2** and **3**. We isolated a dioxygenase from the fungus, which was identical to AusE (Matsuda et al., 2013; Valiante et al., 2017). The dioxygenase can convert preaustinoid A2 to preaustinoid A3 in vitro experiment. However, The transformation of preaustinoid A2 to **1** or **6** was unsuccessful. This data suggested that the other Fe(II)/α-KG-dependent dioxygenase is required for the generation of the methylene bridge in **1** and **6**. The methyl ester of **4** and **5** is derived by the hydrolysis of the lactone in ring-A to yield a carboxylic acid at the side chain, and then methyl esterification (Scheme 1). Although we found **4** and **5** in the HPLC chromatographic profile of EtOAc extract, whether both compounds to be derived by enzymatic step are uncertain.

## Conclusion

In conclusion, compounds **1** and **6** possess unprecedented skeleton featuring the formation of C-2/C-15/C-4 bridge in ring A, while **2** and **3** with 7/6/6/5/5 pentacyclic skeletons of meroterpenoids are found from nature for the first time. The bioassay results indicated that the structural variation directly induced the bioactive spectrum. Compound **1** is the first example of natural products used to promote filaggrin and caspase-14 expression for the protection of the UVB-induced cell damage, suggesting that it is a promising lead for the treatment of dermatological diseases.

## Author contributions

JZ responses for the isolation of compounds. BY performed the biogenetic approach for synthetic gene screening. DL took the antiviral bioassay. SG assayed for the regulation of filaggrin and caspase-14. PP helped to revise the manuscript. WL elucidated the structures and edited the manuscript.

### Conflict of interest statement

The authors declare that the research was conducted in the absence of any commercial or financial relationships that could be construed as a potential conflict of interest.
