# Supplementary material for: Brasilianoids A–F, New Meroterpenoids From the Sponge-Associated Fungus Penicillium brasilianum
Source: Front Chem. 2018 Jul 27;6:314. doi: 10.3389/fchem.2018.00314 (PMC6072878; doi:10.3389/fchem.2018.00314)
Supplement: Supplementary file 1 [file Data_Sheet_1.PDF]

Brasilianoids A–F, New Meroterpenoids from the Sponge-associated Fungus *Penicillium brasilianum*

Supporting Information

Experimental Section

1. General

Optical rotations were measured with an Autopol III automatic polarimeter. IR spectra were measured with a Thermo Nicolet Nexus 470 FT-IR spectrometer. 1D and 2D NMR spectra were recorded on a Bruker Avance-400FT NMR spectrometer using TMS as internal standard. HRESIMS data were acquired on a Bruker APEX IV 70 eV FT-MS spectrometer. ESIMS data was obtained on a Finnigan MAT 95 mass spectrometer. The column chromatographic substrates were as follows: silica gel (200−300 mesh) and HF254 silica gel for TLC (Qingdao Marine Chemistry Co. Ltd.), Sephadex LH-20 (18-110 μm; Pharmacia Co., Ltd.); ODS (50 μm; YMC, Milford, MA). Semipreparative HPLC was performed with an Alltech instrument (426-HPLC pump) equipped with an UV detector at 210 nm and using a Prevail-C_{18} column (Semipreparative, 5 μm). The X-ray data were measured with a Bruker SMART APEX-II DUO instrument.

2. Fungal strain identification

The fungus *Penicillium brasilianum* WZXY-m122-9 was isolated from a marine sponge, which was collected in July 2016 from Weizhou Island in the South China Sea, and identified by microscopic examination and 18S rDNA ITS sequence's BLAST in GenBank (GenBank accession number HM469396). A voucher specimen (WZXY-m122-9) was deposited at the State Key Laboratory of Natural and Biomimetic Drugs, Peking University, China.

The primers to obtained ITS sequence are as follows:
P1: 5'-AGAAGTCGTAACAAGGTTTC-3'
P4: 5'-TCCTCCGCTTATTGATATGC-3'
The ITS sequence (after stitching and proofreading) of *P. brasilianum*WZXY-M122-9 is as follow:
5'-
GTGAACCTGCGGAAGGATCATTACTGAGTGAGGGCCCTCTGGGTCCAACCT
CCCACCCGTGTTTATTGTACCTTGTTGCTTCGGCGCGCCCGCCTCACGGCCG
CCGGGGGGCATCCGCCCCCGGGCCCGCGCCCGCCGAAGACACCATTGAAC
TCTTGTCTGAAGATTGCAGTCTGAGTAGATTAGCTAAATCAGTTAAAACTTT
CAACAACGGATCTCTTGGTTCCGGCATCGATGAAGAACGCAGCGAAATGC
GATAAGTAATGTGAATTGCAGAATTCAGTGAATCATCGAGTCTTTGAACGC
ACATTGCGCCCCCTGGTATTCCGGGGGGCATGCCTGTCCGAGCGTCATTGC

TGCCCTCAAGCACGGCTTGTGTGTTGGGCTTCGCCCCCCGTTCATCGGGGG
GCGGGCCCGAAAGGCAGCGGCGGCACCGCGTCCGGTCCTCGAGCGTATGG
GGCTTTGTCACCCGCTCTGTAGGCCCGGCCGGCGCCCGCCGGCGACACCC
AAATCAATCTATCCAGGTTGACCTCGGATCAGGTAGGGATACCCGCTGAAC
TTAAGCATATCAATAAGACG-3’ (579 bp)

3. Genome Sequencing and Analysis

Genome sequencing of *P. brasilianum* WZXY-M122-9 was performed by Sangon Biotech (Shanghai) Co., Ltd. (Shanghai, China) with an Illumina HiSeq 2000 system. Sequence assembly was performed with SPAdes version 3.5.0 (http://cab.spbu.ru/software/spades/) to yield 1367 contigs covering approximately 34.7 Mb. Gene prediction was then performed with Prokka (https://github.com/tseemann/prokka). Anti-SMASH (antibiotics and Secondary Metabolite Analysis Shell) analysis of genome sequence was performed to detect secondary metabolite gene clusters, and accurate gene cluster alignment was performed manually by comparisons with homologous genes found in the NCBI database.

4. Quantitative RT-PCR for *PM-122-9_1376’* and *PM-122-9_1374*

Two key genes expression level, *PM-122-9_1376’* (prenyltransferase gene) and *PM-122-9_1374* (terpene cyclase gene), were detected by qRT-PCR. We obtained total RNA of PM122-9 in rice culture medium and cDNA was synthesized from 1 μg of total RNA in a total volume of 20 μL using TransScriptIIAll-in-One First-Strand cDNA Synthesis Super Mix (Transgene) for qPCR according to the manufacturer’s instructions. 0.4 μL cDNA, forward primer (10 μM), reverse primer (10 μM) and 10μL 2× TransStart Top Green qPCRSuperMix (Transgene) were used in subsequent RT-PCR reactions with supplement ddH_2O to 20 μL. The specific primers were as follows:

actin-F: 5'-ACCTGCTCTGCGACTACAAC-3'

actin-R: 5'-ACACCGCCCTCATAATAAAG-3'

PM-122-9_1376’-F:5'-CCACCAAAGGGGATTACCA-3'

PM-122-9_1376’-R:5'-GAGCAGAAATGTCGCAGGAA-3'

PM-122-9_1374-F:5'-CGGTAGGATGGTCGGTCAAC-3'

PM-122-9_1374-R: 5'-ACGGCGGAGTGTAGGAAGAA-3'

Optimized PCR conditions were 94 °C for 30 s; 45 cycles of 94 °C for 5 s; 60 °C for 15 s; and 72 °C for 10 s; followed by dissociation stage. Three parallel tests for each reaction and recording their respective Ct values. The *β-actin* gene was treated as an internal reference gene.

Table S1 Proposed functions of the proteins in *P. brasilianum* WZXY-m122-9 and their amino acid identity with those in *P. brasilianum* MG11[1]

Proteins in WZXY-m122-9	Amino acids	Protein homologue	Putative function	Identity%
7564 (MH277559)	1689	PMG11_06808	Isomerase	99
7565 (MH277560)	1662	PMG11_06809	Cytochrome P450 monooxygenase	99
7566 (MH277561)	1449	PMG11_06811	O-acetyltransferase	93
7567 (MH277562)	1551	PMG11_06812	O-acetyltransferase	95
7568 (MH277563)	570	PMG11_06813	Cytochrome P450 monooxygenase	97
7569 (MH277564)	435	PMG11_06814	Isomerase	100
7570 (MH277565)	1884	PMG11_06817	Cytochrome P450 monooxygenase	98
7571 (MH277566)	1197	AusK	Ketoreductase	94
7572 (MH277567)	489	PMG11_06819	Isomerase	99
5653 (MH277568)	840	PMG11_09857	Methyltransferase	99
5654 (MH277569)	7428	PMG11_09856	Polyketide synthase	98
5655 (MH277570)	1941	PMG11_09855	FAD-dependent monooxygenase	99
5656 (MH277571)	1926	PMG11_09854	FAD-dependent monooxygenase	99
5657 (MH277572)	774	PMG11_09853	Short chain dehydrogenase	99
1374 (MH286471)	738	PMG11_09852	Terpene cyclase	99
1375 (MH286472)	2940	PMG 11_09851	Major facilitator superfamily	98
1376 (MH492327)	1431	PMG11_09850	FAD-dependent monooxygenase	95
1376 (MH286473)	951	PMG11_09849	Prenyltransferase	99
1377 (MH286474)	216	PMG11_09848	partial cytochrome P450	63
1378 (MH286475)	906	PMG11_09847	AusE-like dioxygenase	99
3927(MH373561)	444	PMG11_09847	Isomerase	97

[1]PMG11: proteins in *P. brasilianum* MG11.

Table S2 Ct value of *β-actin*, *PM-122-9_1376* and *PM-122-9_1374*

Gene		NTC	Samples
β-actin	1	36.13	28.67
	2	35.02	28.77
	3	37.50	28.79
	Average	36.22	28.74
PM-122-9_1376	1	35.08	20.99
	2	33.71	20.93
	3	34.43	21.00
	Average	34.41	20.97
PM-122-9_1374	1	34.22	21.22
	2	35.77	21.13
	3	34.25	21.22
	Average	34.75	21.19

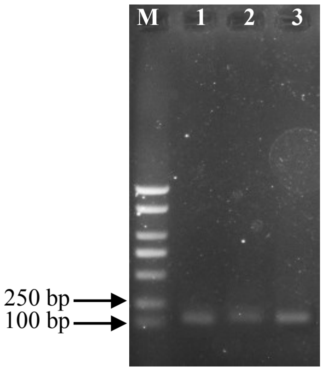

Figure S1 PCR result of PM-122-9 cDNA. 1~3 were *β-actin*, *PM-122-9_1376'* and *PM-122-9_1374* respectively.

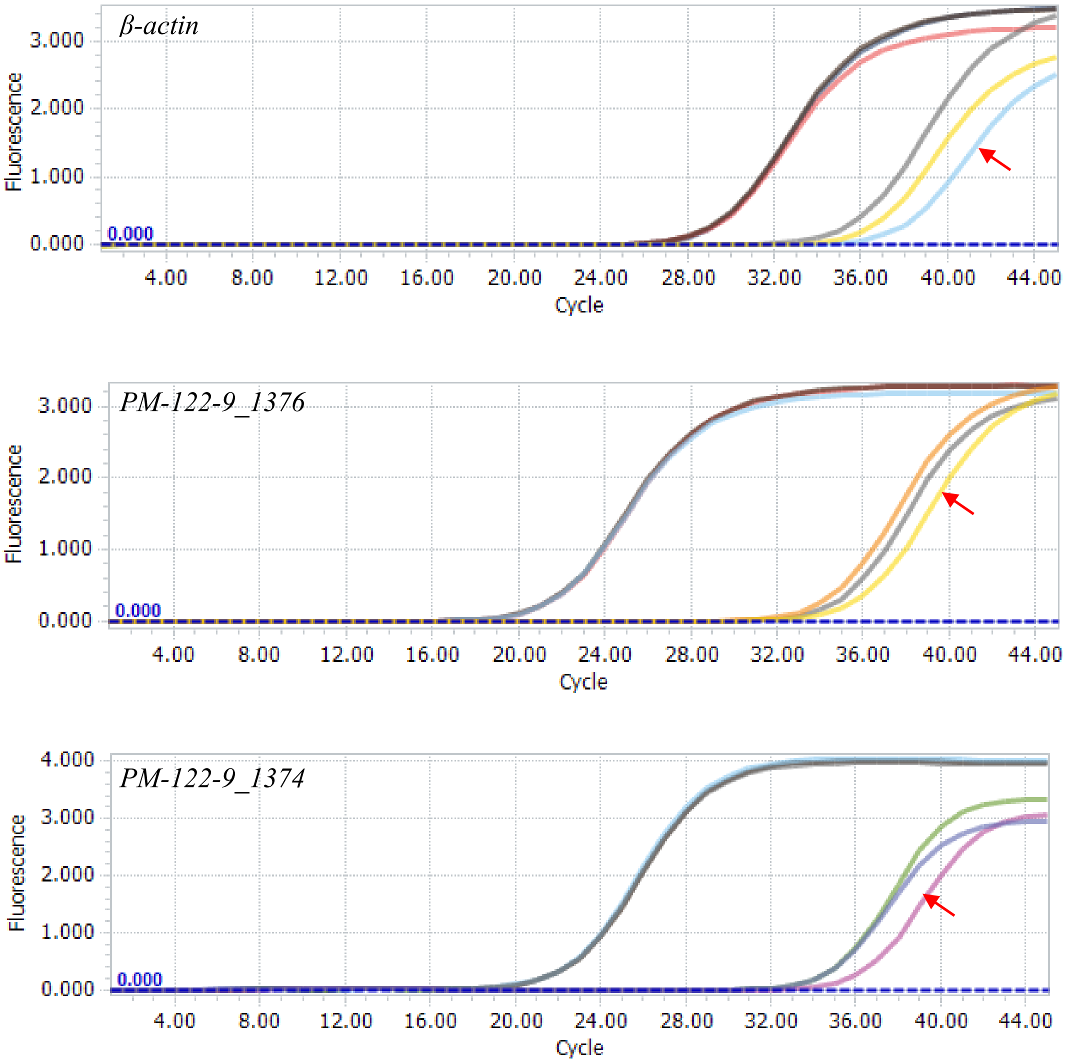

Figure S2. Amplification curves of *β-actin*, *PM-122-9_1376* and *PM-122-9_1374*

Note: Red scissors indicate NTC

```
AdrG_Penicillium_roqueforti_.txt      MTRGNEKEEAPPQAKILGSFPTGIAPYAELMRVHRLLGFY   40
AusN_Emericella_nidulans_.txt         MAVISELKRHHPKTGLLRYLPTGVVPYGELVRIHRALGYY   40
PrhE_Penicillium_brasilianum_.txt     MPTKGDYQ..PPKDGILSKLPESAVPYGELLRIHRPLGYY   38
Consensus                             m          p    l   p    py el r hr lg y

AdrG_Penicillium_roqueforti_.txt      LNTSPYLVGYAFCASISPTKIPITVLLHRTILLSIWSIFL   80
AusN_Emericella_nidulans_.txt         LNTSPYVVGIAYTAATAETKLPLDLLLDRLLLLTLWSLIL   80
PrhE_Penicillium_brasilianum_.txt     LNISPYVVGYAYTAAISPVTLPSTFLLGRLVILSLWGFCI   78
Consensus                             ln spy vg a  a       p   ll r   l  w

AdrG_Penicillium_roqueforti_.txt      RSAGCVWDDLIDMDLDSQISRTRTRPLPRGAVSPKNAFLL  120
AusN_Emericella_nidulans_.txt         RSAGCAWNDLVDVDIDRQISRTQSRPLPRGAISLSAATIF  120
PrhE_Penicillium_brasilianum_.txt     RSAGCAWNDLIDMDIDRQVSRTKLRPLPRGAVSPSGAALL  118
Consensus                             rsagc w dl d d d q srt  rplprga s   a

AdrG_Penicillium_roqueforti_.txt      TVTLFACGGSVLIYLPWPCAVDCLIITFFALLYPFGKRFT  160
AusN_Emericella_nidulans_.txt         TACLFVLGCSLLLFLPRECLFDAGIKVFFALLYPFGKRFT  160
PrhE_Penicillium_brasilianum_.txt     AAFMFGCGGSLLLLLPSQCAFEAAIVVFFALLYPFGKRFS  158
Consensus                                 f  g s l  lp  c     i  ffallypfgkrf

AdrG_Penicillium_roqueforti_.txt      DYPQITLVNIGWAIPMAMHSLGLDPLSQMKPTVCMFLFIG  200
AusN_Emericella_nidulans_.txt         DHPQLILINIAWAIPMAMHSLGMEPSSQILSMLCMCVFFS  200
PrhE_Penicillium_brasilianum_.txt     DHPQLILTNIAWAIPMAMSSLDMSPLDFPIPTLAMSFSIA  198
Consensus                             d pq  l ni waipmam sl   p         m

AdrG_Penicillium_roqueforti_.txt      LVIIMIDVIYSRQDTEEDLKVGVKSMAVRFRESIELLSYS  240
AusN_Emericella_nidulans_.txt         AVIVMIDLVYSRQDTEEDLKVGVKSMAVRYRNCVETMAYS  240
PrhE_Penicillium_brasilianum_.txt     SVIVMIDIVYACQDAEEDKKVGARSMAVRYMEITDQIAYG  238
Consensus                              vi mid  y  qd eed kvg  smavr         y

AdrG_Penicillium_roqueforti_.txt      LLYASTGFLAMAGFFTGLGLSFFVVSVGGHFCGFWVLLKA  280
AusN_Emericella_nidulans_.txt         LFAISSLALLFGGVLGGLRVPFVLFSVGGHIVGFWRFLRA  280
PrhE_Penicillium_brasilianum_.txt     LFFSGTLSLLVGGILRGLGFPFLIFSVGGHFLGFLRFLRA  278
Consensus                             l       l   g   gl   f   svggh  gf   l a

AdrG_Penicillium_roqueforti_.txt      TR..VGNSYGVESYAKSAFFLATLFWLFGFVIEYCLRN..  316
AusN_Emericella_nidulans_.txt         SL..QAGPAGVESRAKSSCLIASVFWVLGLGIEYAEMVKE  318
PrhE_Penicillium_brasilianum_.txt     SLGKGAKSAIVESQAKSSCLLATMLLVFGLCFEYCVRL..  316
Consensus                                       ves aks    a       g  ey

AdrG_Penicillium_roqueforti_.txt      ............                              316
AusN_Emericella_nidulans_.txt         KDNPTDKKKHPH                              330
PrhE_Penicillium_brasilianum_.txt     ............                              316
Consensus
```

Figure S3**.** Prenyltransferase gene sequence alignment between Prh, Aus, and Adr clusters. Red asterisks indicate the conserved sequence.

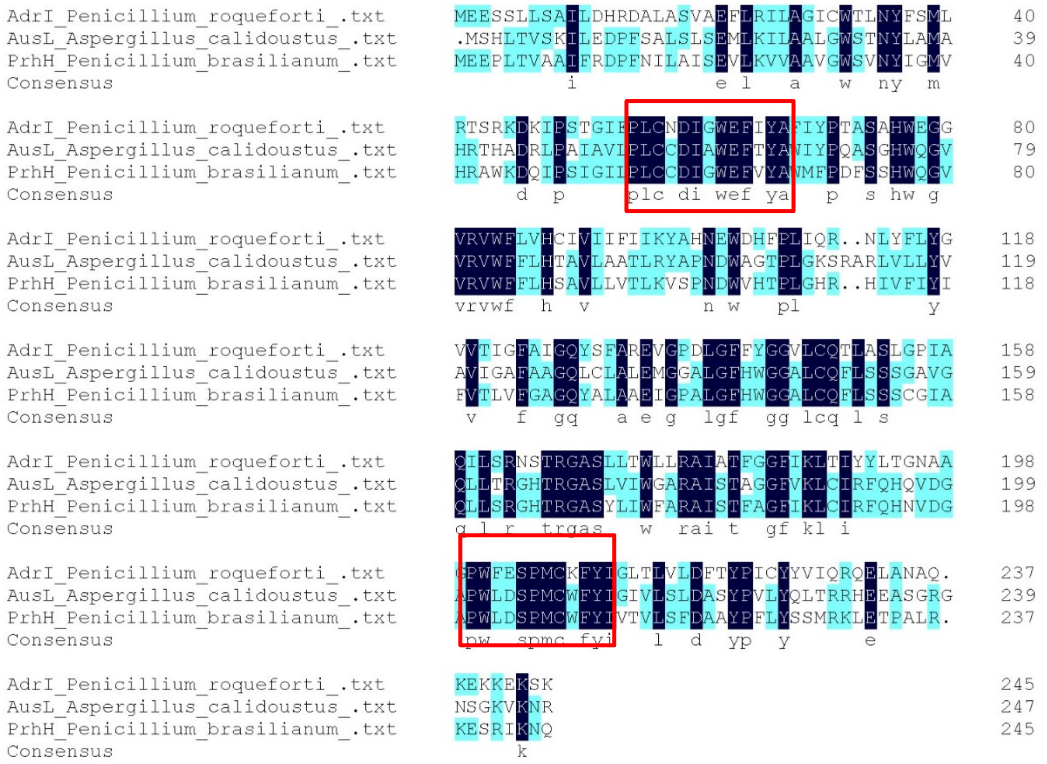

Figure S4. Terpene cyclase gene sequence alignment between Prh, Aus, and Adr clusters. Red asterisks indicate the conserved sequence.

5. Fermentation of the fungus.

The fermentation was carried out in 40 Fernbach flasks (500 mL), each containing 80 g of rice. Distilled H_2O (100 mL) was added to each flask, and the contents were soaked overnight before autoclaving at 15 psi for 30 min. After cooling to room temperature, each flask was inoculated with 5.0 mL of the spore inoculum and incubated at 25 °C for 25 days.

6. Extraction and isolation.

The fermented material was extracted with EtOAc (3 × 1 L), successively. The EtOAc extract was evaporated to dryness under reduced pressure to afford a crude residue (20.5 g). The crude extract was suspended in 90% MeOH in H_2O and then partitioned with petroleum ether (PE) three times to give 8 g of MeOH extract under vacuum. The MeOH portion (8 g) was then subjected to silica gel (200−300 mesh) vacuum liquid chromatography with gradient elution using PE-EtOAc (from 8:1 to 0:1, v/v) to obtain four fractions (Fr.1 to Fr.4). Fr. 3 (1.8 g) was chromatographed over a C_{18} silica gel column eluting with MeOH-H_2O (65:35, v/v) to afford six subfractions (F3a-F3f). F3b (126 mg) was chromatographed on a reversed phase (RP) HPLC column using MeOH-H_2O (50:50, v/v, 2 mL/min) to yield compounds **8** (8 mg), **1** (4

mg). F2c (220 mg) was purified RP-C_{18} HPLC column eluting with MeCN-H_2O (40:60, v/v, 2 mL/min) to afford compounds **2** (6 mg), **3** (6 mg) and **6** (2 mg). F3d (145 mg) was separated using RP-C_{18} HPLC column eluting with MeOH-H_2O (55:45, v/v, 2 mL/min) to afford compound **4** (5 mg). F3e (398 mg) was further subjected to a C_{18} silica gel column eluting with MeOH-H_2O (70:30, v/v) and followed by preparative RP-HPLC column eluting with MeOH-H_2O (60:40, v/v, 2 mL/min) to give compounds **5** (6 mg) and **7** (5 mg).

Brasilianoid A (1)

White amorphous powder; $[\alpha]^{20}_{D}$ –22 (*c* 0.5, MeOH); UV (MeOH) λ_{max} (log ε) (203) nm; IR v_{max} (KBr) cm^{-1}: 3419, 2985, 2935, 1754, 1720, 1708, 1662, 1227 and 1025. ^{1}H and ^{13}C NMR data, see Tables S2; HRESIMS *m/z* 473.2176 [M – H]$^-$ (calcd for $C_{26}H_{33}O_8$, 473.2175).

Brasilianoid B (2)

Colorless monoclinic crystals (MeOH-Acetone-H_2O, 5:5:1, v/v/v); m.p. 170–172 °C; $[\alpha]^{20}_{D}$ –40 (*c* 0.5, MeOH); UV (MeOH) λ_{max} (log ε) (206) nm; IR v_{max} (KBr) cm^{-1}: 3420, 2931, 2966, 1754, 1693, 1604, 1384 and 1043. ^{1}H and ^{13}C NMR data, see Table S3; HRESIMS *m/z* 473.2170 [M + HCOO]$^-$ (cald for $C_{26}H_{33}O_8$, 473.2175).

Brasilianoid C (3)

Colorless monoclinic crystals (MeOH-H_2O, 8:1, v/v); m.p. 171–173 °C; $[\alpha]^{20}_{D}$ –40 (*c* 0.5, MeOH); UV (MeOH) λ_{max} (log ε) (208) nm; IR v_{max} (KBr) cm^{-1}: 3420, 2930, 2876, 1760, 1694, 1604, 1385 and 1027. ^{1}H and ^{13}C NMR data, see Table S4; HRESIMS *m/z* 473.2181 [M + HCOO]$^-$ (cald for $C_{26}H_{33}O_8$, 473.2175).

Brasilianoid D (4)

Colorless monoclinic crystals; (MeOH-$CHCl_3$, 5:1, v/v); m.p. 255–258 °C; $[\alpha]^{20}_{D}$ –60 (*c* 0.5, MeOH); UV (MeOH) λ_{max} (log ε) (202) nm; IR v_{max} (KBr) cm^{-1}: 3420, 2965, 2856, 1750, 1661, 1634, 1384 and 1072. ^{1}H and ^{13}C NMR data, see Tables S5;

HRESIMS *m/z* 507.2590 [M + HCOO]$^-$ (calcd for $C_{27}H_{39}O_9$, 507.2594).

Brasilianoid E (5)

Colorless orthorhombic crystals (MeOH-$CHCl_3$-H_2O, 5:3:1, v/v/v); m.p. 225–228 °C; $[\alpha]^{20}_D$ –60 (*c* 0.5, MeOH); UV (MeOH) λ_{max} (log ε) (202) nm; IR v_{max} (KBr) cm^{-1}: 3445, 2966, 2939, 1750, 1662, 1607, 1385 and 1065. ^{1}H and ^{13}C NMR data, Table S6; HRESIMS *m/z* 507.2600 [M + HCOO]$^-$ (cald for $C_{27}H_{39}O_9$, 507.2594).

Brasilianoid F (6)

White amorphous powder; $[\alpha]^{20}_D$ –48 (*c* 0.5, MeOH); UV (MeOH) λ_{max} (log ε) (202) nm; IR v_{max} (KBr) cm^{-1}: 3419, 2986, 2949, 1754, 1720, 1603, 1383 and 1088. ^{1}H and ^{13}C NMR data, Table S7; HRESIMS *m/z* 443.2077 [M – H]$^-$ (cald for $C_{25}H_{31}O_7$, 443.2070).

(-) Preaustinoid D (7)

White amorphous powder; $[\alpha]^{20}_D$ –40 (*c* 0.2, MeOH); UV (MeOH) λ_{max} (log ε) (202) nm; IR v_{max} (KBr) cm^{-1}: 3523, 2970, 2885, 1735, 1709, 1662, 1644, 1222 and 1027. ^{1}H NMR (DMSO-d_6, 500 MHz) δ_H 1.40, 2.42 (m, H_2-1); 1.51 (m, H-2a), 2.25 (ddd, *J* = 4.0, 12.2, 15.7 Hz, H-2b), 1.21 (dd, *J* = 2.0, 11.5 Hz, H-5), 1.46 (m, H-6a), 1.65 (m, H-6b), 1.66 (m, H-7a), 1.99 (dt, *J* = 3.2, 13.2 Hz, H-7b), 0.52 (dd, *J* = 2.5, 13.6 Hz, H-9), 1.57 (t, *J* = 13.2 Hz, H-11a), 1.74 (dd, *J* = 2.3, 13.2 Hz, H-11b), 1.19 (s, H_3-12), 0.87 (s, H_3-13), 1.06 (s, H_3-14), 1.11 (s, H_3-15), 4.65 (s, H-7'a), 5.19 (s, H-7'b), 1.35 (s, H_3-8'), 1.23 (s, H_3-9'), 3.56 (s, OMe), 3.57 (s, OMe), 6.52 (s, OH-5'); ^{13}C NMR (DMSO-d_6, 125 MHz) δ_C 33.9 (C-1), 27.7 (C-2), 173.9 (C-3), 74.0 (C-4), 50.6 (C-5), 21.9 (C-6), 32.8 (C-7), 47.6 (C-8), 44.9 (C-9), 41.7 (C-10), 39.1 (C-11), 17.4 (C-12), 20.5 (C-13), 28.0 (C-14), 33.7 (C-15), 72.5 (C-1'), 144.8 (C-2'), 51.2 (C-3'), 209.7 (C-4'), 78.1 (C-5'), 205.1 (C-6'), 111.1 (C-7'), 22.1 (C-8'), 16.4 (C-9'), 169.7 (C-10'), 51.7 (OMe), 52.3 (OMe); HRESIMS *m/z* 491.2639 [M – H]$^-$ (calcd for $C_{26}H_{33}O_7$, 491.2645).

7. ECD calculation

Conformational searches were carried out by random searching in the Sybyl-X 2.0 using the MMFF94S force field with an energy cutoff of 5.0 kcal/mol.[1] Due to the confirmed NOESY correlations and relatively rigid skeleton, the results showed the lowest energy conformers: one for (1*R*, 2*S*, 4*R*, 5*S*, 8*S*, 9*R*, 10*S*, 1′*R*, 3′*R*, 5′*S*)-1′, one for (1*R*, 2*S*, 4*R*, 5*S*, 8*S*, 9*R*, 10*S*, 1′*R*, 3′*R*, 5′*R*, 6′*R*)-6′ and one for (5*S*, 8*S*, 9*R*, 10*S*, 1′*R*, 5′*S*)-7′ within 5.0 kcal/mol. Subsequently, the conformers were re-optimized using DFT at the B3LYP/6-31+G(d) level in gas phase by the GAUSSIAN 09 program.[2] The energies, oscillator strengths, and rotational strengths (velocity) of the first 60 electronic excitations were calculated using the TDDFT methodology at the b3lyp/6-311++g(d, p) level in vacuum. The ECD spectra were simulated by the overlapping Gaussian function (half the bandwidth at 1/e peak height, σ = 0.3 for **1**, **6** and **7**).[3] By comparison of the calculated ECD spectra with the experimental ones, the absolute configuration of **1**, **6** and **7** were resolved.

References

(1) Sybyl Software, version X 2.0; Tripos Associates Inc.: St. Louis, MO, 2013.

(2) Frisch, M. J.; Trucks, G. W.; Schlegel, H. B.; Scuseria, G. E.; Robb, M. A.; Cheeseman, J. R.; Scalmani, G.; Barone, V.; Mennucci, B.; Petersson, G. A.; Nakatsuji, H.; Caricato, M.; Li, X.; Hratchian, H. P.; Izmaylov, A. F.; Bloino, J.; Zheng, G.; Sonnenberg, J. L.; Hada, M.; Ehara, M.; Toyota, K.; Fukuda, R.; Hasegawa, J.; Ishida, M.; Nakajima, T.; Honda, Y.; Kitao, O.; Nakai, H.; Vreven, T.; Montgomery, Jr., J. A.; Peralta, J. E.; Ogliaro, F.; Bearpark, M.; Heyd, J. J.; Brothers, E.; Kudin, K. N.; Staroverov, V. N.; Kobayashi, R.; Normand, J.; Raghavachari, K.; Rendell, A.; Burant, J. C.; Iyengar, S. S.; Tomasi, J.; Cossi, M.; Rega, N.; Millam, J. M.; Klene, M.; Knox, J. E.; Cross, J. B.; Bakken, V.; Adamo, C.; Jaramillo, J.; Gomperts, R.; Stratmann, R. E.; Yazyev, O.; Austin, A. J.; Cammi, R.; Pomelli, C.; Ochterski, J. W.; Martin, R. L.; Morokuma, K.; Zakrzewski, V. G.; Voth, G. A.; Salvador, P.; Dannenberg, J. J.; Dapprich, S.; Daniels, A. D.; Farkas, Ö.; Foresman, J. B.; Ortiz, J. V.; Cioslowski, J.; Fox, D. J. Gaussian 09, Rev. C 01; Gaussian, Inc., Wallingford CT. **2009**.

(3) Stephens, P. J.; Harada, N. ECD cotton effect approximated by the Gaussian curve and other methods. *Chirality*. **2010**, *22*, 229–233.

7. Assay for inhibition toward nitric oxide production in RAW264.7 macrophages

The experiment was conducted according to a literature procedure.[1] Murine monocytic RAW264.7 cells grown in 96-well cell culture plates (2 × 10^5 cells/well) and pretreated with serial concentrations of the compounds **1–8** for 30 min, followed by stimulation with LPS (1 μg/mL) under a humidified atmosphere with 5% CO_2 at 37°C. After 24 h, the NO production of the supernatant was determined by adding the 100 μL of Griess reagent. Subsequently, the MTT [3-(4,5-dimethylthiazol-2-yl)-2,5-diphenyltetrazolium bromide] reduction[2] was used to evaluate the cell viability, and results were expressed as the mean value of triplicate determinations. The positive control was aminoguanidine.[2]

References

(1) Qin, J. J.; Jin, H. Z.; Zhu, J. X.; Fu, J. J.; Hu, X. J.; Liu, X. H.; Zhu, Y.; Yan, S. K.; Zhang, W. D. *Planta. Med.* **2010**, *76*, 278–283.

(2) Alley, M. C.; Scudiero, D. A.; Monks, A.; Hursey, M. L.; Czerwinski, M. J.; Fine, D. L.; Abbott, B. J.; Mayo, J. G.; Shoemaker, R. H.; Boyd, M. R. *Cancer. Res.* **1988**, *48*, 589–601.

8. Assay for *in vitro* anti-HBV effects

HepG2. 2. 15 cells were cultured with DMEM in 48-well plate at 1×10^5 cells/well for 24 h, then treated with 10 μM of compounds for 72 h. At d 4, these cells were washed 2 times with precooled PBS, and treated with 10 μM of compounds for 72 h. HBV progeny DNA of HepG2. 2. 15 cells were extracted using QIAamp DNA Blood Mini kit (Biomiga) according to the manufacturer's instruction. Then total DNA was reverse transcribed using PrimeScript RT reagent Kit (Takara, Dalian, China). The primers were designed and synthesized by Takara, and the sequences of the primers are indicated in Table S9. PCR amplification was performed on an StepOne Plus real time PCR system (Applied Biosystems, Foster City, CA) using the SYBR Green Master Mix (Applied Biosystems, Foster City, CA). All experiments were performed in triplicate, and the relative levels of assayed HBV DNA were calculated with the

delta–delta CT method using lamivudine expressions as positive control, and normalized to non-treated control.

9. Assay for protective effects on skin barrier functions in *in vitro*

HaCaT cells were cultured with DMEM in 6-well plate at 1×10^5 cells/well for 24 h, then treated with 20, 10, 5 μM of compounds **1**–**8** for 72 h. Total RNA of HaCaT cells were extracted using RNA Miniprep kit (Biomiga) according to the manufacturer's instruction. Then total RNA was reverse transcribed using PrimeScript RT reagent Kit (Takara, Dalian, China). The primers were designed and synthesized by Takara, the sequences of the primers are indicated in Table S10. PCR amplification was performed on an StepOne Plus real time PCR system (Applied Biosystems, Foster City, CA) using the SYBR Green Master Mix (Applied Biosystems, Foster City, CA). All experiments were performed in triplicate, and the relative levels of assayed mRNAs were calculated with the delta–delta CT method using ACTIN expressions as endogenous control, and normalized to non-treated control.

Table S3 ^{1}H and ^{13}C NMR data and HMBC correlations of **1** (DMSO-d_6)

No	^{13}C (pm)	^{1}H (ppm, J in Hz)	HMBC (H→C)
1	70.0	3.47, t (4.2)	C-2, C-3, C-5, C-9, C-10, C-13, C-15
2	48.0	2.50, dd (4.2, 4.9)	C-1, C-3, C-4, C-10, C-15
3	177.5		
4	86.8		
5	48.3	1.24, dd (1.7, 11.7)	C-4, C-6, C-7, C-10, C-13
6	18.6	1.43, dt (3.2, 13.0)<br>1.61, br ddd (1.7, 2.0, 13.0)	C-4, C-5, C-7, C-8, C-10<br>C-4, C-5, C-7, C-8, C-10
7	32.7	1.85, dt (3.2, 12.0)<br>2.07, ddd (2.0, 3.2, 12.0)	C-5, C-6, C-8, C-9, C-1'<br>C-5, C-6, C-8, C-9, C-1'
8	47.0		
9	44.0	1.50, dd (3.6, 12.9)	C-1, C-5, C-8, C-11, C-3'
10	42.6		
11	37.5	1.68, t (12.9)<br>1.74, dd (3.6, 12.9)	C-8, C-9, C-10, C-2', C-3, C-4'<br>C-8, C-9, C-10, C-2', C-3, C-4'
12	18.1	1.17, s	C-7, C-8, C-9, C-1'
13	17.6	0.76, s	C-1, C-5, C-9, C-10
14	23.0	1.29, s	C-4, C-5, C-15
15	40.7	1.81, br dd (4.9, 11.2)<br>2.24, d (11.2)	C-1, C-2, C-3, C-4, C-5<br>C-1, C-2, C-3, C-4, C-5
1'	72.6		
2'	144.1		
3'	50.5		
4'	208.0		
5'	76.9		
6'	206.0		
7'	112.3	4.67, s; 5.21, s	C-1', C-3'
8'	22.0	1.34, s	C-11, C-2', C-3', C-4'
9'	17.5	1.33, s	C-4', C-5', C-6'
10'	169.8		
MeO	52.2	3.57, s	C-10'
OH-1		5.16, d (4.2)	C-1, C-2, C-10
OH-5’		6.28, s	C-4', C-5', C-6'

Measured in 500 MHz

Table S4 ^{1}H and ^{13}C NMR data and HMBC correlations of **2** (DMSO-d_6)

No	^{13}C (pm)	^{1}H (ppm, *J* in Hz)	HMBC (H→C)
1	155.0	6.30, d (12.6)	C-3, C-5, C-9, C-10
2	119.8	5.76, d (12.6)	C-3, C-10
3	166.9		
4	85.1		
5	55.5	2.02, br d (12.1)	C-1, C-4, C-6, C-7, C-10, C-13
6	22.7	1.65, m<br>1.68, m	C-4, C-5, C-7, C-8, C-10<br>C-4, C-5, C-7, C-8, C-10
7	32.6	1.70, m<br>2.23, ddd (3.0, 12.0, 12.5)	C-5, C-6, C-8, C-9, C-1'<br>C-5, C-6, C-8, C-9, C-1'
8	41.3		
9	47.4	1.99, dd (2.0, 12.5)	C-1, C-5, C-8, C-11, C-3'
10	44.2		
11	39.4	1.84, dd (12.5, 13.0)<br>1.87, dd (2.0, 13.0)	C-8, C-9, C-10, C-2', C-3, C-4'<br>C-8, C-9, C-10, C-2', C-3, C-4'
12	18.8	1.18, s	C-7, C-8, C-9, C-1'
13	15.7	1.08, s	C-1, C-5, C-9, C-10
14	26.3	1.35, s	C-4, C-5, C-15
15	32.2	1.30, s	C-4, C-5, C-15
1'	66.5		
2'	147.6		
3'	55.3		
4'	213.4		
5'	76.4	4.30, q (6.4)	C-4', C-6', C-9', C-10'
6'	90.8		
7'	106.5	4.86, s; 5.09, s	C-1', C-3'
8'	16.3	1.20, s	C-11, C-2', C-3', C-4'
9'	13.2	1.14, d (6.4)	C-5', C-6'
10'	172.6		
OH-6'		6.95, s	C-1', C-4', C-5', C-6'

Measured in 500 MHz

Table S5 ^{1}H and ^{13}C NMR data and HMBC correlations of **3** (DMSO-d_6)

No	^{13}C (pm)	^{1}H (ppm, *J* in Hz)	HMBC (H→C)
1	155.6	6.31, d (12.2)	C-3, C-5, C-9, C-10
2	120.1	5.76, d (12.2)	C-3, C-10
3	167.0		
4	85.2		
5	55.7	1.94, dd (2.7, 12.7)	C-1, C-4, C-6, C-7, C-10, C-13
6	23.0	1.62, ddd (2.7, 3.0, 13.0)<br>1.75, ddt (3.0, 12.7, 13.0)	C-4, C-5, C-7, C-8, C-10<br>C-4, C-5, C-7, C-8, C-10
7	32.6	1.63, dt (3.0, 13.0)<br>2.30, dt (3.0, 13.0)	C-5, C-6, C-8, C-9, C-1'<br>C-5, C-6, C-8, C-9, C-1'
8	42.1		
9	47.2	2.10, br dd (4.1, 13.7)	C-1, C-5, C-8, C-11, C-3'
10	44.0		
11	39.7	1.84, t (13.7)<br>1.89, dd (4.1, 13.7)	C-8, C-9, C-10, C-2', C-3, C-4'<br>C-8, C-9, C-10, C-2', C-3, C-4'
12	18.5	1.20, s	C-7, C-8, C-9, C-1'
13	15.6	1.08, s	C-1, C-5, C-9, C-10
14	26.5	1.35, s	C-4, C-5, C-15
15	32.3	1.31, s	C-4, C-5, C-15
1'	65.0		
2'	148.8		
3'	55.7		
4'	215.2		
5'	83.5	4.46, q (7.2)	C-4', C-6', C-9', C-10'
6'	90.3		
7'	107.6	4.93, s; 5.14, s	C-1', C-3'
8'	15.9	1.16, s	C-11, C-2', C-3', C-4'
9'	18.2	1.03, d (7.2)	C-5', C-6'
10'	172.6		
OH-6'		7.09, s	C-1', C-4', C-5', C-6'

Measured in 500 MHz

Table S6 ^{1}H and ^{13}C NMR data and HMBC correlations of **4** (DMSO-d_6)

No	^{13}C (pm)	^{1}H (ppm, J in Hz)	HMBC (H→C)
1	34.3	1.32, m; 2.43, m	C-3, C-5, C-9, C-10
2	27.7	1.72, m; 2.42, m	C-3, C-10
3	174.3		
4	74.3		
5	51.1	1.29, m	C-1, C-4, C-6, C-7, C-10, C-13
6	22.5	1.49, m; 1.51, m	C-4, C-5, C-7, C-8, C-10
7	32.7	1.53, m<br>2.23, dt (3.0, 12.5)	C-5, C-6, C-8, C-9, C-1'<br>C-5, C-6, C-8, C-9, C-1'
8	42.2		
9	42.5	2.07, br dd (5.8, 12.1)	C-1, C-5, C-8, C-11, C-3'
10	41.1		
11	39.0	1.60, dd (5.8, 13.0)<br>1.63, dd (12.1, 13.0)	C-8, C-9, C-10, C-2', C-3, C-4'<br>C-8, C-9, C-10, C-2', C-3, C-4'
12	18.5	1.12, s	C-7, C-8, C-9, C-1'
13	20.3	0.87, s	C-1, C-5, C-9, C-10
14	28.0	1.10, s	C-4, C-5, C-15
15	33.9	1.13, s	C-4, C-5, C-15
1'	65.7		
2'	149.4		
3'	55.6		
4'	215.9		
5'	83.3	4.42, q (7.2)	C-4', C-6', C-9', C-10'
6'	90.3		
7'	106.8	4.89, s; 5.08, s	C-1', C-3'
8'	16.0	1.12, s	C-11, C-2', C-3', C-4'
9'	18.3	1.03, d (7.2)	C-5', C-6'
10'	172.8		
OMe	51.6	3.57, s	C-10'
OH-6'		7.04, s	C-1', C-4', C-5', C-6'
OH-4		4.09, s	

Measured in 500 MHz

Table S7 ^{1}H and ^{13}C NMR data and HMBC correlations of **5** (DMSO-d_6)

No	^{13}C (pm)	^{1}H (ppm, J in Hz)	HMBC (H→C)
1	34.3	1.33, m; 2.43, m	C-3, C-5, C-9, C-10
2	27.7	1.73, ddd (4.0, 12.5, 15.0)<br>2.42, m	C-3, C-10
3	174.3		
4	74.3		
5	51.2	1.29, dd (2.0, 12.0)	C-1, C-4, C-6, C-7, C-10, C-13
6	22.3	1.48, m; 1.50, m	C-4, C-5, C-7, C-8, C-10
7	32.7	1.54, m<br>2.15, dt (4.0, 12.3)	C-5, C-6, C-8, C-9, C-1'<br>C-5, C-6, C-8, C-9, C-1'
8	41.3		
9	42.8	1.92, dd (8.9, 13.0)	C-1, C-5, C-8, C-11, C-3'
10	41.1		
11	38.6	1.48, dd (8.9, 12.0)<br>1.57, dd (12.0, 13.0)	C-8, C-9, C-10, C-2', C-3, C-4'<br>C-8, C-9, C-10, C-2', C-3, C-4'
12	18.6	1.11, s	C-7, C-8, C-9, C-1'
13	20.4	0.88, s	C-1, C-5, C-9, C-10
14	27.9	1.10, s	C-4, C-5, C-15
15	34.0	1.13, s	C-4, C-5, C-15
1'	67.2		
2'	148.1		
3'	55.2		
4'	214.0		
5'	76.1	4.29, q (6.4)	C-4', C-6', C-9', C-10'
6'	90.9		
7'	105.8	4.82, s; 5.04, s	C-1', C-3'
8'	16.4	1.16, s	C-11, C-2', C-3', C-4'
9'	13.4	1.15, d (6.4)	C-5', C-6'
10'	172.8		
OMe	51.6	3.57, s	C-10'
OH-6'		6.76, s	C-1', C-4', C-5', C-6'

Measured in 500 MHz

Table S8 ^{1}H and ^{13}C NMR data and HMBC correlations of **6** (DMSO-d_6)

No	^{13}C (pm)	^{1}H (ppm, *J* in Hz)	HMBC (H→C)
1	70.3	3.50, t (4.5)	C-2, C-3, C-5, C-9, C-10, C-13, C-15
2	48.3	2.54, ddd (4.5, 5.0, 10.1)	C-1, C-3, C-4, C-10, C-15
3	177.8		
4	87.0		
5	48.2	1.67, dd (2.0, 12.0)	C-4, C-6, C-7, C-10, C-13
6	19.4	1.51, m<br>1.62, m	C-4, C-5, C-7, C-8, C-10<br>C-4, C-5, C-7, C-8, C-10
7	33.6	1.63, m<br>2.16, dt (4.1, 12.7)	C-5, C-6, C-8, C-9, C-1'<br>C-5, C-6, C-8, C-9, C-1'
8	41.5		
9	43.1	2.59, dd (3.8, 13.7)	C-1, C-5, C-8, C-11, C-3'
10	42.7		
11	38.2	1.56, dd (11.7, 13.7)<br>1.93, dd (3.8, 11.7)	C-8, C-9, C-10, C-2', C-3, C-4'<br>C-8, C-9, C-10, C-2', C-3, C-4'
12	20.2	1.14, s	C-7, C-8, C-9, C-1'
13	17.9	0.78, s	C-1, C-5, C-9, C-10
14	23.3	1.30, s	C-4, C-5, C-15
15	41.1	1.87, dd (5.0, 11.1)<br>2.29, brd (11.1)	C-1, C-2, C-3, C-4, C-5<br>C-1, C-2, C-3, C-4, C-5
1'	66.8		
2'	148.4		
3'	55.3		
4'	213.0		
5'	76.2	4.21, q (6.4, 6.4, 6.4)	C-1', C-4', C-6', C-9, C-10'
6'	90.7		
7'	105.9	4.81, s; 5.03, s	C-1', C-3'
8'	16.5	1.16, s	C-11, C-2', C-3', C-4'
9'	13.3	1.15, d (6.4)	C-5', C-6'
10'	172.8		
OH-1		5.16, d (4.5)	C-1, C-2, C-10
OH-6'		6.68, s	C-1', C-4', C-5', C-6'

Measured in 500 MHz

Table S9. Inhibitory effects against LPS induced NO production in RAW264.7 macrophages.

compounds	IC_{50} (μM)[a]	CC_{50} (μM)[b]
**1**	>50	>50
**2**	37.69	>50
**3**	33.76	>50
**4**	>50	>50
**5**	>50	>50
**6**	>50	>50
**7**	>50	>50
**8**	>50	>50
aminoguanidine[c]	7.62	>50

[a] IC_{50}: 50% inhibitory concentration

[b] CC_{50}: 50% cytotoxic concentration

[c] Positive control

Table S10. Primers of HBV

Name of primer	Sequence (5'-3')
HBV-F	CAACCTCCAATCATCACCAAC
HBV-R	ACGGGCAACATACCTTGGTAG

Table S11. Primers of Filaggrin and Caspase 14

Name of primer	Sequence (5'-3')
Filaggrin-F	CCCAGGTCCCATCAAGAAGA
Filaggrin-R	TGAGTCTGTGGAGCTGTCTG
Caspase14-F	GCACCATGAAAAGAGACCCC
Caspase14-R	TCTCCAGCTTGACCATCTCC

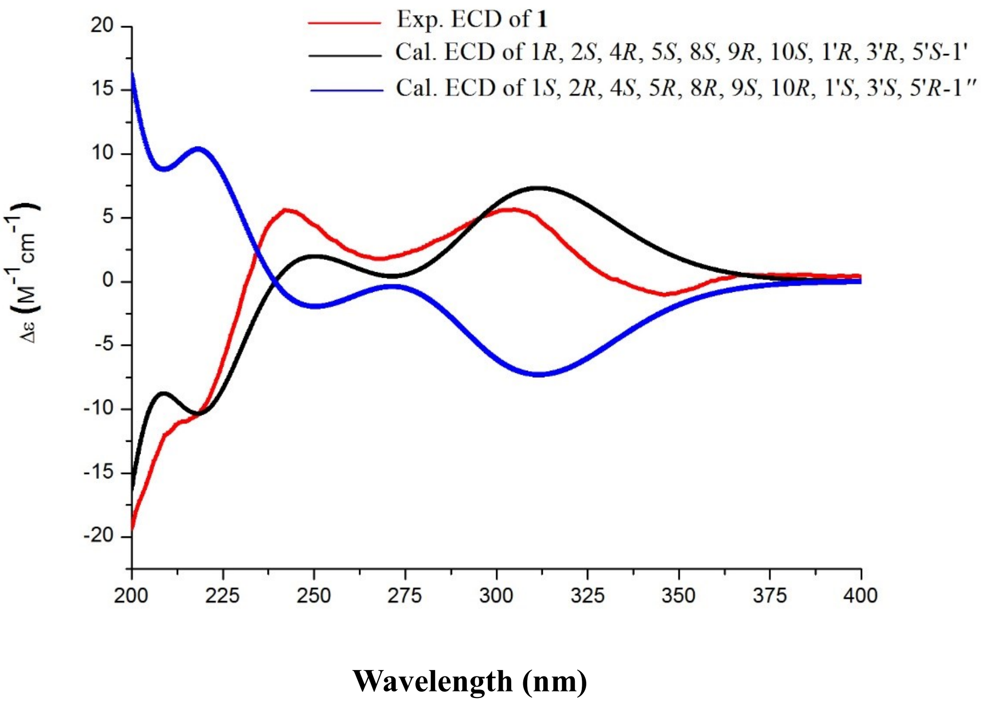

Figure S5. Experimental ECD spectra (200–400 nm) of **1** in MeOH and the calculated ECD spectra of the model molecules of **1** at the B3LYP/6-311++G(d, p) level**.**

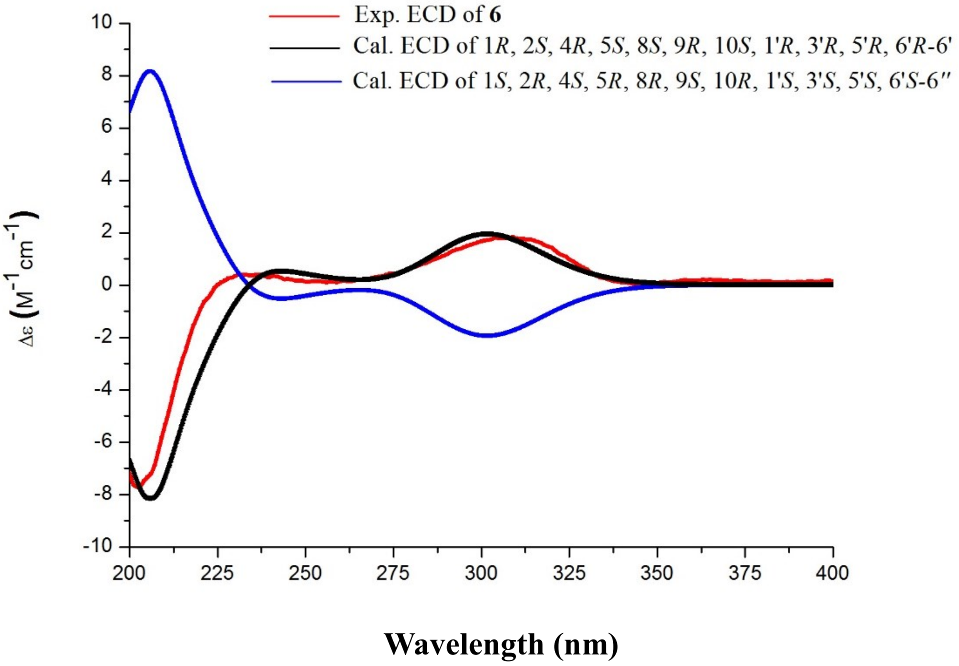

Figure S6. Experimental ECD spectra (200–400 nm) of **6** in MeOH and the calculated ECD spectra of the model molecules of **6** at the B3LYP/6-311++G(d, p) level.

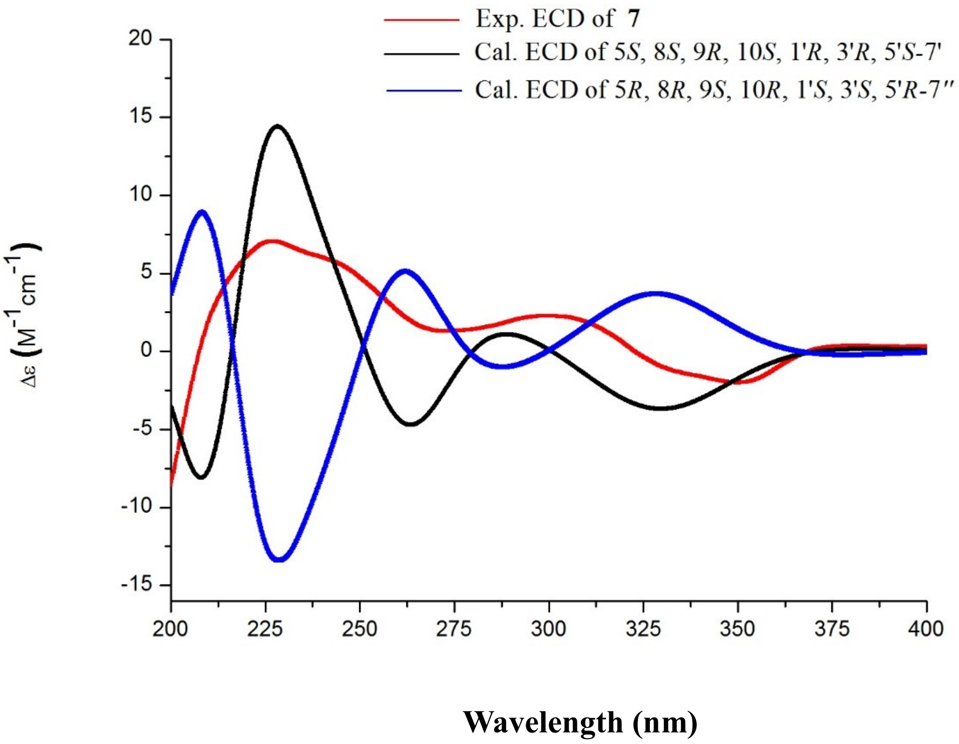

Figure S7. Experimental ECD spectra (200–400 nm) of **7** in MeOH and the calculated ECD spectra of the model molecules of **7** at the B3LYP/6-311++G(d, p) level.

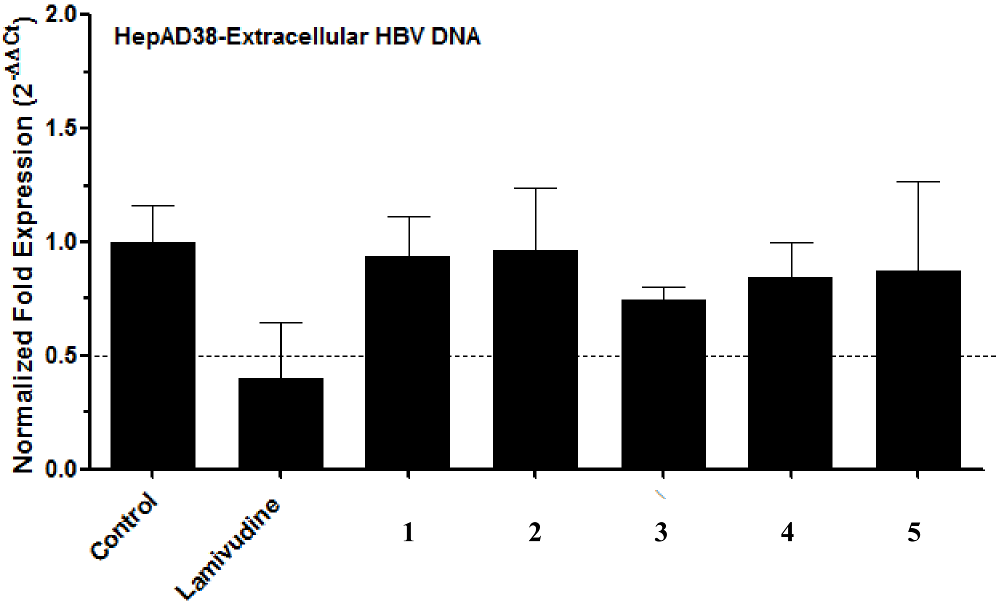

Figure S8**.** Inhibition of DNA expression of HBV in HepG2. 2. 15 cells

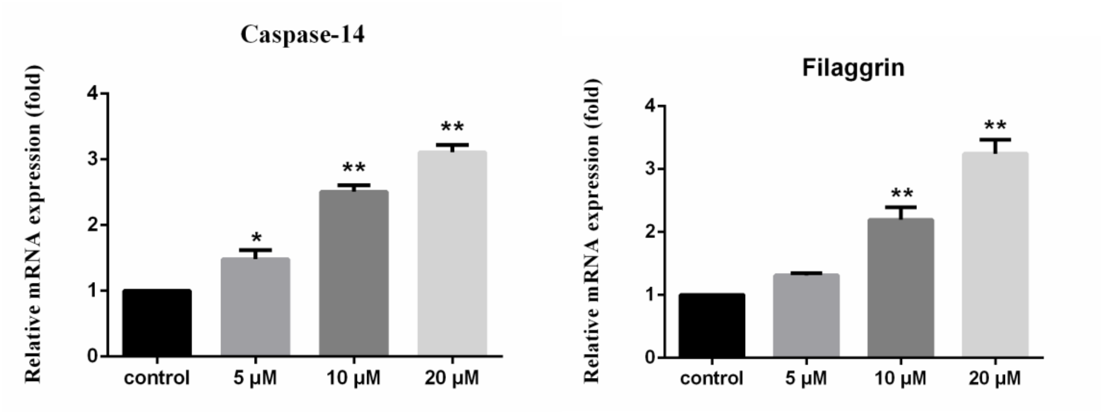

Compound 1

Figure S9. Promotion mRNA expression of caspase 14 and filaggrin in HaCaT cells

Figure S10. HR-ESIMS spectrum of 1

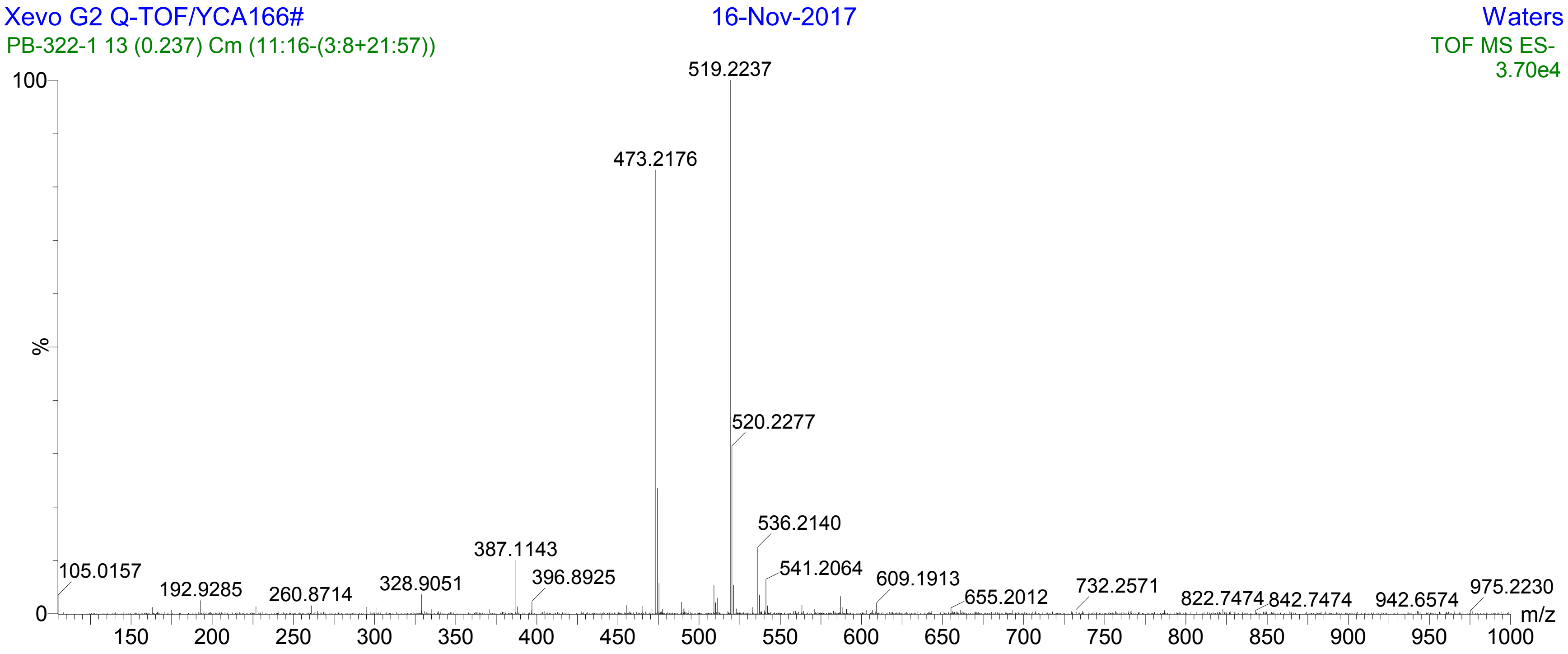

Mass	Calc. Mass	mDa	PPM	DBE	i-FIT	Norm	Conf(%)	Formula
473.2176	473.2175	0.1	0.2	10.5	57.5	0.082	92.09	$C_{26}H_{33}O_8$

Figure S11.IR spectrum of 1

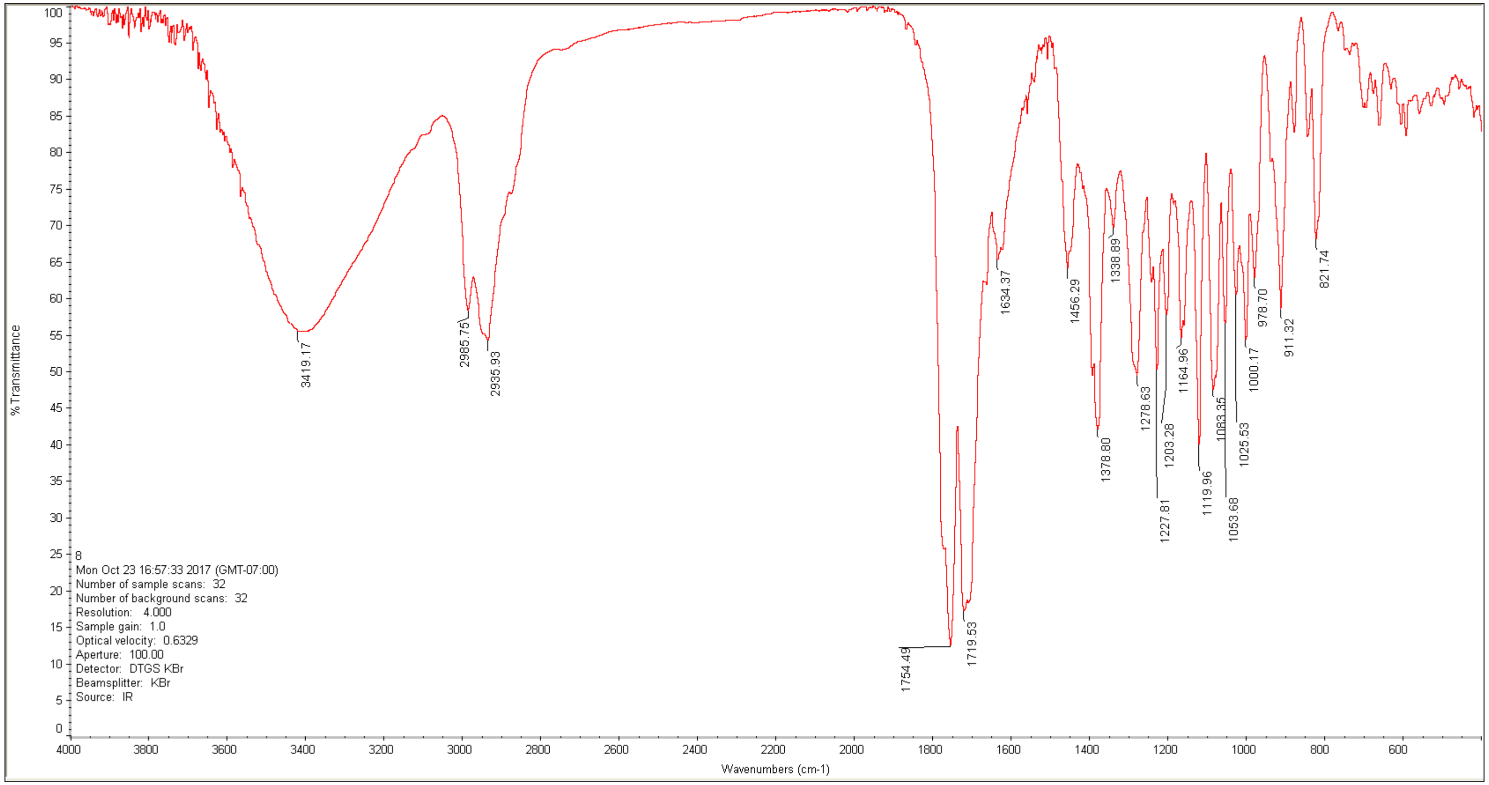

Figure S12. UV spectrum of 1 in CH_3OH

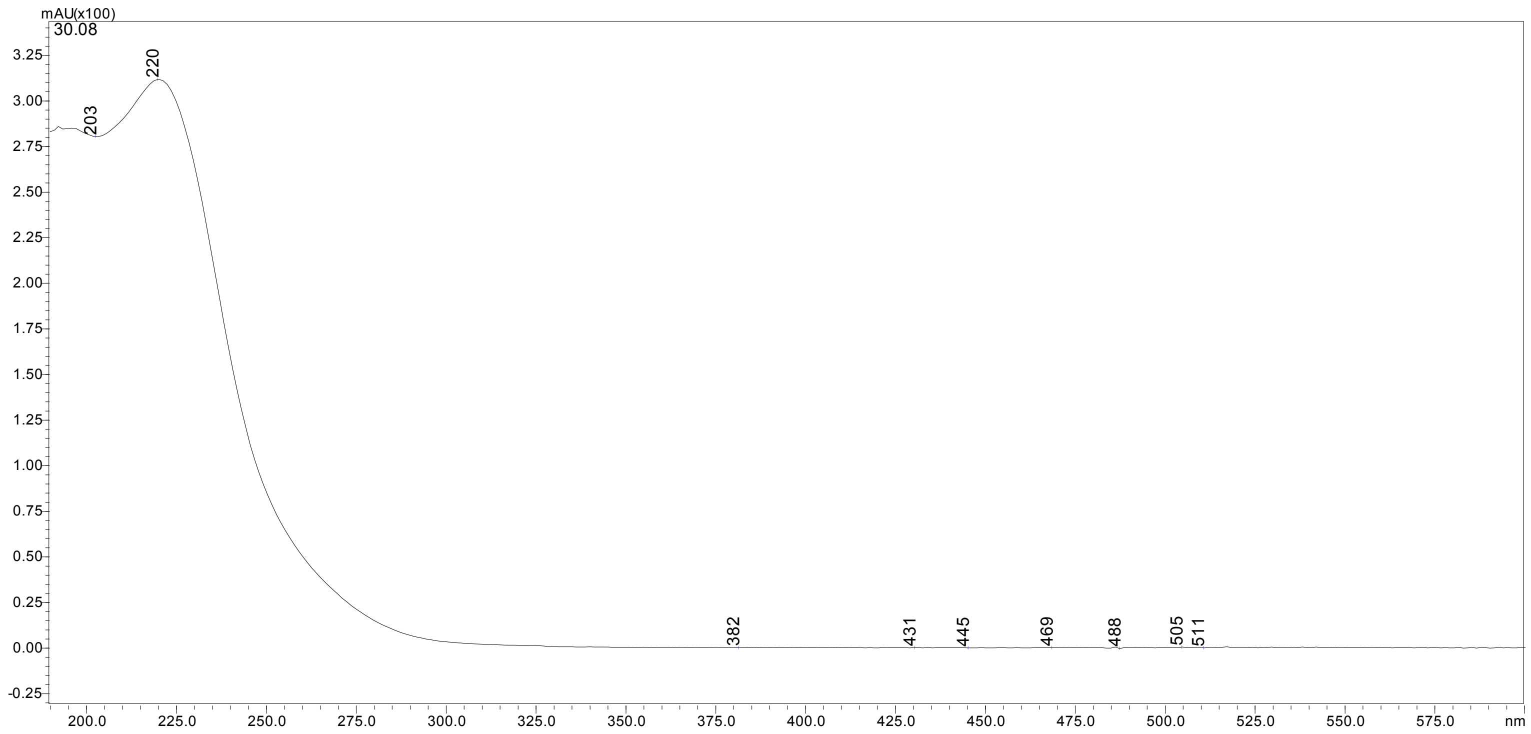

Figure S13. ^{1}H NMR spectrum of 1 in DMSO-d_6

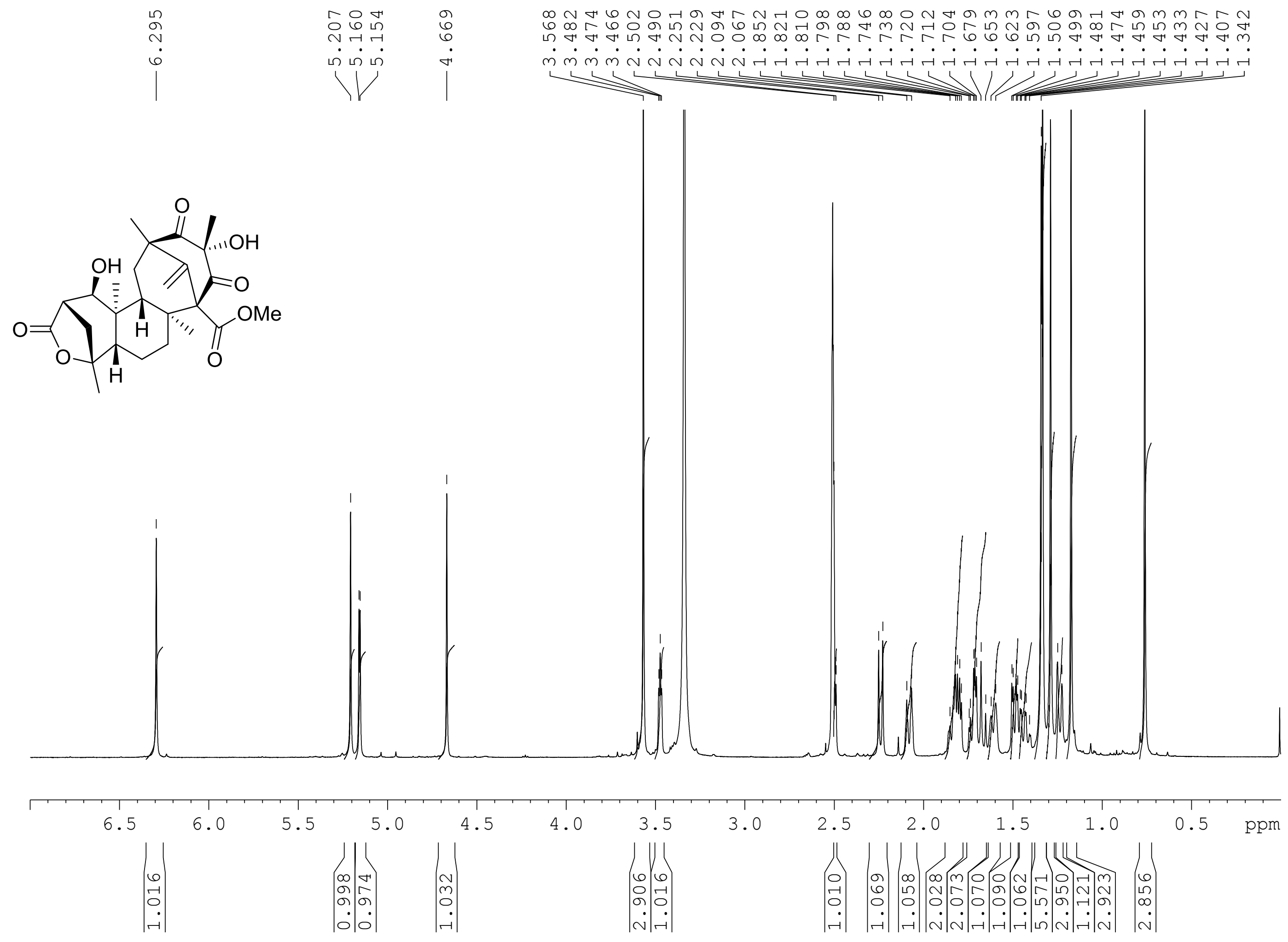

Figure S14. APT spectrum of 1 in DMSO-d_6

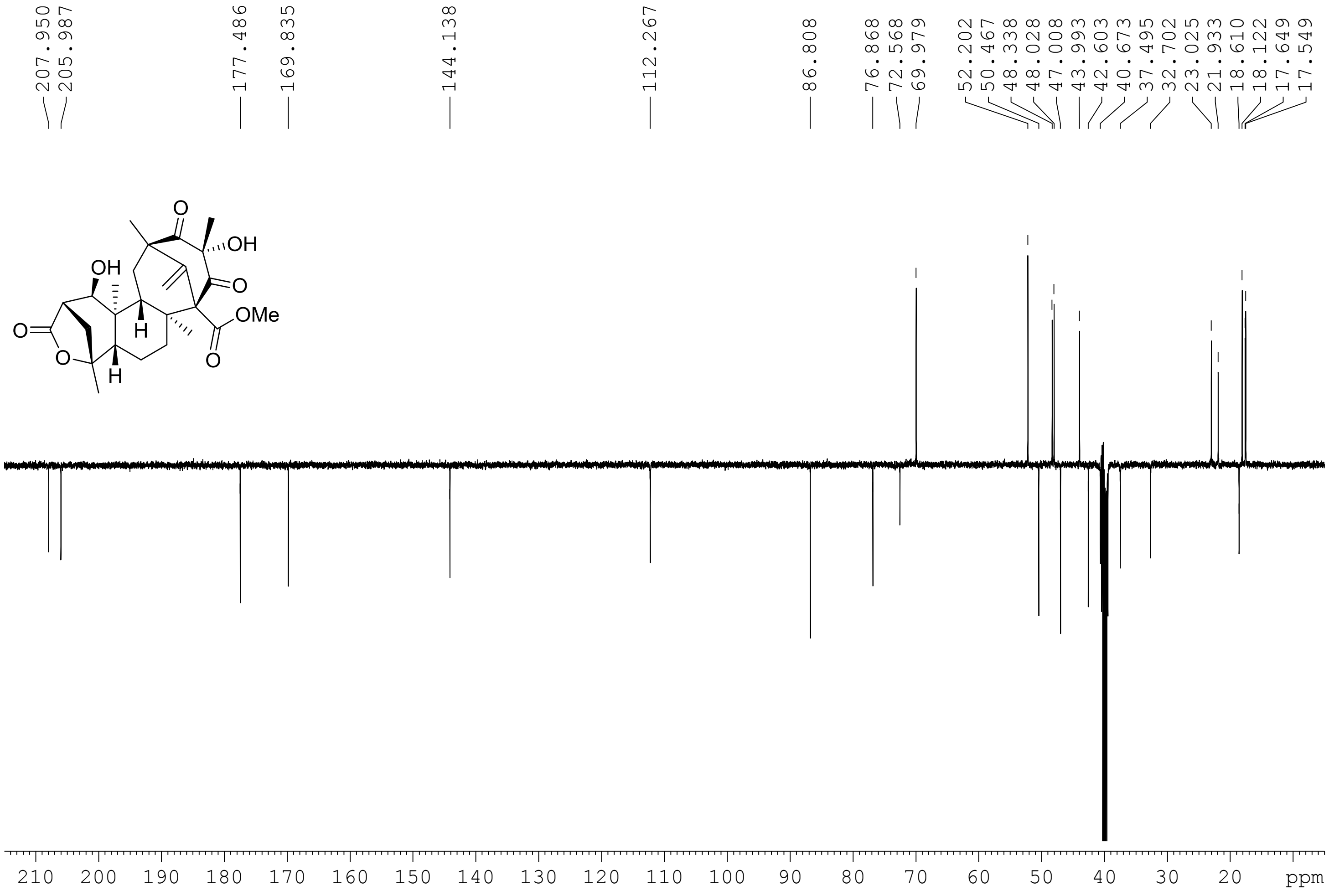

Figure S15. ^{1}H-^{1}H COSY spectrum of 1 in DMSO-d_6

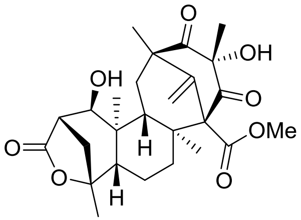

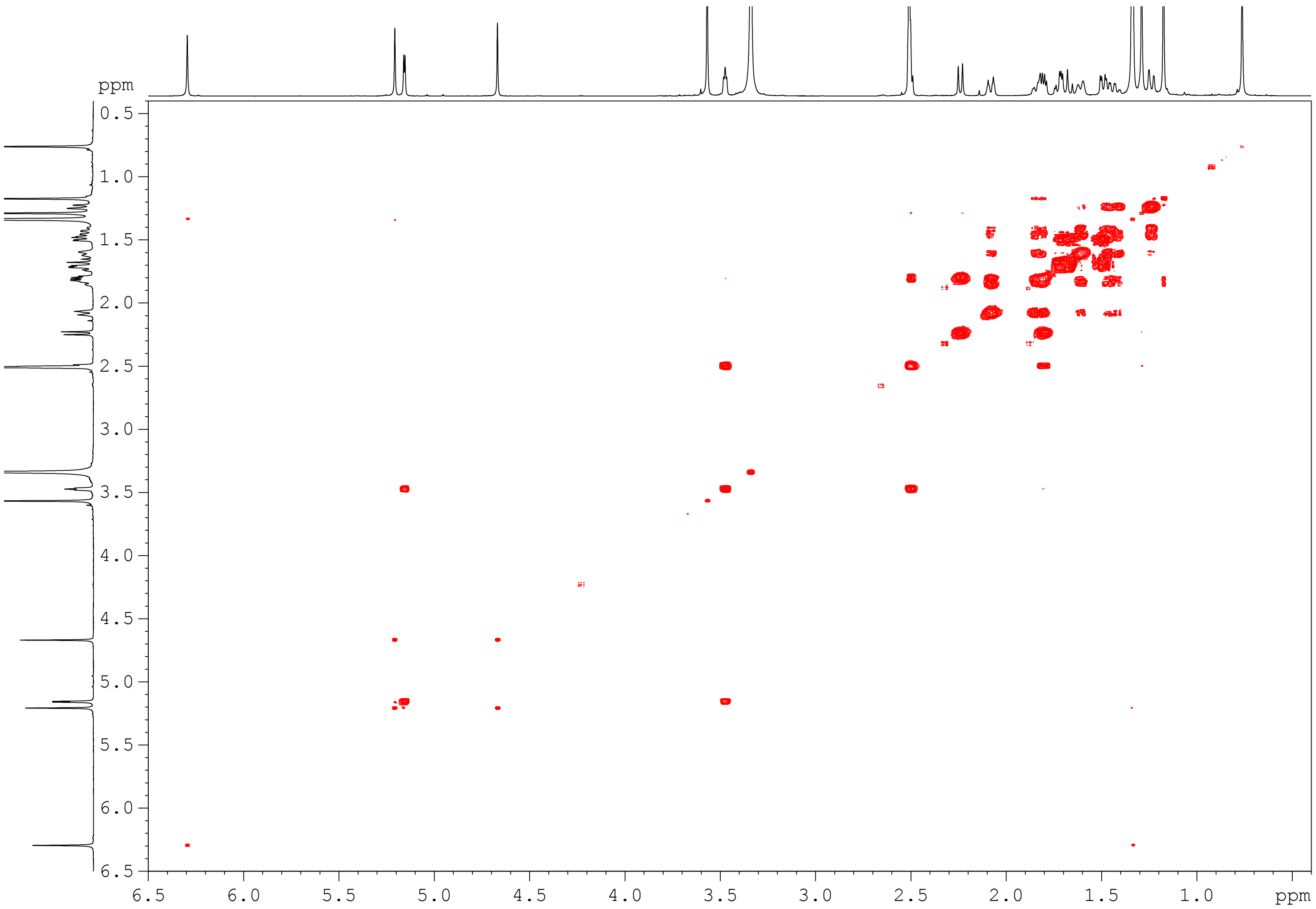

Figure S16. HSQC spectrum of 1 in DMSO-d_6

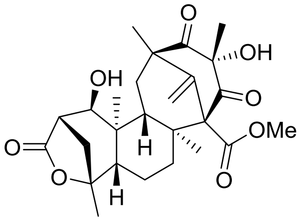

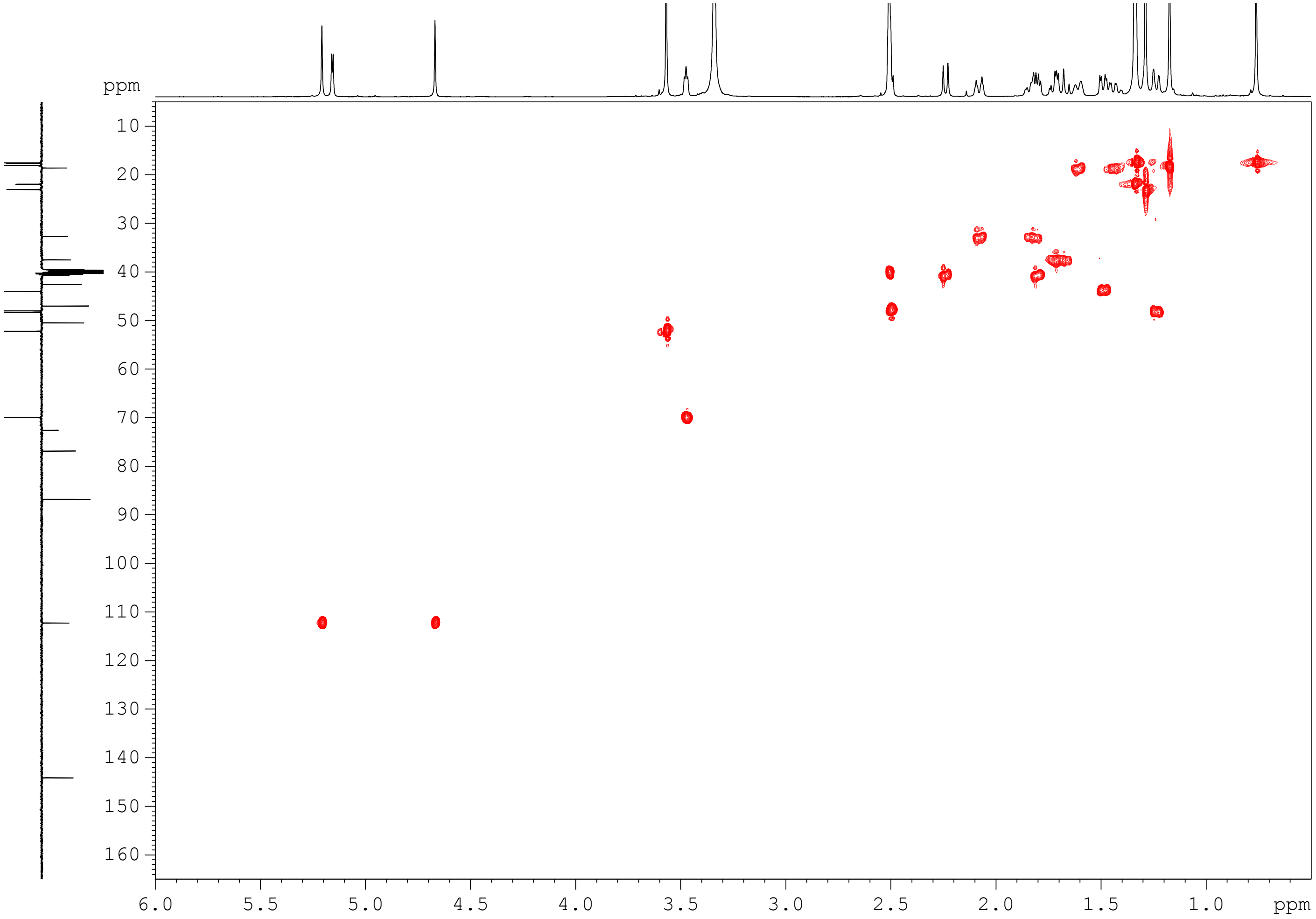

Figure S17. HMBC spectrum of 1 in DMSO-d_6

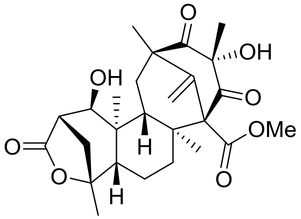

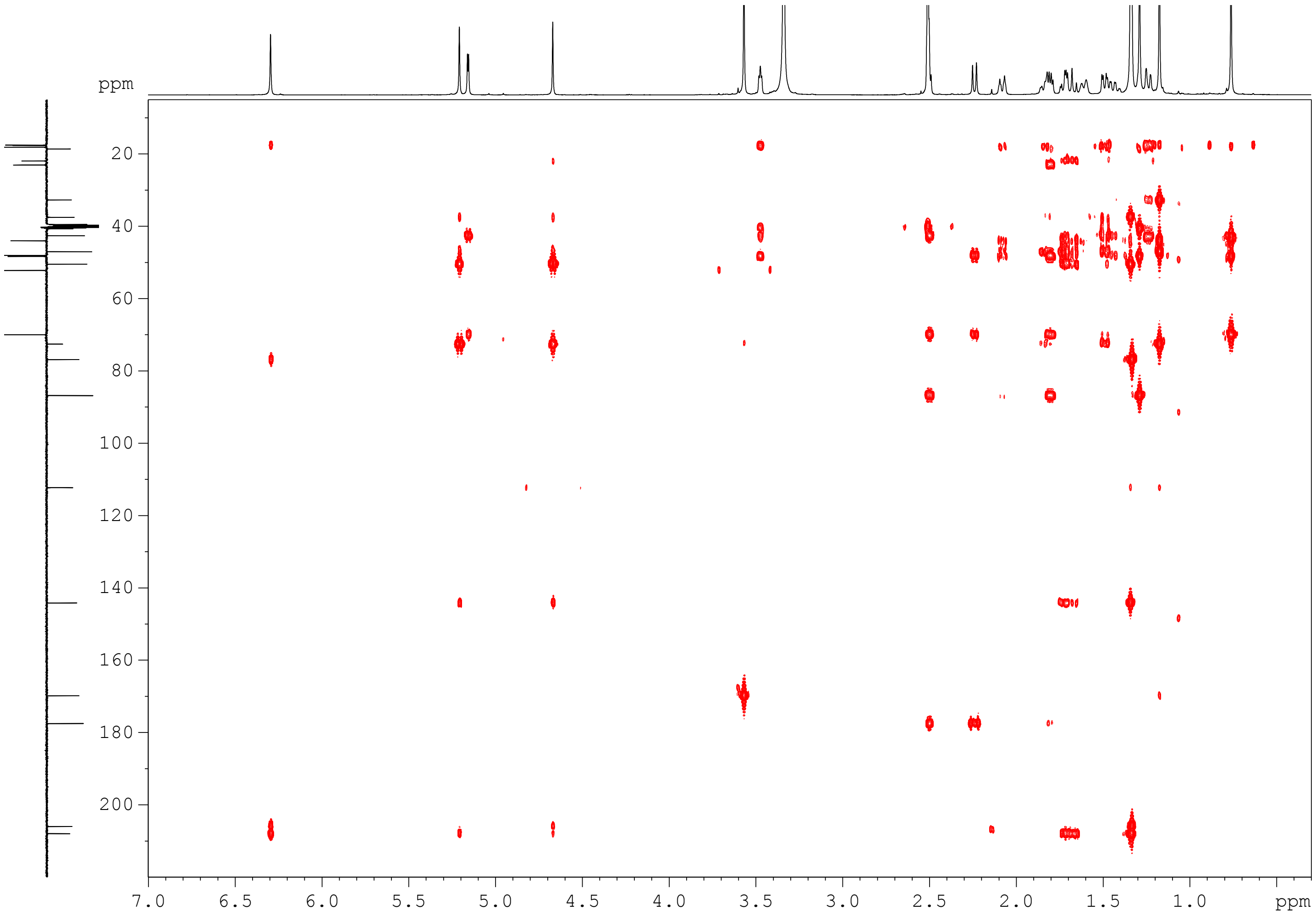

Figure S18. NOESY spectrum of 1 in DMSO-d_6

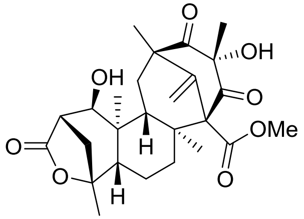

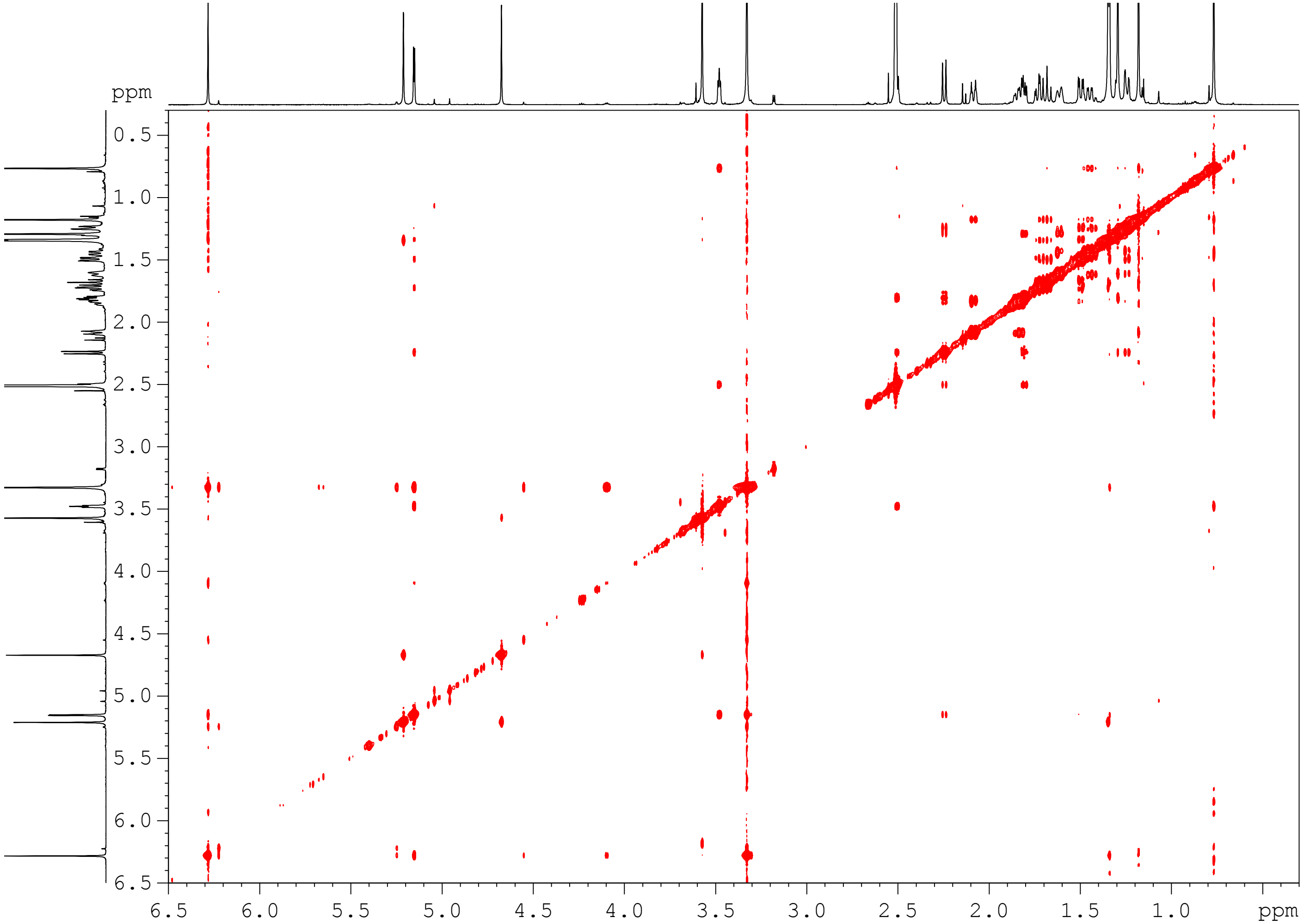

Figure S19. NOESY spectrum of 1 in DMSO-d_6 showing the key correlations from OH-1 to H_2-15, H-5, H-9 and H_3-9'; H_2-7' to H_3-12

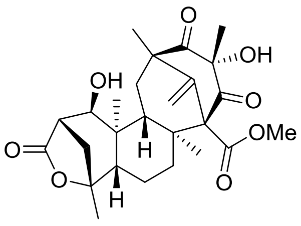

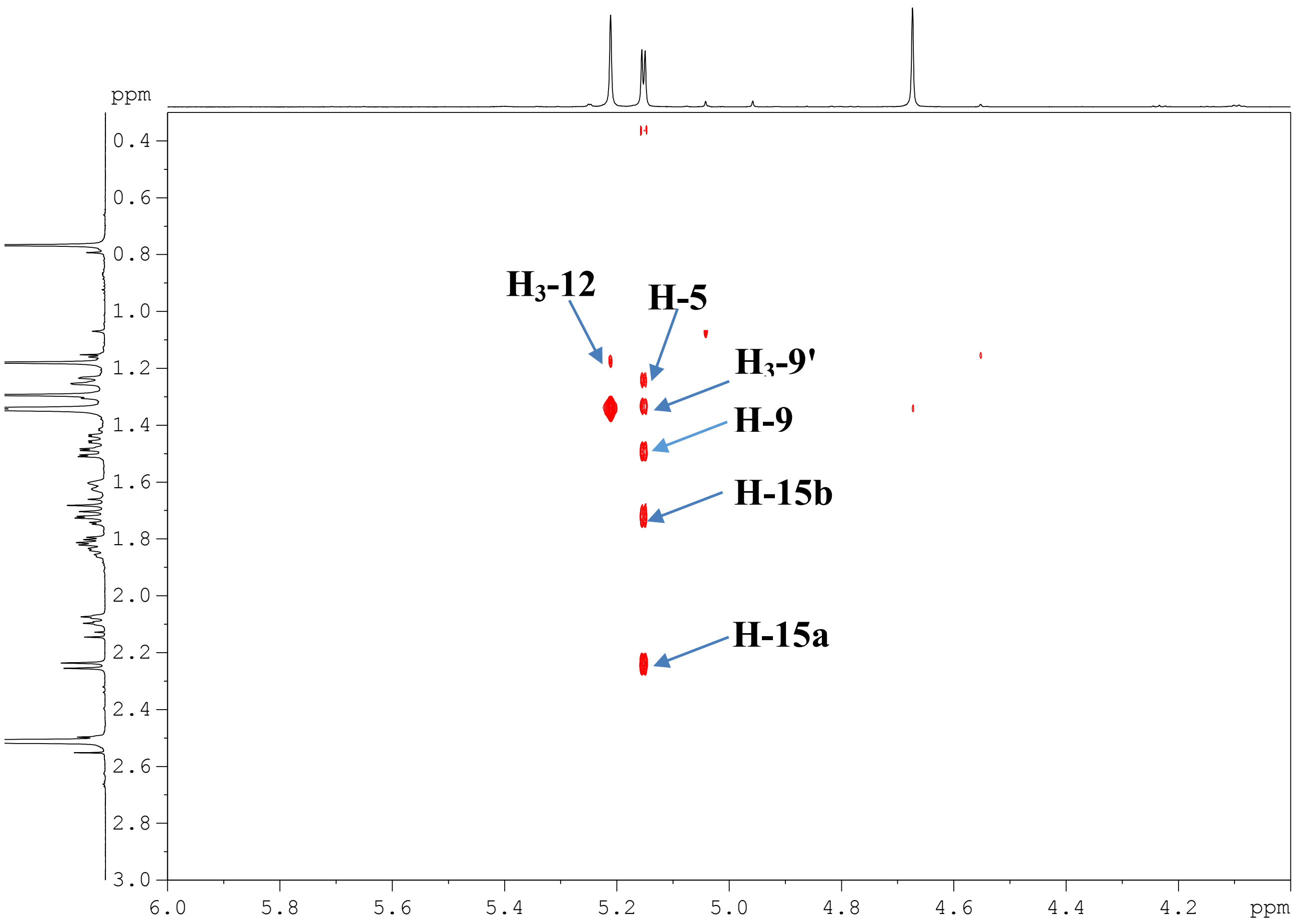

Figure S20. NOESY spectrum of 1 in DMSO-d_6 showing the key correlations from H-9 to H-5 and H_3-9'

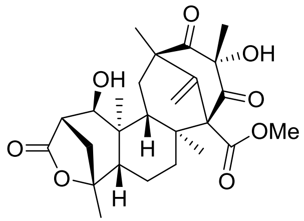

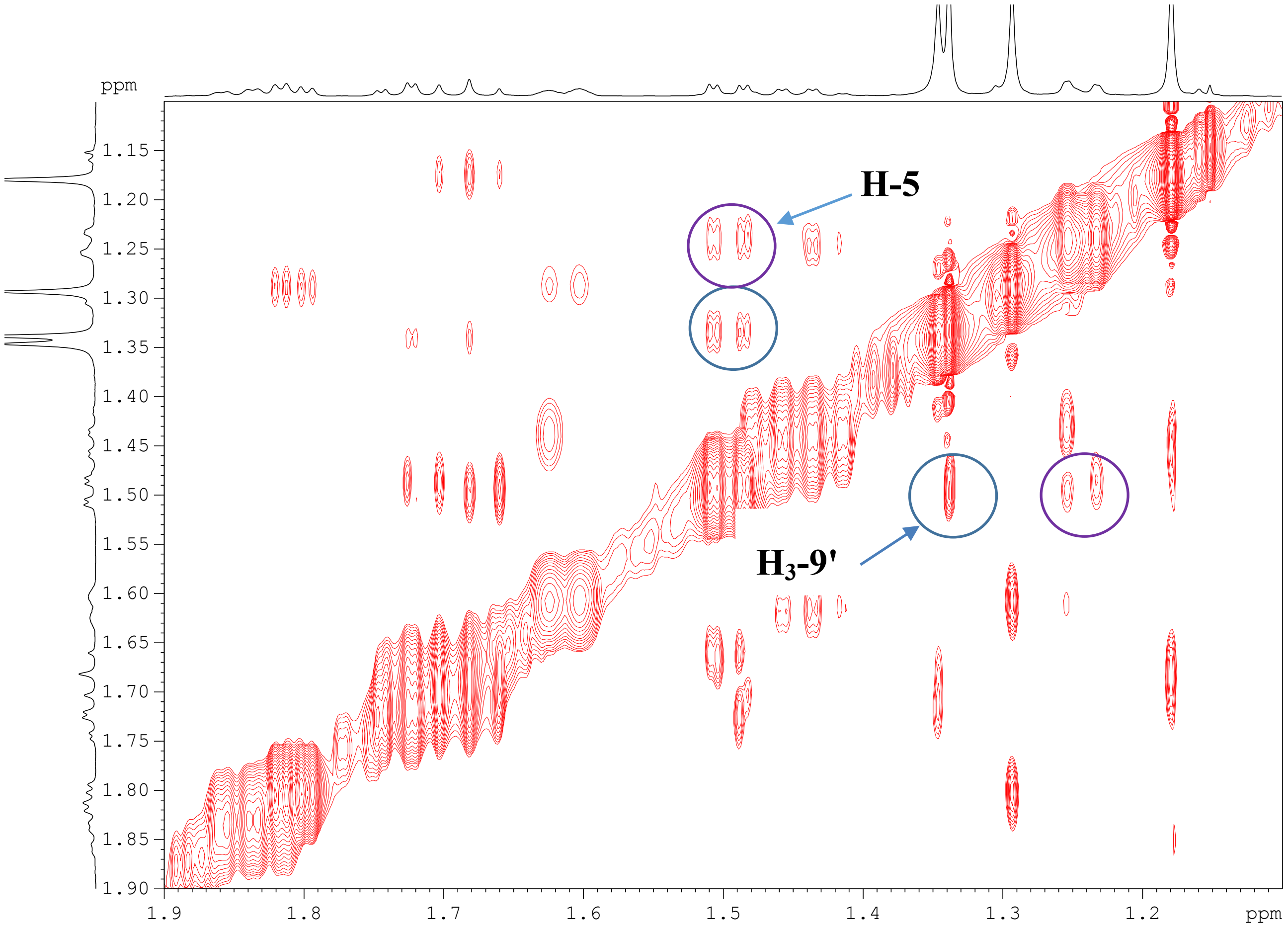

Figure S21. NOESY spectrum of 1 in DMSO-d_6 showing the key correlations from OH-5' to H_3-12, H_3-13 and H_3-8'

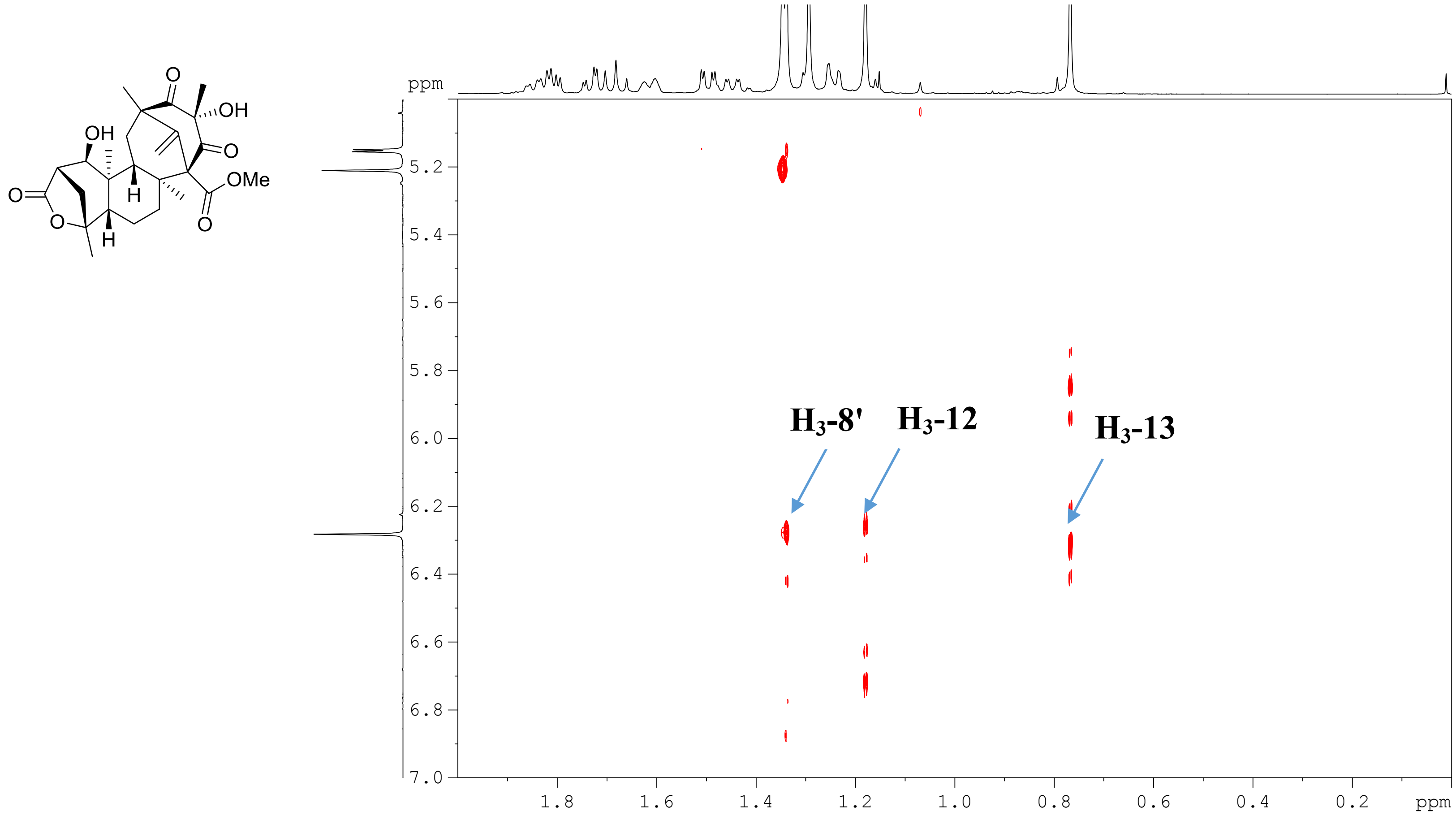

Figure S22. HR-ESIMS spectrum of 2

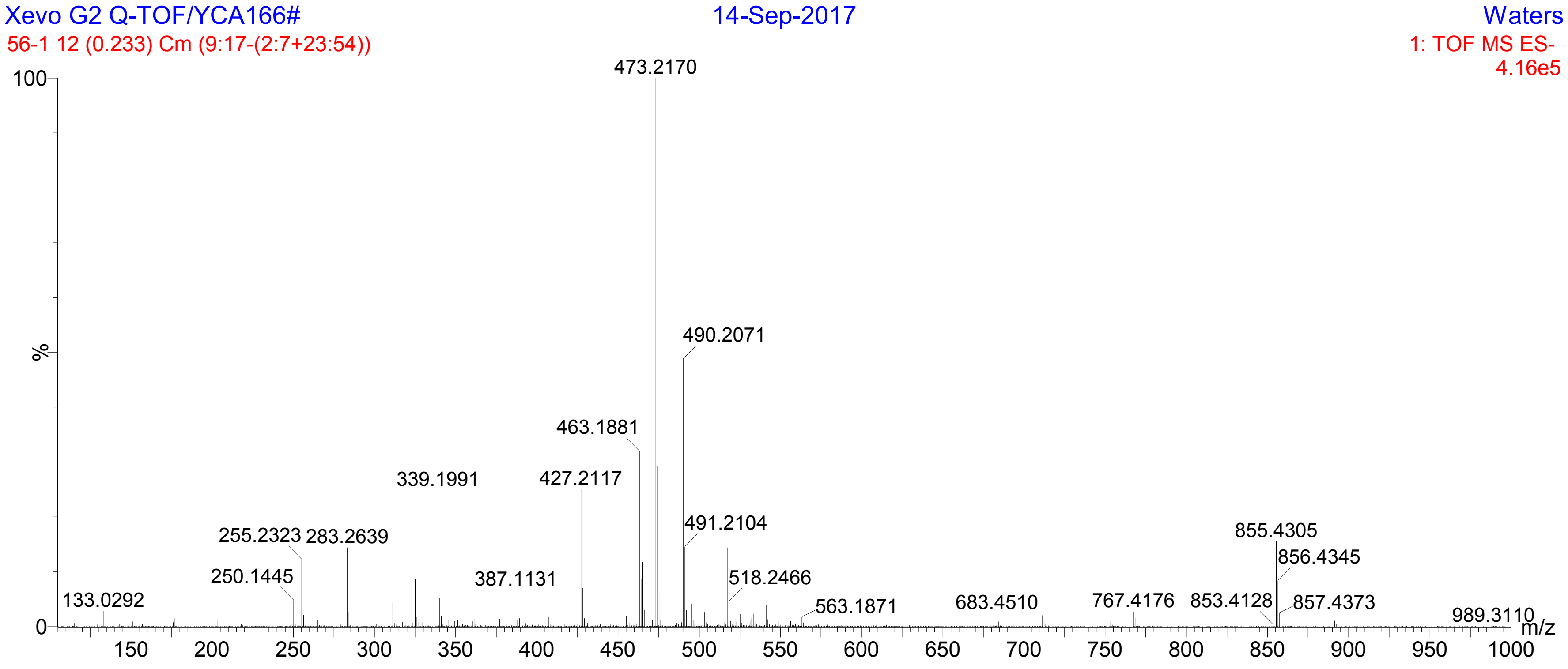

Mass	Calc. Mass	mDa	PPM	DBE	i-FIT	Norm	Conf(%)	Formula
473.2170	473.2175	-0.5	-1.1	10.5	185.7	0.000	100.00	$C_{26}H_{33}O_8$

Figure S23. IR spectrum of 2

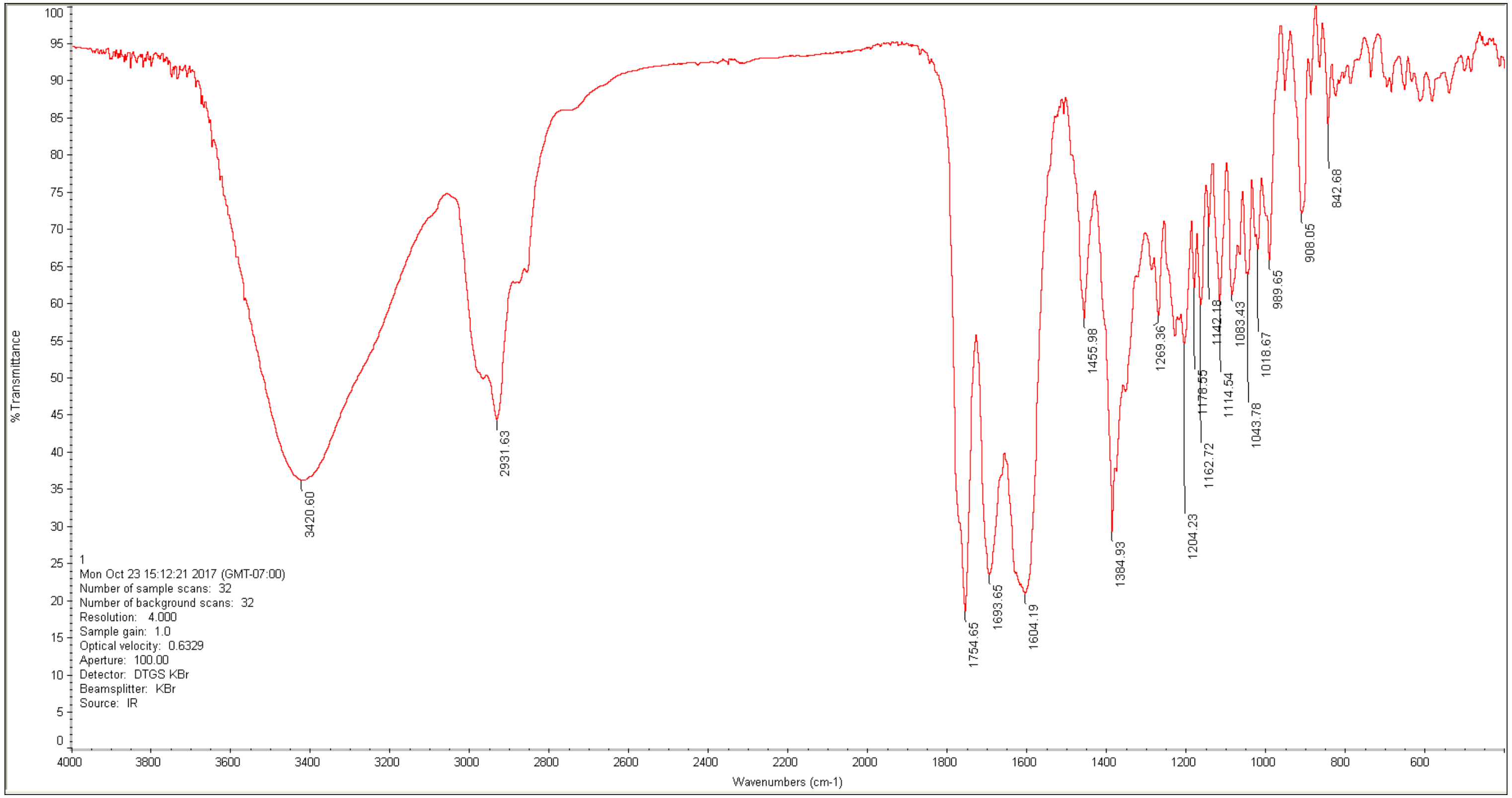

Figure S24. UV spectrum of 2 in CH_3OH

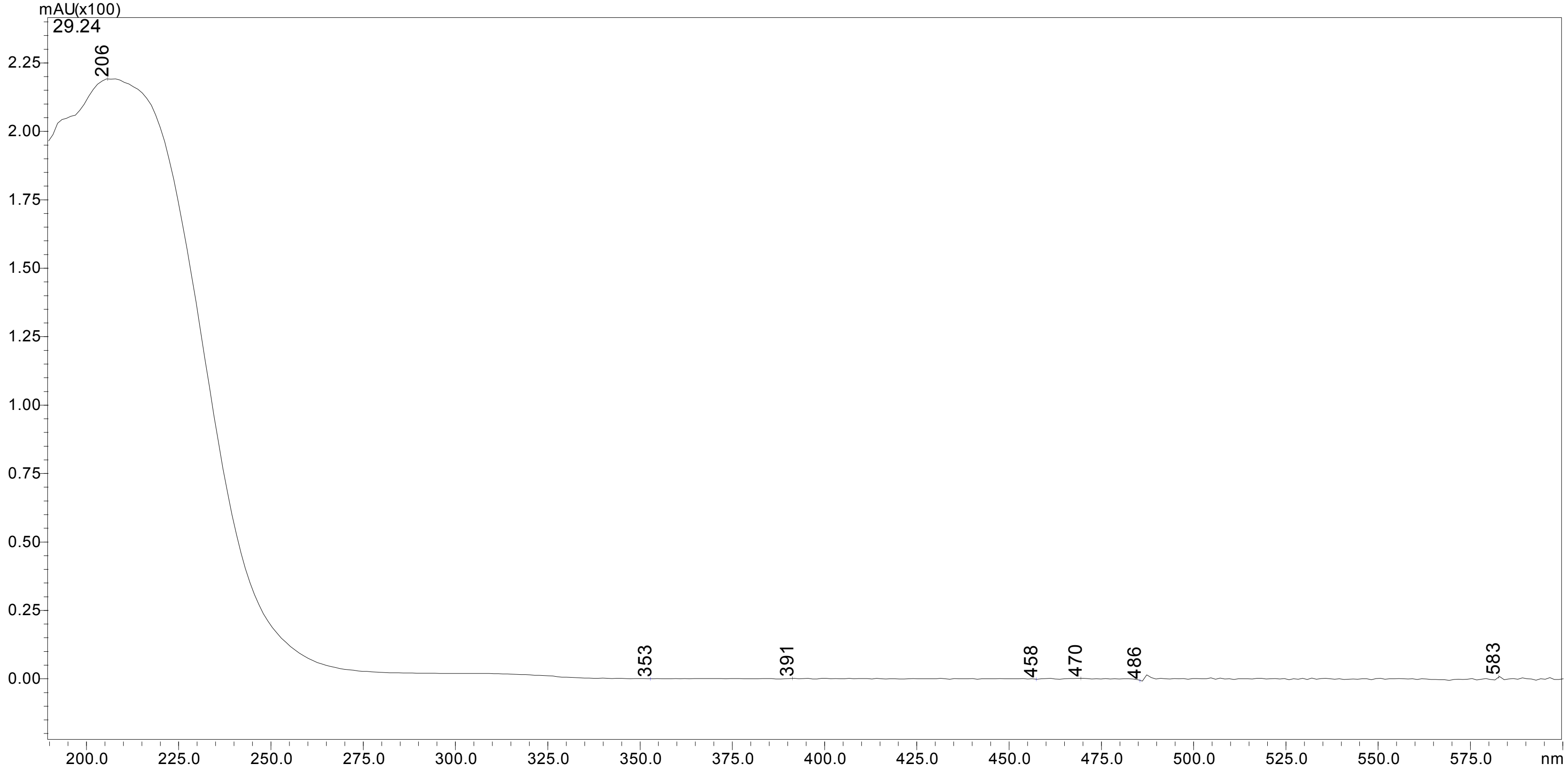

Figure S25. ^{1}H- NMR spectrum of 2 in DMSO-d_6

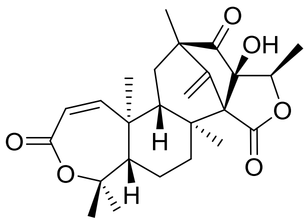

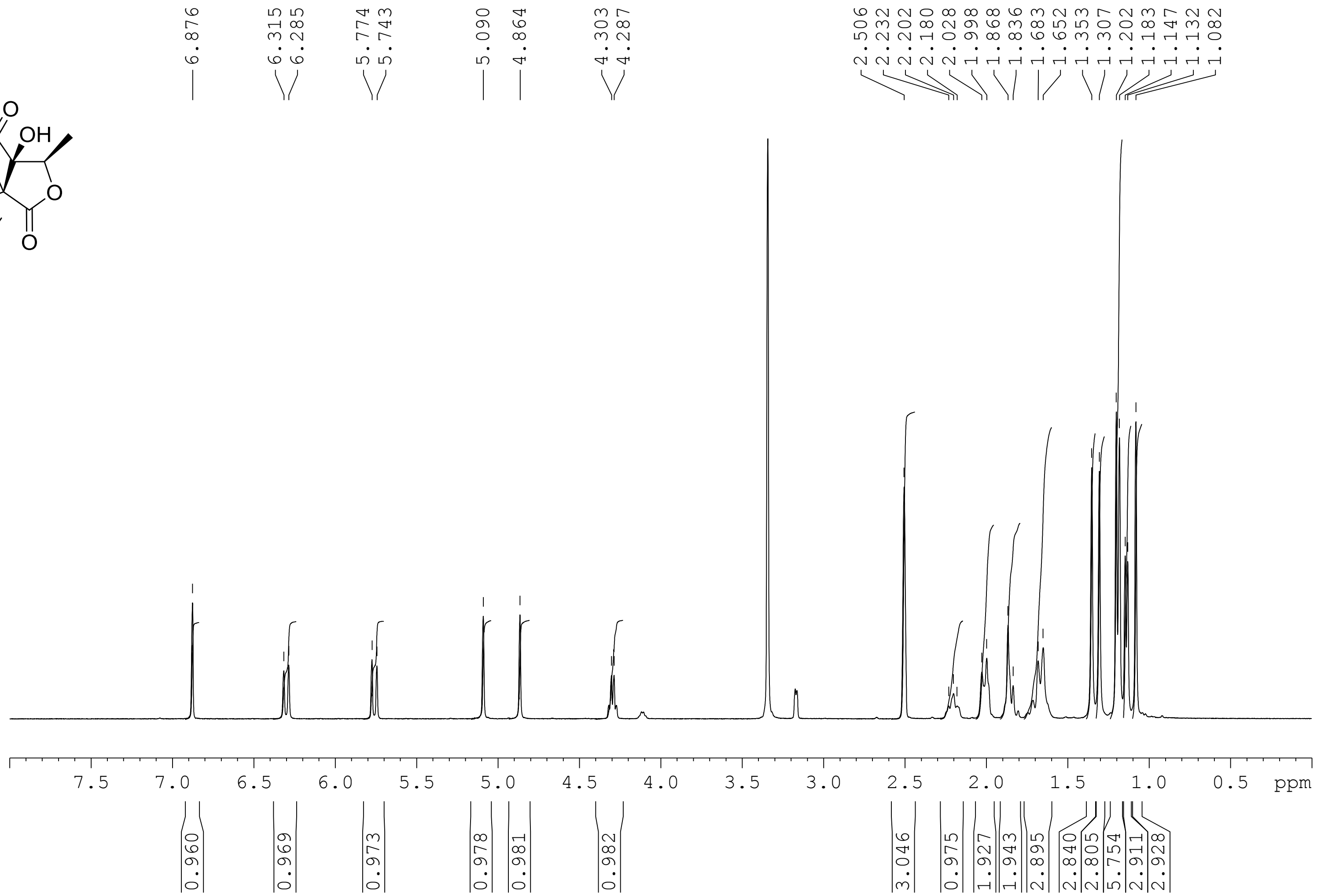

Figure S26. ^{13}C NMR spectrum of 2 in DMSO-d_6

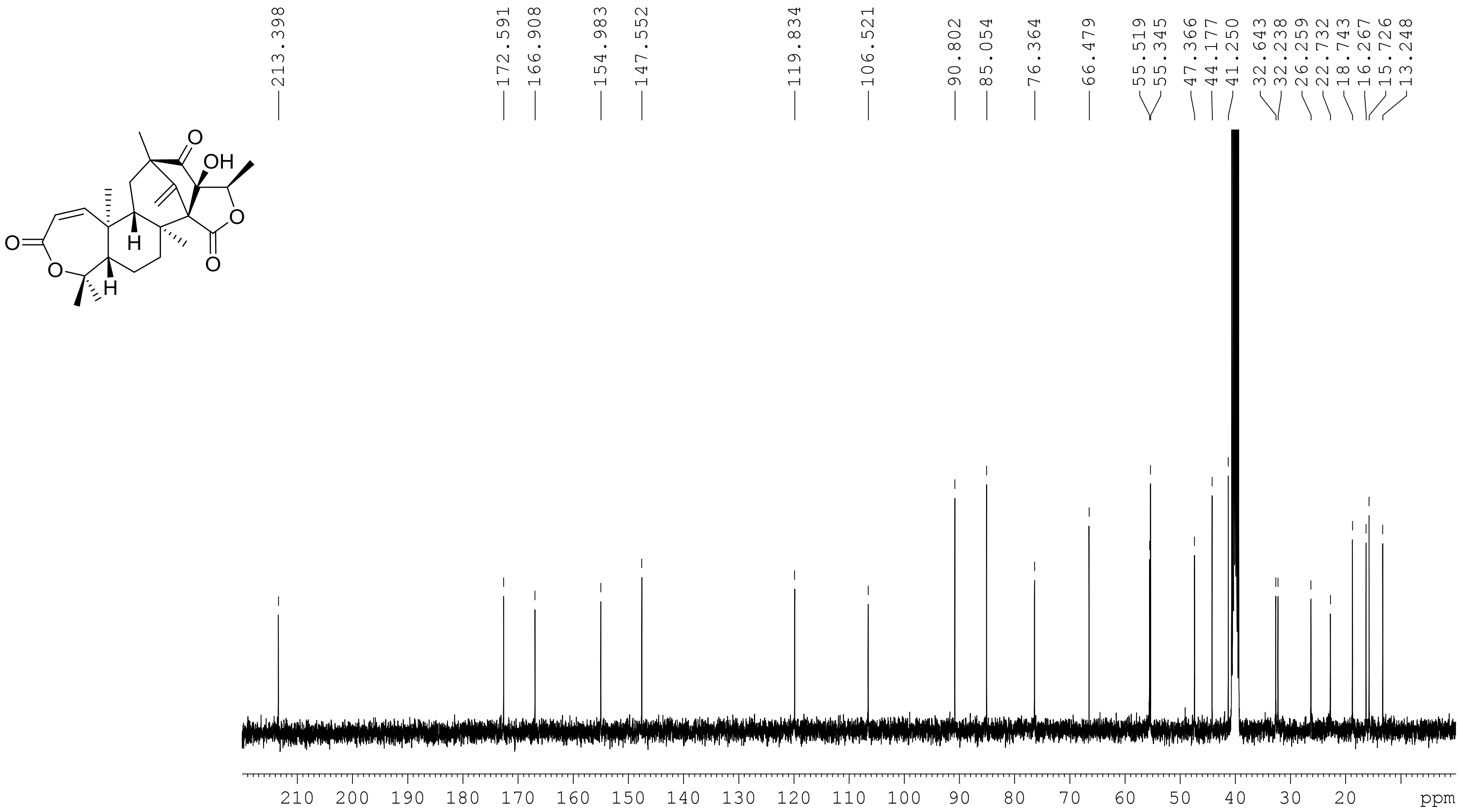

Figure S27. DEPT-135 spectrum of 2 in DMSO-d_6

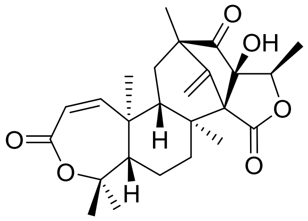

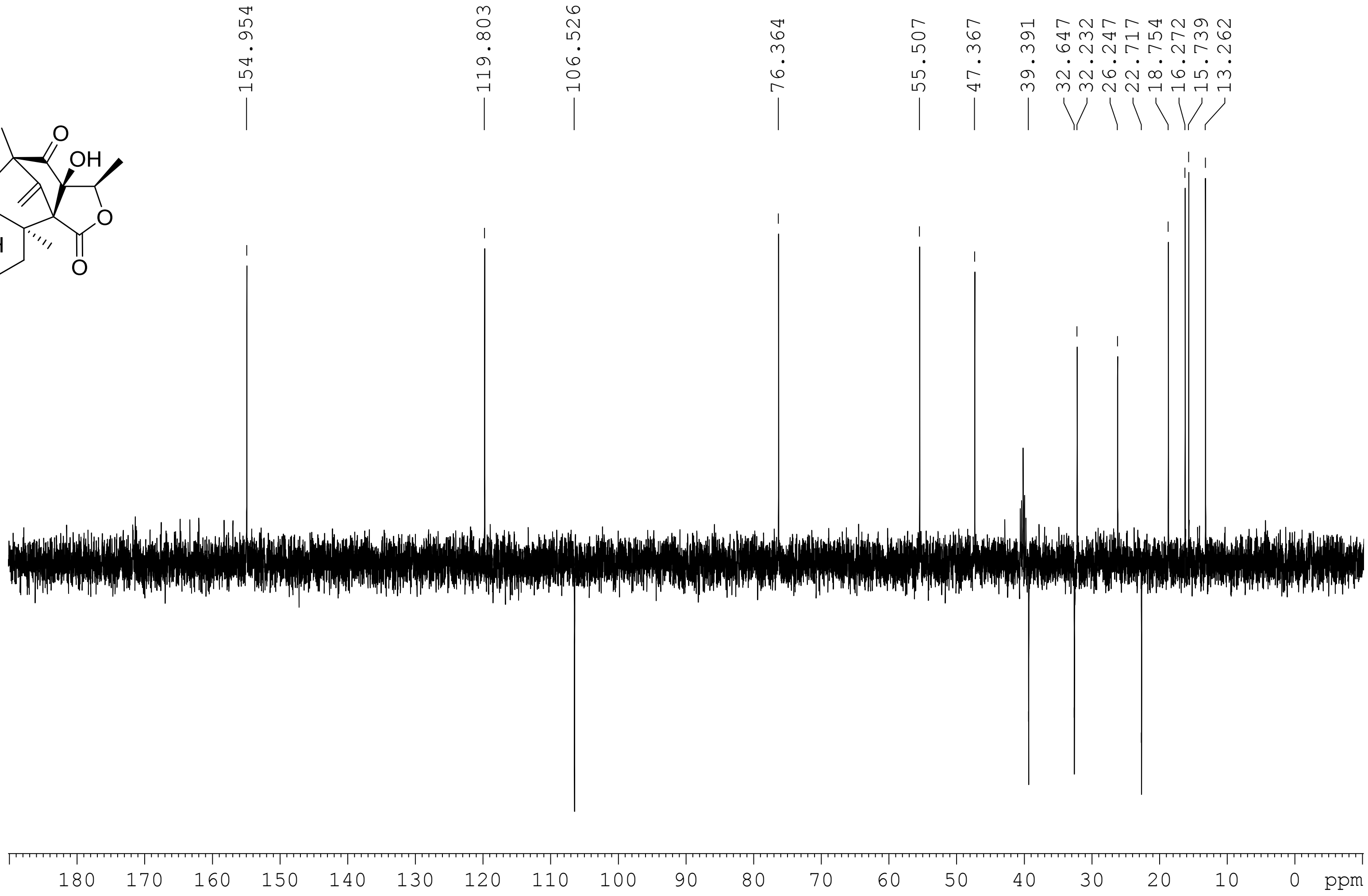

Figure S28. ^{1}H-^{1}H COSY spectrum of 2 in DMSO-d_6

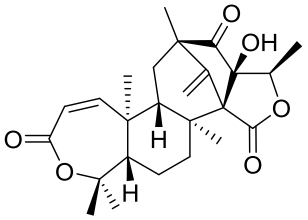

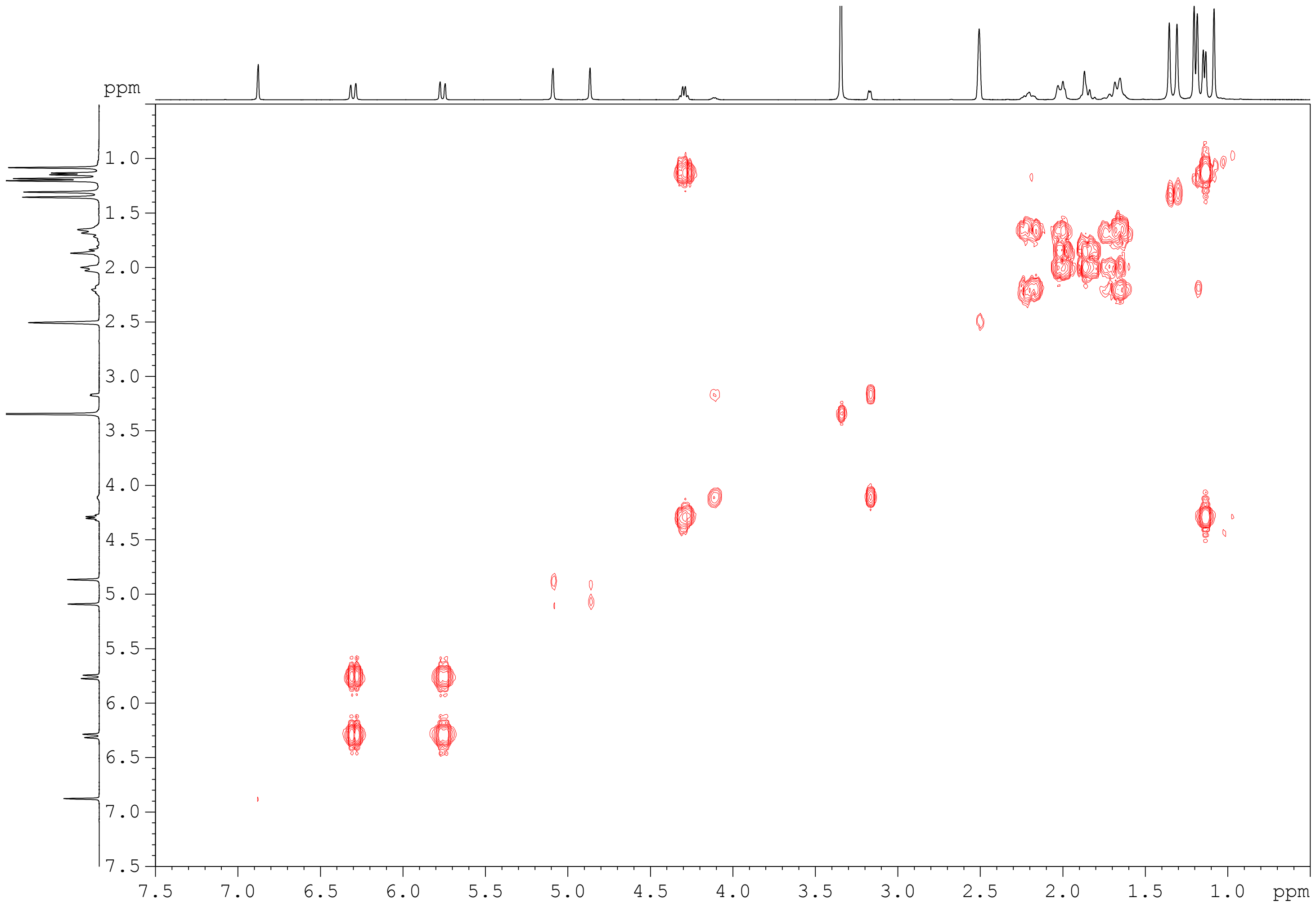

Figure S29. HSQC spectrum of 2 in DMSO-d_6

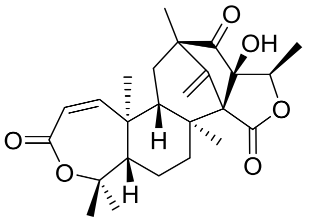

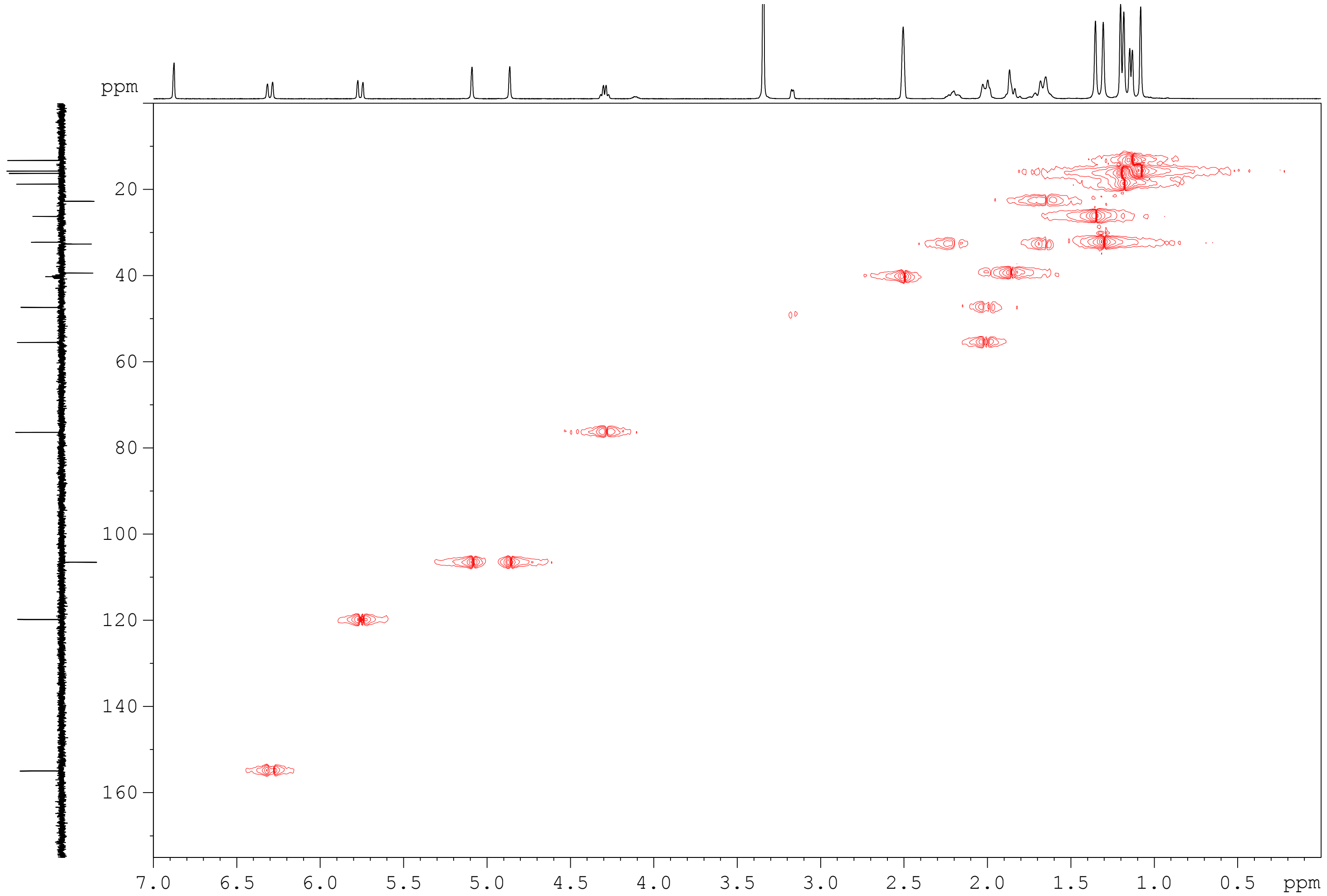

Figure S30. HMBC spectrum of 2 in DMSO-d_6

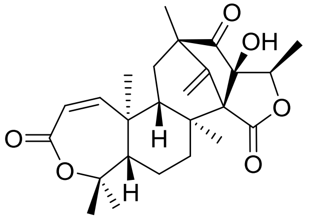

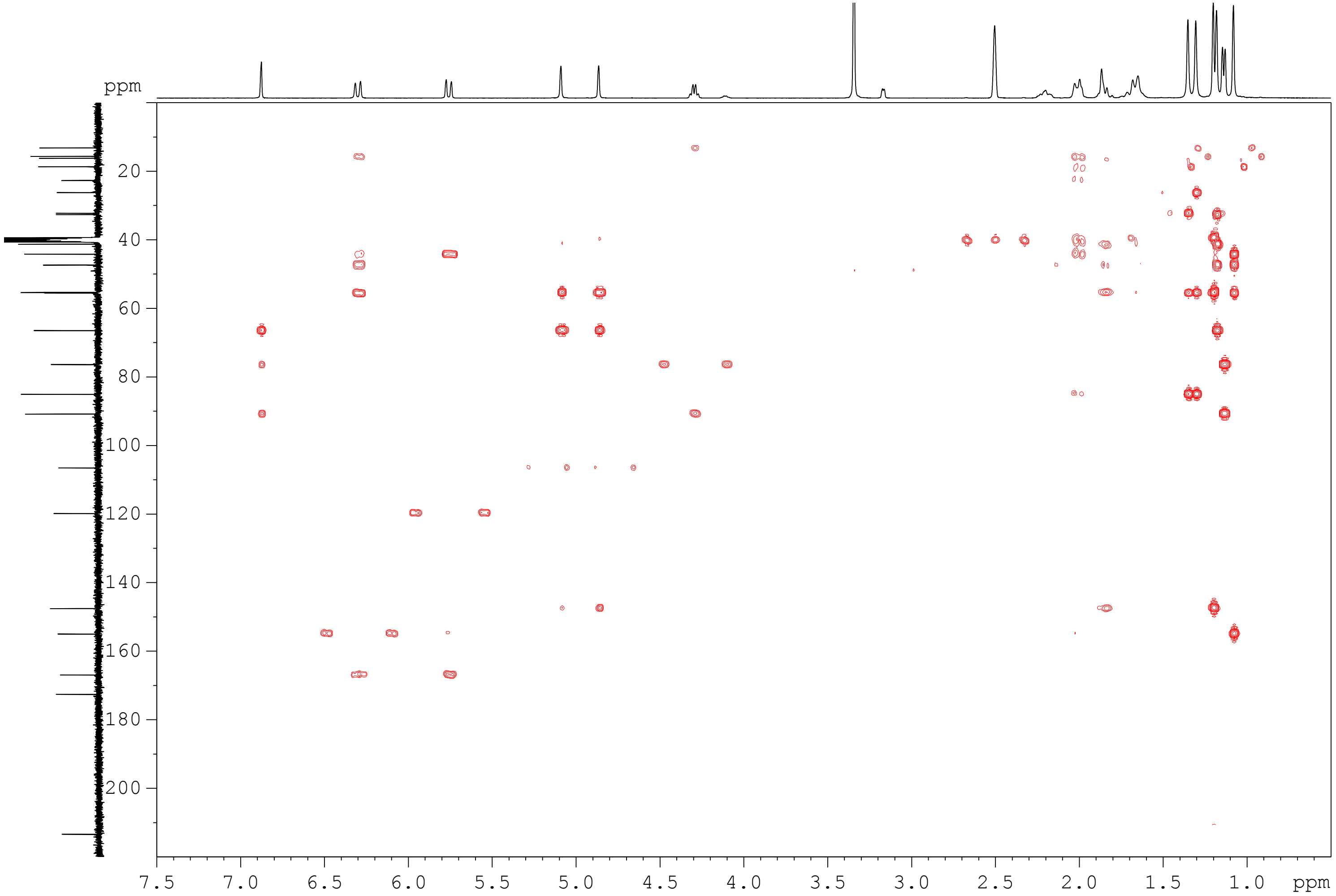

Figure S31. NOESY spectrum of 2 in DMSO-d_6

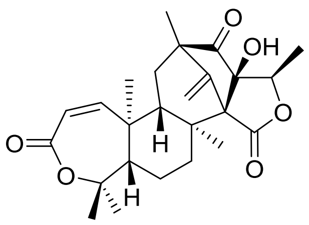

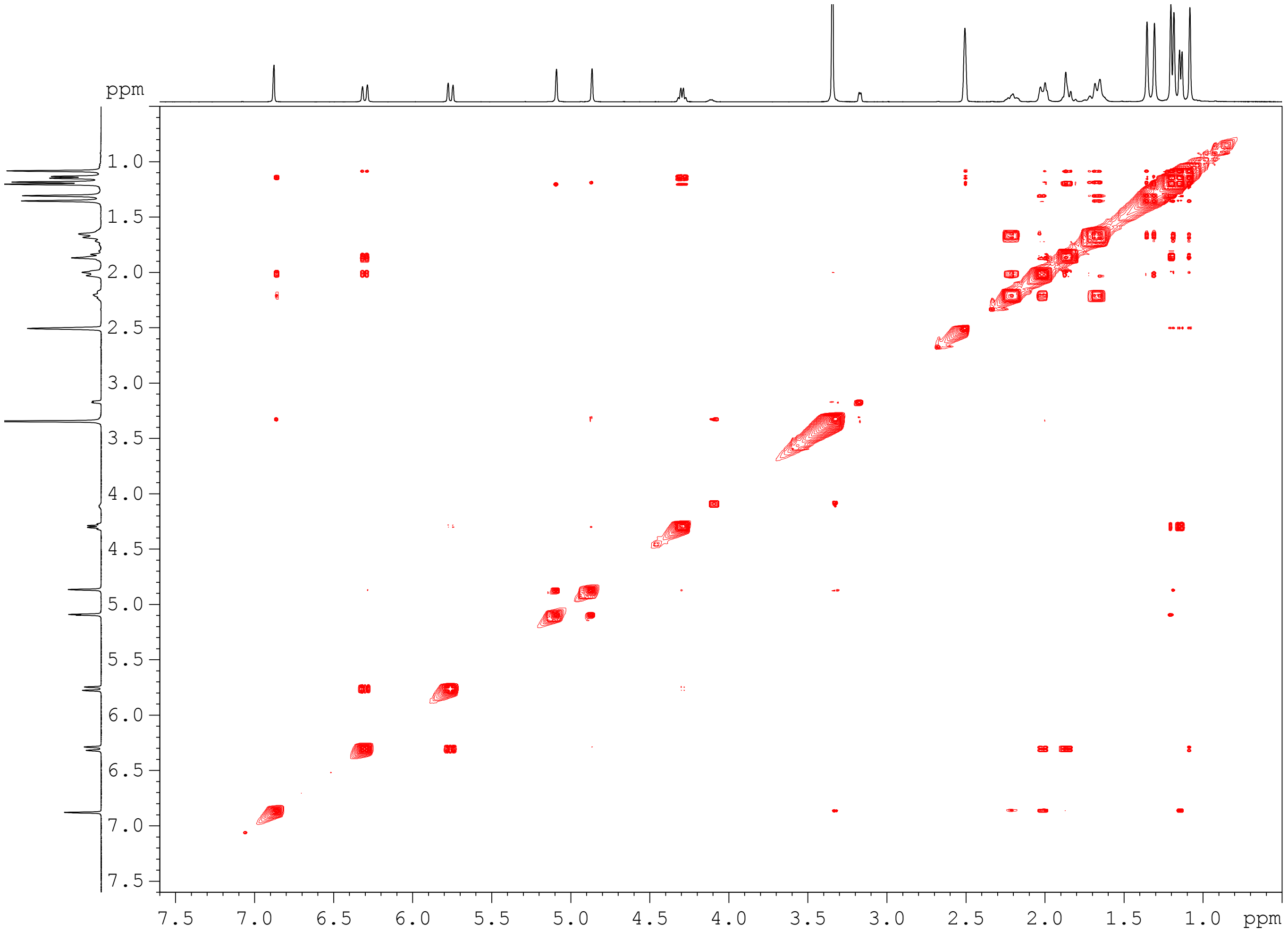

X-ray crystallographic data of 2

Empirical formula	$C_{25}H_{32}O_6$
Formula weight	428.50
Temperature	105.0 K
Wavelength	1.54178 Å
Crystal system	monoclinic
Space group	$P2_1$
Unit cell dimensions	
	a = 8.1092(3) Å α = 90°.
	b = 9.1689(3) Å β = 103.296(3)°.
	c = 15.1336(5) Å γ = 90°.
Volume	1009.05(6) Å^3
Z	2
Density (calculated)	1.300 mg/m^3
Absorption coefficient	0.747 mm^{-1}
F(000)	460
Crystal size	0.340 × 0.300 × 0.150 mm^3
Theta range for data collection	6 o 142.422°
Index ranges	-8 ≤ h ≤ 9, -11 ≤ k ≤ 11, -17 ≤ l ≤ 18
Reflections collected	7410
Independent reflections	4052 [R(int) = 0.0260]
Absorption correction	Semi-empirical from equivalents
Refinement method	Full-matrix least-squares on F^2
Data / restraints / parameters	4052 /1/ 287
Goodness-of-fit on F^2	1.042
Final R indices [I>2sigma(I)]	R1 = 0.0389, wR2 = 0.1016
R indices (all data)	R1 = 0.0397, wR2 = 0.1030
Absolute structure parameter	-0.04(13)
Largest diff. peak and hole	0.206 /-0.324 e.Å^{-3}

Figure S32. **X-ray structure of 2**

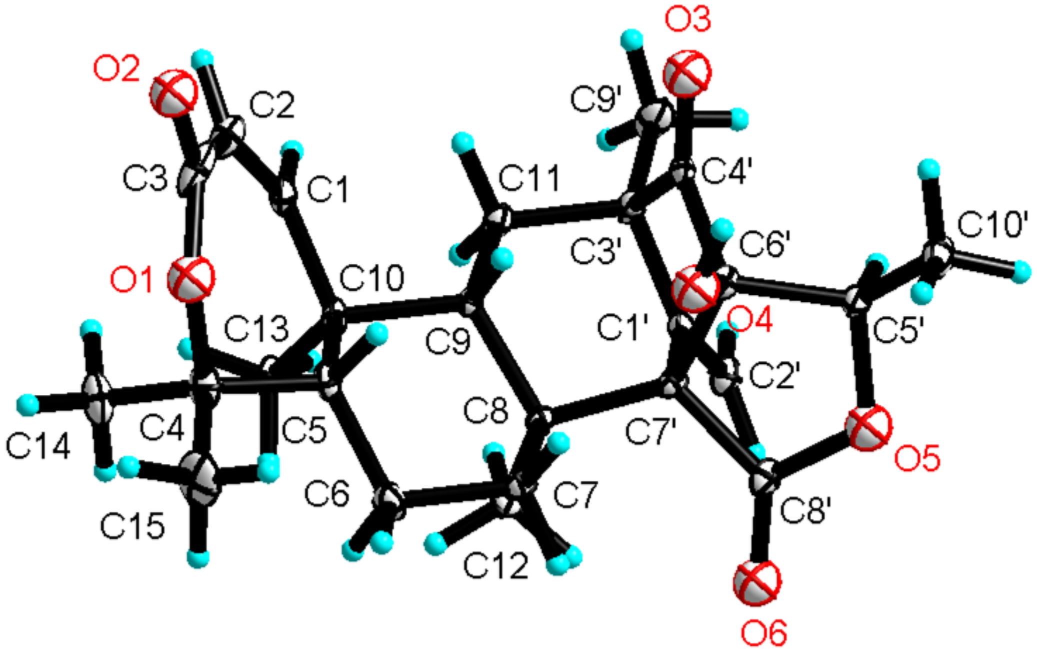

Figure S33. **HR-ESIMS spectrum of 3**

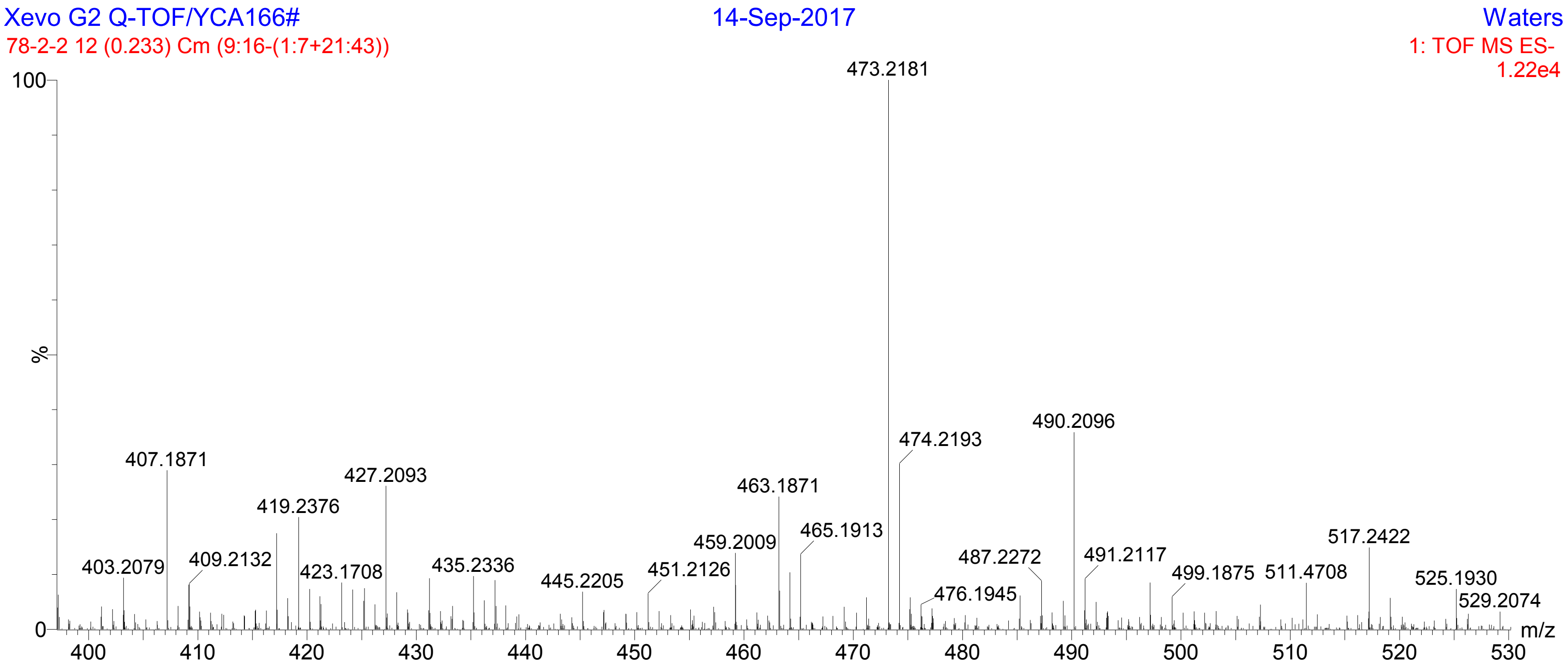

Mass	Calc. Mass	mDa	PPM	DBE	i-FIT	Norm	Conf(%)	Formula
473.2181	473.2175	0.6	1.3	10.5	185.4	n/a	n/a	$C_{26}H_{33}O_{8}$

Figure S34. IR spectrum of 3

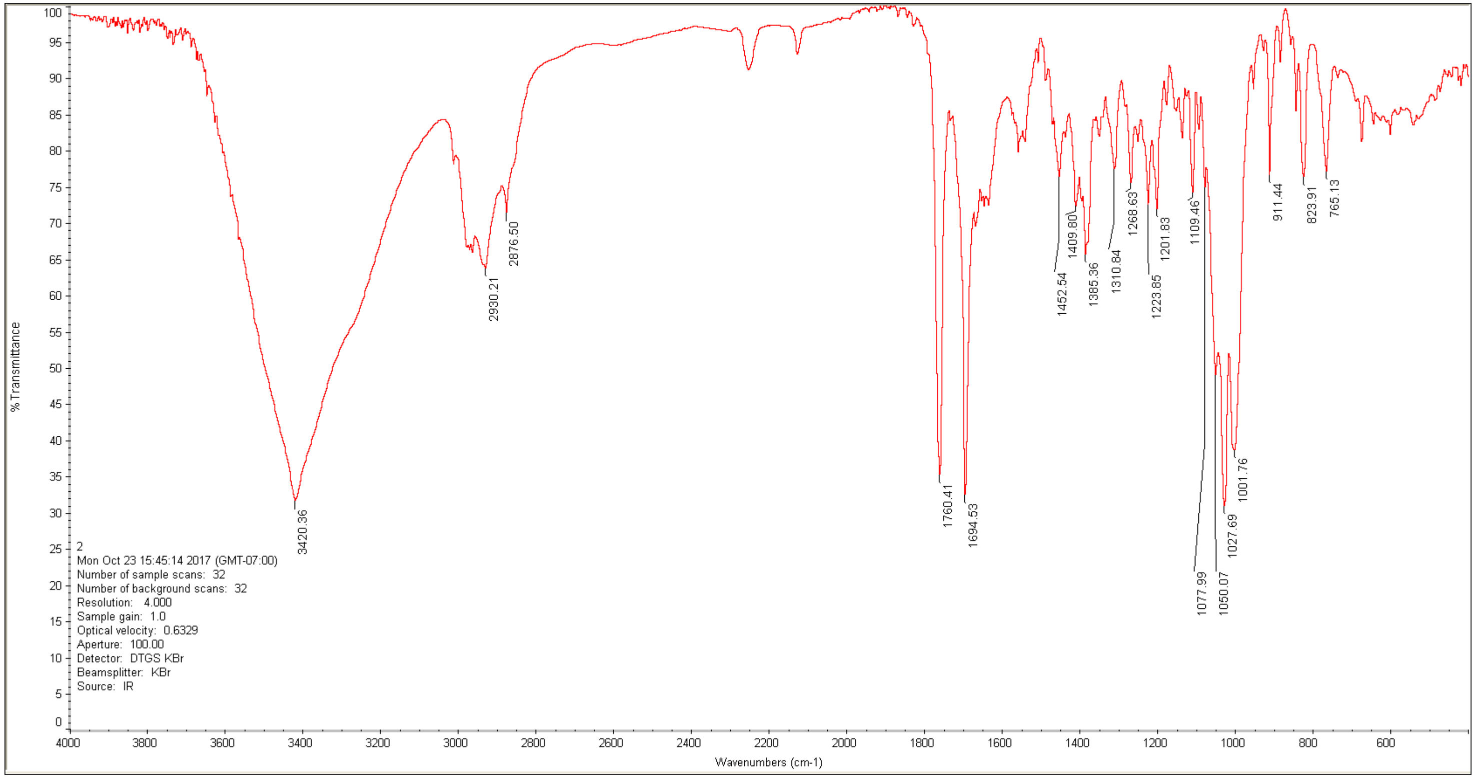

Figure S35. UV spectrum of 3 in CH_3OH

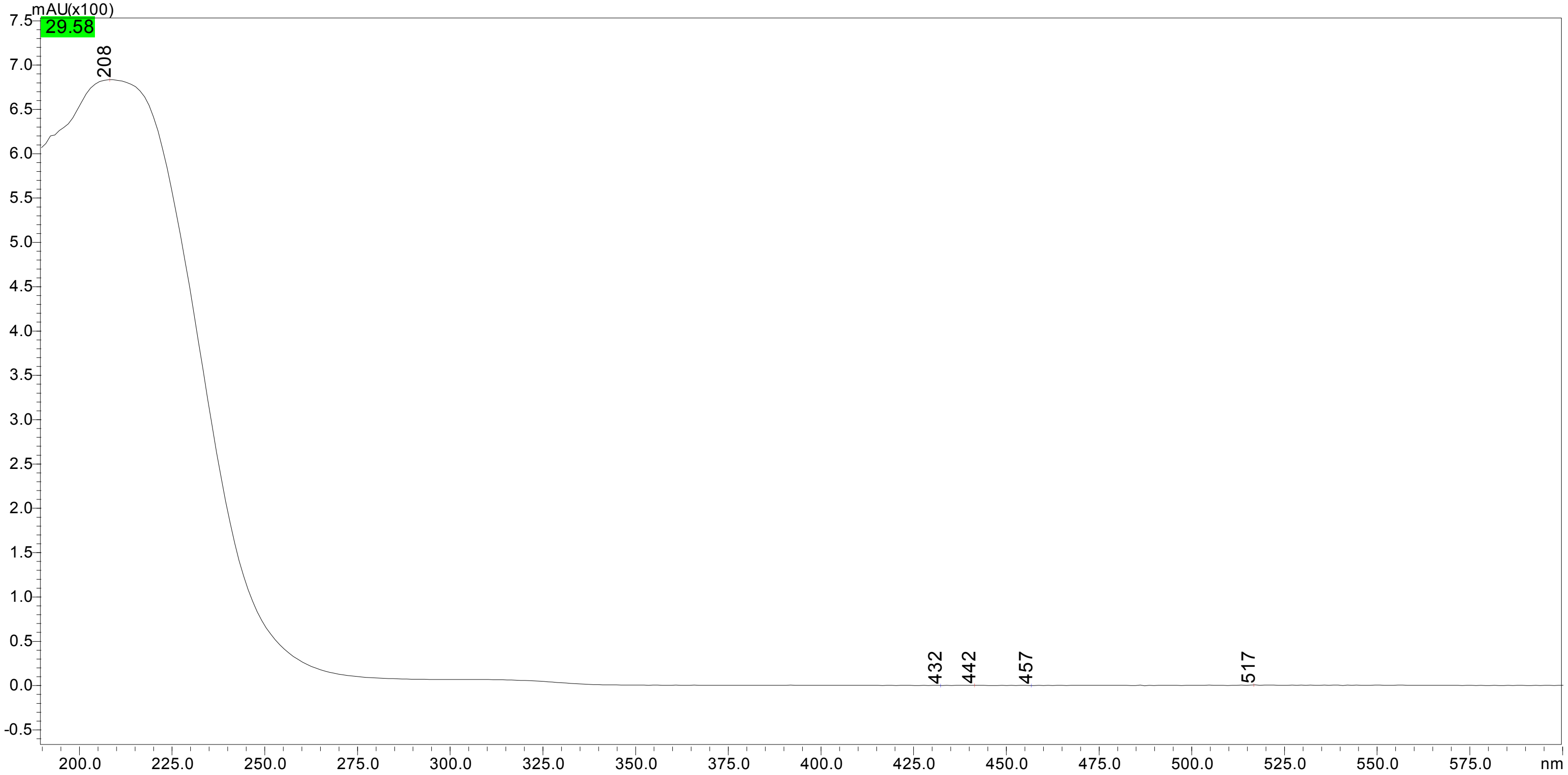

Figure S36. ^{1}H NMR spectrum of 3 in DMSO-d_6

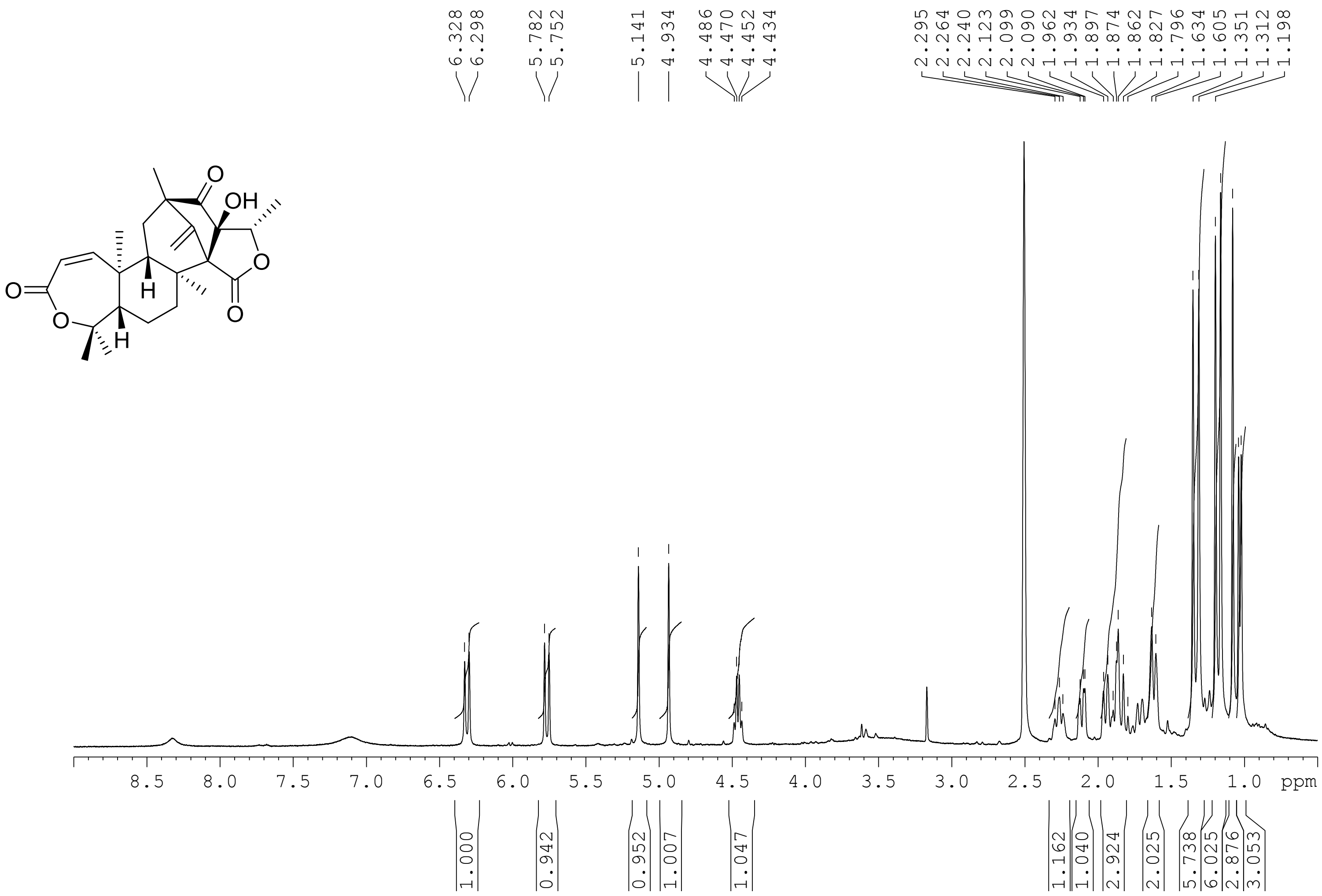

Figure S37. ^{13}C NMR spectra of 3 in DMSO-d_6

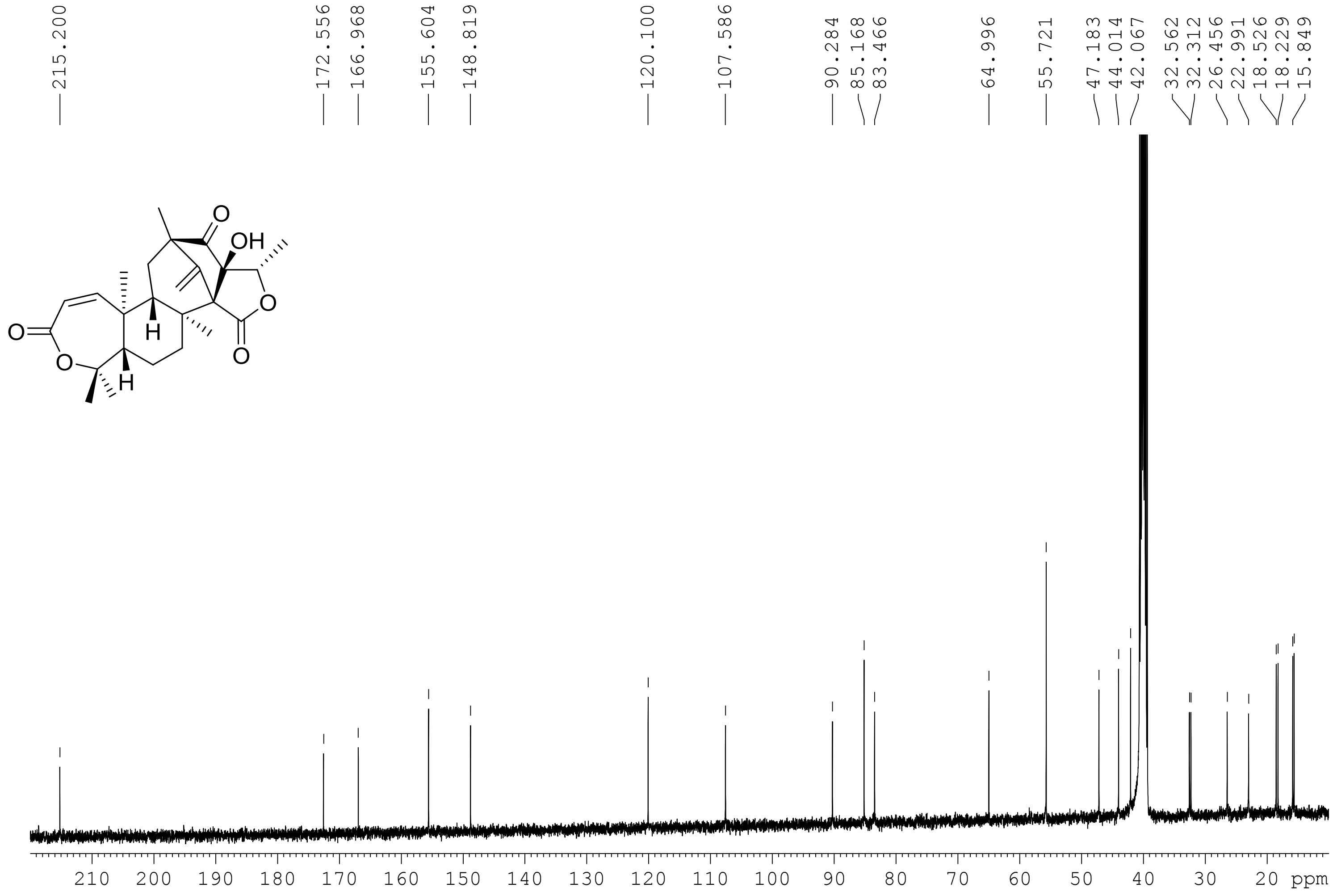

Figure S38. DEPT spectra of 3 in DMSO-d_6

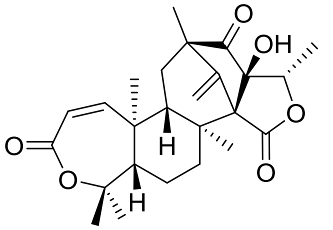

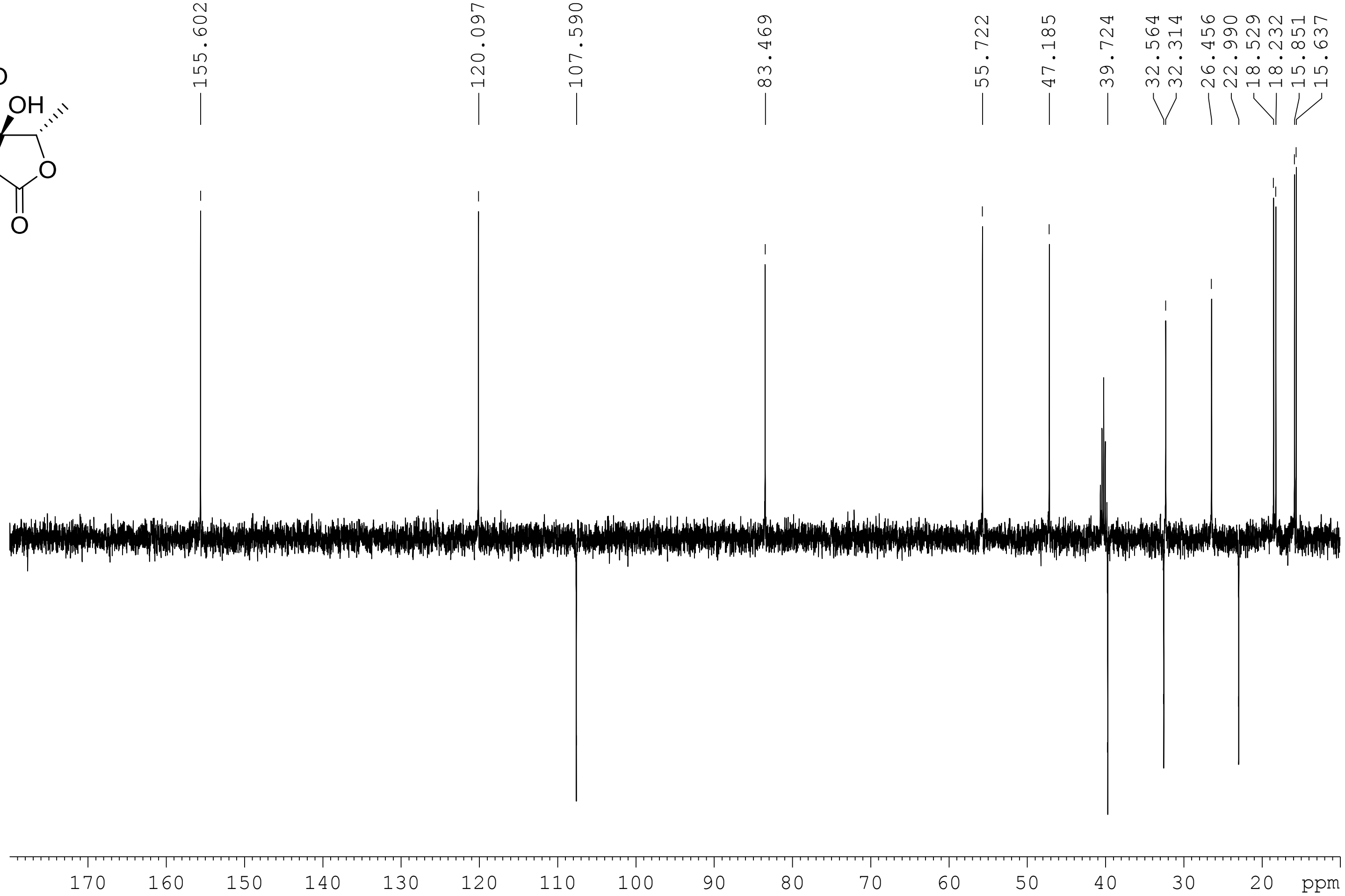

Figure S39. ^{1}**H-**^{1}**H COSY spectrum of 3 in DMSO-**d_6

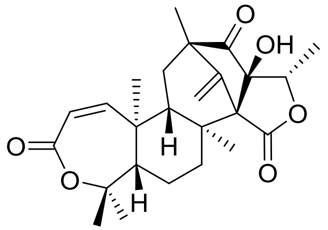

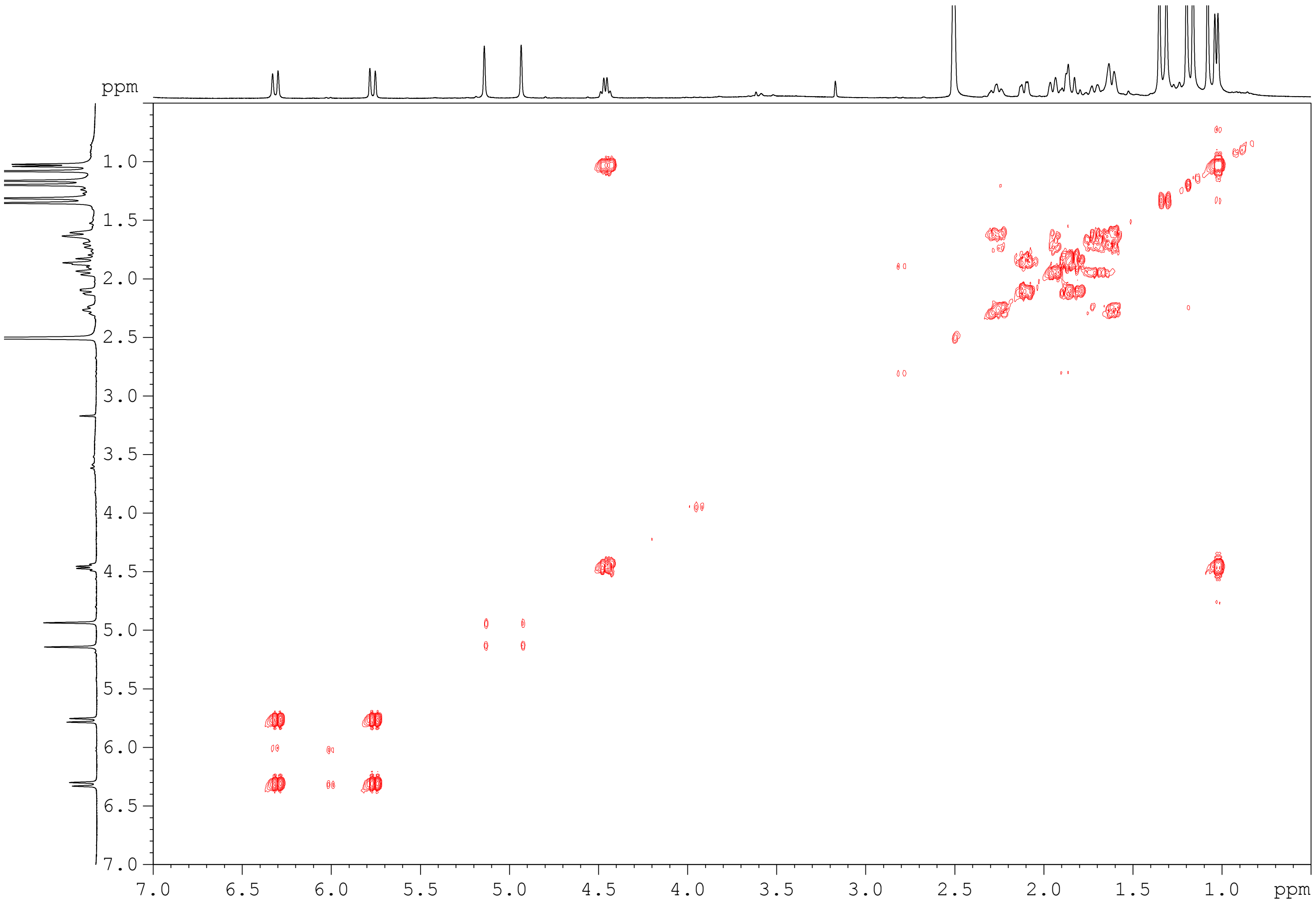

Figure S40. HSQC spectrum of 3 in DMSO-d_6

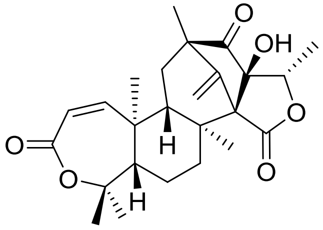

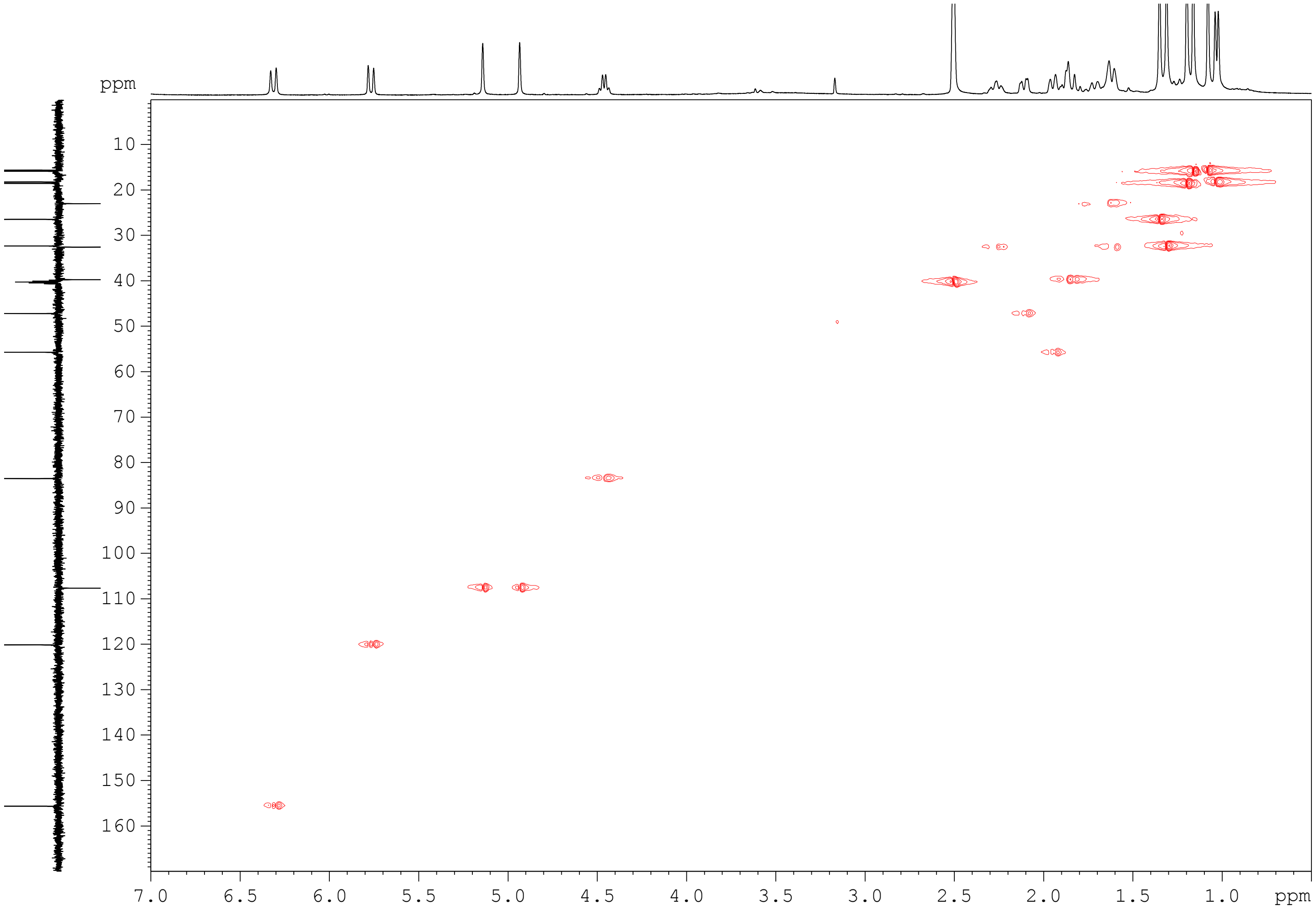

Figure S41. HMBC spectrum of 3 in DMSO-d_6

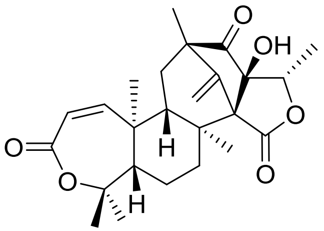

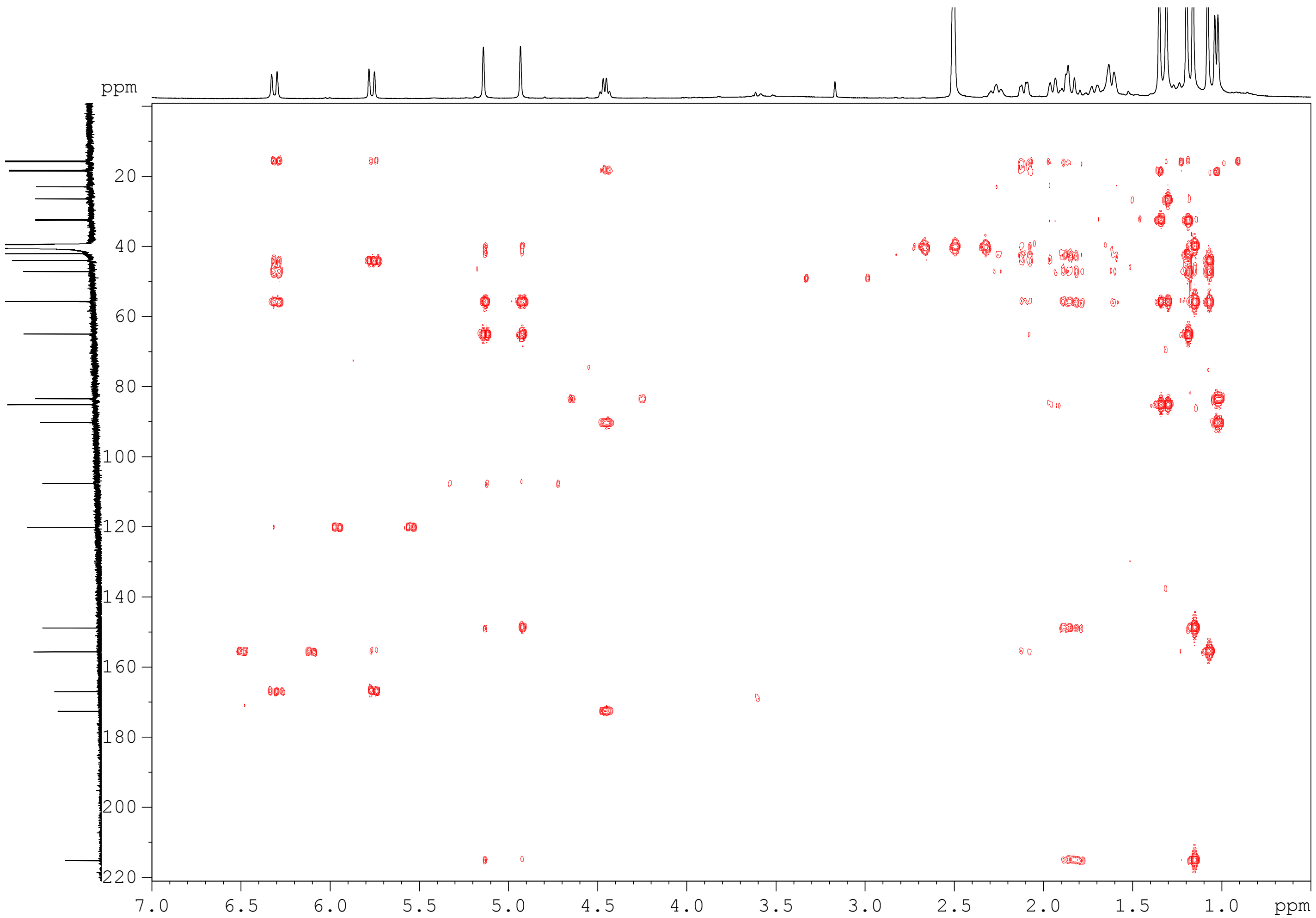

Figure S42. NOESY spectrum of 3 in DMSO-d_6

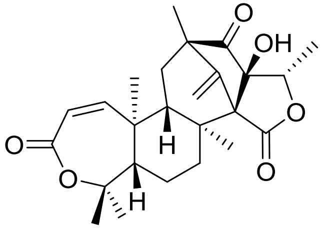

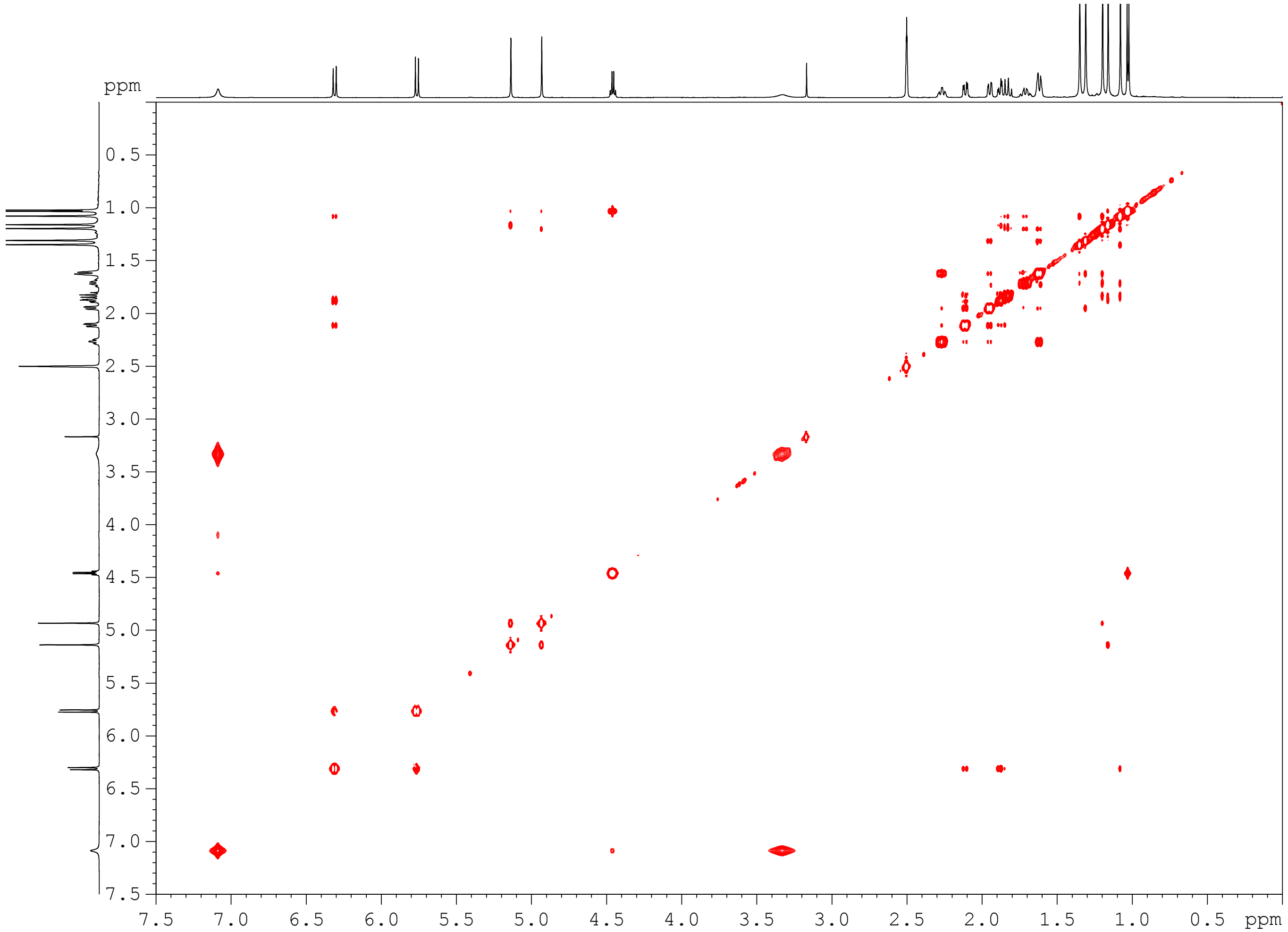

X-ray crystallographic data of 3

Empirical formula	$C_{25}H_{32}O_6$	
Formula weight	428.51	
Temperature	105.6 K	
Wavelength	1.54178 Å	
Crystal system	monoclinic	
Space group	$P2_1$	
Unit cell dimensions	a = 8.0821(3) Å	α = 90°.
	b = 9.4702(4) Å	β = 102.874(3)°.
	c = 14.8847(6) Å	γ = 90°.
Volume	1110.62(8) $Å^3$	
Z	2	
Density (calculated)	1.281 mg/m^3	
Absorption coefficient	0.737 mm^{-1}	
F(000)	460	
Crystal size	0. 400 × 0.300 × 0.120 mm^3	
Theta range for data collection	11.16 o 142.34°	
Index ranges	$-8 \leq h \leq 9$, $-11 \leq k \leq 11$, $-18 \leq l \leq 18$	
Reflections collected	7335	
Independent reflections	4198 [R(int) = 0.0228]	
Absorption correction	Semi-empirical from equivalents	
Refinement method	Full-matrix least-squares on F^2	
Data / restraints / parameters	4198 /1/ 287	
Goodness-of-fit on F^2	1.032	
Final R indices [I>2sigma(I)]	R1 = 0.0356, wR2 = 0.0928	
R indices (all data)	R1 = 0.0361, wR2 = 0.0934	
Absolute structure parameter	-0.06(13)	
Largest diff. peak and hole	0.203 /-0.205 e.$Å^{-3}$	

Figure S43. **X-ray structure of 3**

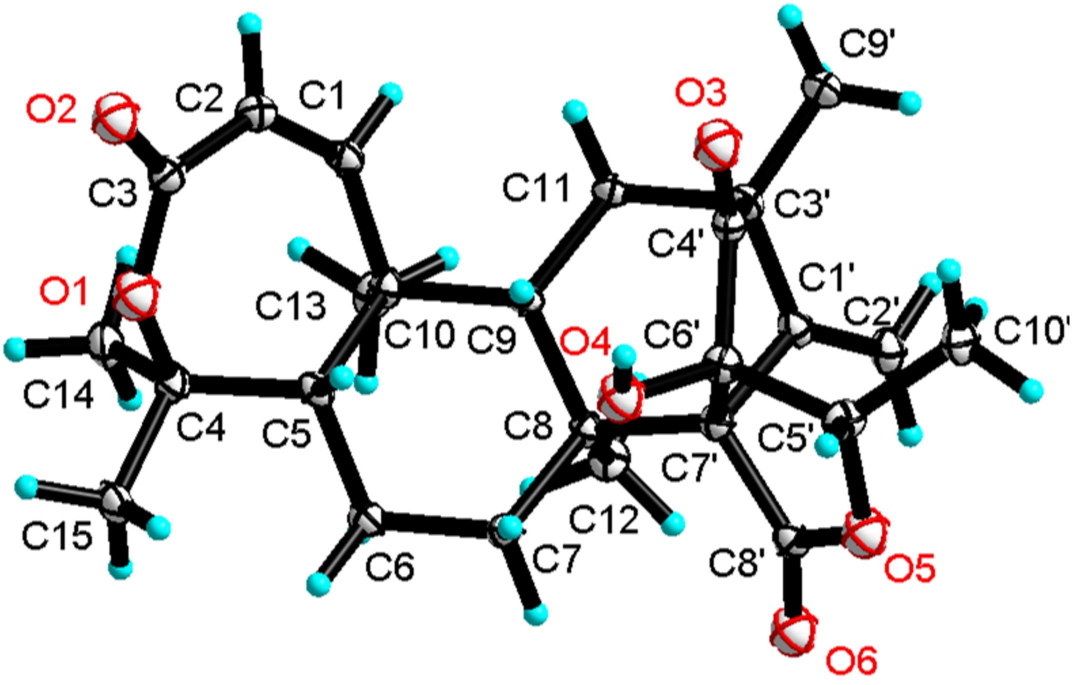

Figure S44. HRESIMS spectrum of 3

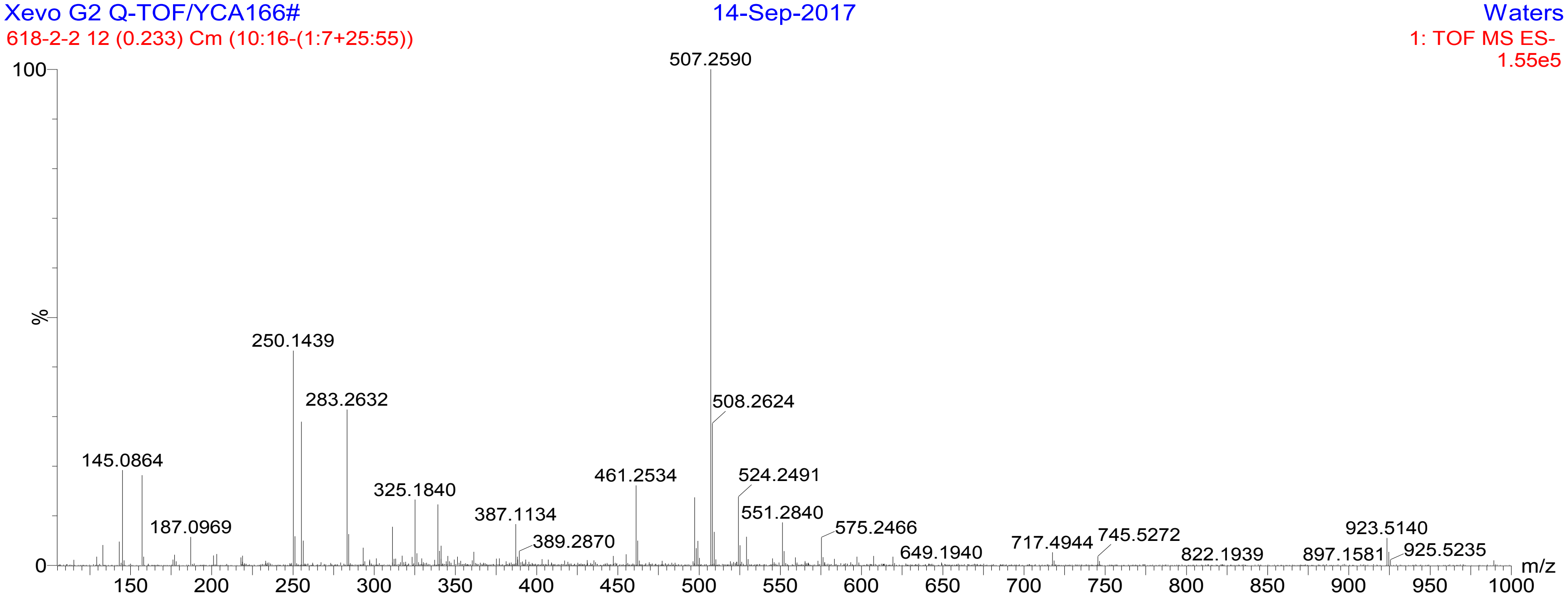

Mass	Calc. Mass	mDa	PPM	DBE	i-FIT	Norm	Conf(%)	Formula
507.2590	507.2594	-0.4	-0.8	8.5	130.2	0.000	100.00	$C_{27}H_{39}O_9$

Figure S45. IR spectrum of 4 in DMSO-d_6

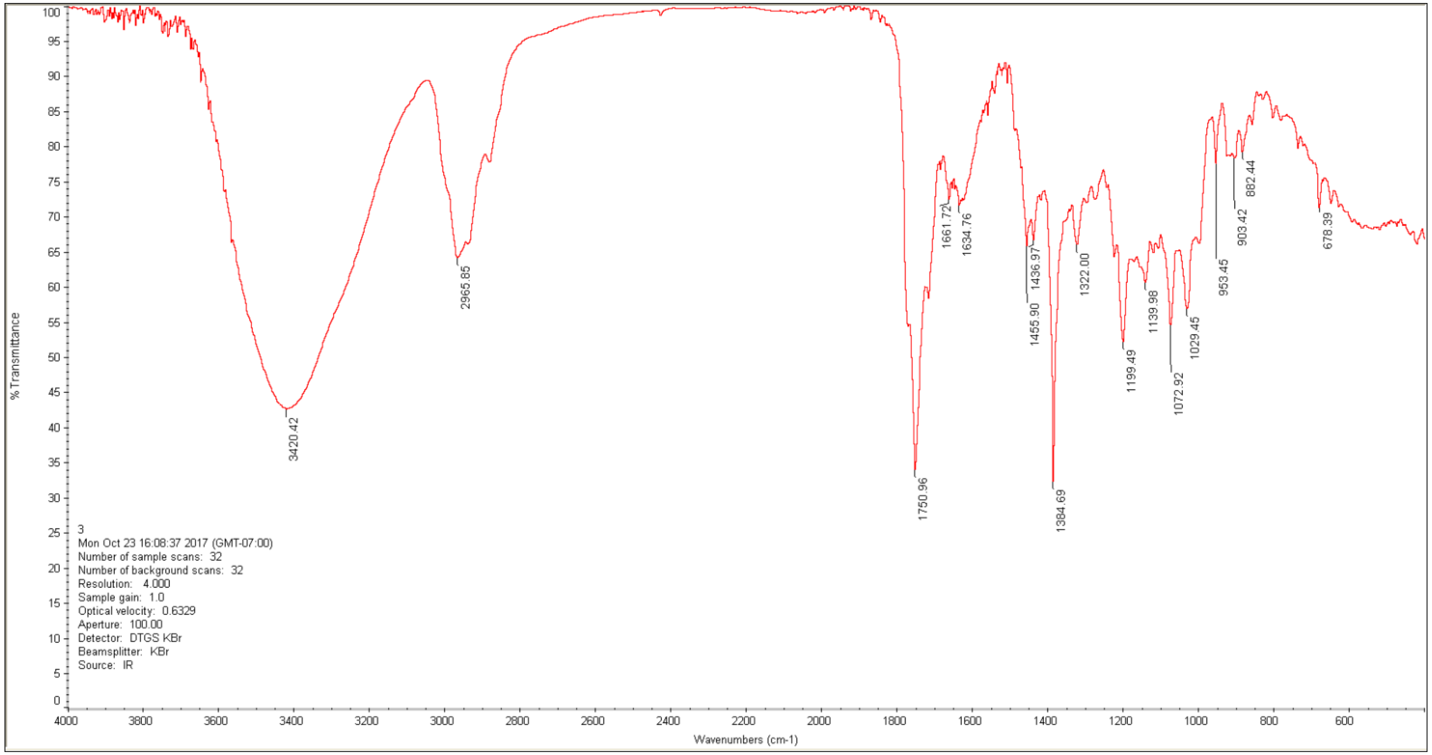

Figure S46. UV spectrum of 4 in CH_3OH

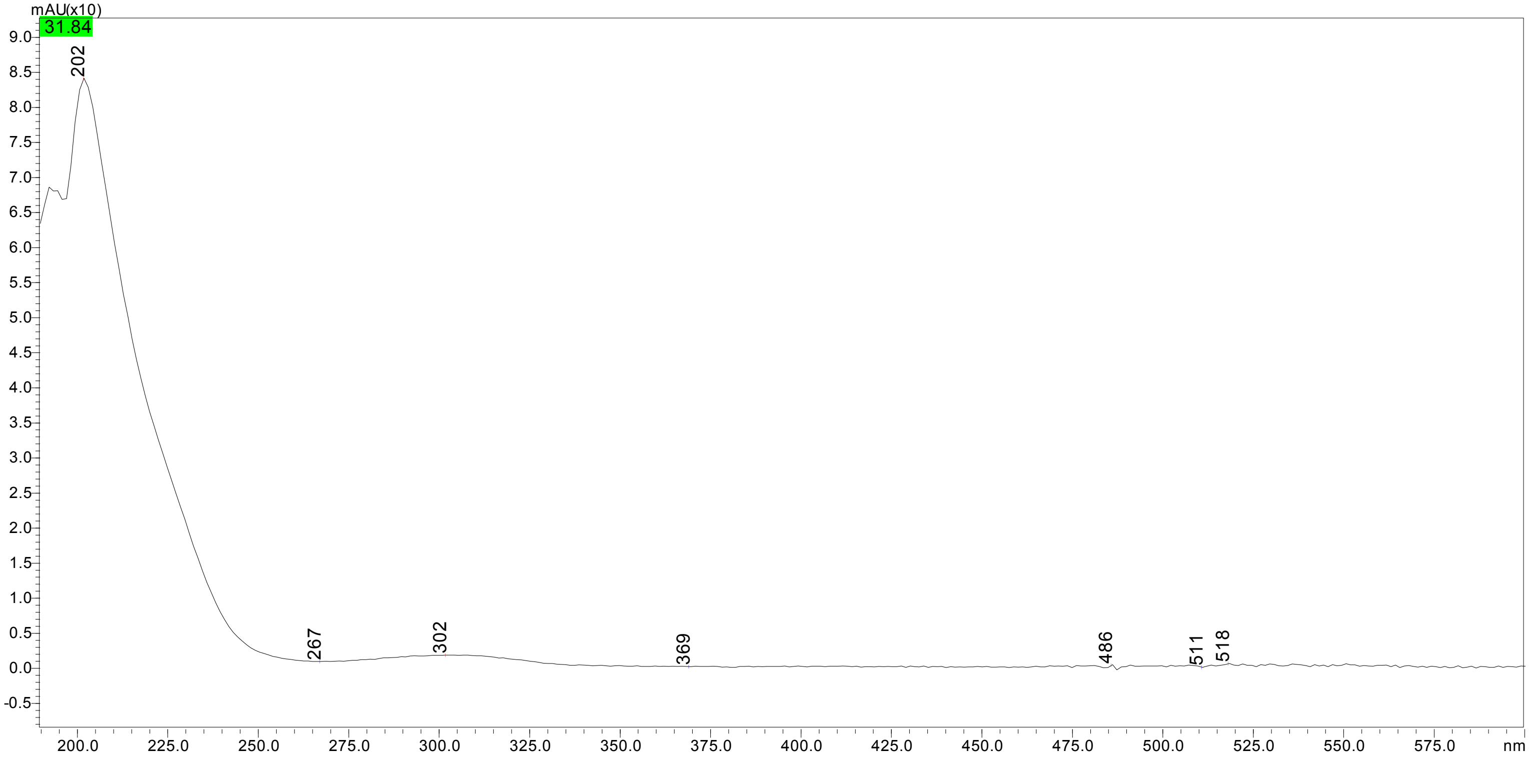

Figure S47. ^{1}H NMR spectrum of 4 in DMSO-d_6

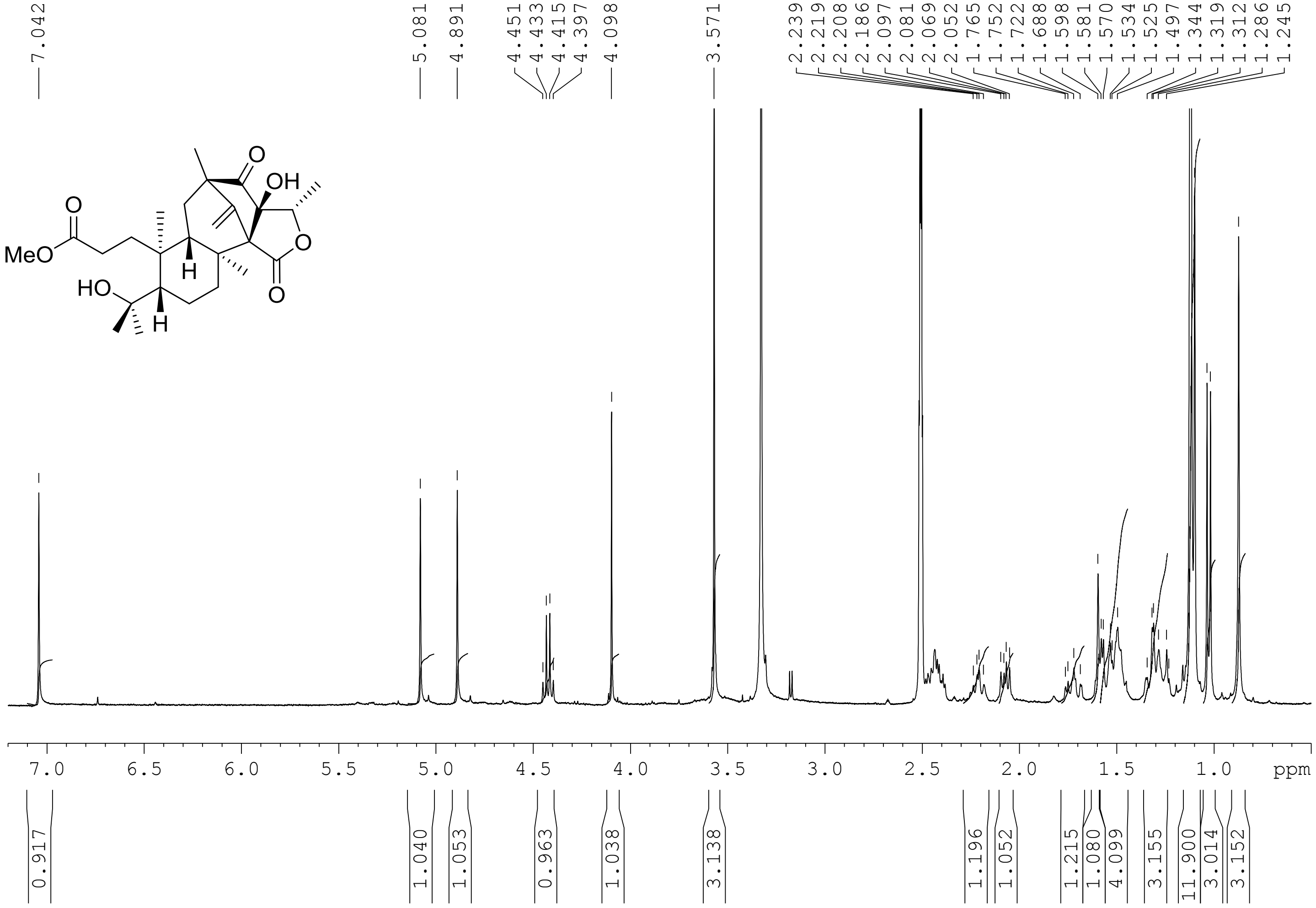

Figure S48. APT spectra of 4 in DMSO-d_6

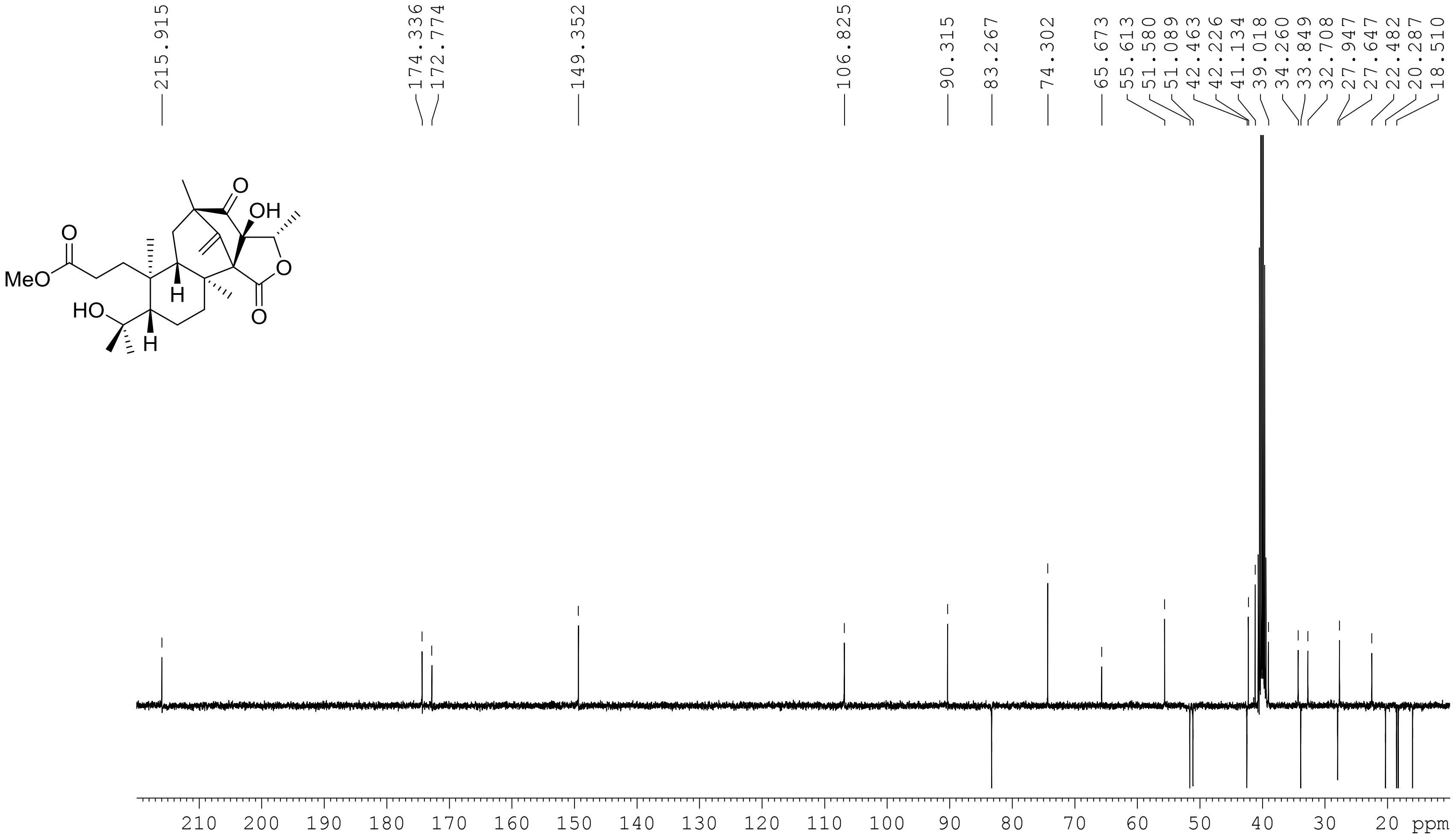

Figure S49. ^{1}H-^{1}H COSY spectrum of 4 in DMSO-d_6

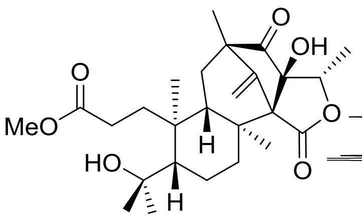

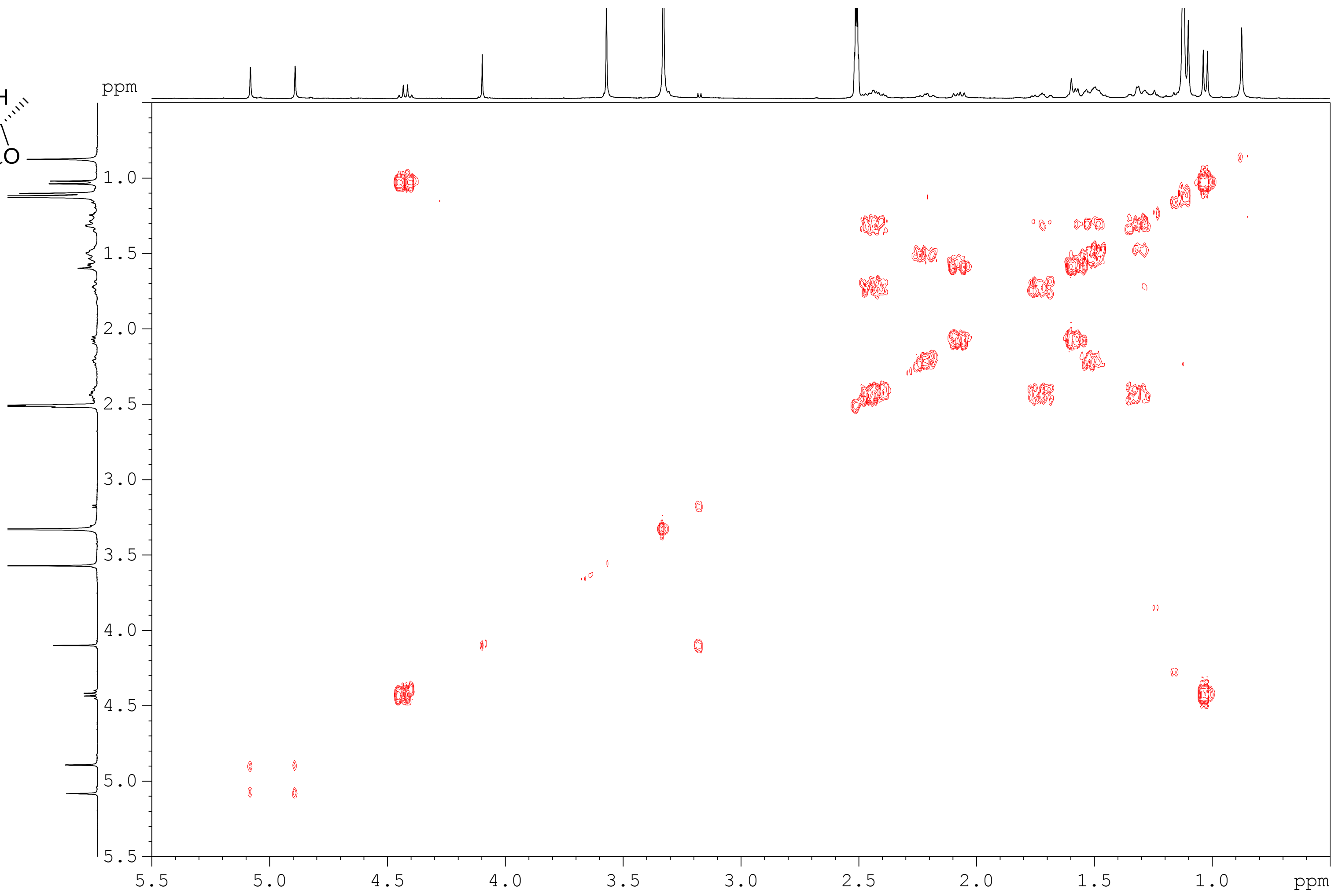

Figure S50. HSQC spectrum of 4 in DMSO-d_6

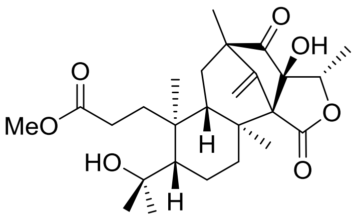

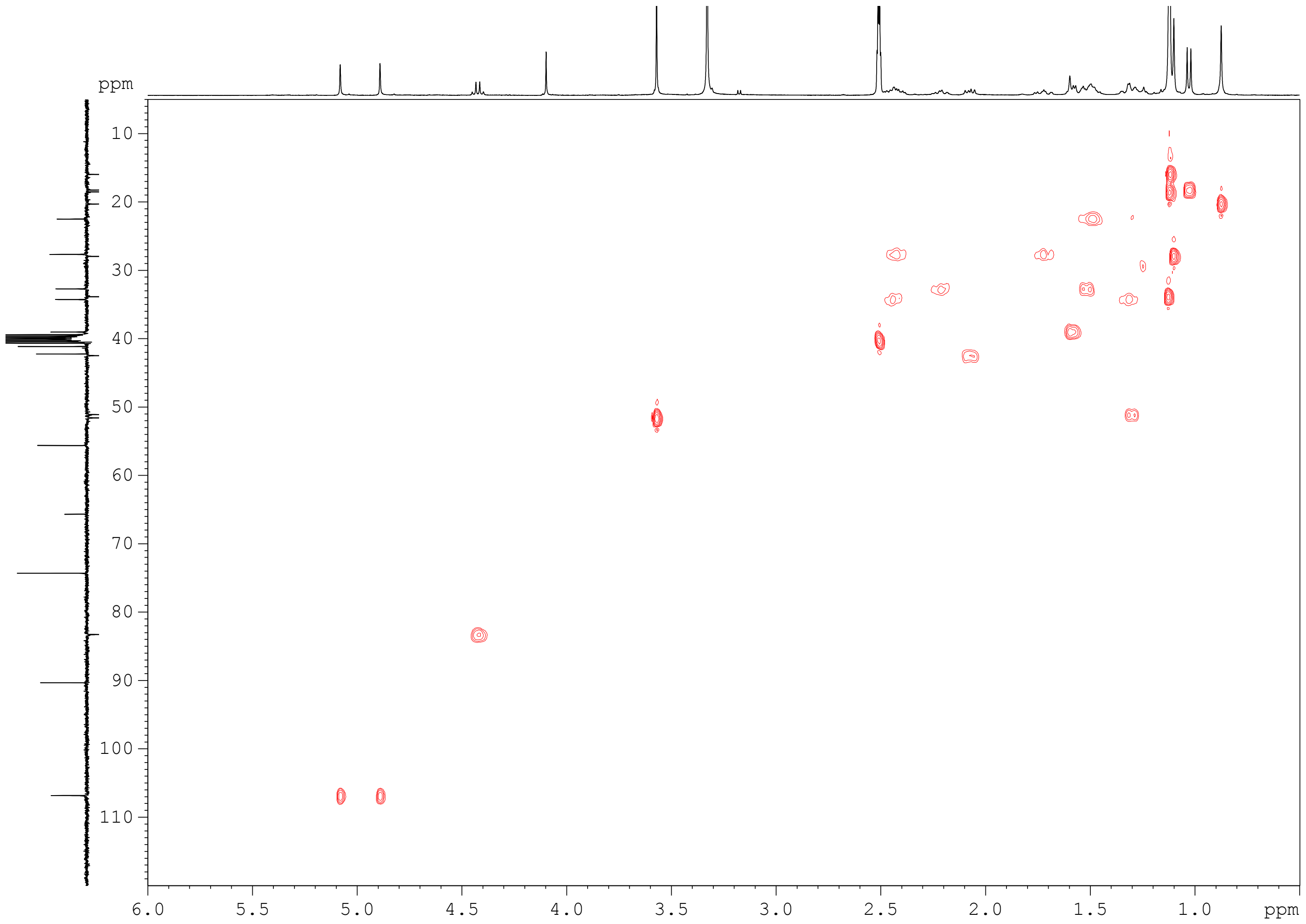

Figure S51. HMBC spectrum of 4 in DMSO-d_6

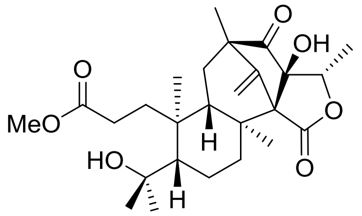

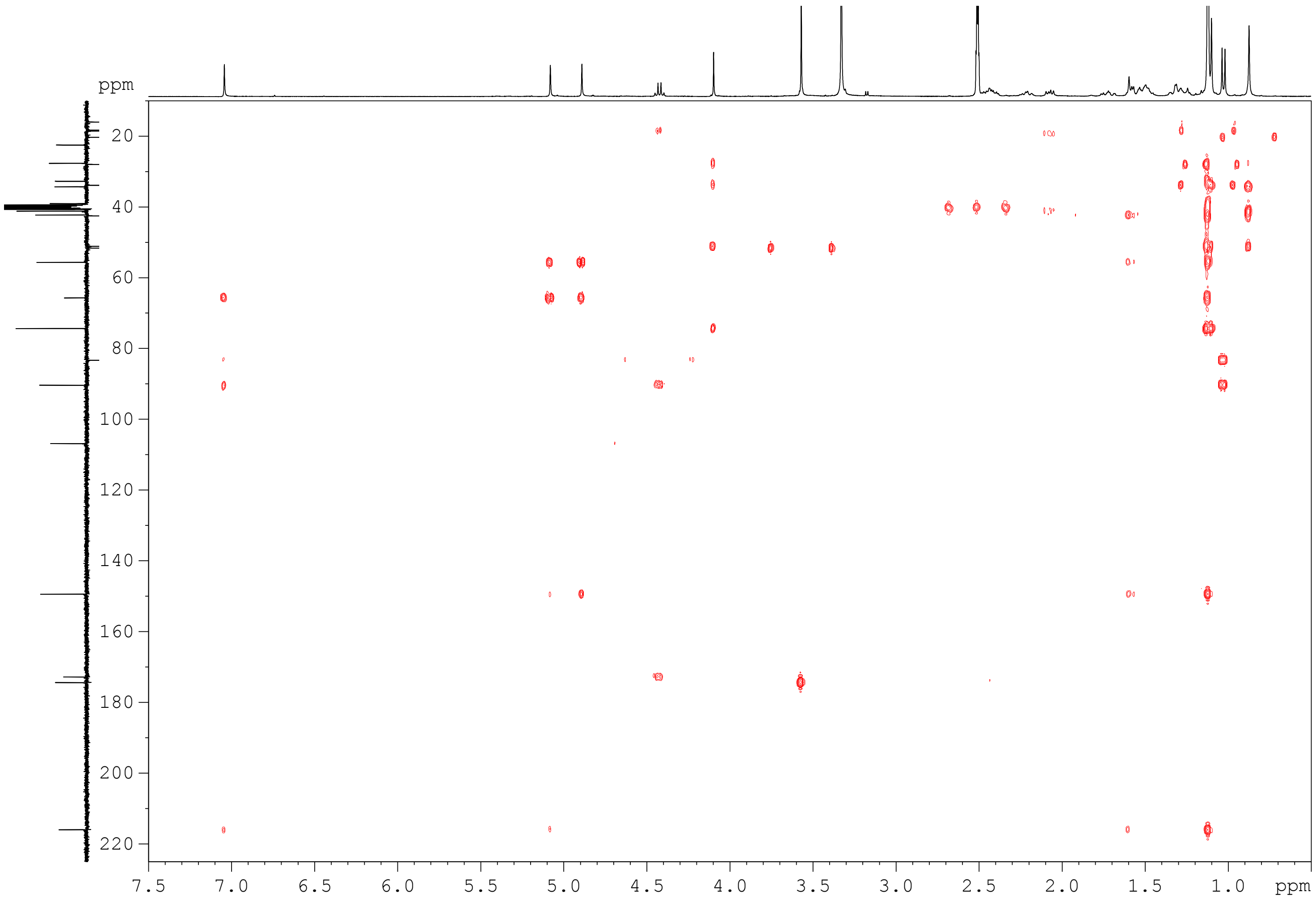

Figure S52. NOESY spectrum of 4 in DMSO-d_6

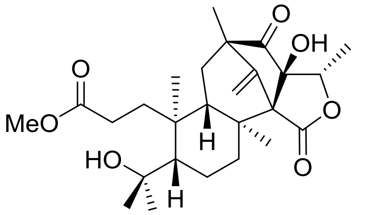

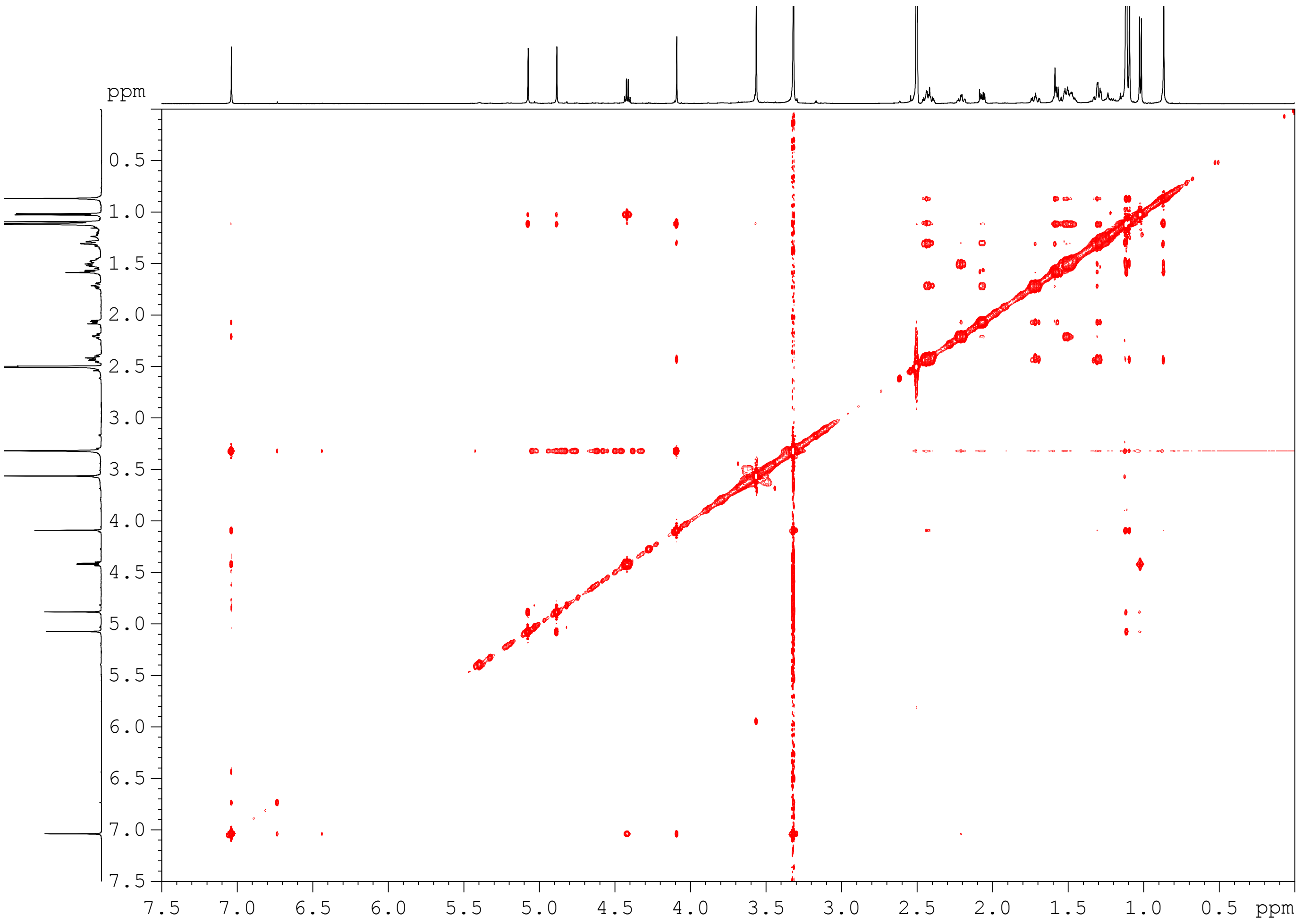

X-ray crystallographic data of 4

Empirical formula	$C_{26}H_{38}O_7$
Formula weight	462.56
Temperature	108.45(10) K
Crystal system	monoclinic
Space group	$P2_1$
Unit cell dimensions	
	a = 8.2632(8) Å α = 90°.
	b = 11.6480(8) Å β = 108.051(11)°.
	c = 13.0725(13) Å γ = 90°.
Volume	1196.3(2) $Å^3$
Z	2
Density (calculated)	1.284 mg/m^3
Absorption coefficient	0.751 mm^{-1}
F(000)	500
Crystal size	0.140 × 0.120 × 0.050 mm^3
Theta range for data collection	7.112 o 142.232°
Index ranges	$-9 \leq h \leq 10$, $-12 \leq k \leq 14$, $-16 \leq l \leq 15$
Reflections collected	8382
Independent reflections	4118 [R(int) = 0.0286]
Absorption correction	Semi-empirical from equivalents
Refinement method	Full-matrix least-squares on F^2
Data / restraints / parameters	4118 /1/ 307
Goodness-of-fit on F^2	1.048
Final R indices [I>2sigma(I)]	R1 = 0.0362 wR2 = 0.0930
R indices (all data)	R1 = 0.0387, wR2 = 0.0952
Absolute structure parameter	0.08(11)
Largest diff. peak and hole	0.264 /-0.189 e.$Å^{-3}$

Figure S53. X-ray structure of 4

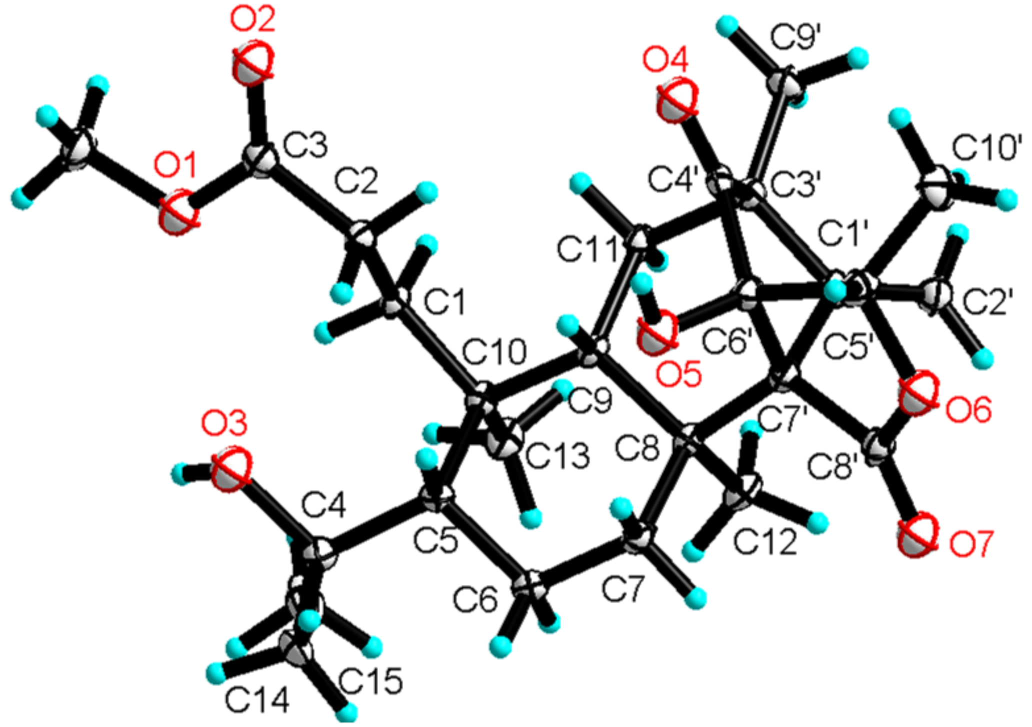

Figure S54. HR-ESIMS spectrum of 5

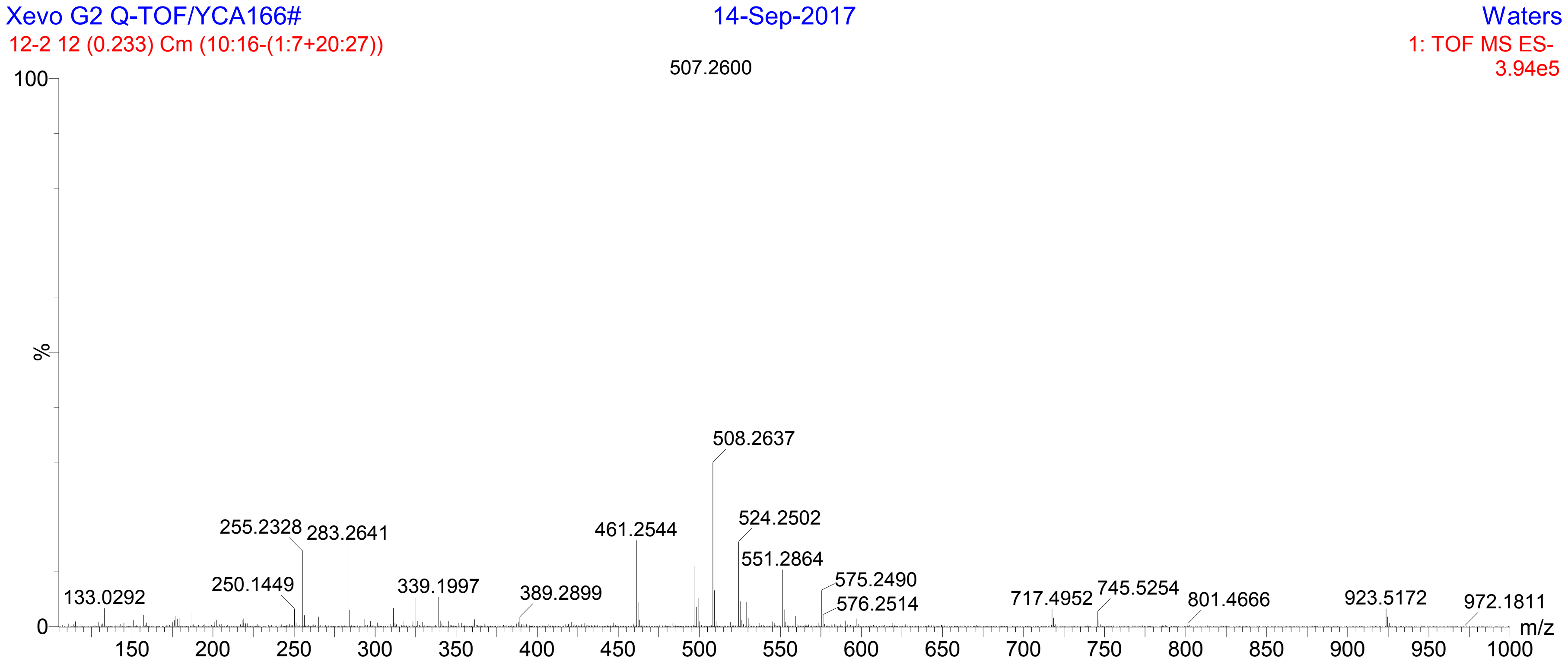

Mass	Calc. Mass	mDa	PPM	DBE	i-FIT	Norm	Conf(%)	Formula
507.2600	507.2594	0.6	1.2	8.5	292.8	n/a	n/a	$C_{27}H_{39}O_9$

Figure S55. IR spectrum of 5

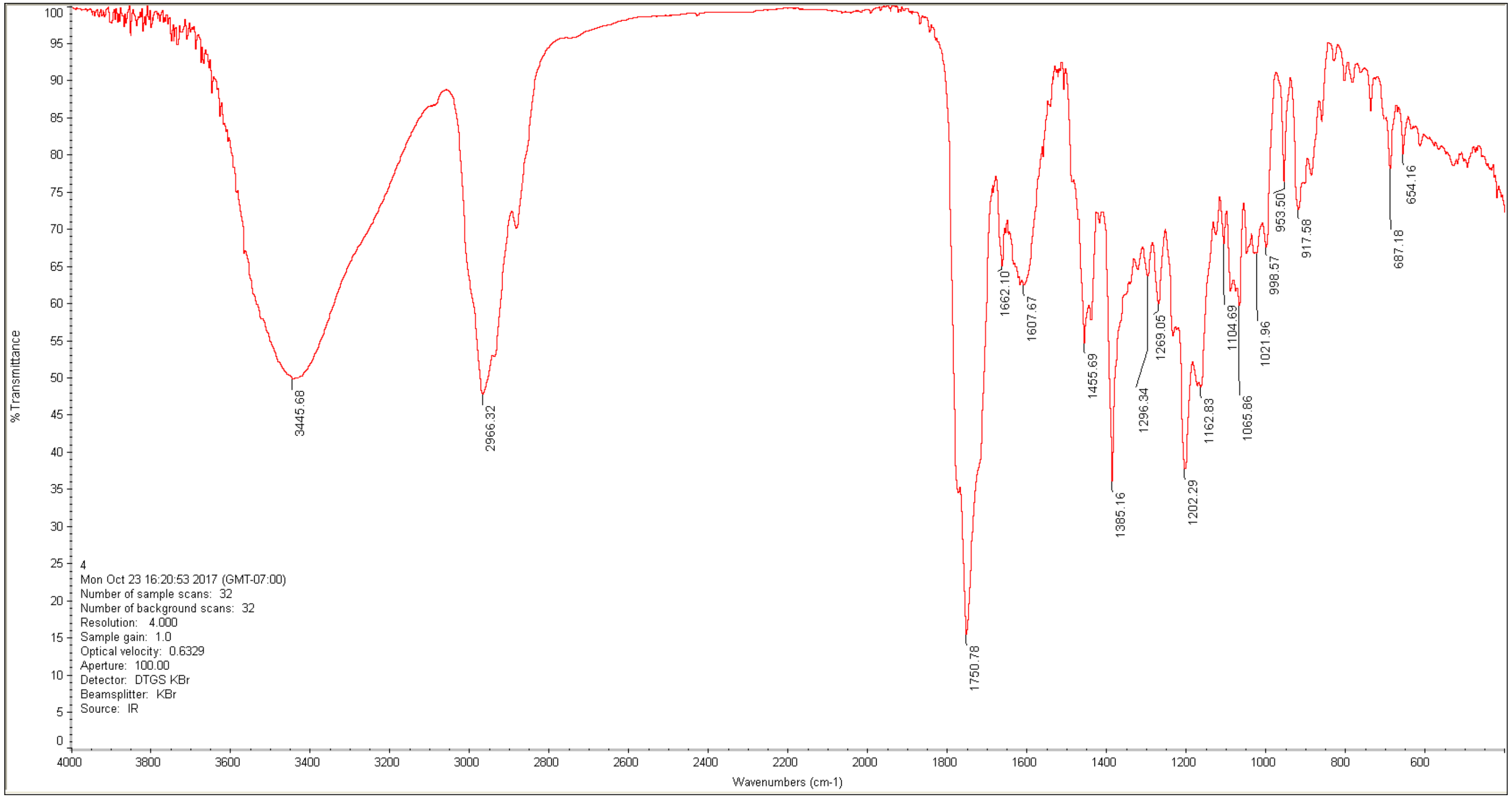

Figure S56. UV spectrum of 5 in CH_3OH

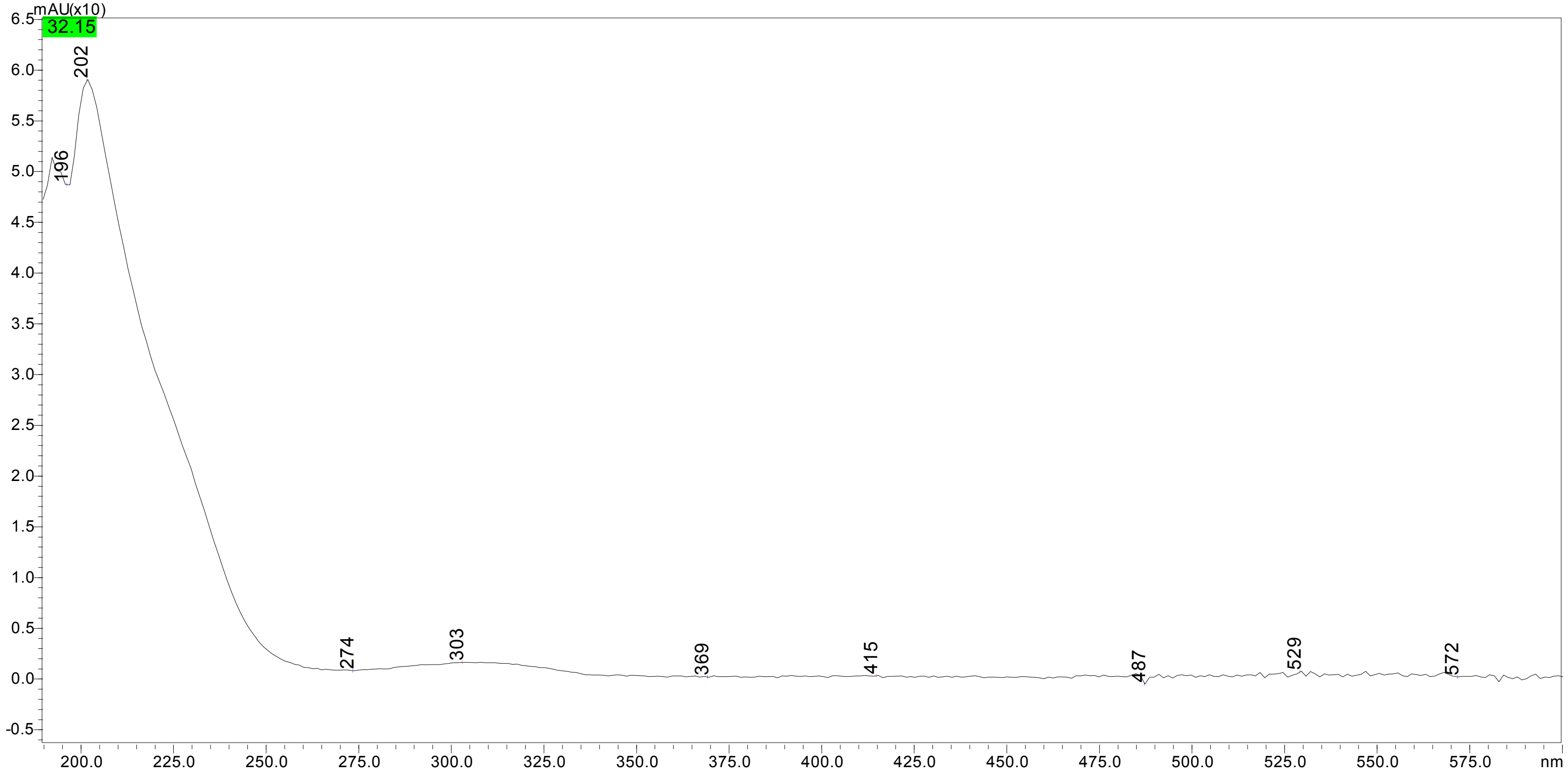

Figure S57. ^{1}H NMR spectrum of 5 in DMSO-d_6

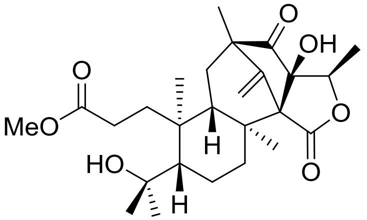

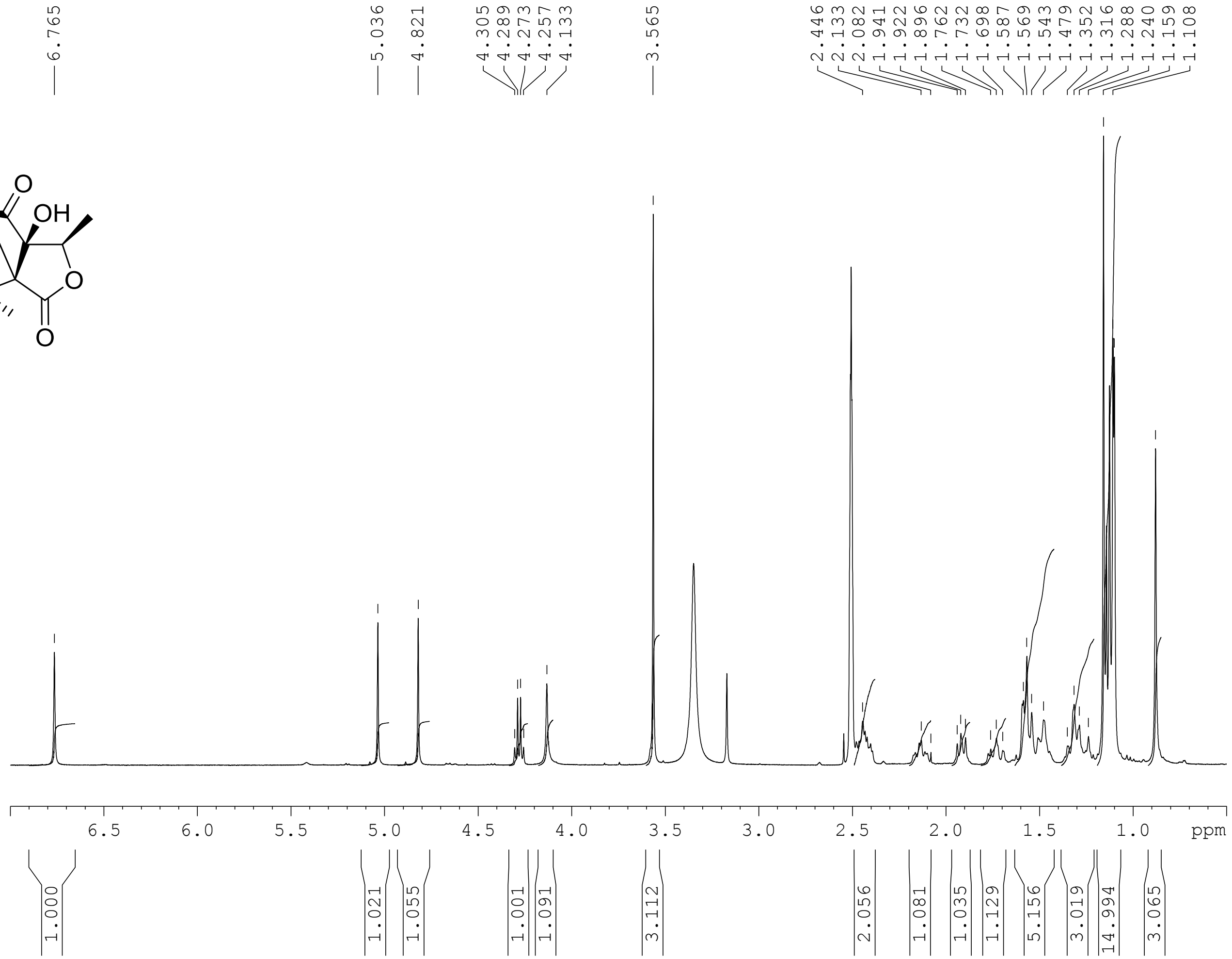

Figure S58. ^{13}C NMR spectra of 5 in DMSO-d_6

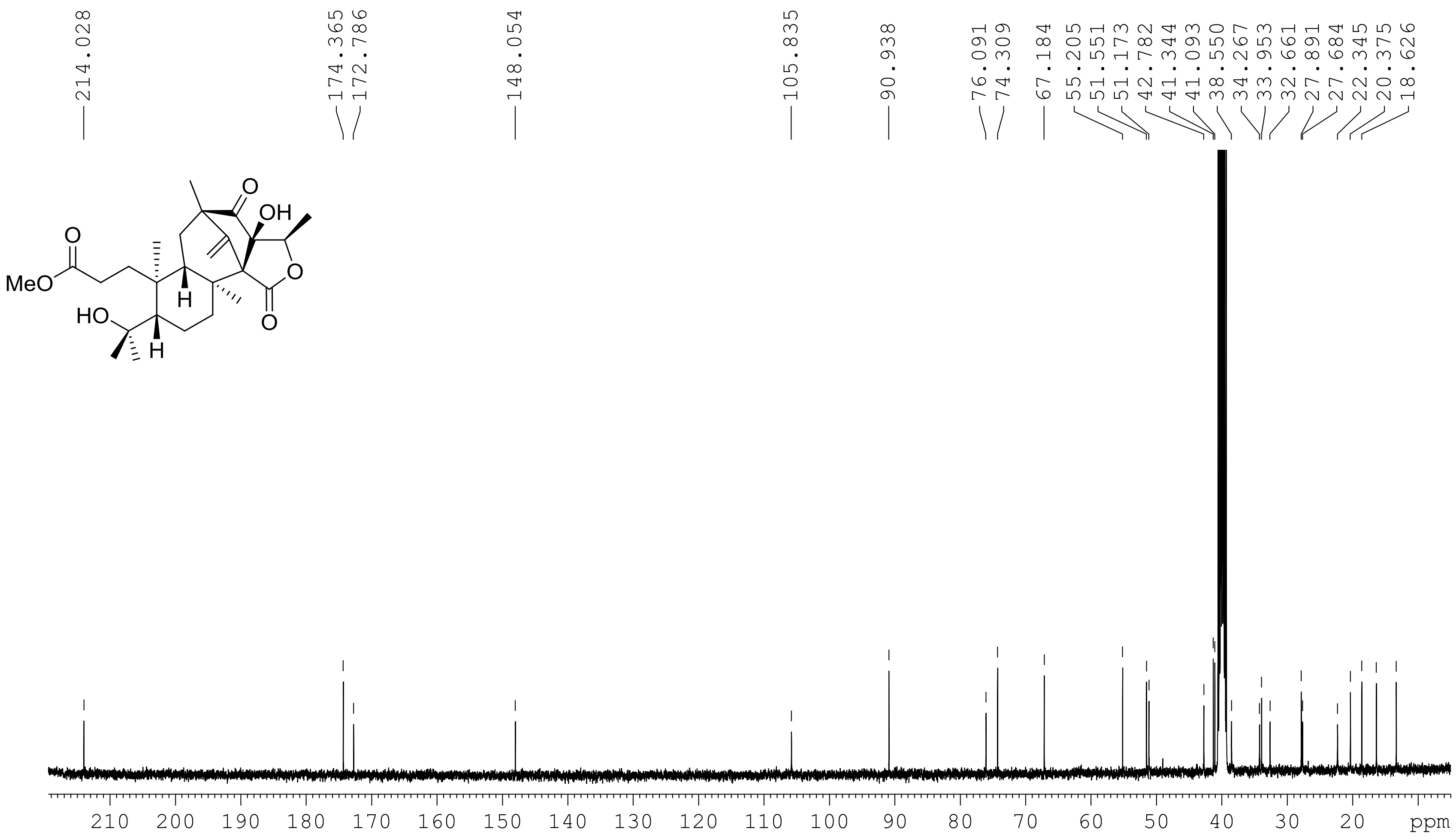

Figure S59. DEPT spectra of 5 in DMSO-d_6

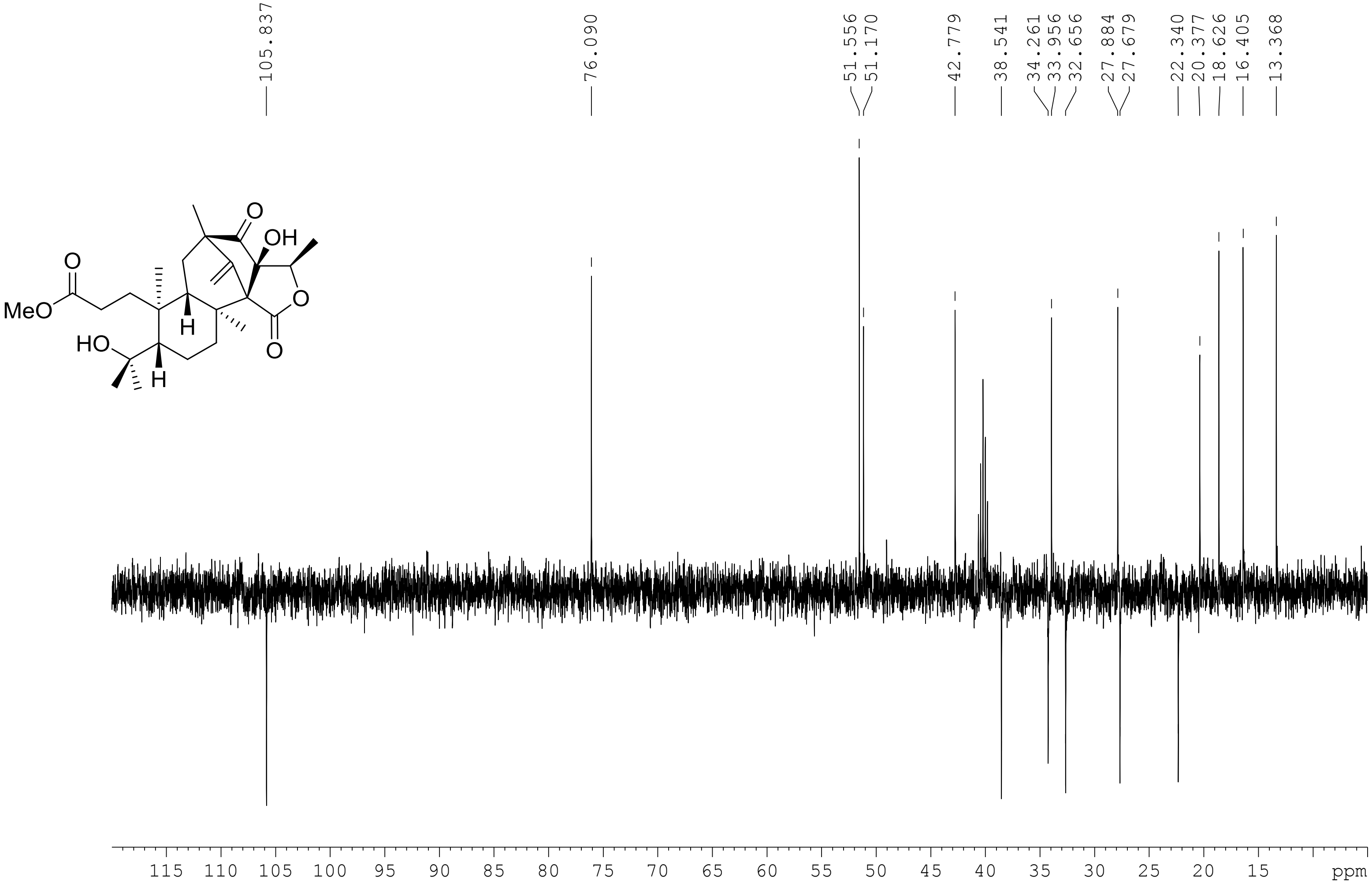

Figure S60. ^{1}H-^{1}H COSY spectrum of 5 in DMSO-d_6

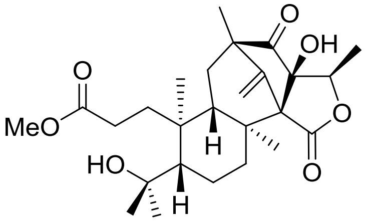

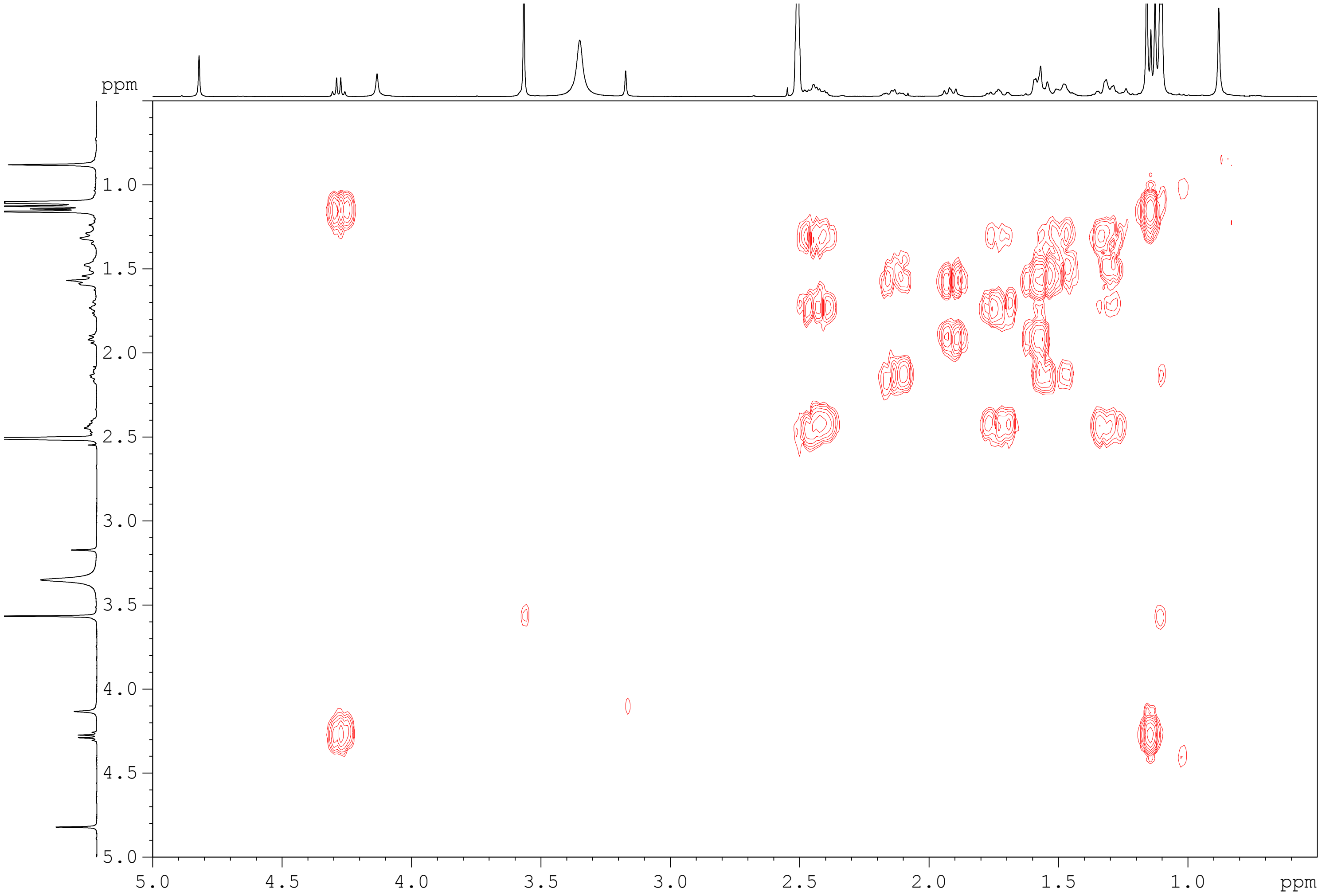

Figure S61. HSQC spectrum of 5 in DMSO-d_6

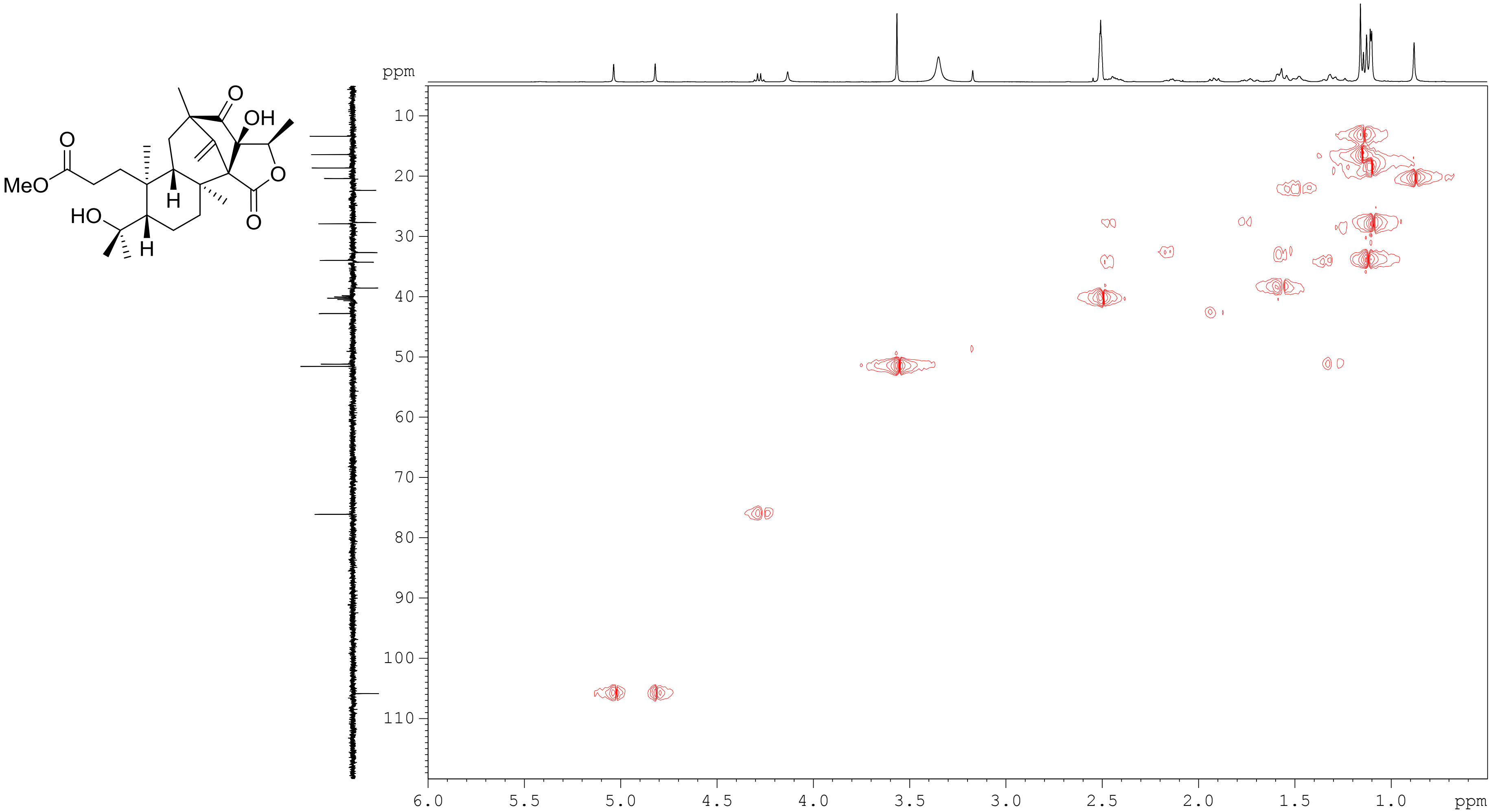

Figure S62. HMBC spectrum of 5 in DMSO-d_6

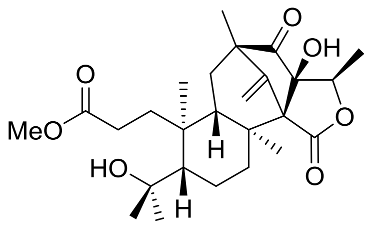

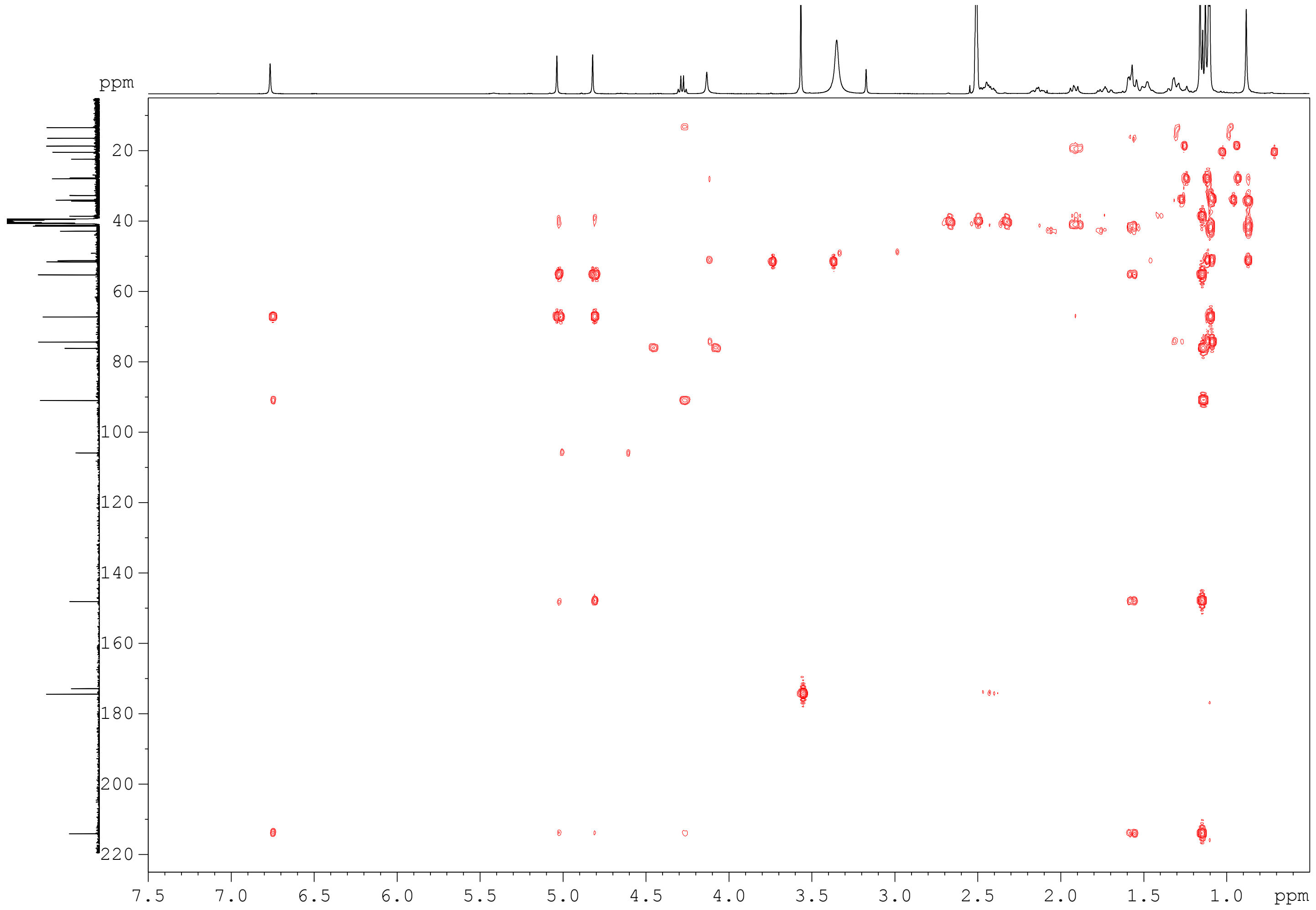

Figure S63. NOESY spectrum of 5 in DMSO-d_6

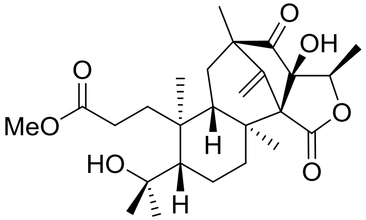

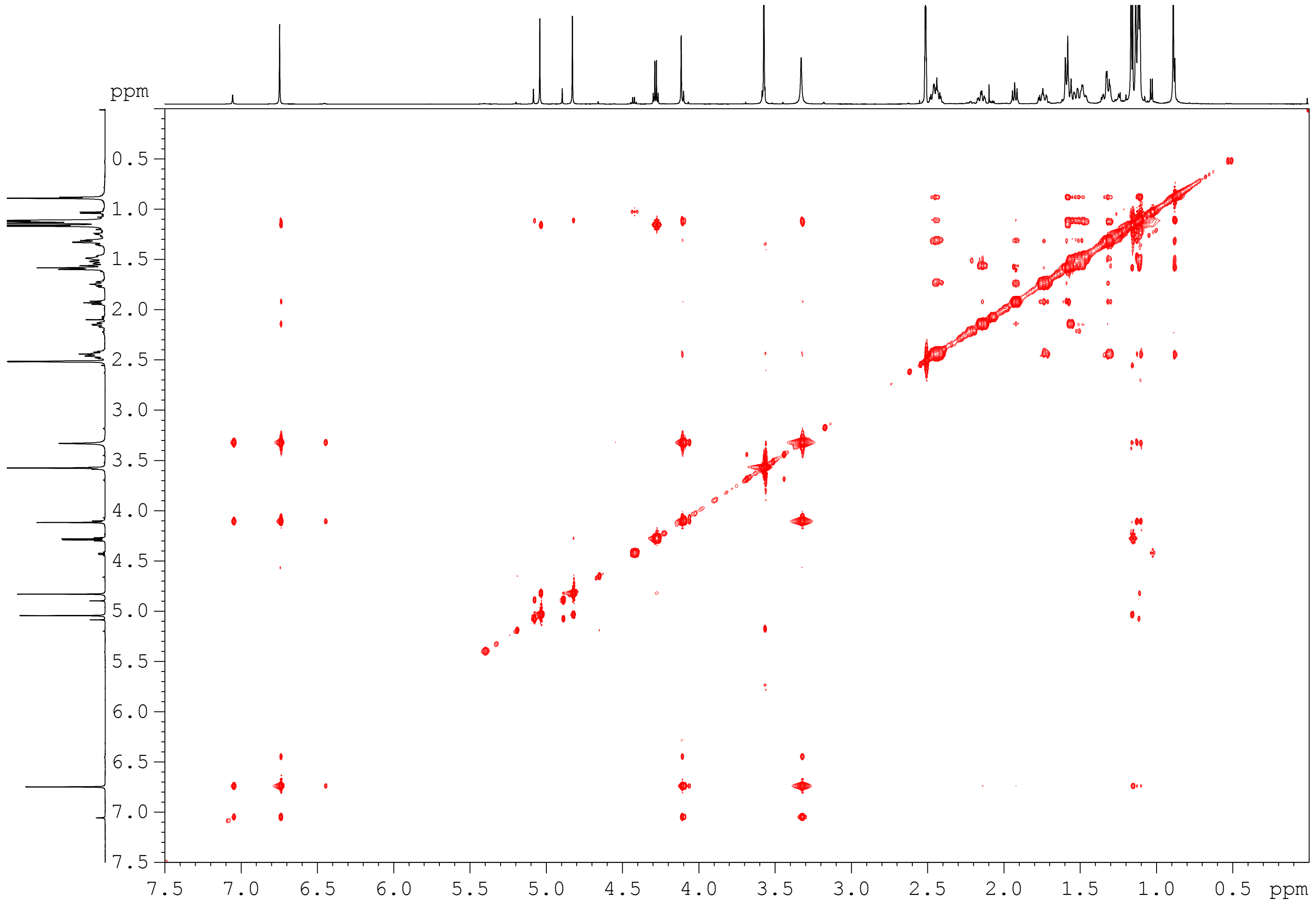

X-ray crystallographic data of 5

Empirical formula	$C_{26}H_{38}O_7$
Formula weight	462.56
Temperature	108.1 K
Crystal system	orthorhombic
Space group	$P2_12_12_1$
Unit cell dimensions	
	a = 9.6361(2) Å α = 90°.
	b = 11.6949(3) Å β = 90°.
	c = 21.1293(5) Å γ = 90°.
Volume	2381.12(2) $Å^3$
Z	4
Density (calculated)	1.290 mg/m^3
Absorption coefficient	0.754 mm^{-1}
F(000)	1000
Crystal size	0.350 × 0.300 × 0.280 mm^3
Theta range for data collection	8.37 o 142.32°
Index ranges	$-10 \leq h \leq 11$, $-14 \leq k \leq 12$, $-25 \leq l \leq 23$
Reflections collected	8393
Independent reflections	4484 [R(int) = 0.0228]
Absorption correction	Semi-empirical from equivalents
Refinement method	Full-matrix least-squares on F^2
Data / restraints / parameters	4484 /0/ 307
Goodness-of-fit on F^2	1.038
Final R indices [I>2sigma(I)]	R1 = 0.0376 wR2 = 0.0973
R indices (all data)	R1 = 0.0383, wR2 = 0.0978
Absolute structure parameter	-0.06(9)
Largest diff. peak and hole	0.369 /-0.195 e.$Å^{-3}$

Figure S64. X-ray structure of 5

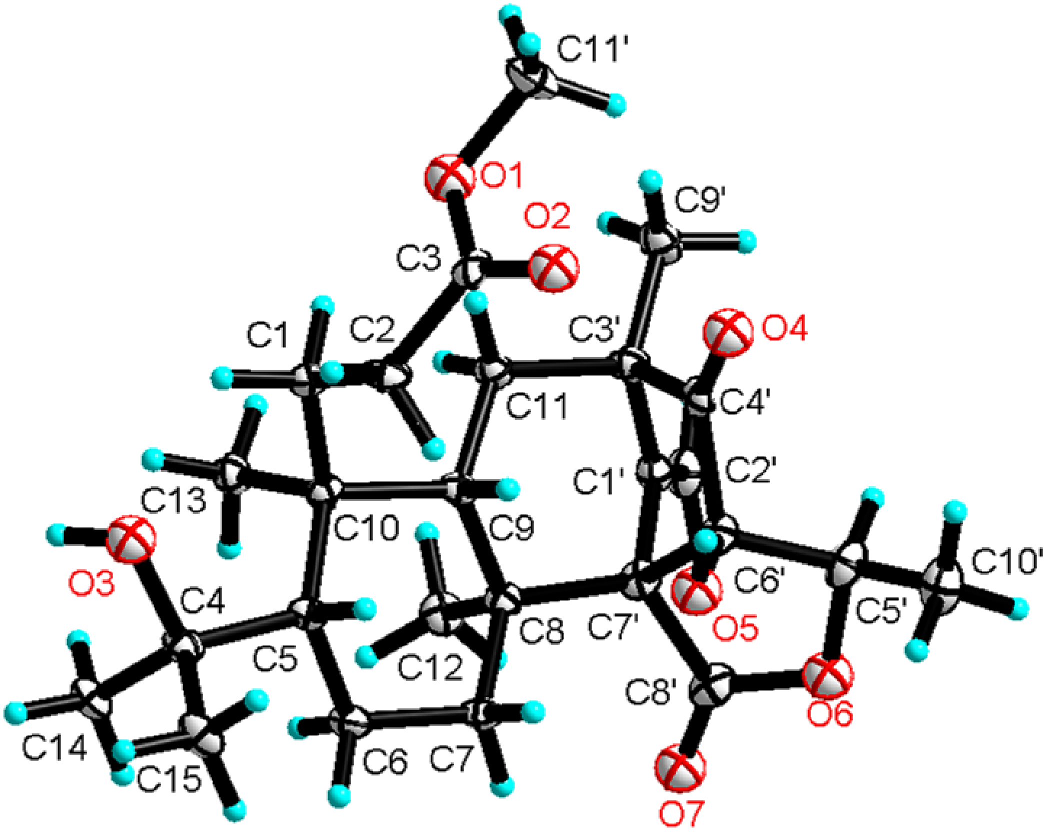

Figure S65. HR-ESIMS spectrum of 6

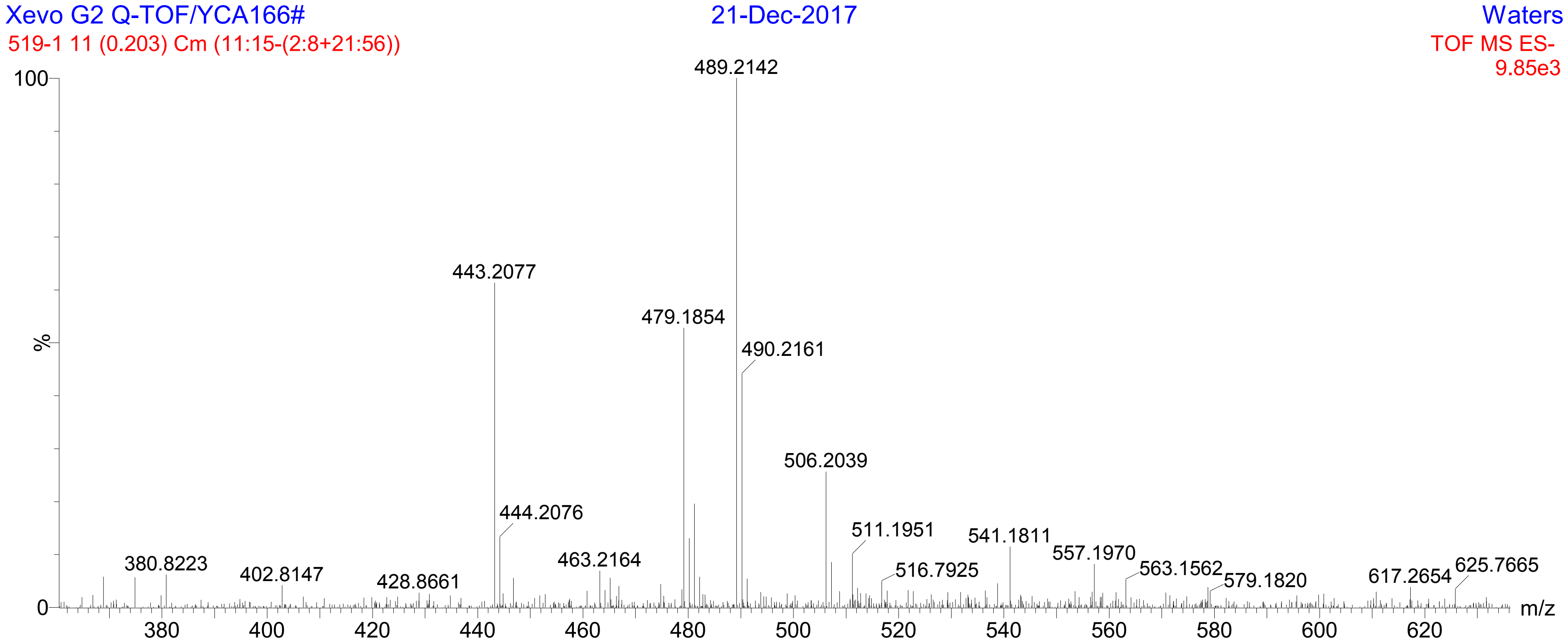

Mass	Calc. Mass	mDa	PPM	DBE	i-FIT	Norm	Conf(%)	Formula
443.2077	443.2070	0.7	1.6	10.5	110.5	n/a	n/a	$C_{25} H_{31} O_7$

Figure S66. IR spectrum of 6

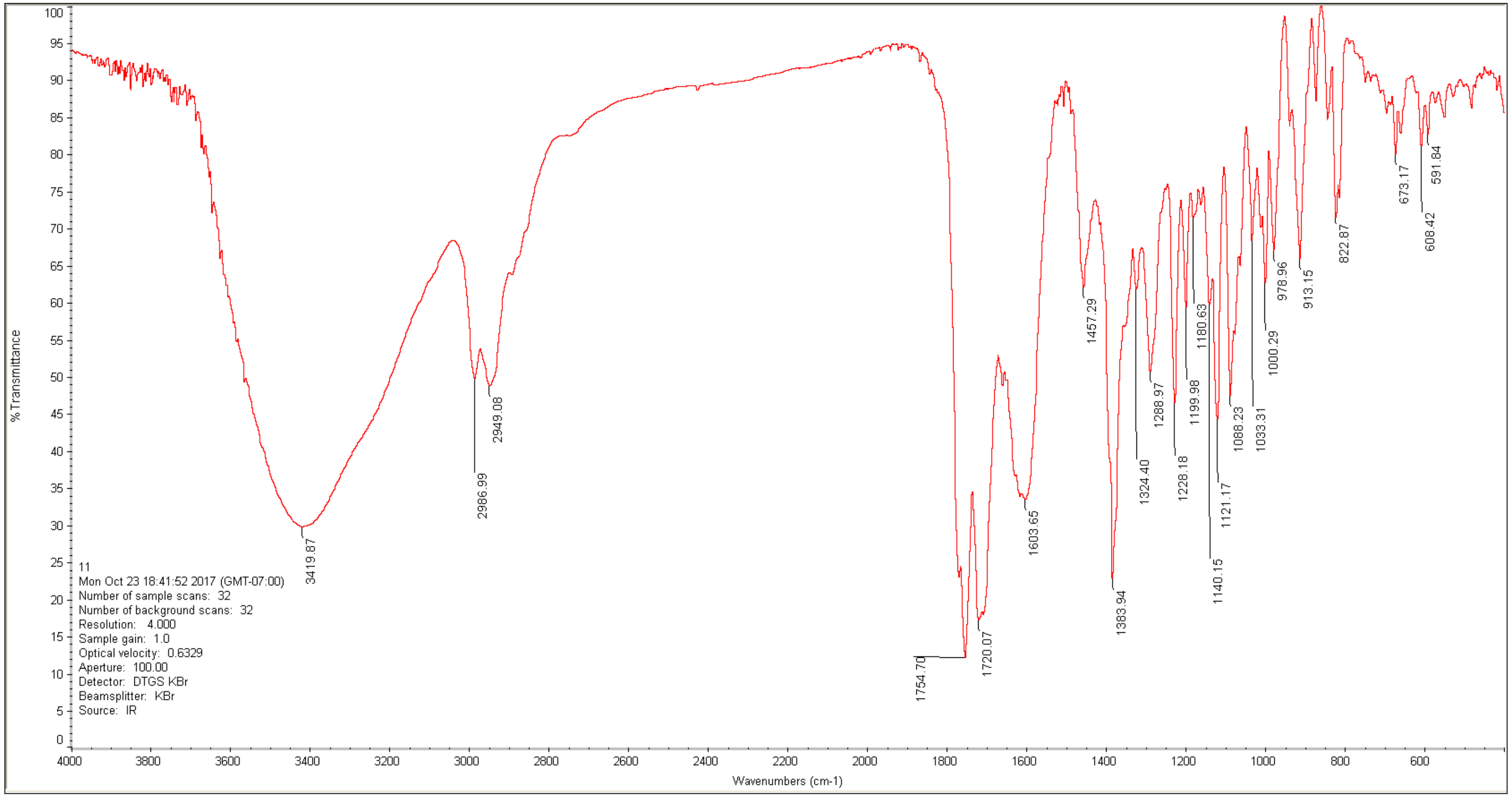

Figure S67. UV spectrum of 6 in CH_3OH

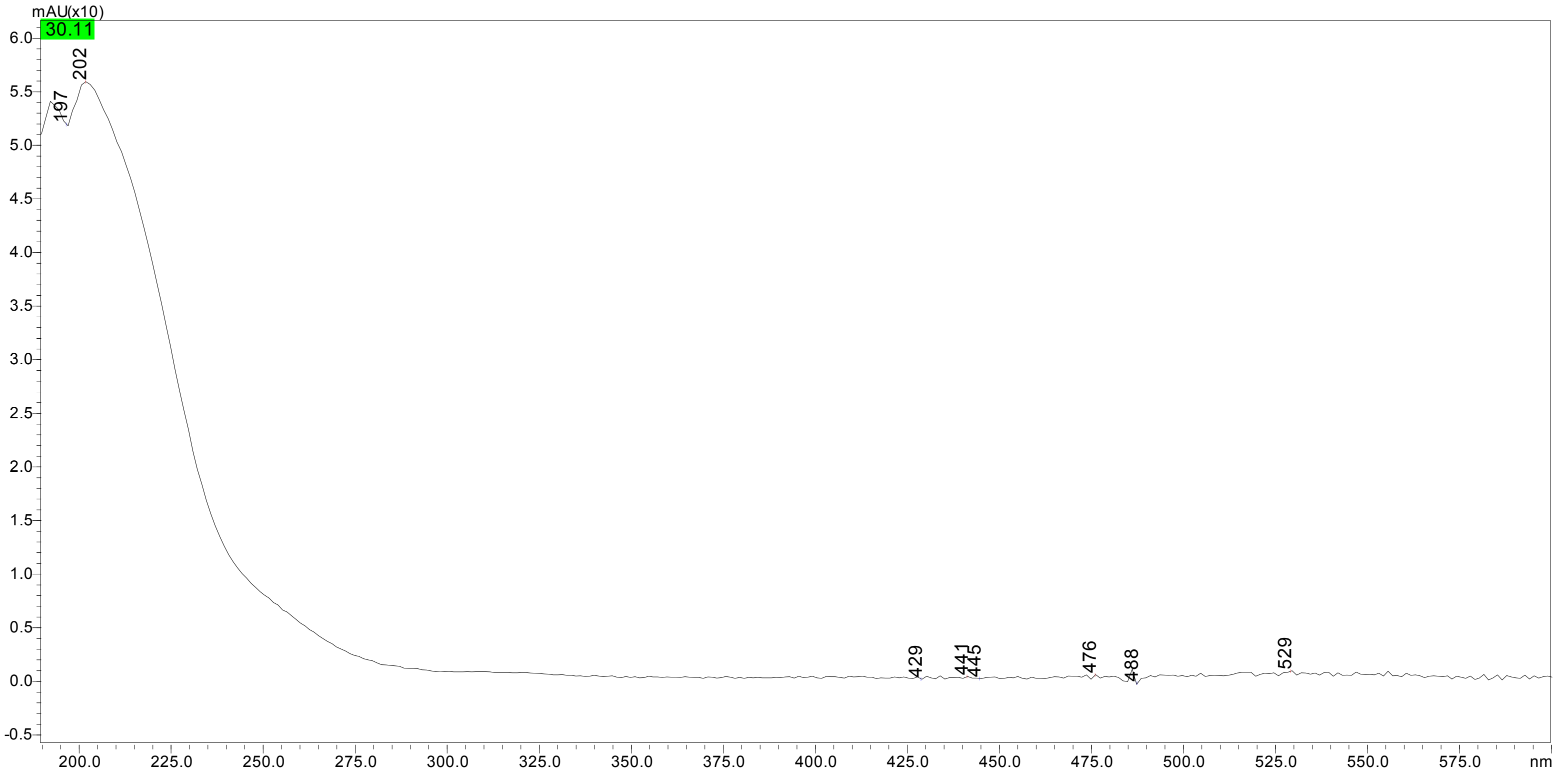

Figure S68. ^{1}H- NMR spectrum of 6 in DMSO-d_6

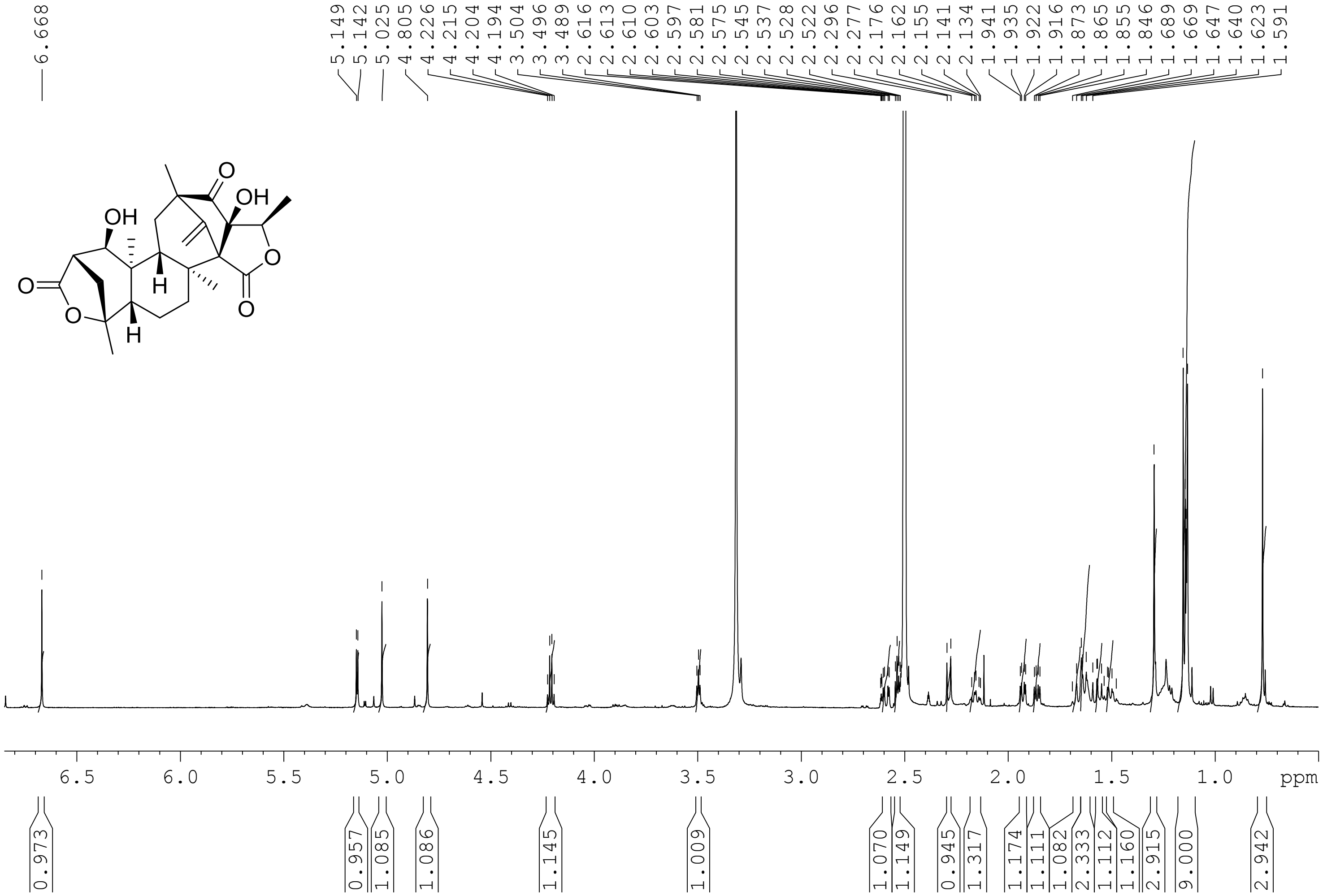

Figure S69. APT spectra of 6 in DMSO-d_6

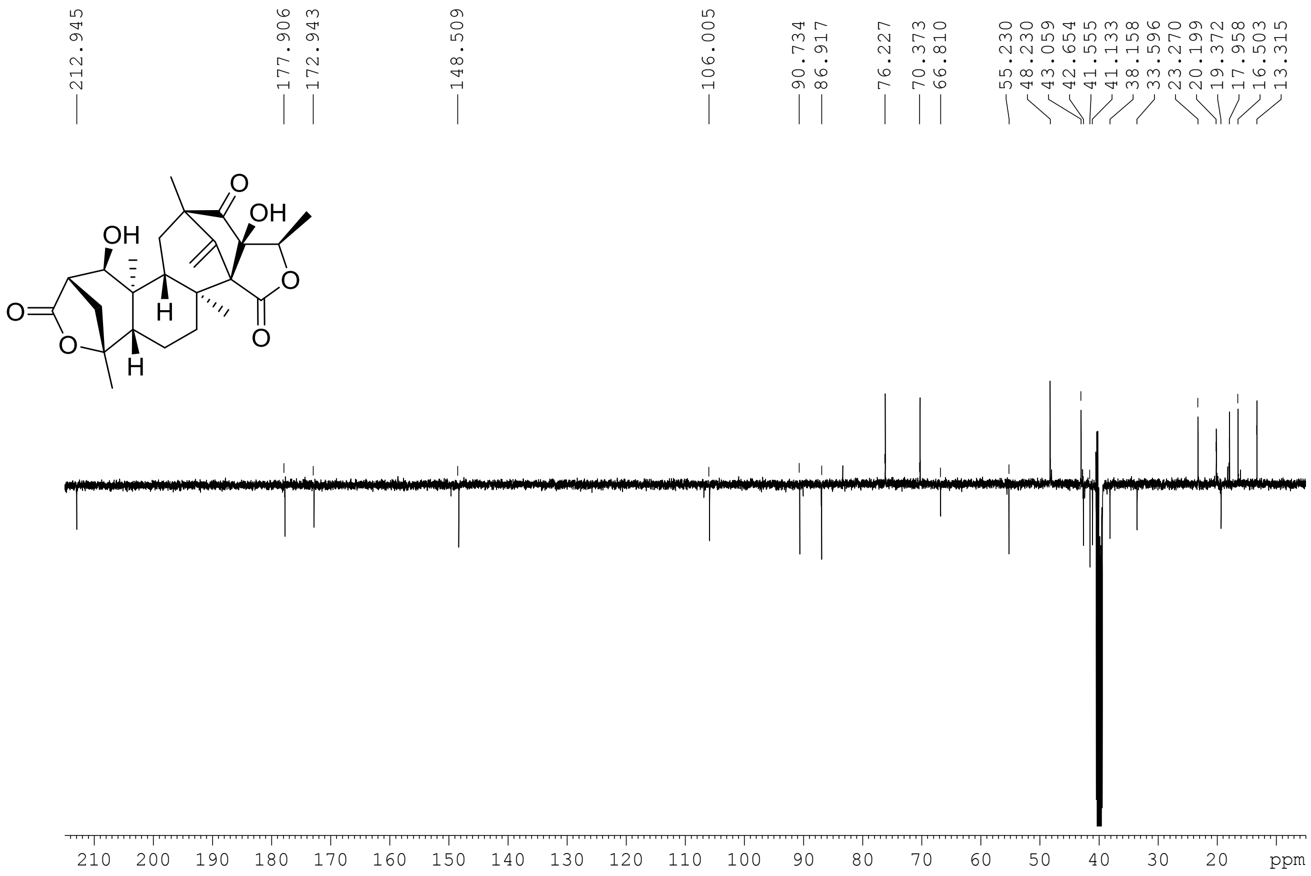

Figure S70. ^{1}H-^{1}H COSY spectrum of 6 in DMSO-d_6

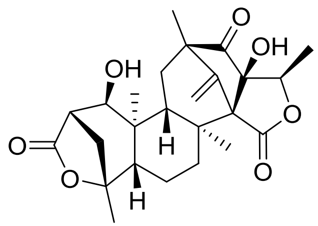

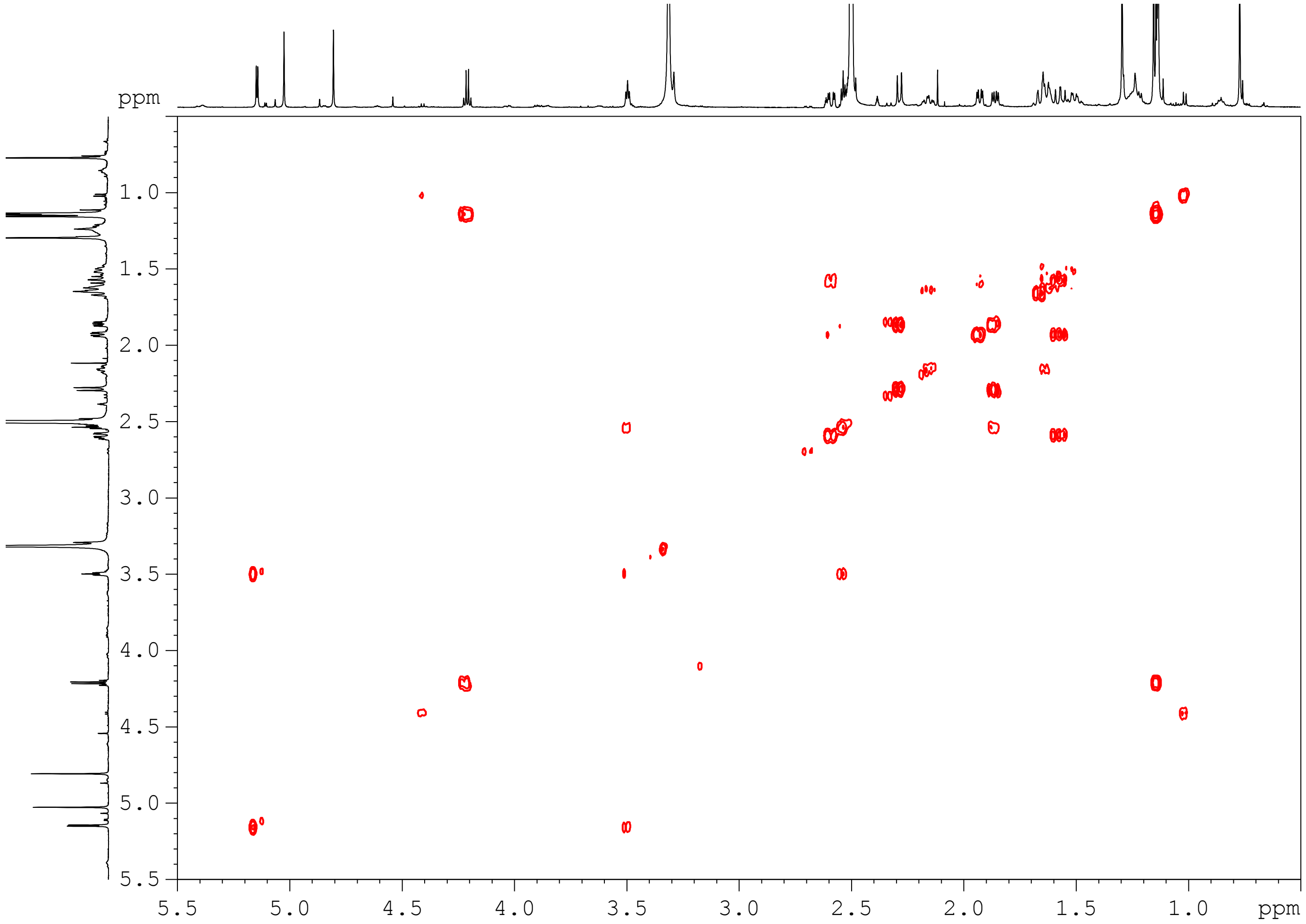

Figure S71. HSQC spectrum of 6 in DMSO-d_6

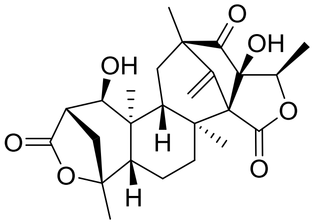

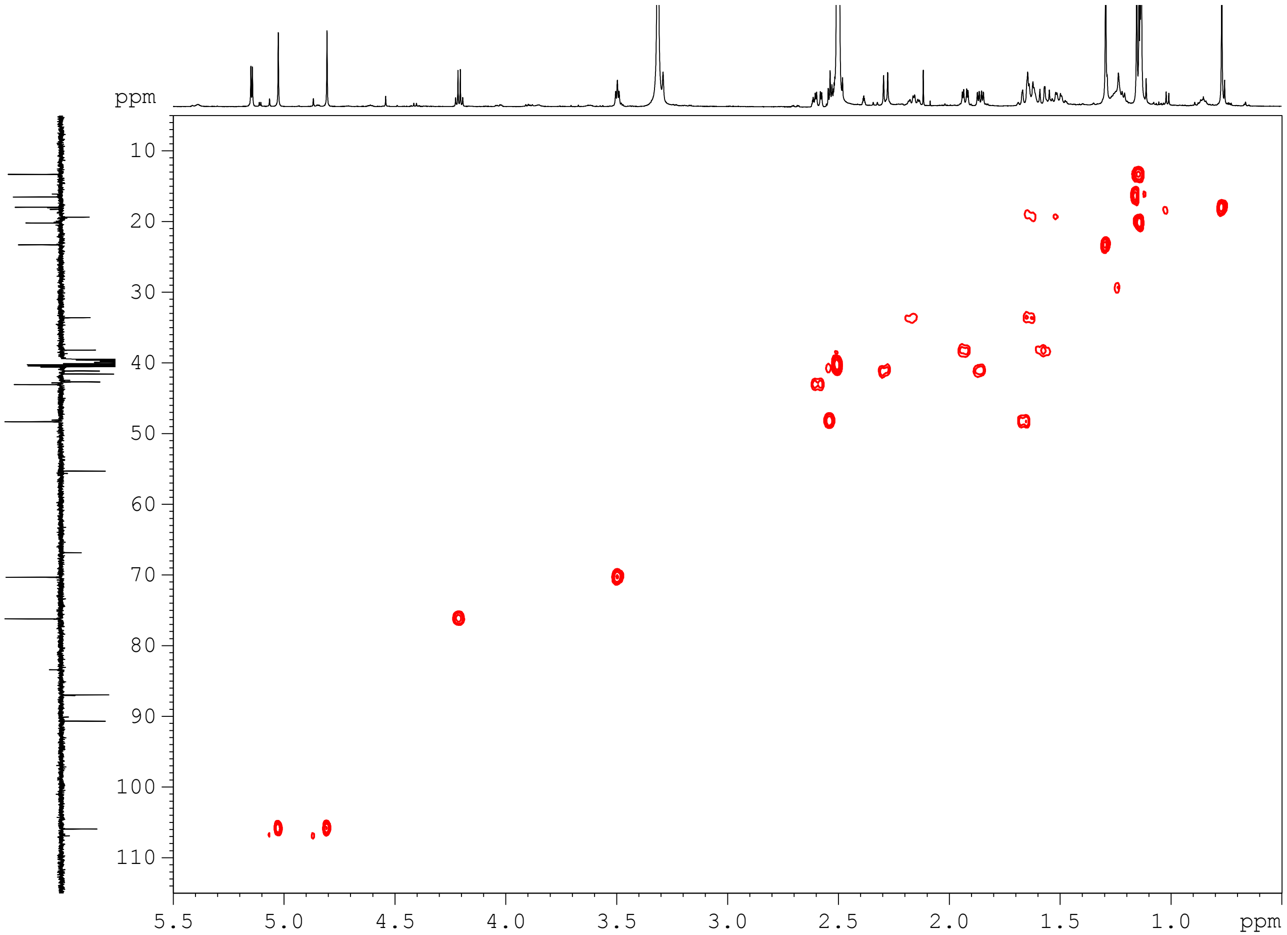

Figure S72. HMBC spectrum of 6 in DMSO-d_6

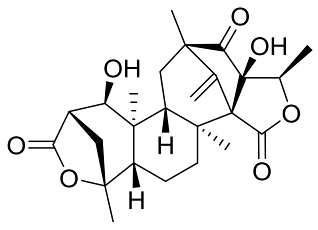

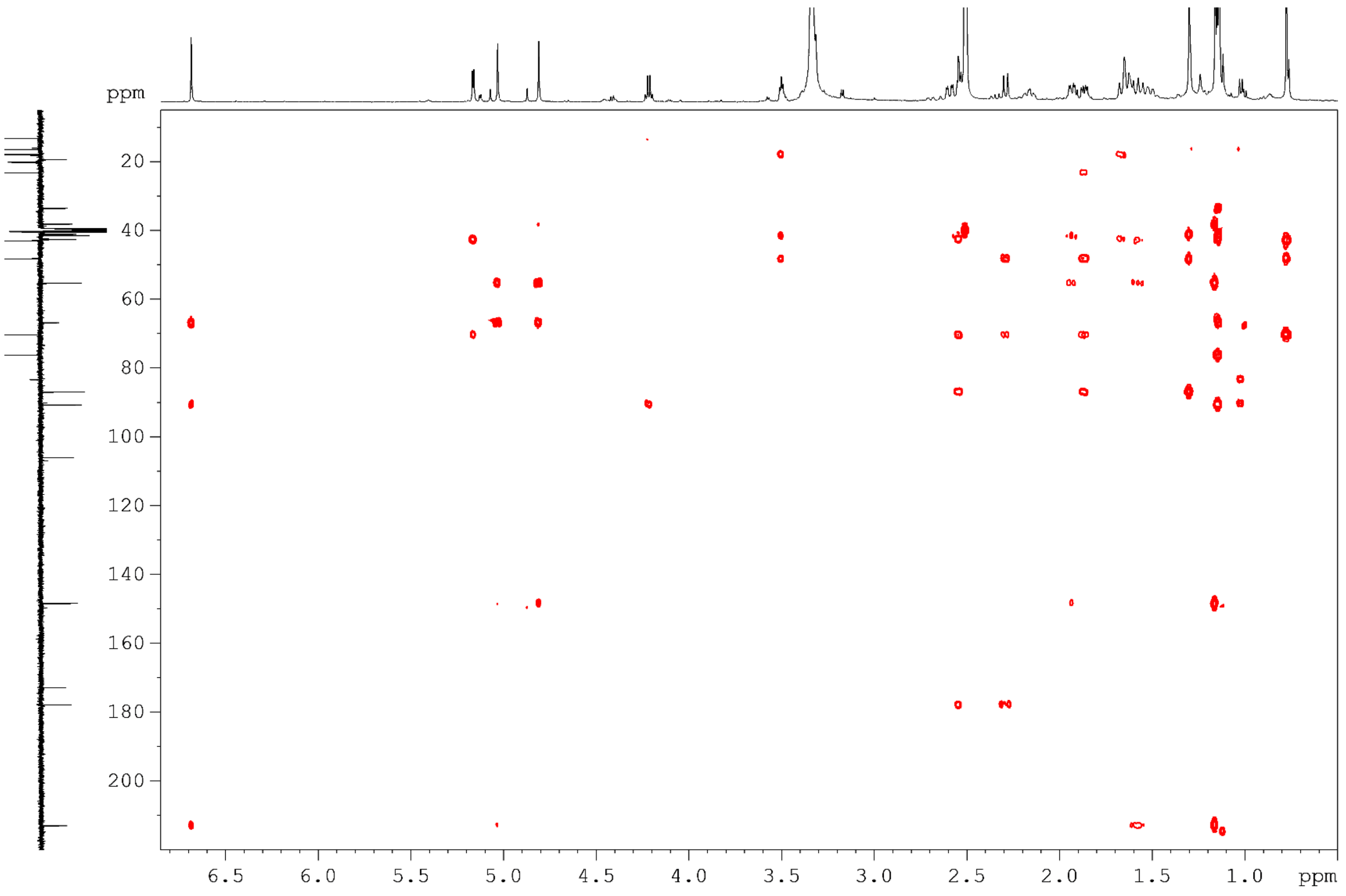

Figure S73. NOESY spectrum of 6 in DMSO-d_6

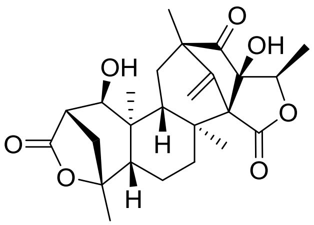

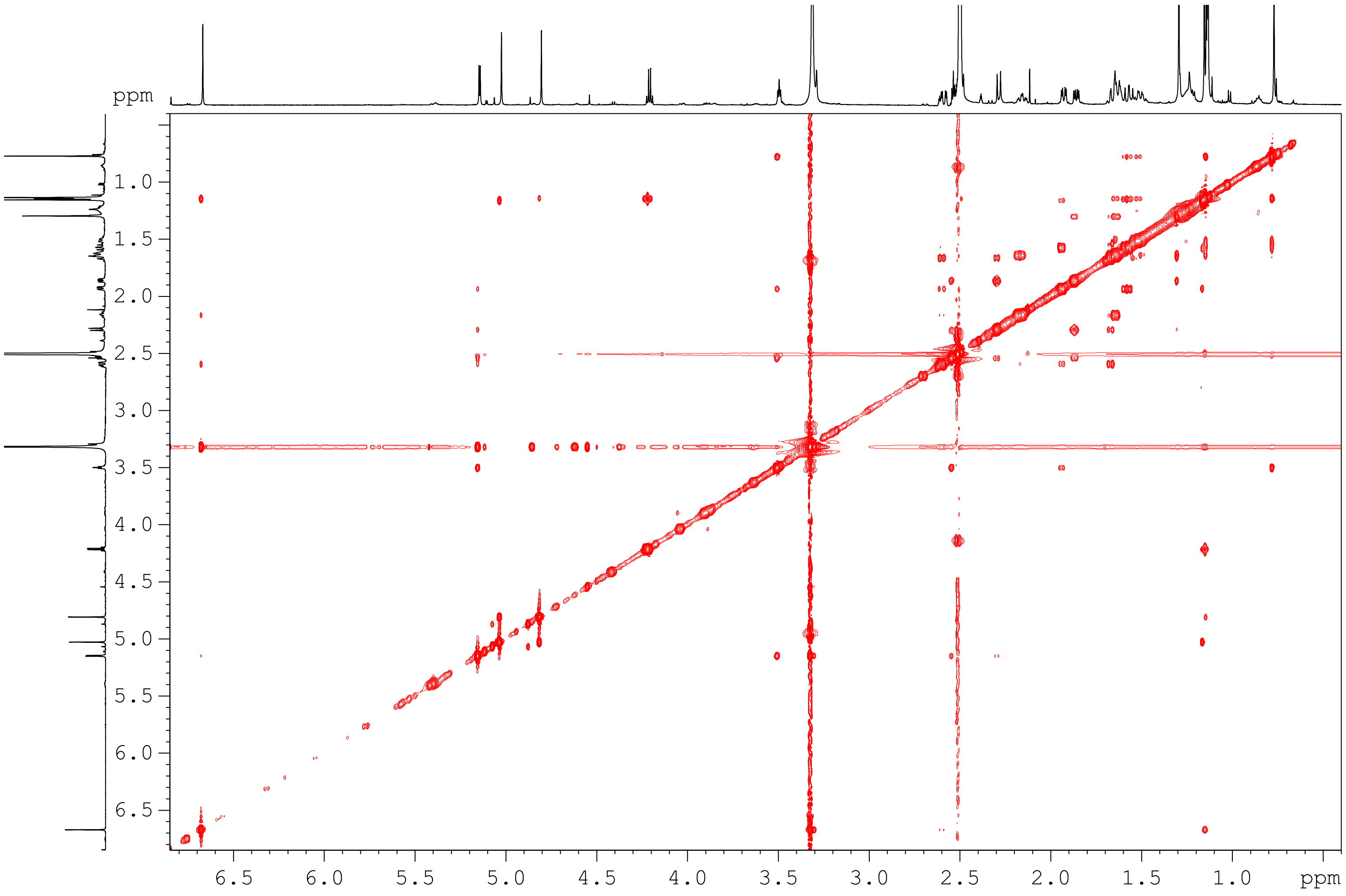

Figure S74. NOESY spectrum of 6 in DMSO-d_6 showing the key correlations from OH-6' to H_3-9' and H-9; OH-1 to H_2-15 and H-9

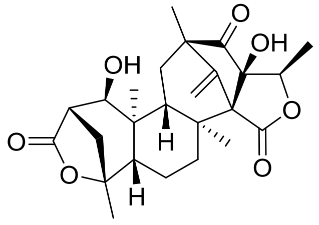

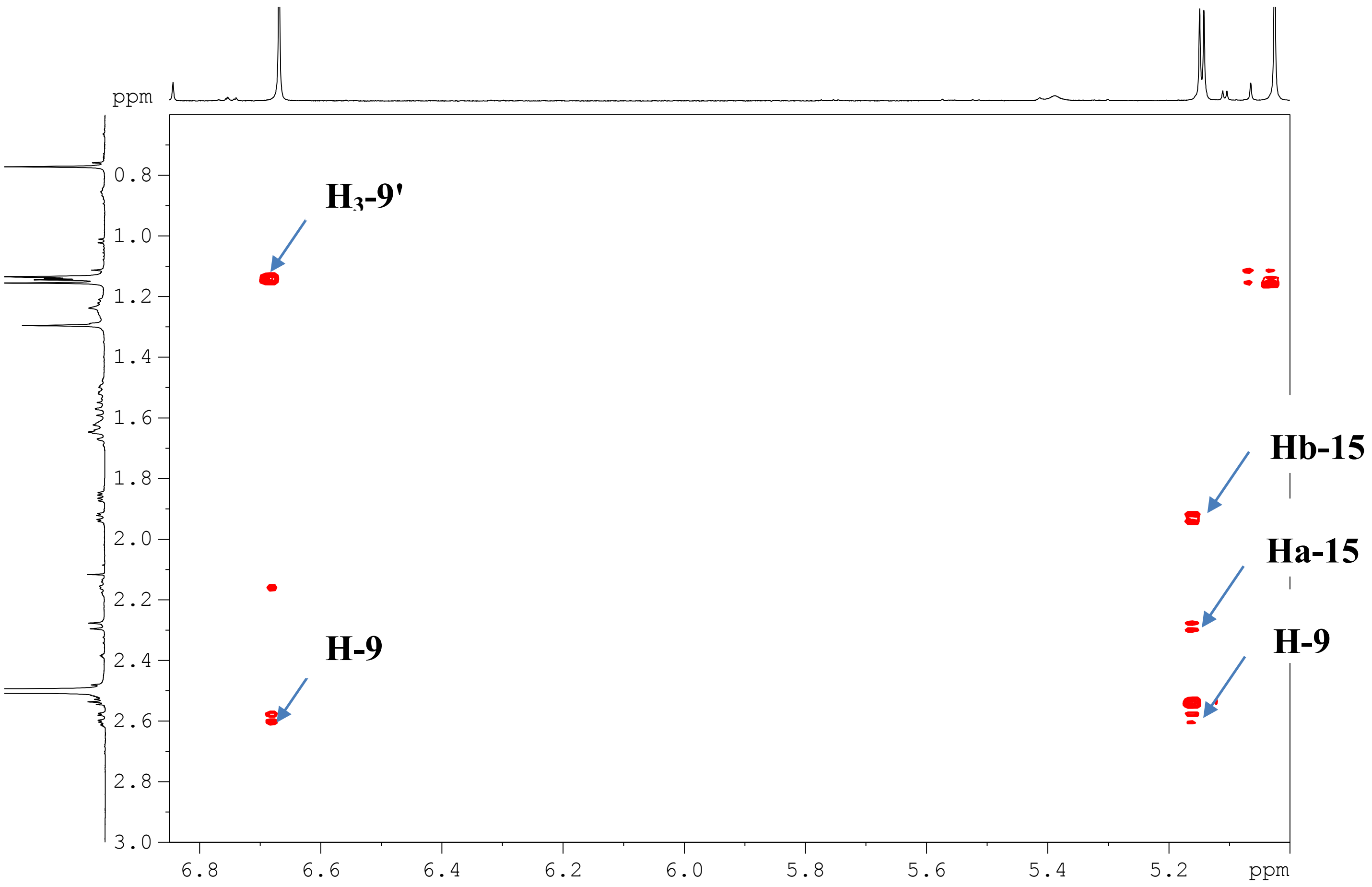

Figure S75. NOESY spectrum of 6 in DMSO-d_6 showing the key correlations from H-5 to H_2-15 and H-9

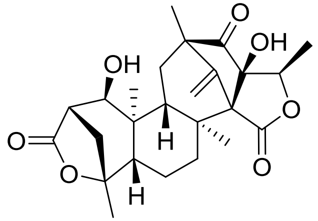

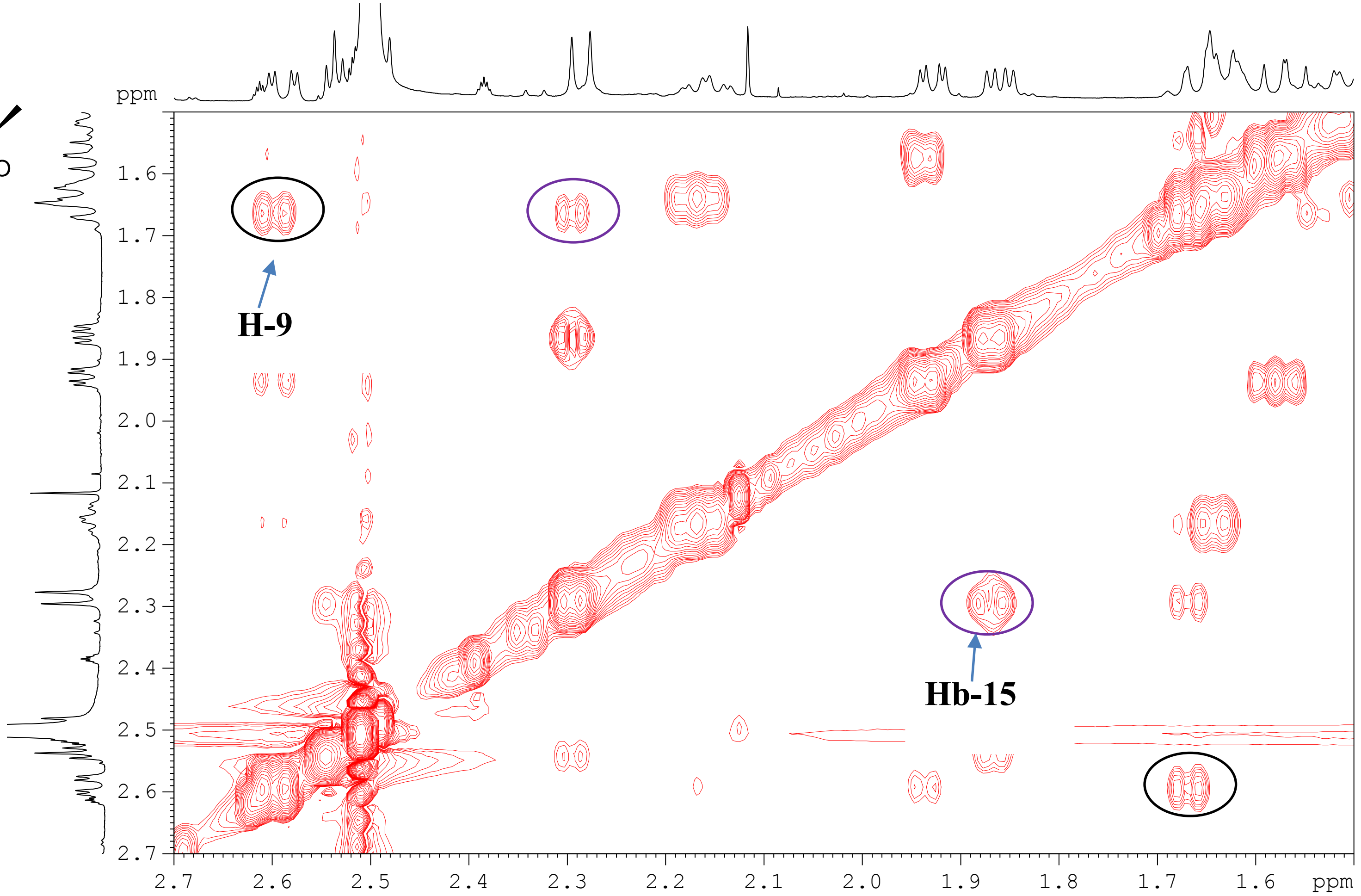

Figure S76. HR-ESIMS spectrum of 7

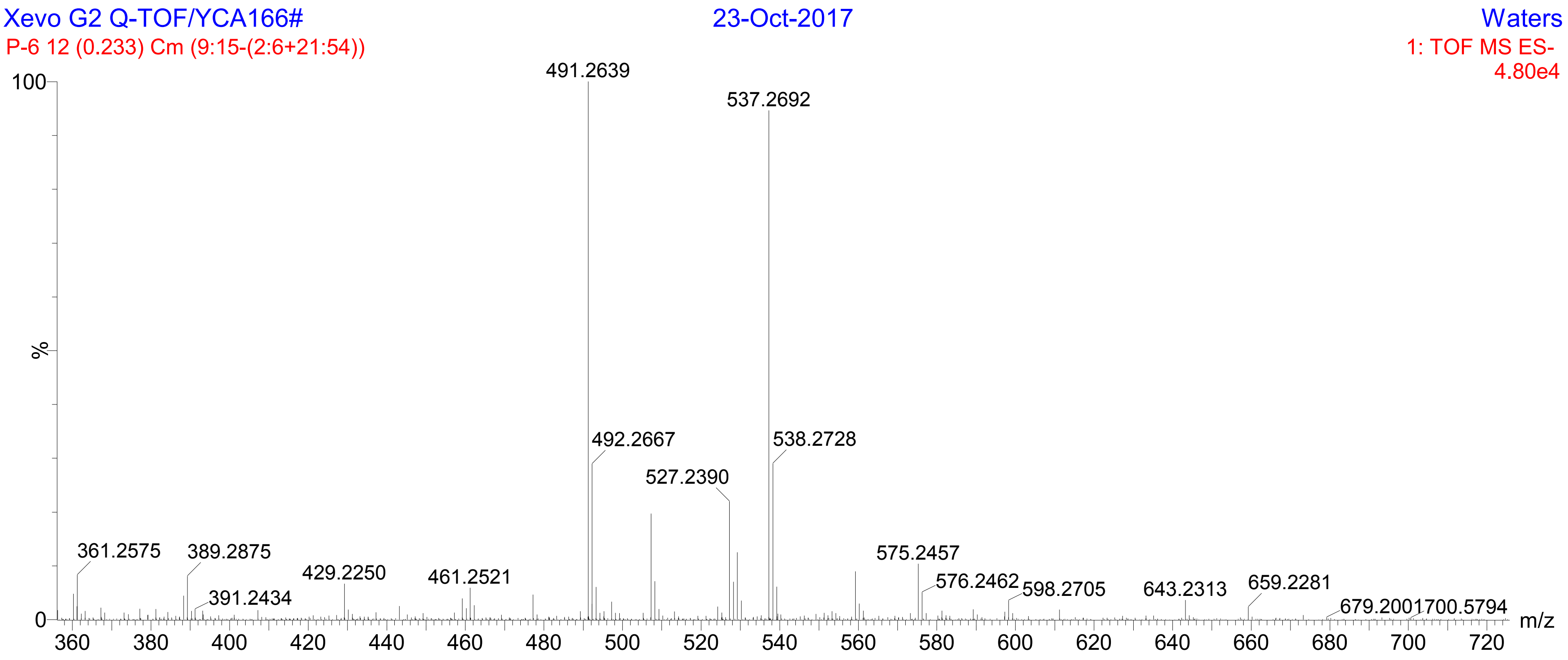

Mass	Calc. Mass	mDa	PPM	DBE	i-FIT	Norm	Conf(%)	Formula
491.2639	491.2645	-0.6	-1.2	8.5	113.3	0.000	100.00	$C_{27}H_{39}O_8$

Figure S77. IR spectrum of 7

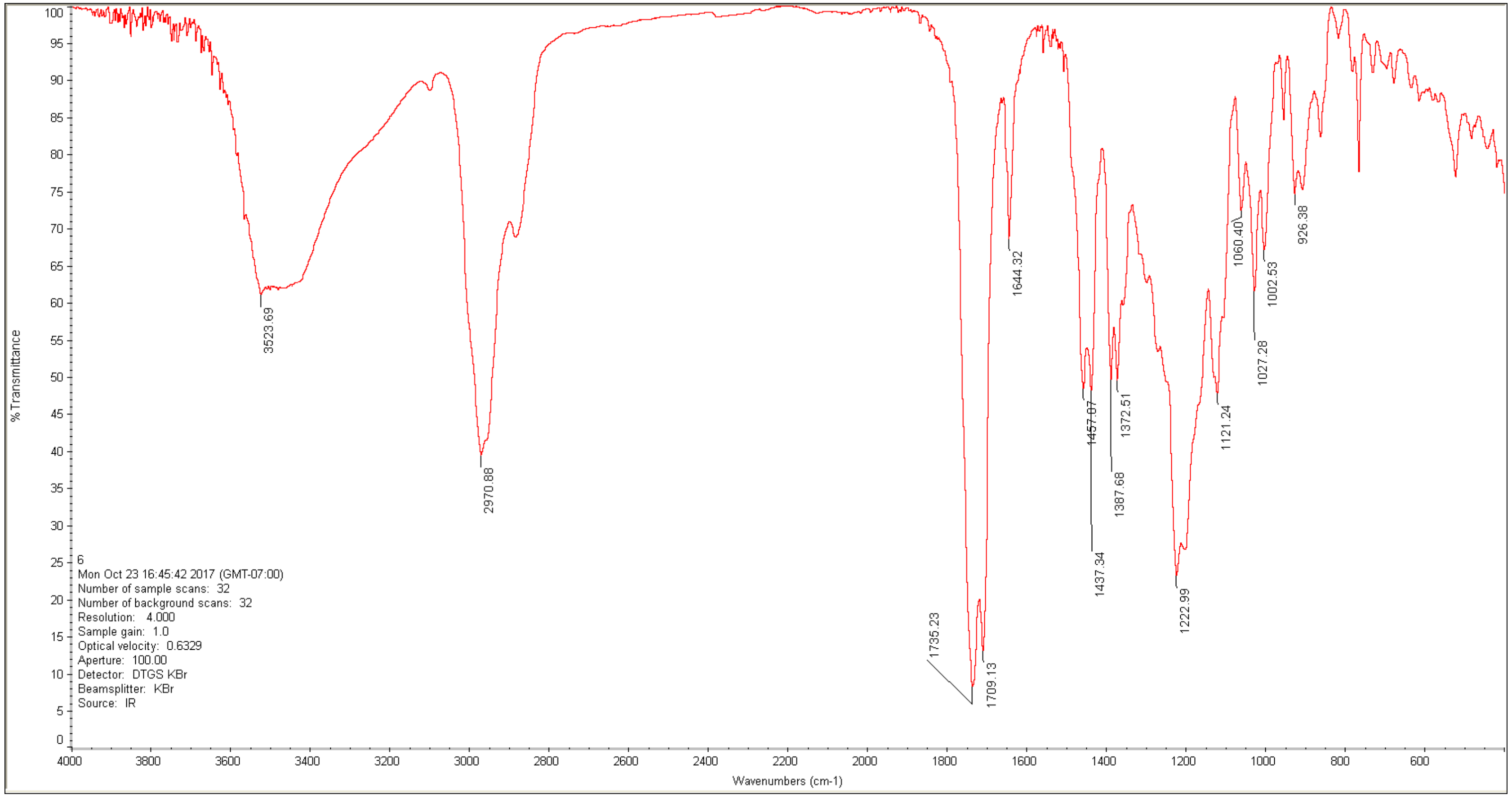

Figure S78. UV spectrum of 7 in CH_3OH

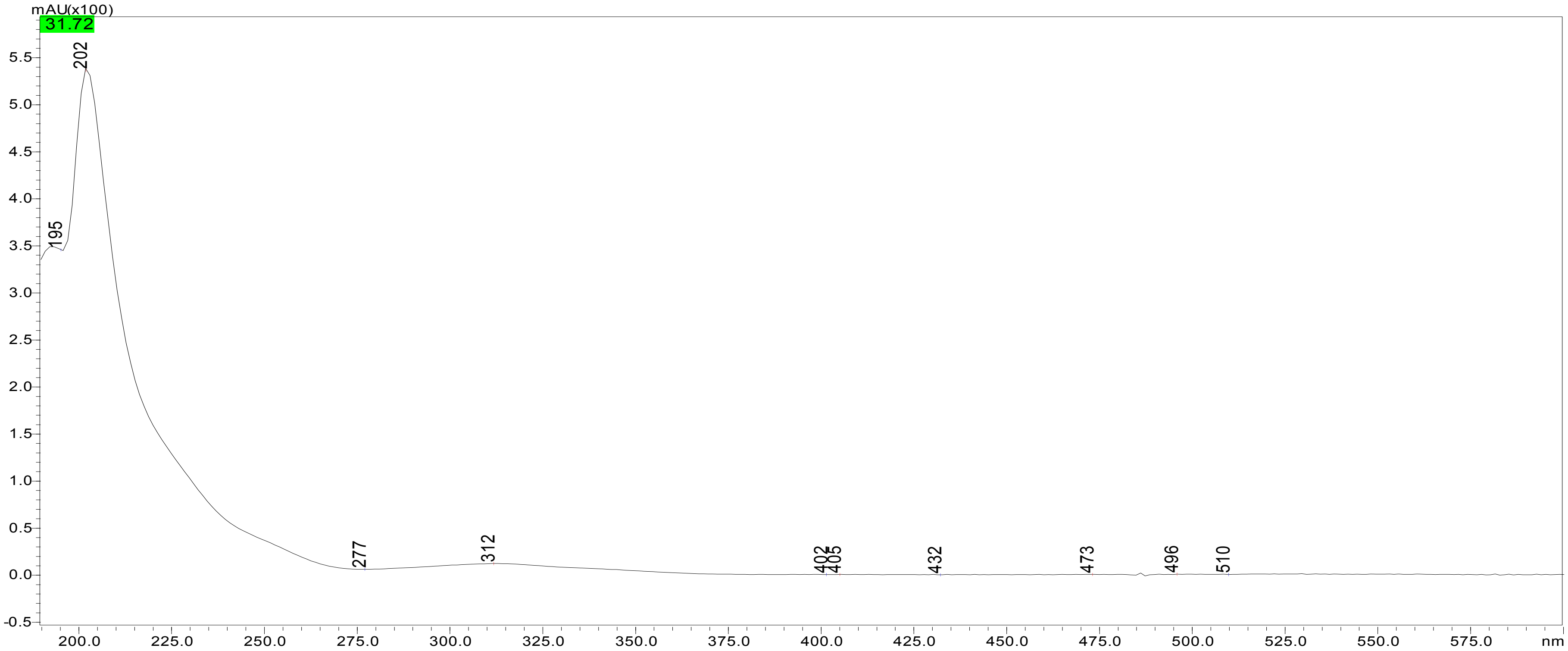

Figure S79. ^{1}H NMR spectrum of 7 in DMSO-d_6

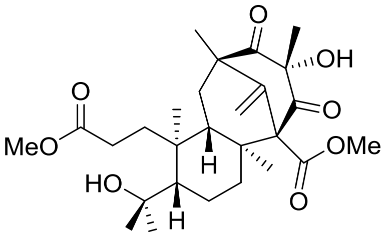

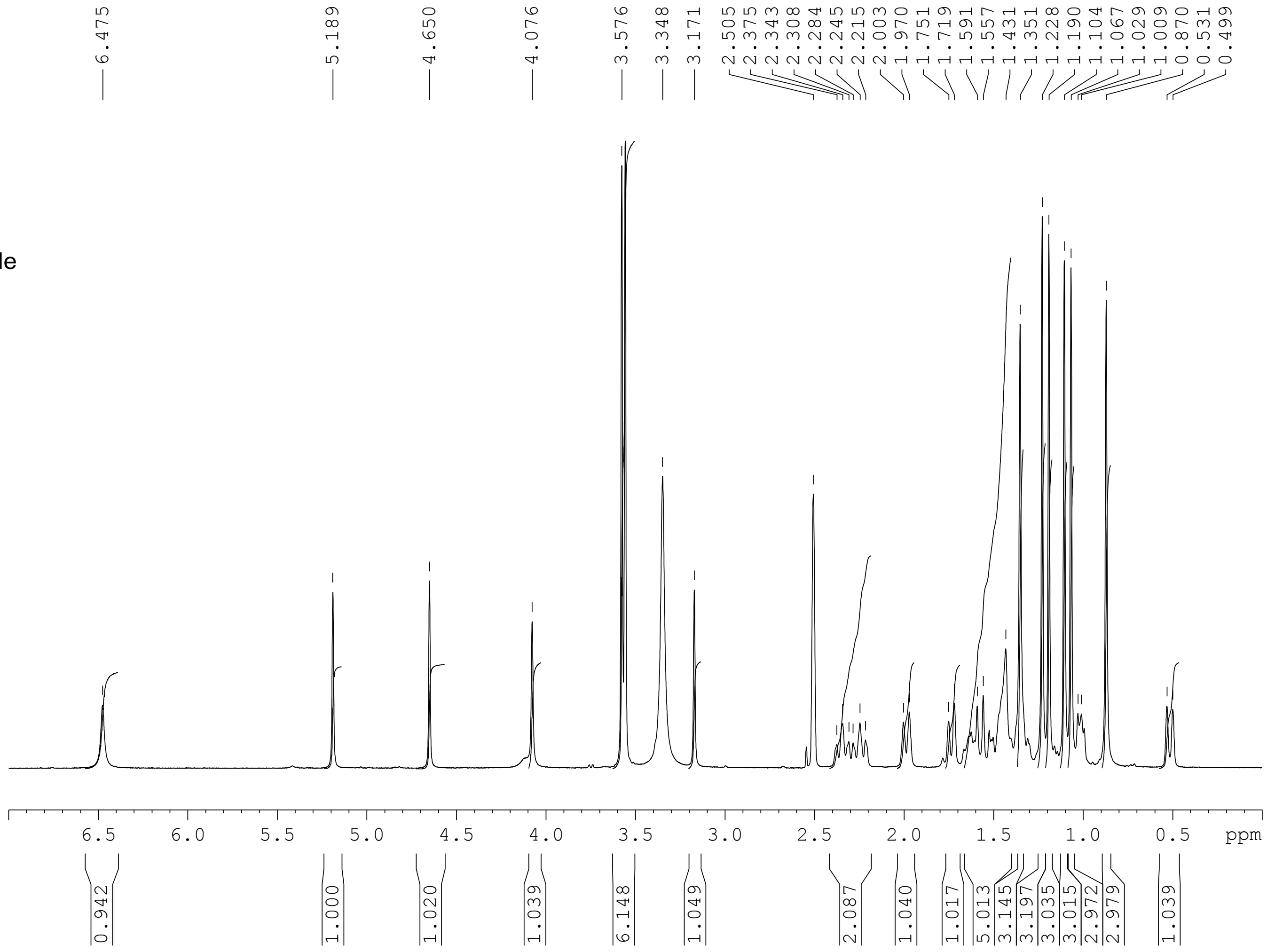

Figure S80. ^{13}C NMR spectra of 7 in DMSO-d_6

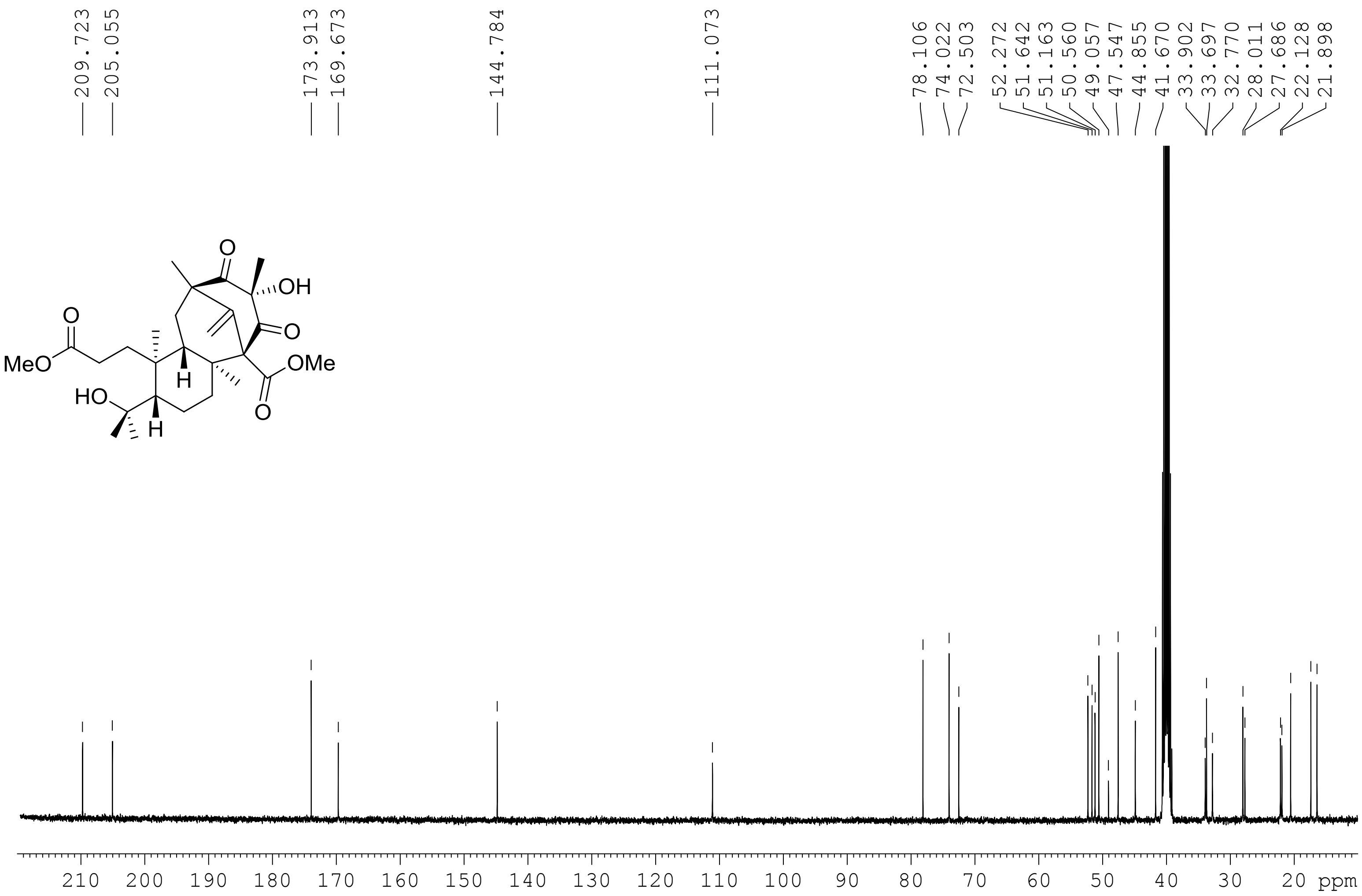

Figure S81. DEPT spectra of 7 in DMSO-d_6

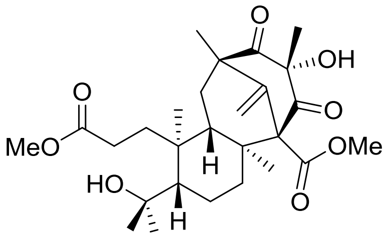

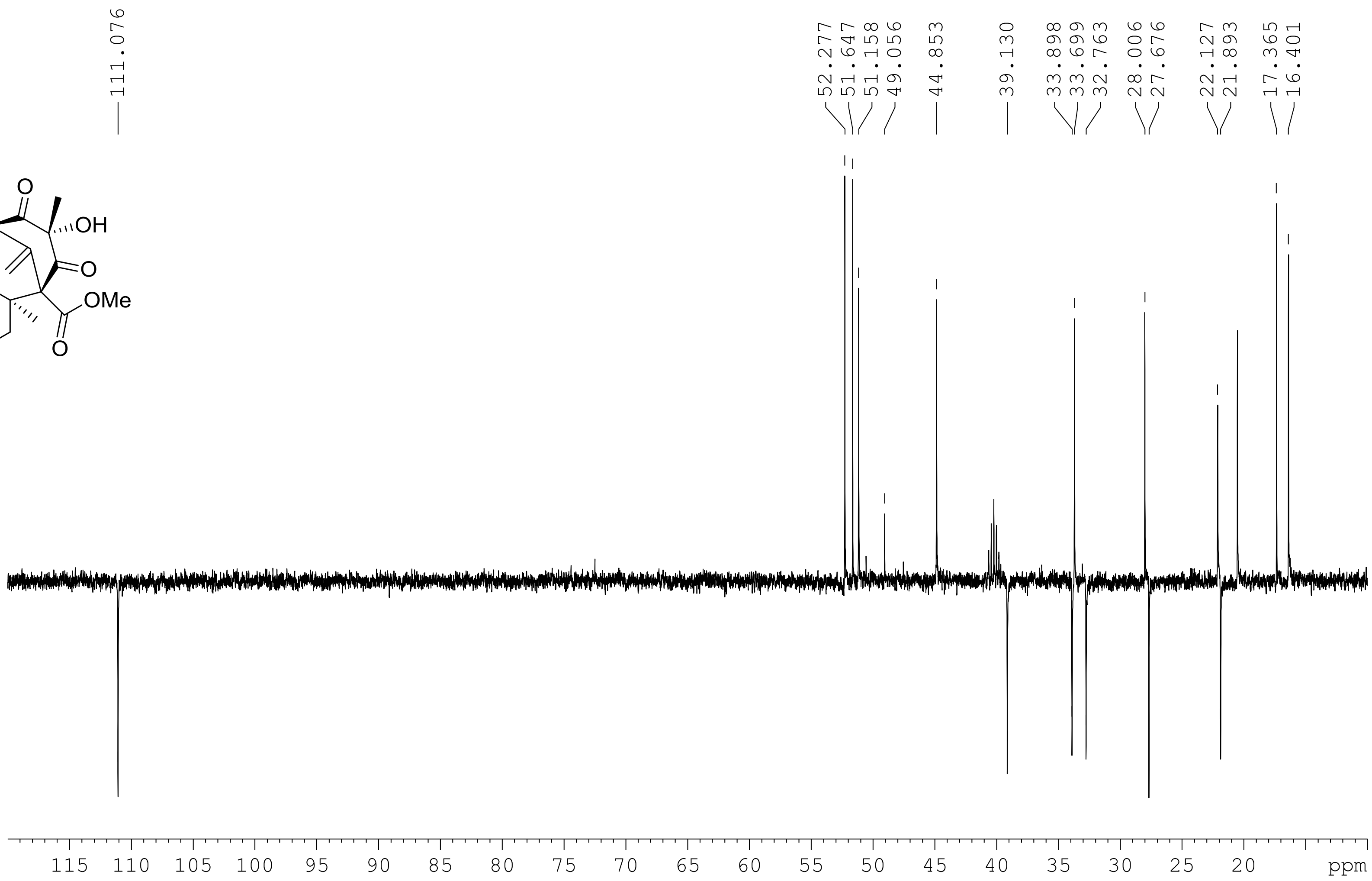

Figure S82. ^{1}H-^{1}H COSY spectrum of 7 in DMSO-*d*6

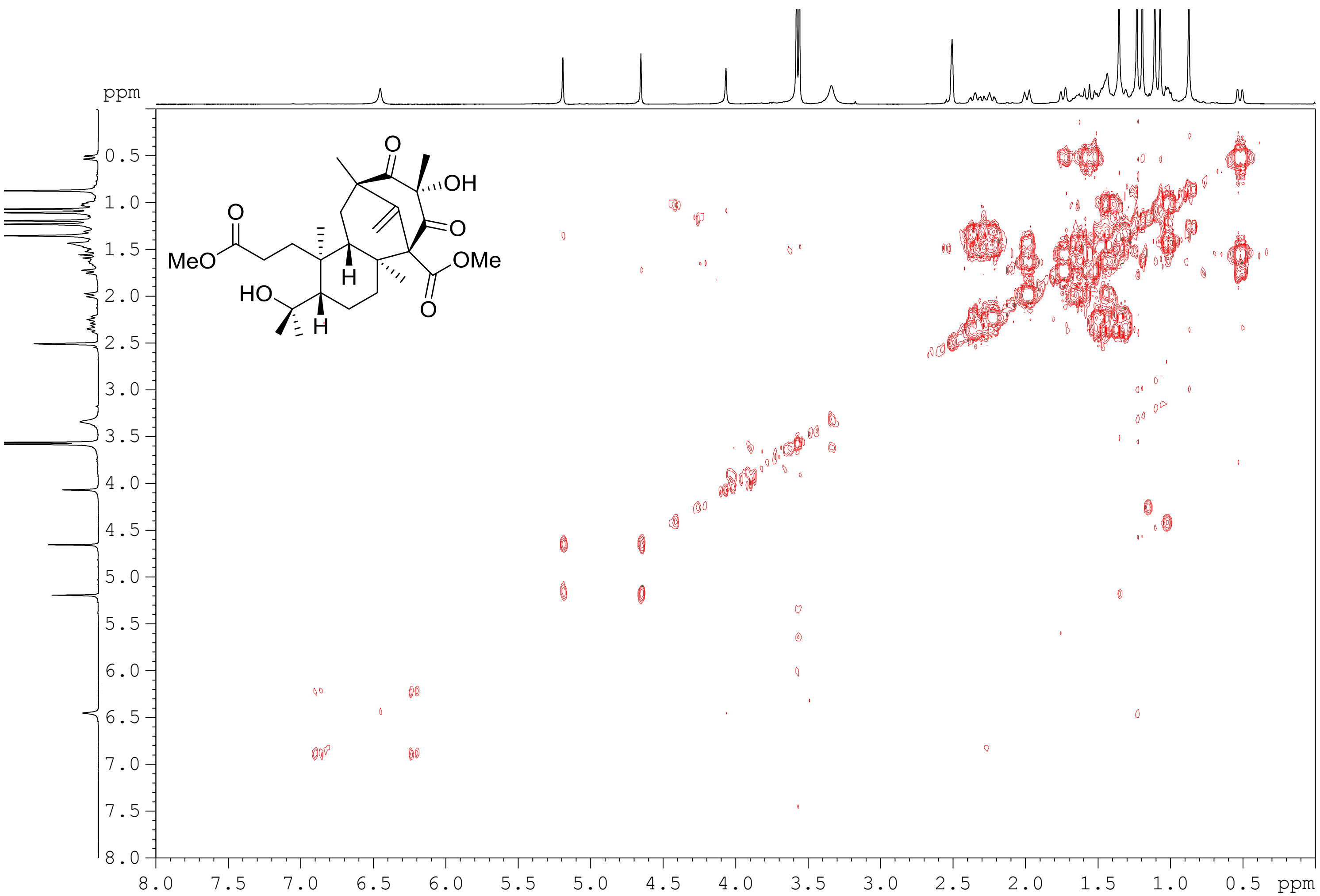

Figure S83. HSQC spectrum of 7 in DMSO-*d*6

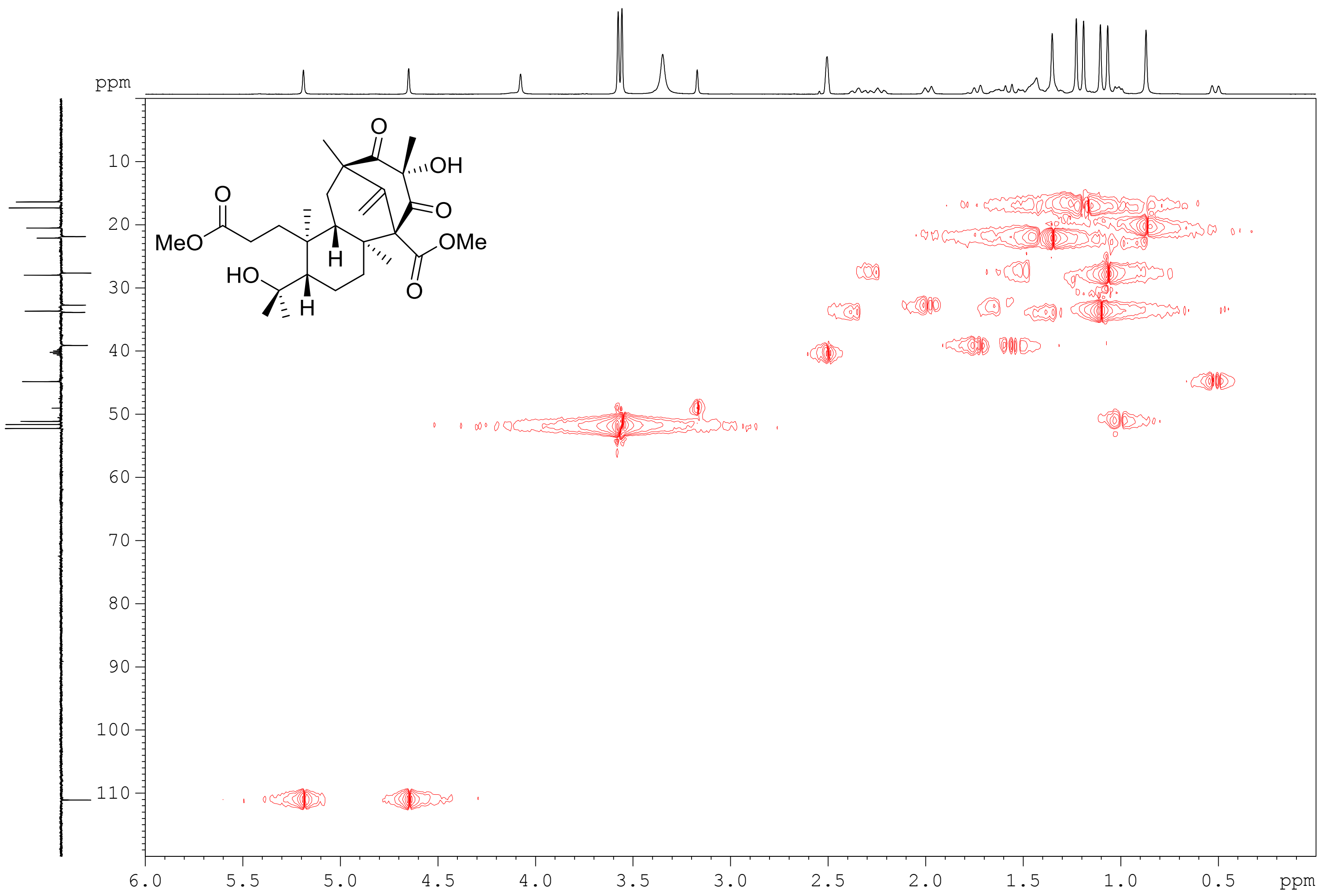

Figure S84. HMBC spectrum of 7 in DMSO-*d*6

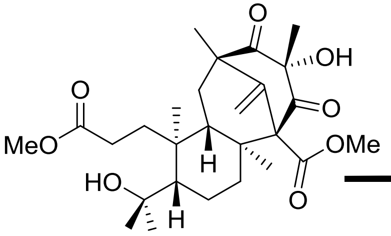

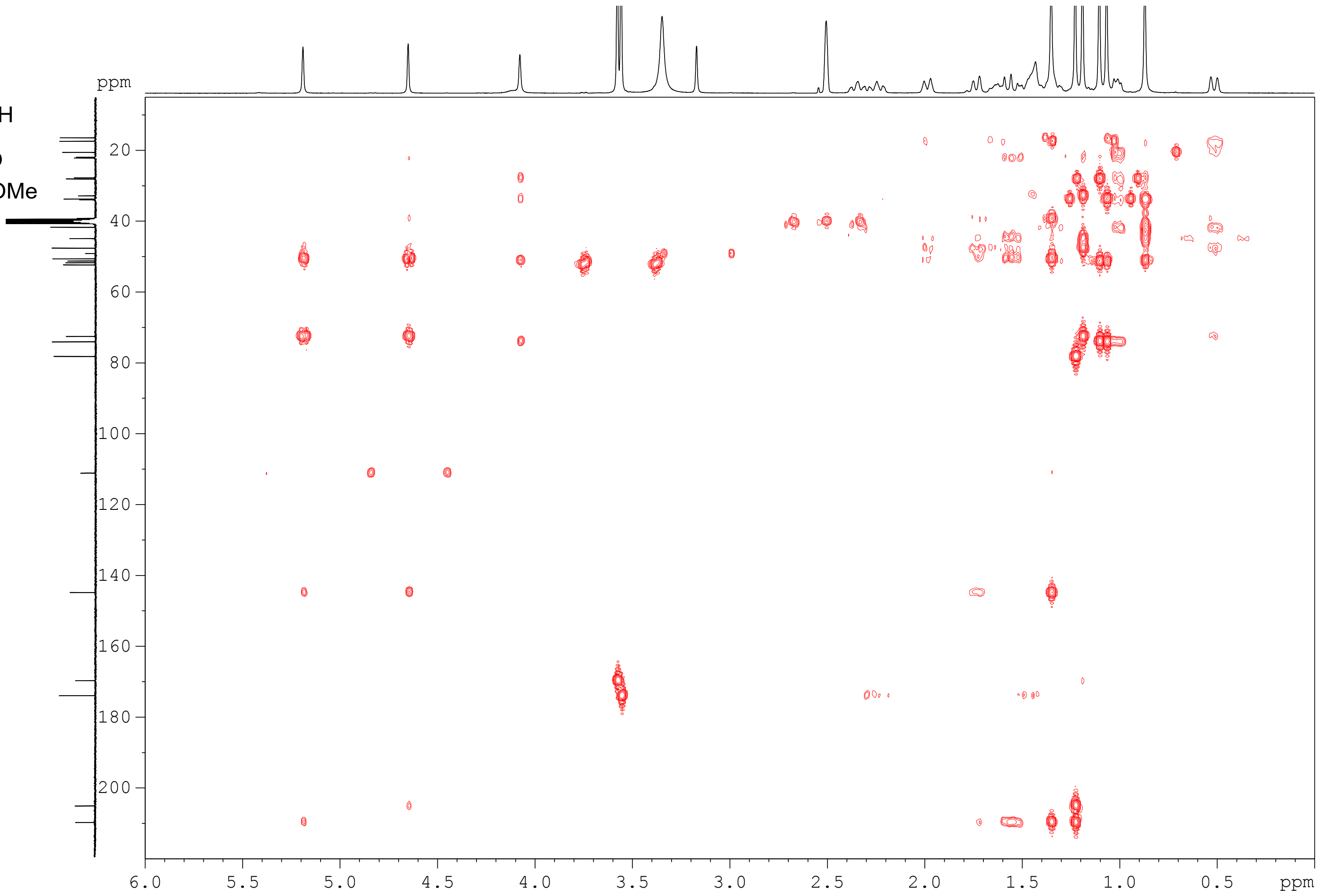

Figure S85. NOESY spectrum of 7 in DMSO-d_6

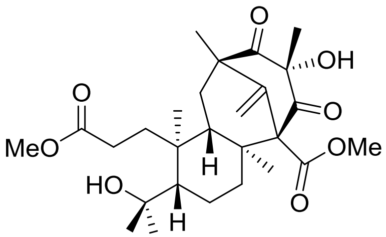

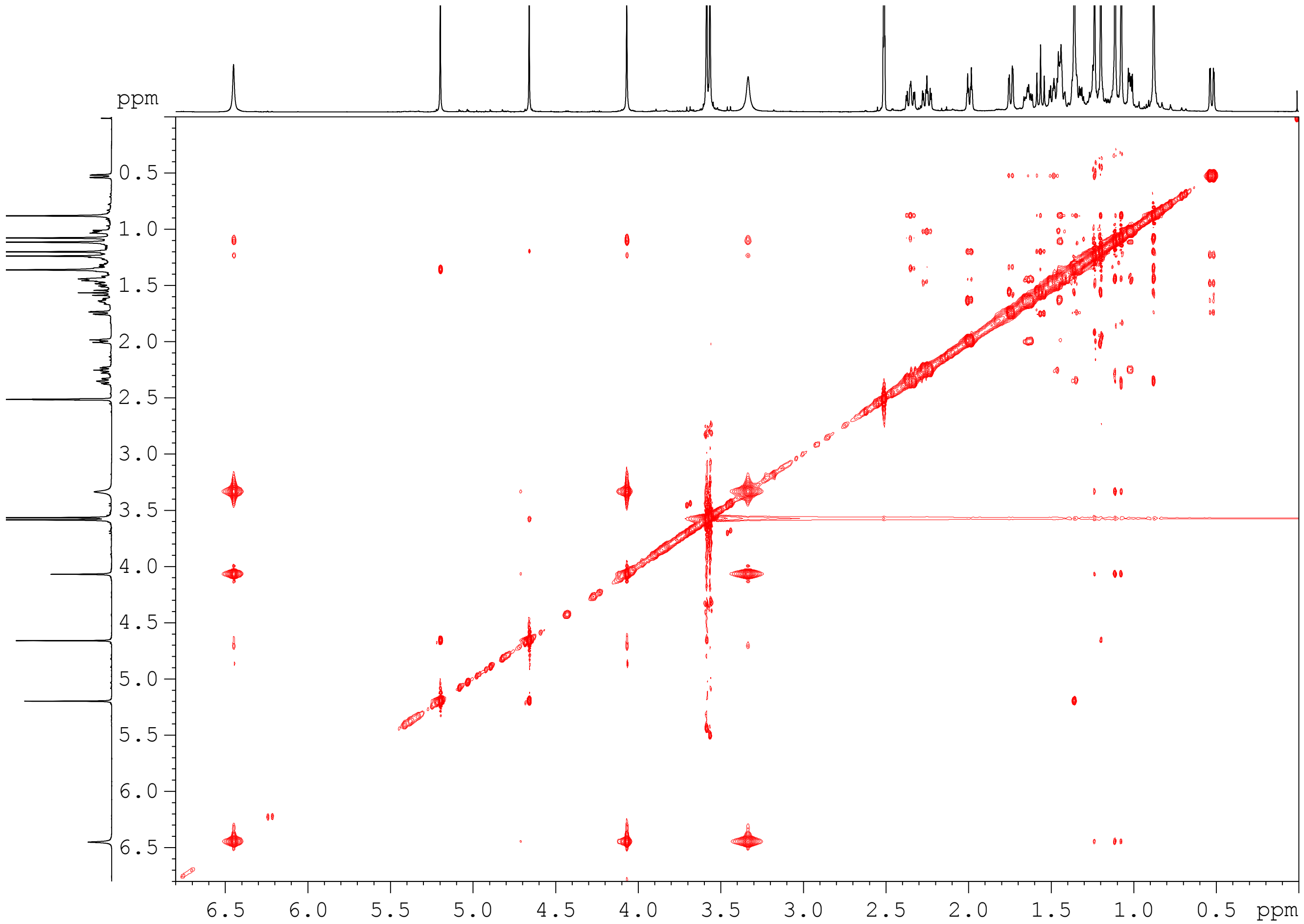

Figure S86. HR-ESIMS spectrum of 8

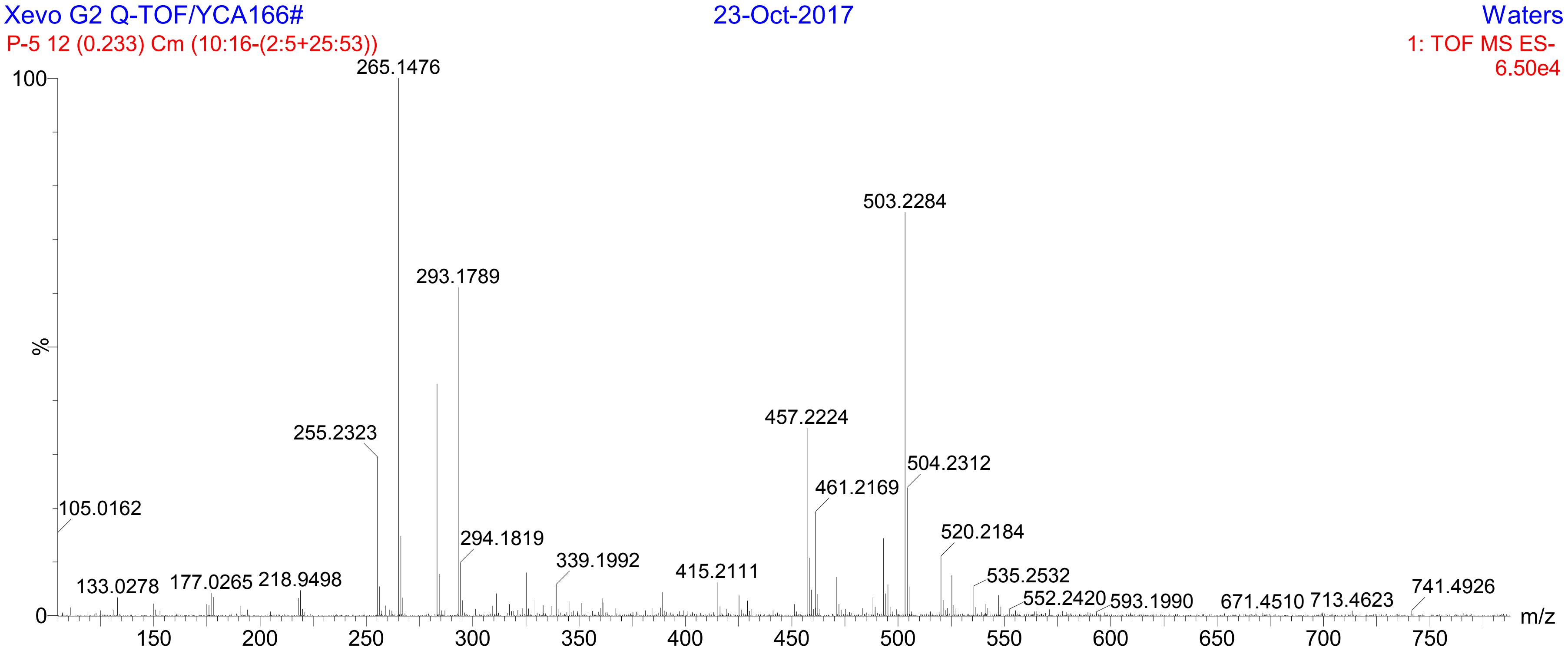

Mass	Calc. Mass	mDa	PPM	DBE	i-FIT	Norm	Conf(%)	Formula
457.2224	457.2226	-0.2	-0.4	10.5	93.4	0.740	47.70	$C_{26}H_{33}O_7$

Figure S87. ^{1}H NMR spectrum of 8 in DMSO-d_6

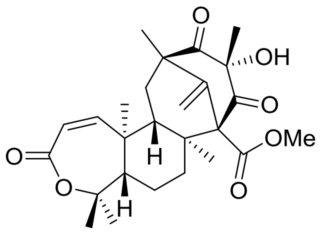

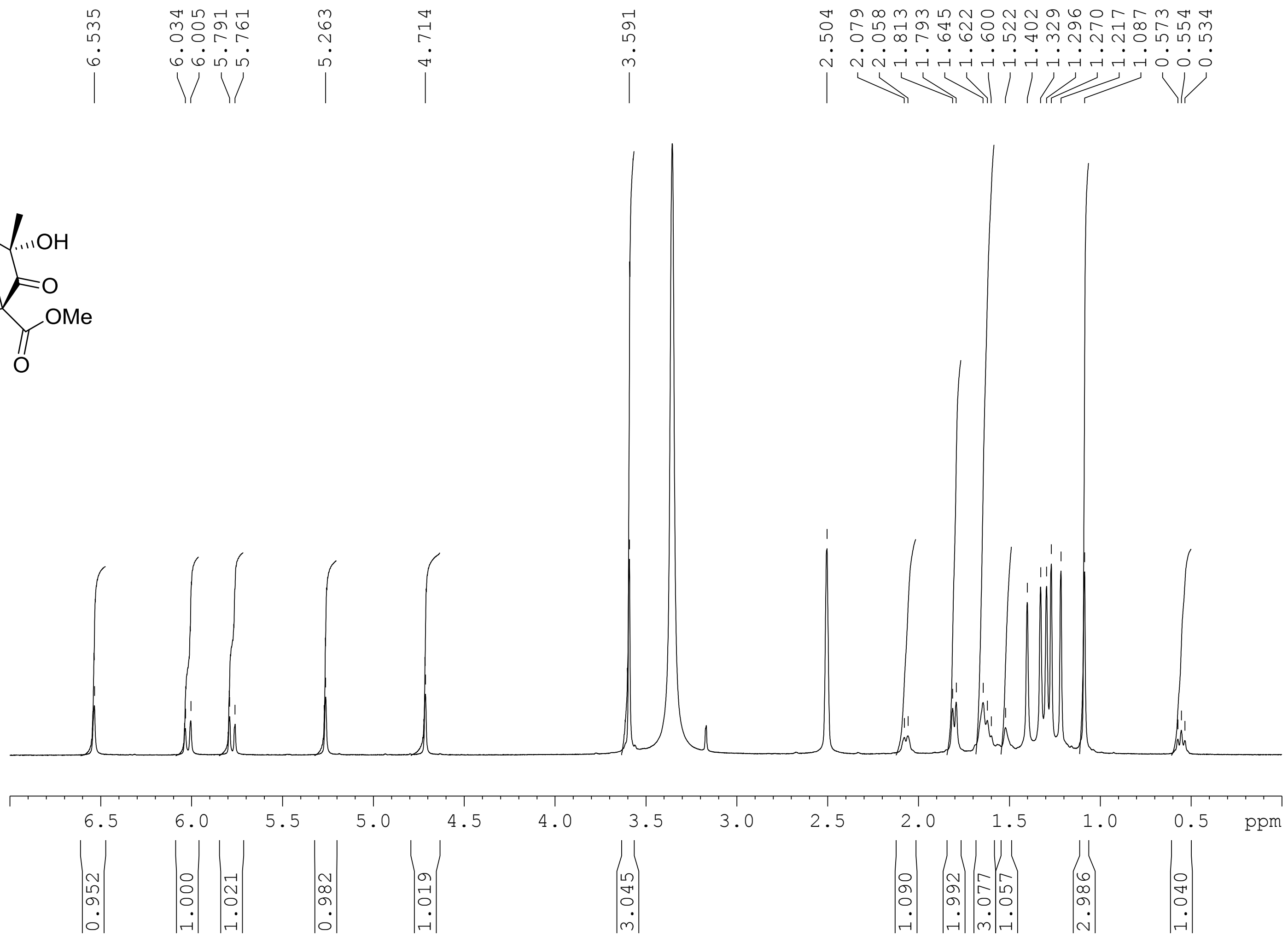

Figure S88. ^{13}C NMR spectra of 8 in DMSO-d_6

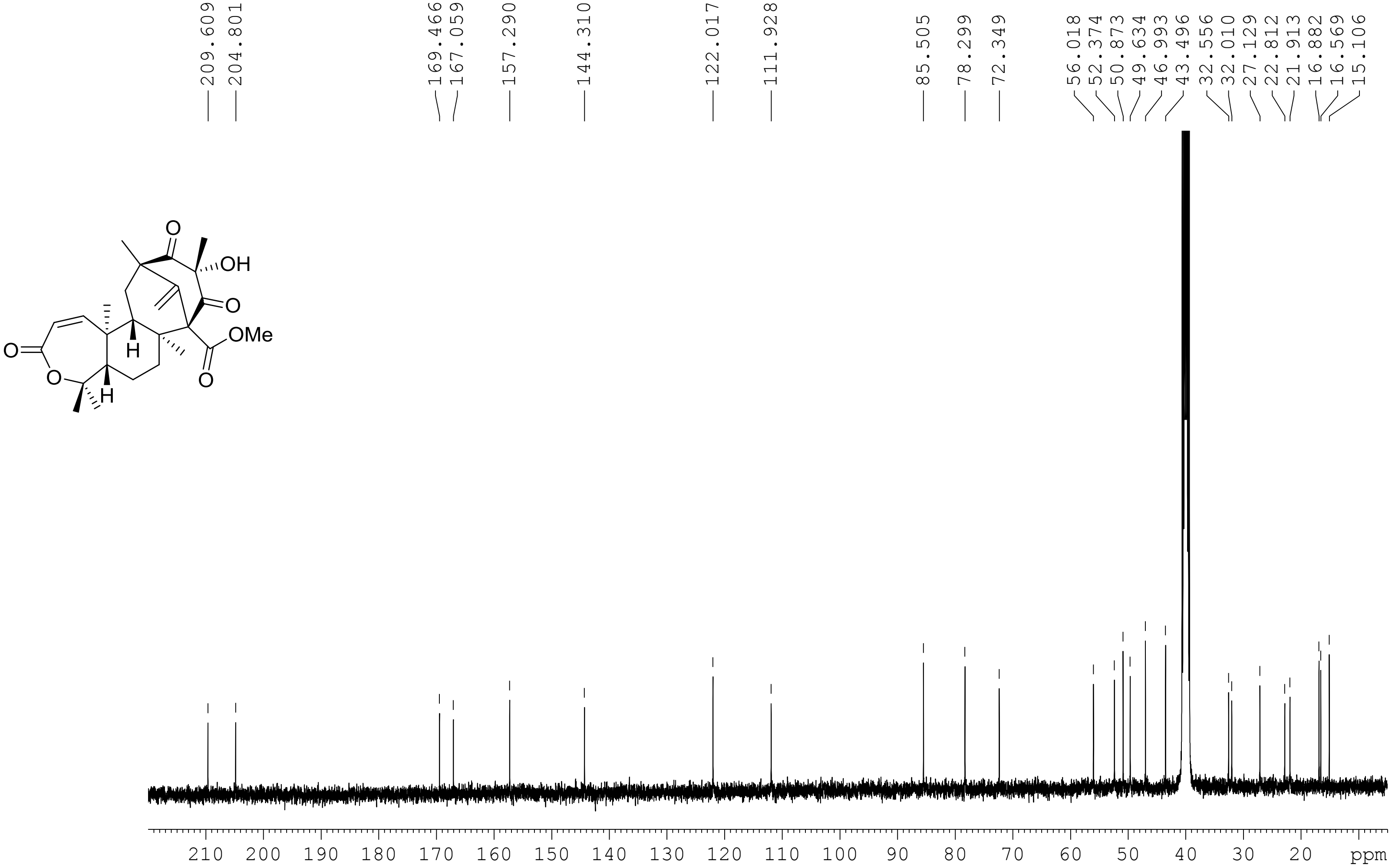

Figure S89. DEPT spectra of 8 in DMSO-d_6

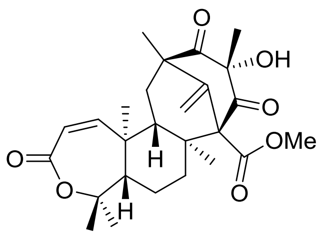

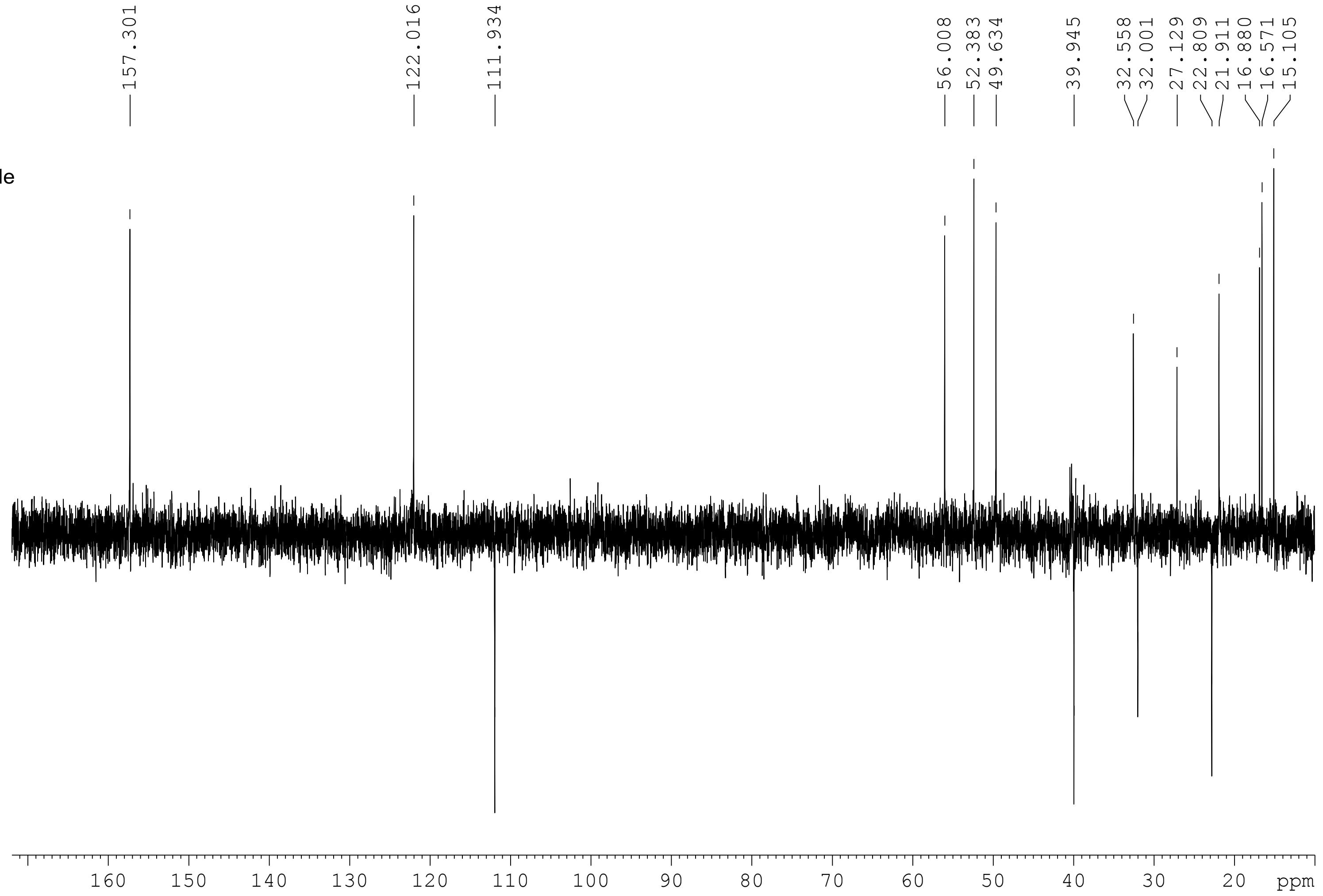

Figure S90. ^1H-^1H **COSY spectrum of 8 in DMSO-** d_6

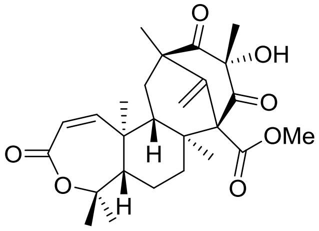

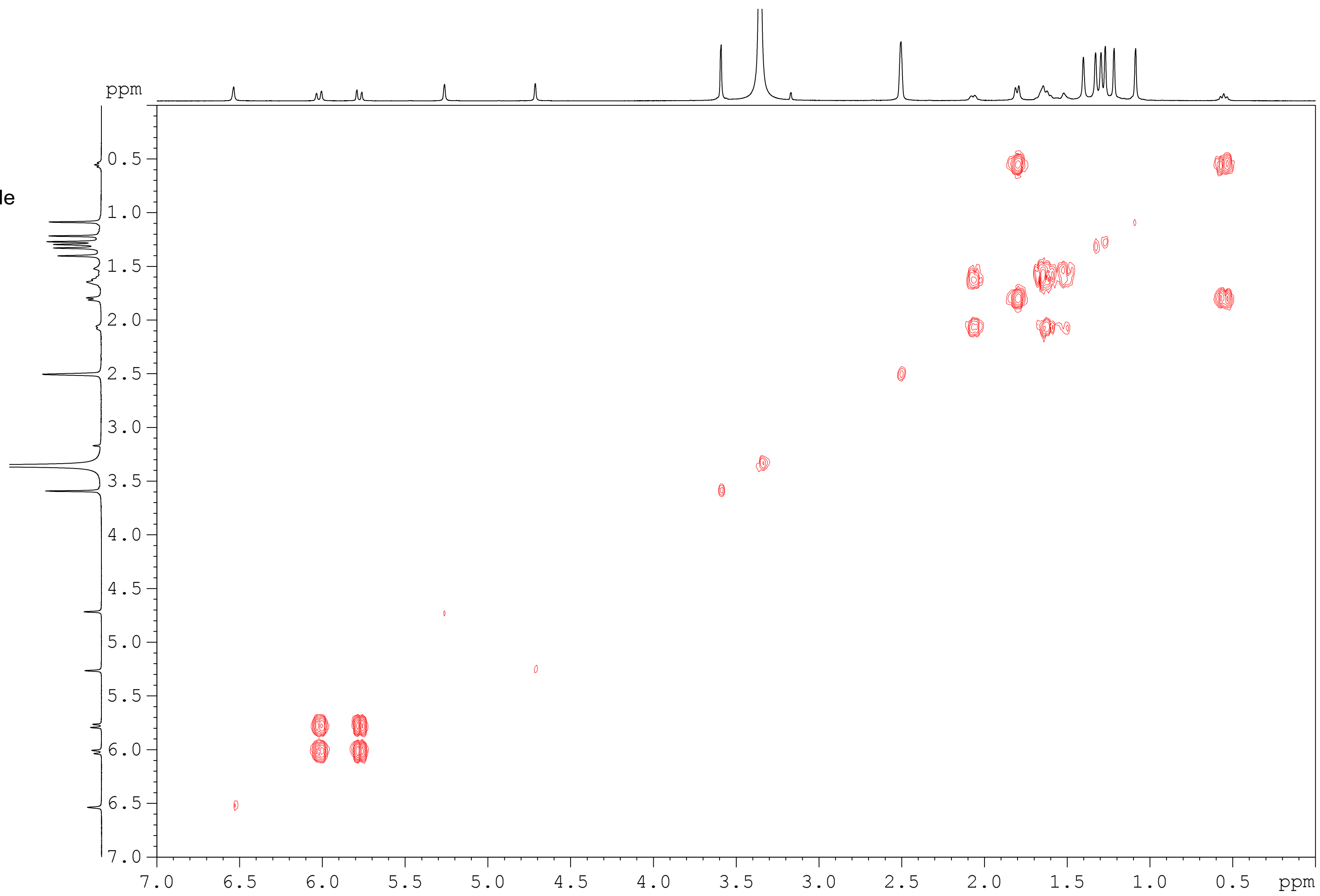

Figure S91. HSQC spectrum of 8 in DMSO-d_6

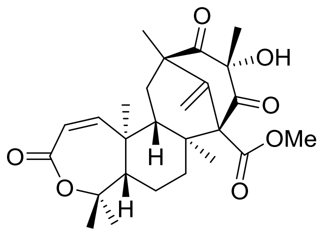

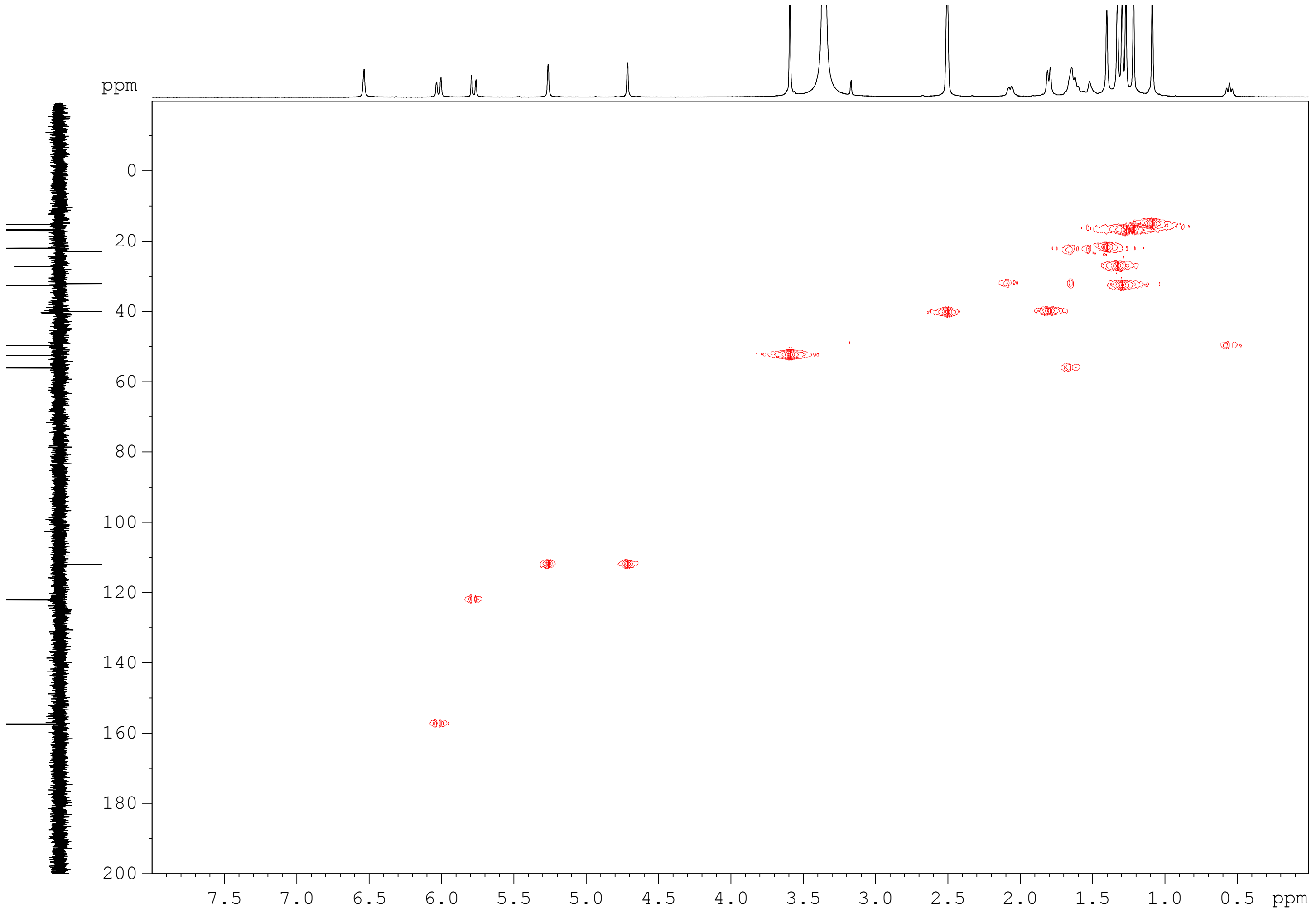

Figure S92. HMBC spectrum of 8 in DMSO-d_6

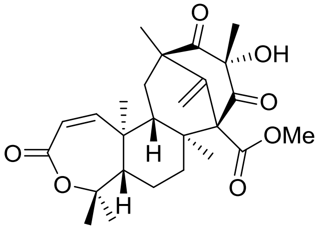

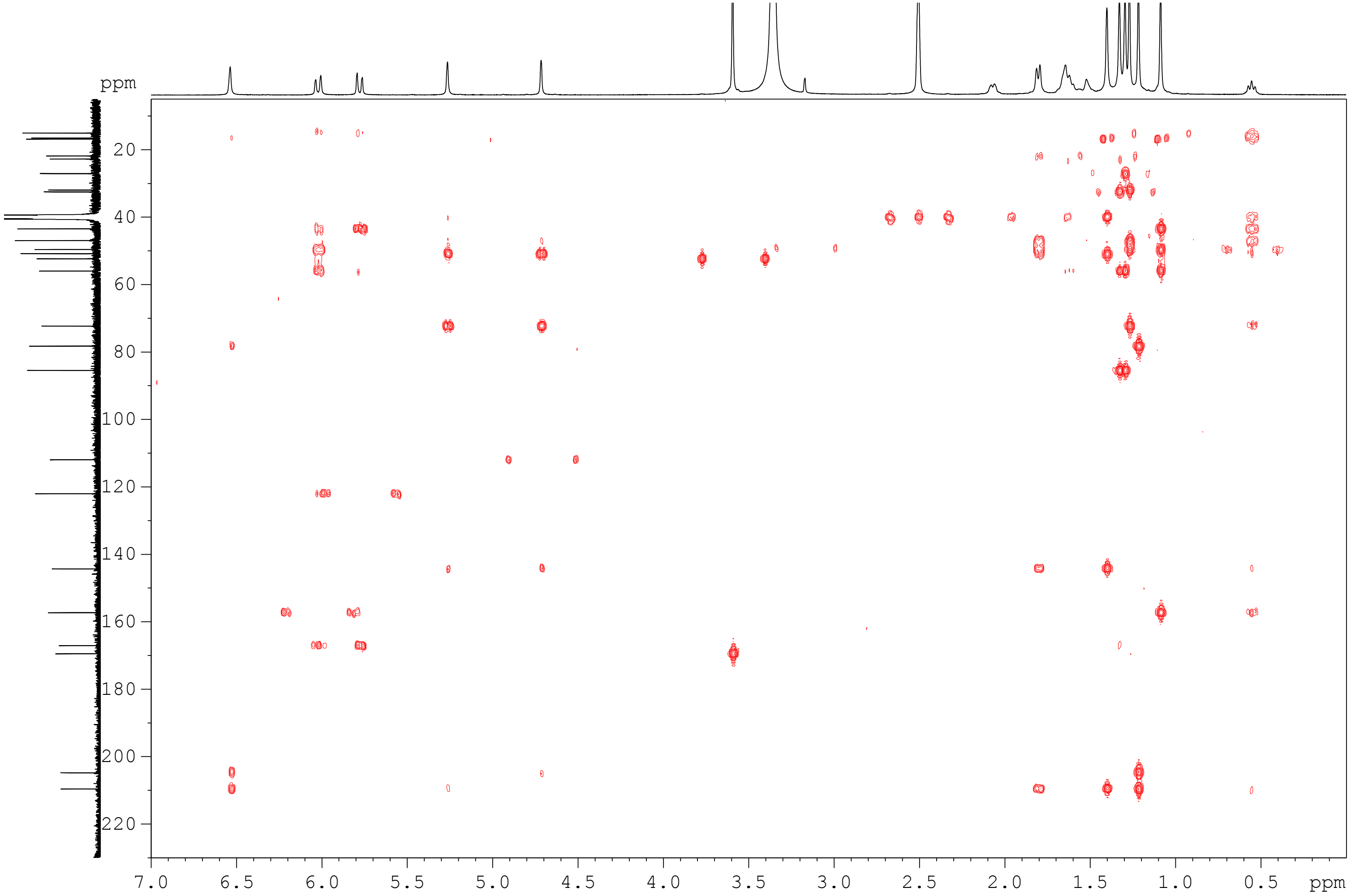

X-ray crystallographic data of 8

Empirical formula	$C_{26}H_{34}O_7$
Formula weight	458.53
Temperature	108.8 K
Crystal system	orthorhombic
Space group	$P2_12_12_1$
Unit cell dimensions	
	a = 8.2880(2) Å α = 90°.
	b = 13.9972(3) Å β = 90°.
	c = 19.4914(5) Å γ = 90°.
Volume	2261.18(10) $Å^3$
Z	4
Density (calculated)	1.347 mg/m^3
Absorption coefficient	0.794 mm^{-1}
F(000)	984
Crystal size	0.240 × 0.180 × 0.080 mm^3
Theta range for data collection	7.776 o 142.292°
Index ranges	$-10 \leq h \leq 7$, $-15 \leq k \leq 16$, $-23 \leq l \leq 23$
Reflections collected	10851
Independent reflections	4296 [R(int) = 0.0305]
Absorption correction	Semi-empirical from equivalents
Refinement method	Full-matrix least-squares on F^2
Data / restraints / parameters	4296 /0/ 306
Goodness-of-fit on F^2	1.039
Final R indices [I>2sigma(I)]	R1 = 0.0380 wR2 = 0.0994
R indices (all data)	R1 = 0.0403, wR2 = 0.1017
Absolute structure parameter	-0.16(11)
Largest diff. peak and hole	0.426 /-0.210 e.$Å^{-3}$

Figure S93. X-ray structure of 8

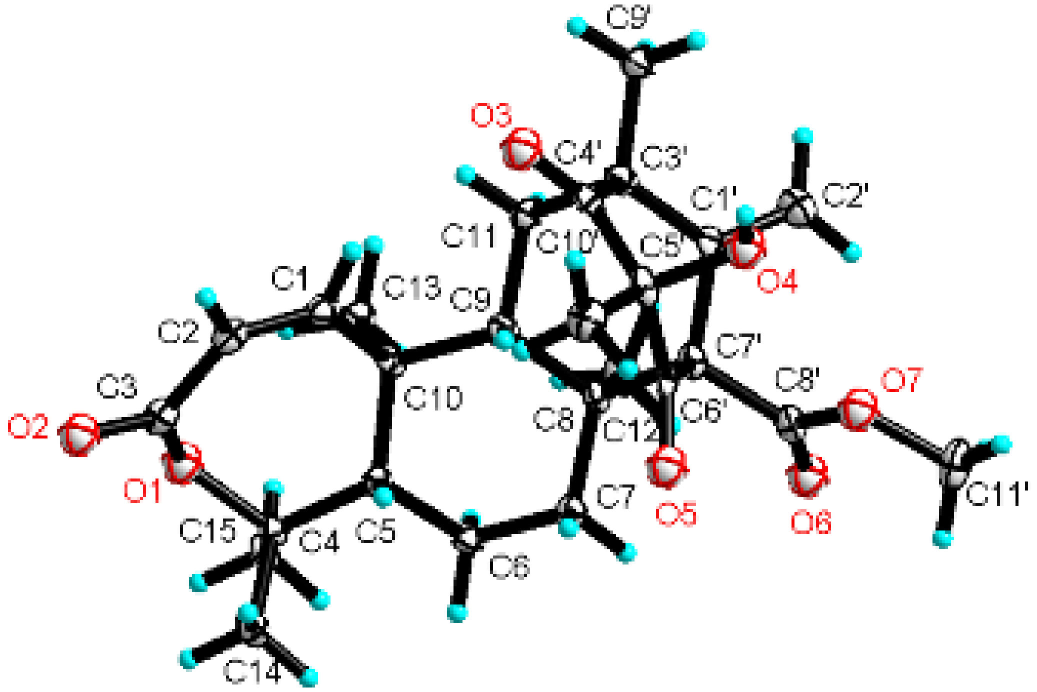

Figure S94. LC/ESI-MS analysis of 4, 5 and 7

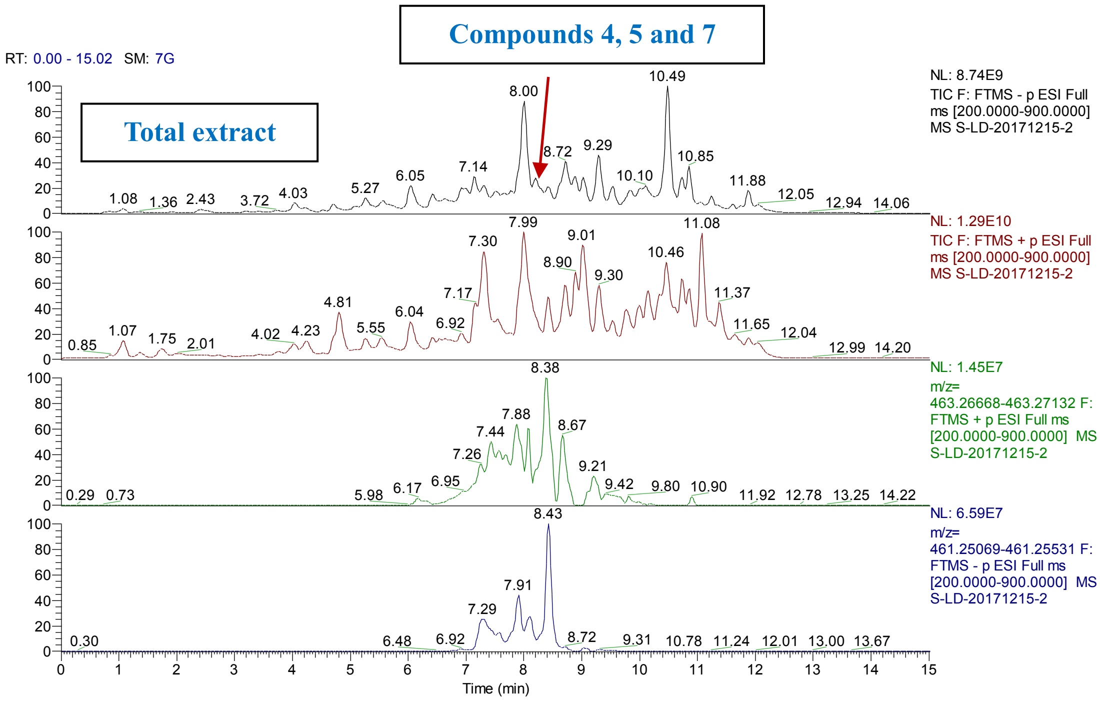

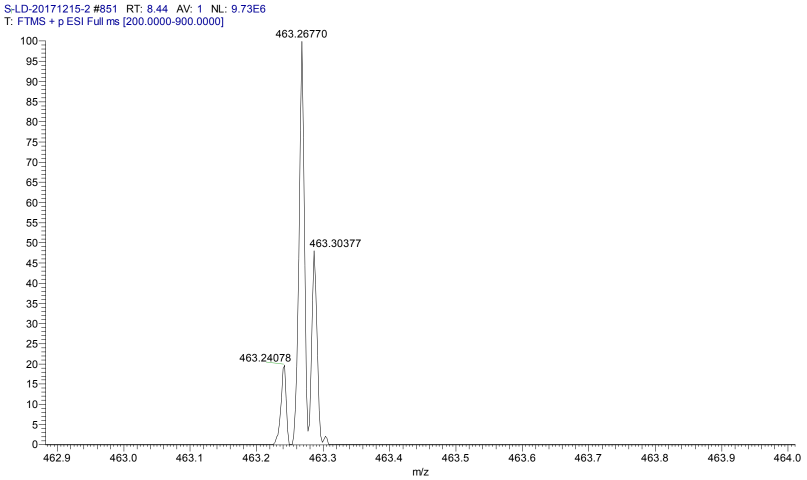
S-LD-20171215-2 #851 RT: 8.44 AV: 1 NL: 9.73E6
T: FTMS + p ESI Full ms [200.0000-900.0000]
463.26770
463.30377
463.24078
m/z

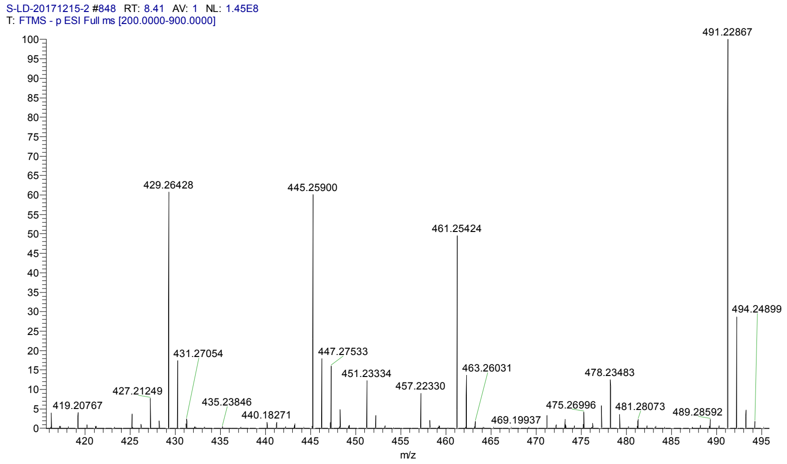
S-LD-20171215-2 #848 RT: 8.41 AV: 1 NL: 1.45E8
T: FTMS - p ESI Full ms [200.0000-900.0000]
491.22867
429.26428
445.25900
461.25424
494.24899
431.27054
447.27533
451.23334
463.26031
478.23483
427.21249
457.22330
419.20767
435.23846
440.18271
469.19937
475.26996
481.28073
489.28592
m/z

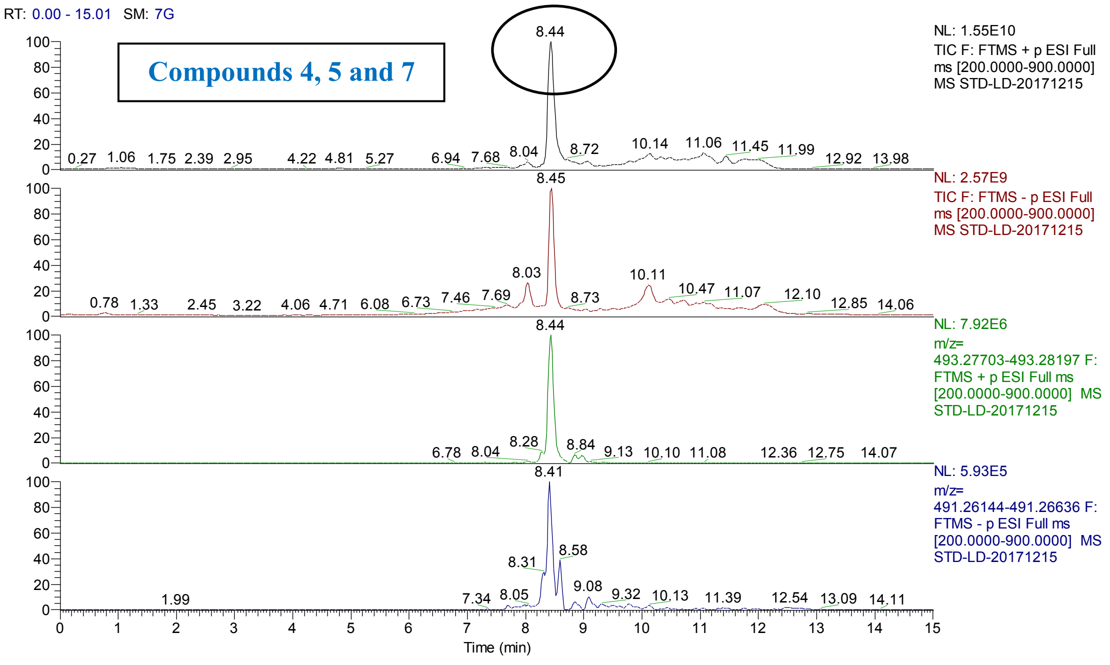
RT: 0.00 - 15.01 SM: 7G
Compounds 4, 5 and 7
NL: 1.55E10
TIC F: FTMS + p ESI Full ms [200.0000-900.0000] MS STD-LD-20171215
NL: 2.57E9
TIC F: FTMS - p ESI Full ms [200.0000-900.0000] MS STD-LD-20171215
NL: 7.92E6
m/z= 493.27703-493.28197 F: FTMS + p ESI Full ms [200.0000-900.0000] MS STD-LD-20171215
NL: 5.93E5
m/z= 491.26144-491.26636 F: FTMS - p ESI Full ms [200.0000-900.0000] MS STD-LD-20171215
Time (min)

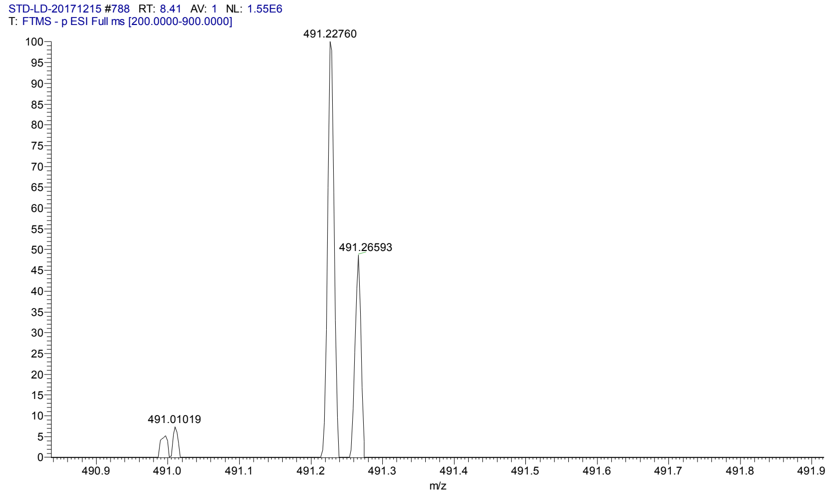
STD-LD-20171215 #788 RT: 8.41 AV: 1 NL: 1.55E6
T: FTMS - p ESI Full ms [200.0000-900.0000]
491.22760
491.26593
491.01019
m/z

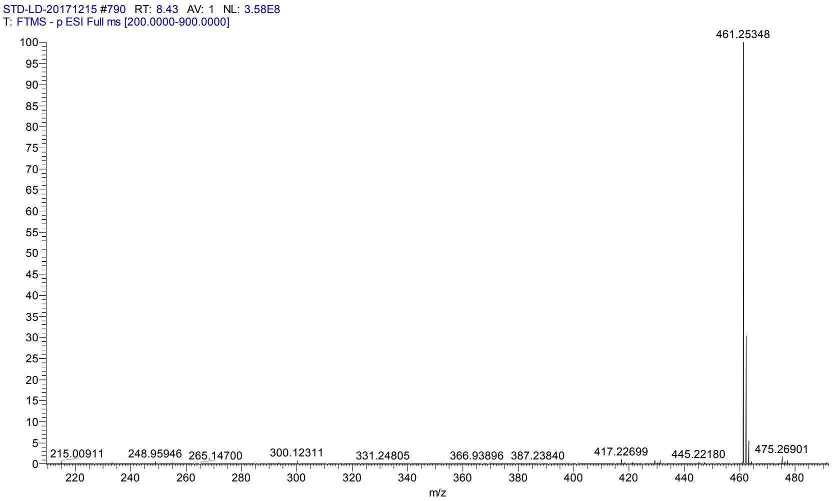
STD-LD-20171215 #790 RT: 8.43 AV: 1 NL: 3.58E8
T: FTMS - p ESI Full ms [200.0000-900.0000]
461.25348
215.00911
248.95946
265.14700
300.12311
331.24805
366.93896
387.23840
417.22699
445.22180
475.26901
m/z
